# Small Molecule Ligands of the BET-like Bromodomain, *Sm*BRD3, Affect *Schistosoma mansoni* Survival, Oviposition, and Development

**DOI:** 10.1021/acs.jmedchem.3c01321

**Published:** 2023-12-04

**Authors:** Matthias Schiedel, Darius J. B. McArdle, Gilda Padalino, Anthony K. N. Chan, Josephine Forde-Thomas, Michael McDonough, Helen Whiteland, Manfred Beckmann, Rosa Cookson, Karl F. Hoffmann, Stuart J. Conway

**Affiliations:** † Department of Chemistry, Chemistry Research Laboratory, University of Oxford, Mansfield Road, Oxford OX1 3TA, U.K.; ‡ The Department of Life Sciences (DLS), Aberystwyth University, Wales SY23 3DA, U.K.; § GlaxoSmithKline R&D, Stevenage, Hertfordshire SG1 2NY, U.K.; ∥ Department of Chemistry & Biochemistry, University of California Los Angeles, 607 Charles E. Young Drive East, P.O. Box 951569, Los Angeles, California 90095-1569, United States

## Abstract

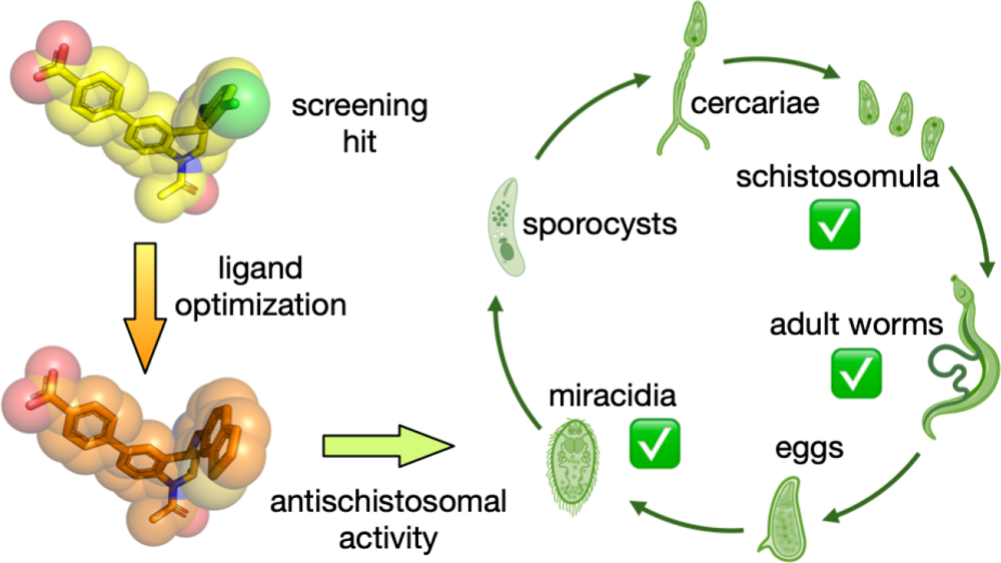

Schistosomiasis is a disease affecting >200 million people worldwide, but its treatment relies on a single agent, praziquantel. To investigate new avenues for schistosomiasis control, we have conducted the first systematic analysis of bromodomain-containing proteins (BCPs) in a causative species, *Schistosoma mansoni*. Having identified 29 putative bromodomains (BRDs) in 22 *S. mansoni* proteins, we selected *Sm*BRD3, a tandem BRD-containing BCP that shows high similarity to the human bromodomain and extra terminal domain (BET) family, for further studies. Screening 697 small molecules identified the human BET BRD inhibitor I-BET726 as a ligand for *Sm*BRD3. An X-ray crystal structure of I-BET726 bound to the second BRD of *Sm*BRD3 [*Sm*BRD3(2)] enabled rational design of a quinoline-based ligand (**15**) with an ITC *K*_d_ = 364 ± 26.3 nM for *Sm*BRD3(2). The ethyl ester pro-drug of compound **15** (compound **22**) shows substantial effects on sexually immature larval schistosomula, sexually mature adult worms, and snail-infective miracidia in *ex vivo* assays.

## Introduction

**Figure 1:**
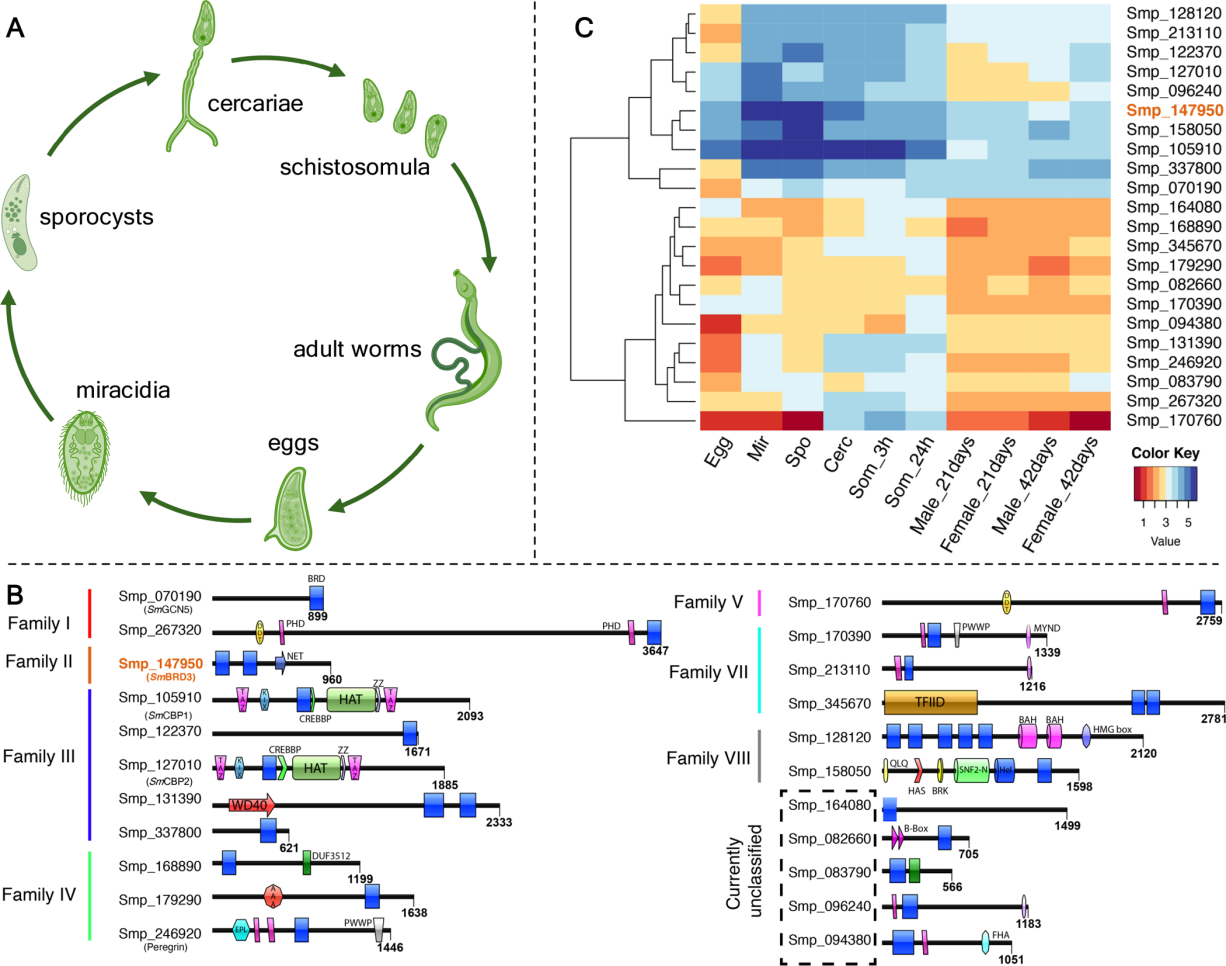
(A) Simplified cartoon of the *S. mansoni* lifecycle. (B) Domain architecture representation of the 22 putative *S. mansoni* BCPs classified by family (further defined in the Methods section).^16^ Where no family can be defined, the BCP is labeled “currently unclassified”. Gene ID (Smp; according to the *S. mansoni* v7 genome annotation; IDs remain stable in the v10 assembly), common name (in parentheses) and total protein length (in number of amino acids–aa) are all indicated. (C) Heat map illustration of *bcp* abundances across the *S. mansoni* lifecycle (derived from RNA-Seq meta data); *Sm*BRD3 (Smp_147950) is highlighted in orange. Blue colors indicate higher abundance and red colors indicate lower abundance. Abbreviations: Mir: miracidia; Spo: sporocyst; Cerc: cercaria; Som: schistosomula.

Epigenetic processes link changes in gene expression that do not result from alterations in the genetic code to phenotypic diversity in a population. The blood fluke parasite *Schistosoma mansoni*, which is responsible for the devastating neglected tropical disease schistosomiasis, adopts phenotypically diverse developmental forms as it progresses through its complex lifecycle (Figure 1A). However, the molecular processes that control these changes are poorly understood.^1^ As these phenotypic changes must occur without alteration of the parasite’s inherited genome, the involvement of epigenetic processes and cellular machinery is certain.^2^ At the molecular level, covalent modification of DNA and histone proteins modulate chromatin structure and recruit transcriptional machinery to modulate gene expression.^3^ The proteins that add these modifications are called epigenetic writers and those that remove them are called epigenetic erasers. A third class of proteins, known as epigenetic readers, bind to the covalent modifications on DNA or histones and recruit transcriptional machinery to that site.^4^ Bromodomain (BRD)-containing proteins (BCPs) are a particular class of epigenetic readers that bind to acetylated lysine (KAc) residues on histones, and many other proteins.^5^ Human BRDs are a therapeutic target for many oncology indications,^6^ but little is known about the role of BRDs in parasites,^7^ and almost nothing is known about their role in *S. mansoni*.^8−10^

Functional studies of schistosome BCPs will not only enrich our understanding of parasite development, but also reveal new targets for control of a disease that contributes to 200000 human deaths per year, increases the risk of developing certain types of cancers, modulates the immune system in infected individuals leading to reduced efficacy of vaccines, and is responsible for millions of disability adjusted life years lost in affected communities.^11^ Due to limited control options as well as its public health and zoonotic importance, the WHO has targeted schistosomiasis for elimination by 2030 in their most recent roadmap for ‘*Ending the Neglect to Attain the Sustainable Development Goals*’.^12^ Achieving this ambitious agenda will require basic investigations of schistosome biology to expose parasite vulnerabilities that, in turn, guide and target the development of novel therapeutic interventions.

Here, we report the first systematic study of BCPs in *S. mansoni*. Using a combination of sequence similarity and domain-based searches, applied previously to the characterization of schistosome histone methyltransferases and histone demethylases,^13^ we have identified 22 BCPs in *S. mansoni* containing 29 distinct BRDs. We have focused our investigation on *Sm*BRD3 (Smp_147950) due to its relatively high temporal expression when compared to most other *Sm*BCPs and similarity to the human bromodomain and extra terminal domain (BET) family of BCPs. *Sm*BRD3 shares 37.5% sequence identity with human BRD3 (*Hs*BRD3) and, similar to the BET BCPs, possesses two BRDs, *Sm*BRD3(1) and *Sm*BRD3(2). Using differential scanning fluorimetry (DSC) and isothermal titration calorimetry (ITC), we identified a number of human bromodomain ligands that bind to either *Sm*BRD3(1) or *Sm*BRD3(2). An X-ray crystal structure of I-BET726 bound to *Sm*BRD3(2) allowed the structure-based optimization of this compound series to give the quinoline-derived compound **15**, which has a *K*_d_ value of 364 ± 26.3 nM for *Sm*BRD3(2). In a variety of whole organism phenotypic assays, the ethyl ester prodrug of compound **15**, compound **22**, demonstrated profound effects on both larval schistosomula and sexually reproductive adult worms. Interestingly, compound **22** also prevents the developmental transformation of snail-infective miracidia into asexually proliferative sporocysts. This suggests a role for *Sm*BRD3 in the parasite developmental life cycle, mirroring the role of the BET bromodomains in humans.^14,15^ Our use of a structure- and target-based approach has enabled the rapid development of high affinity ligands for *Sm*BRD3. While further work is required to determine the selectivity of the compounds, the development of chemical tools with defined cellular targets paves the way for contemporary medicinal chemistry techniques to be applied to *S. mansoni*.

## Results

### Identification of 22 Putative Bromodomain-Containing Proteins in *S. mansoni*

**Figure 2:**
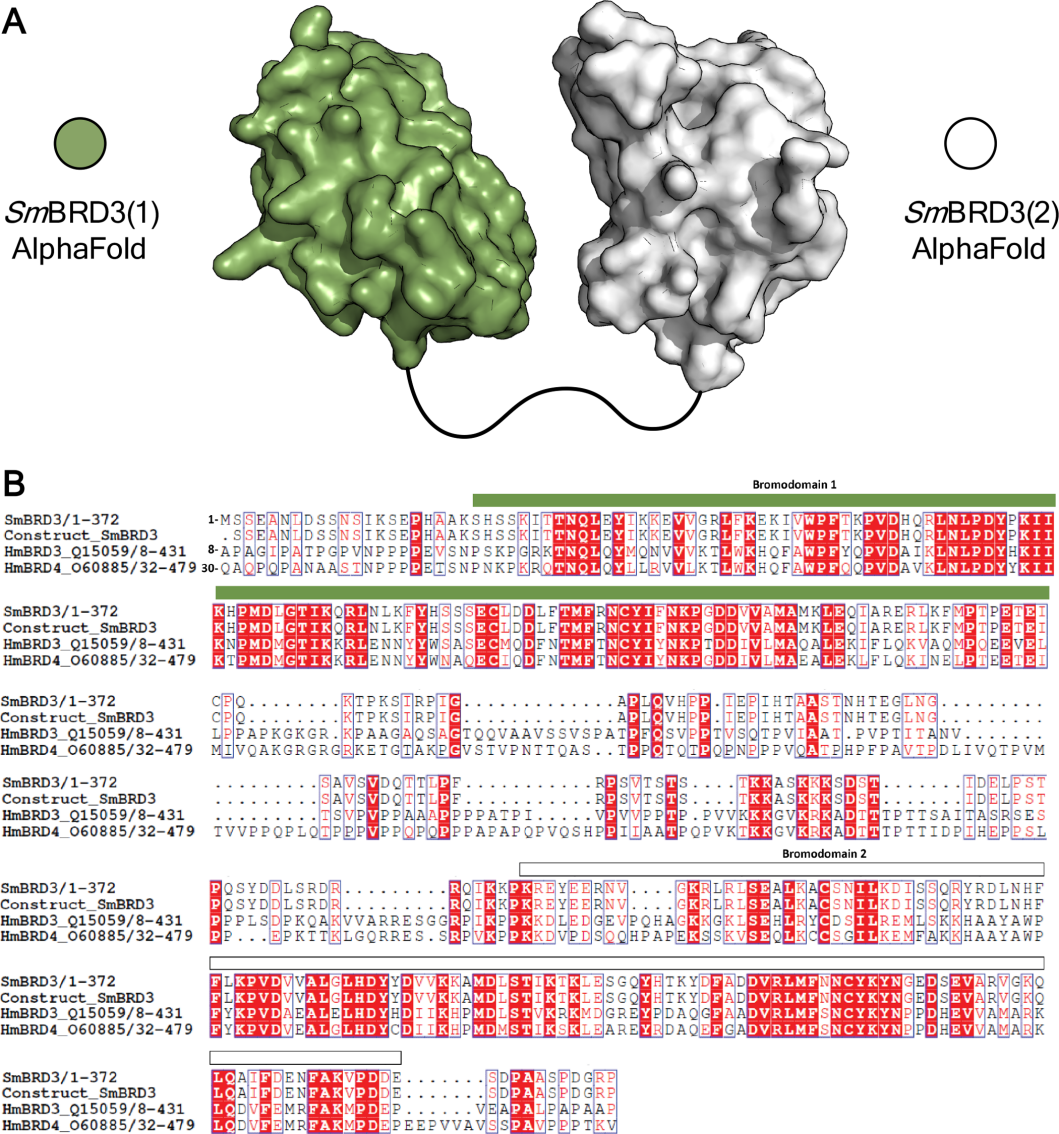
(A) Cartoon of the *Sm*BRD3(1) (green) and *Sm*BRD3(2) (white) structures predicted using AlphaFold.^21^ (B) Multiple sequence alignment of the *Sm*BRD3(1,2) construct, *Sm*BRD3, *Hs*BRD3, and *Hs*BRD4. *Sm*BRD3 has 36.64% and 34.69% sequence similarity with *Hs*BRD3 and *Hs*BRD4, respectively. The region identified as the first bromodomain is indicated with a green bar and the region identified as the second bromodomain is indicated with a white bar. Sequences were aligned using MUSCLE^22^ and the resulting multiple sequence alignment was visualized using ESPript 3 server.^23^ Vertical blue boxes indicate conserved residues, white letters in red boxes indicate strict identity and red letters in white boxes indicate similarity.

While BRDs have previously been identified in the *S. mansoni* histone acetyltransferases (HATs) *Sm*GCN5,^17^*Sm*CBP1 and *Sm*CBP2,^18,19^ there has been no systematic search for these protein modules in the parasite proteome more generally. Additionally, while there have been some studies in which human BRD ligands have been assessed for their phenotypic effects on *S. mansoni*,^20^ there has not been a rational design approach taken to identifying and optimizing ligands for *S. mansoni* BRDs. Using a combination of genome- and domain-based bioinformatic searches of the *S. mansoni* genome, we identified 29 putative BRDs found in 22 distinct BCPs (Figure 1B). This differentially expressed gene family (Figure 1C) includes the previously characterized *Sm*GCN5,^17^*Sm*CBP1 and *Sm*CBP2 proteins,^18,19^ but also a number of previously uncharacterized BCPs. One BCP, that is highly expressed throughout the *S. mansoni* lifecycle, possesses 37.5% sequence identity to human BRD3 (*Hs*BRD3) and 33.2% identity to human BRD4 (*Hs*BRD4); we have annotated this BCP as *S. mansoni* bromodomain-containing protein 3 (*Sm*BRD3; Smp_147950; UniProt: G4 V8 V7), in analogy to *Hs*BRD3. Sequence analysis shows that, similar to its human homologue, *Sm*BRD3 possesses two bromodomains [*Sm*BRD3(1) and *Sm*BRD3(2)] that are located toward the N-terminus of the protein (Figure 1B), and which are separated by approximately 100 residues (Figure 2).

### Expression of the *Sm*BRD3 Bromodomains

Extensive structural studies on the human BET BCPs (including *Hs*BRD3) have identified the residues that are important for its interactions with acetylated lysine (KAc) residues in target proteins. In *Hs*BRD4(1), these are Y97 and N140; N140 forms a hydrogen bond directly with KAc, while Y97 interacts with KAc through a water-mediated hydrogen bond.^24,25^ The primary sequence of *Sm*BRD(1) and *Sm*BRD3(2) indicates that both of these BRDs possess equivalent residues to Y97 and N140, [Y67 & N110 in *Sm*BRD3(1) and Y296 & N339 in *Sm*BRD3(2)], indicating that they are canonical BRDs and that they can bind to KAc. A second region of the BET BRDs that is important for binding to KAc, and small molecule ligands, is the WFP shelf. Named after the residues that define it,^26^ this region binds to the lipophilic chain of a second KAc residue when bound to chromatin (e.g., PDB code: 3UVX).^16^ The lipophilic nature of this region has been exploited in the design of BRD4 ligands, with many high affinity compounds interacting with the WPF shelf. Interestingly, while *Sm*BRD3(1) possesses the WPF residues (W56, P57, F58), these are replaced by an HFF motif (H280, F281, F282) in *Sm*BRD3(2) perhaps indicating the ability of this BRD to recognize different residues, proteins, and/or small molecules from *Sm*BRD3(1).

Development of biochemical/biophysical assays to identify *Sm*BRD3 BRD ligands required the recombinant expression of *Sm*BRD3(1), *Sm*BRD3(2), and a construct that contained both bromodomains and the residues that link them [*Sm*BRD3(1,2)]. While we were able to select and produce stable constructs of *Sm*BRD3(2) and *Sm*BRD3(1,2) in good yields (Figures S1–S5, Table S1), this was not possible for *Sm*BRD3(1). Analysis of the full protein structure predicted by AlphaFold indicates that the helix comprising residues 617–638 and the subsequent disordered residues 639–660 form intraprotein interactions in the region between *Sm*BRD3(1) and *Sm*BRD3(2) (Figure S6A). We propose that these interactions are required to stabilize *Sm*BRD3(1) (Figure S6B), and while they can be compensated for by other hydrophobic interactions in *Sm*BRD3(1,2), this is not possible in *Sm*BRD3(1) rendering it unstable when expressed alone.

### Identification of Small Molecules That Bind to *Sm*BRD3(1,2) and/or *Sm*BRD3(2)

**Figure 3:**
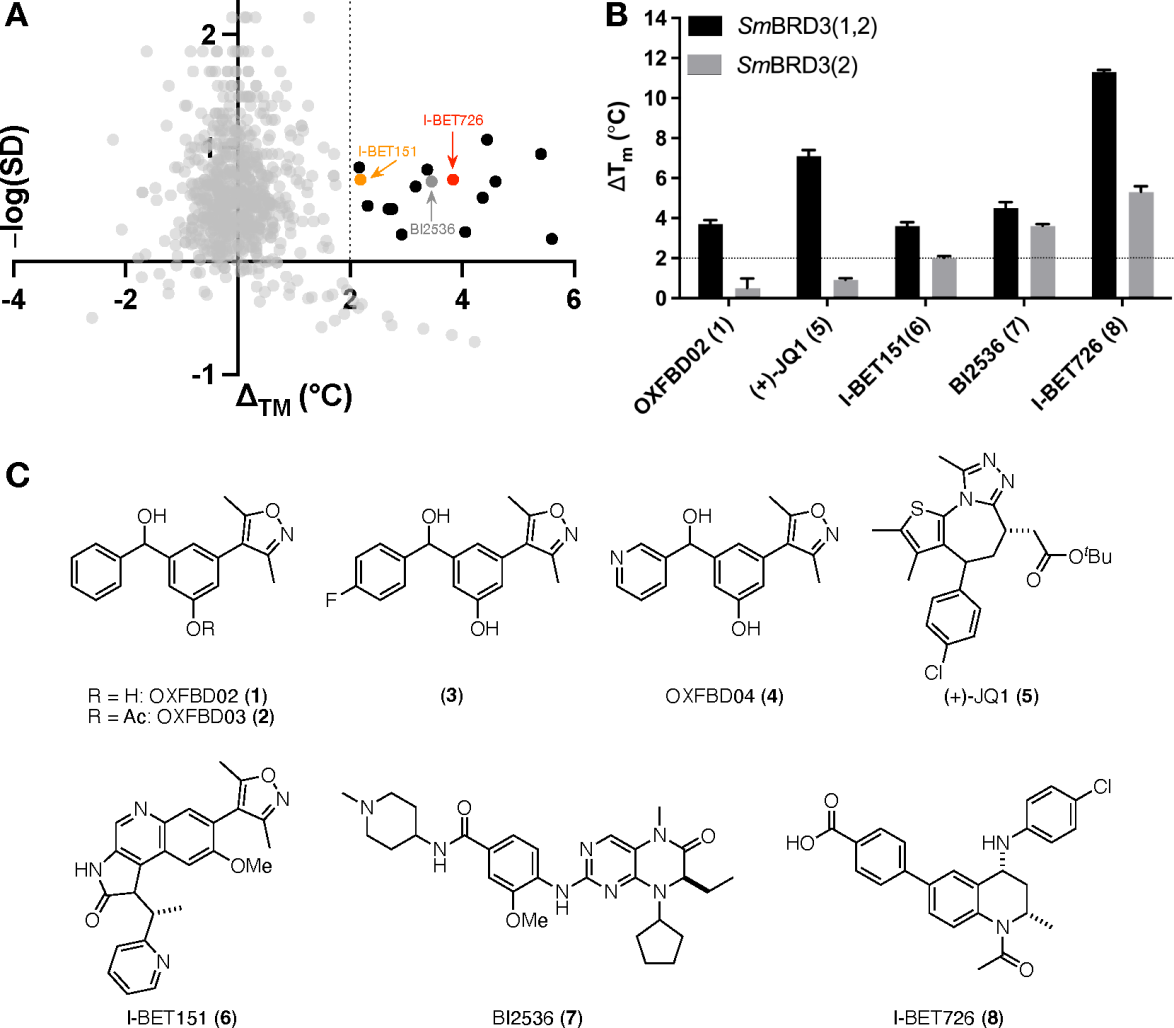
(A) Scatter plot showing the thermal shift (Δ*T*_m_) values of the 697 compounds screened against *Sm*BRD3(2), plotted against–log(SD). Δ*T*_m_ correlates with compound affinity for *Sm*BRD3(2) and higher values of–log(SD) correspond to greater confidence in the Δ*T*_m_ value quoted. The dark gray dot shows the Δ*T*_m_ value for BI2536, which was used as a positive control. The black dots show compounds that have a Δ*T*_m_ value >2 °C, which we typically consider to be a hit, and the red dot shows the Δ*T*_m_ value for I-BET726, and the orange dot shows the Δ*T*_m_ value for I-BET151. Reported Δ*T*_m_ values are the mean of two replicates. (B) Thermal shift (Δ*T*_m_) values of compounds **1, 5**–**8** against *Sm*BRD3(1,2) (black bars) and *Sm*BRD3(2) (gray bars). Compounds were tested at a concentration of 50 μM (*n* = 3), error bars show standard deviation (SD). (C)Chemical structures of compounds **1**–**8**.

Using the *Sm*BRD3(2) construct detailed above, we employed differential scanning fluorimetry (DSF)^27^ to screen 697 compounds, comprising 153 compounds from the PPI-net library, 160 compounds from our in-house library of human and *Trypanosoma cruzi* BRD ligands, and 384 compounds from the Maybridge Fragment Library against *Sm*BRD3(2).^28−37^ Analysis of Δ*T*_m_ values plotted against −log(SD) reveals a number of hits, including the known human BET bromodomain ligand, I-BET726 (Figure 3A).^38^ Selected compounds were then assessed against *Sm*BRD3(1,2), and interestingly the human BET bromodomain ligands, OXFBD02 (**1**)^29^ and (+)-JQ1 (**5**),^39^ were observed to stabilize *Sm*BRD3(1,2) but not *Sm*BRD3(2), implying that these compounds bind preferentially to *Sm*BRD3(1) (Figure 3B). Conversely, BI-2536 (**7**)^40^ and I-BET726 (**8**)^38^ stabilize both *Sm*BRD3(1,2) and *Sm*BRD3(2), suggesting that these compounds either bind to both *Sm*BRD3 BRDs, or to *Sm*BRD3(2) alone. Biophysical analysis using ITC (Table 1 and Table S2) confirmed the DFS results, and revealed that OXFBD02 (**1**) and (+)-JQ1 (**5**) have *K*_d_ values for *Sm*BRD3(1,2) of 683 ± 166 and 445 ± 106 nM, respectively, with no binding detected to *Sm*BRD3(2). The OXFBD02 (**1**) analogues OXFBD04 (**4**) and compound **3** showed the same behavior, but with a higher *K*_d_ value of 2950 ± 261 and 1750 ± 301 nM, respectively, for *Sm*BRD3(1,2). I-BET726 (**8**) has a *K*_d_ = 1520 ± 520 nM for *Sm*BRD3(1,2) and *K*_d_ = 1850 ± 361 nM, for *Sm*BRD3(2) consistent with the idea that this compound either binds to both *Sm*BRD3 BRDs or just to *Sm*BRD3(2). I-BET151 (**6**) and BI-2536 (**7**) show similar behavior, but as their *Sm*BRD3(2) affinities are lower, we progressed our studies with I-BET726 (**8**).

**Table 1:**
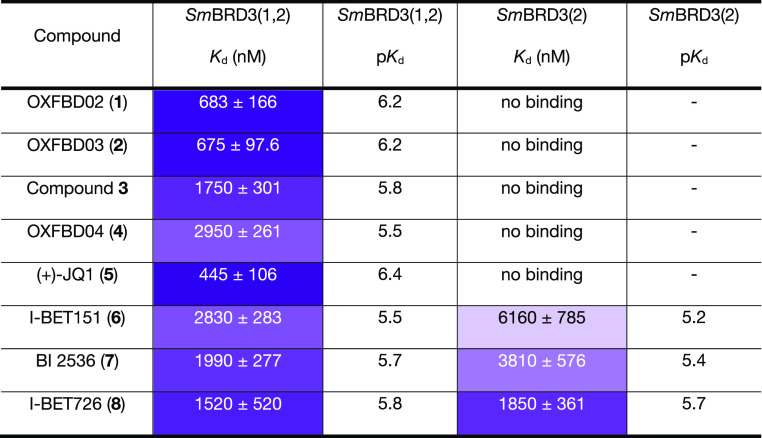
*Sm*BRD3(1,2) and *Sm*BRD3(2) ITC *K*_d_ and p*K*_d_ Values for Compounds **1**–**8**^a^

a A color scale is used with darker purple representing lower *K*_d_ values. Values are n = 1 ± error of the curve fit. ITC traces are shown in Figures S26–S33.

**Figure 4:**
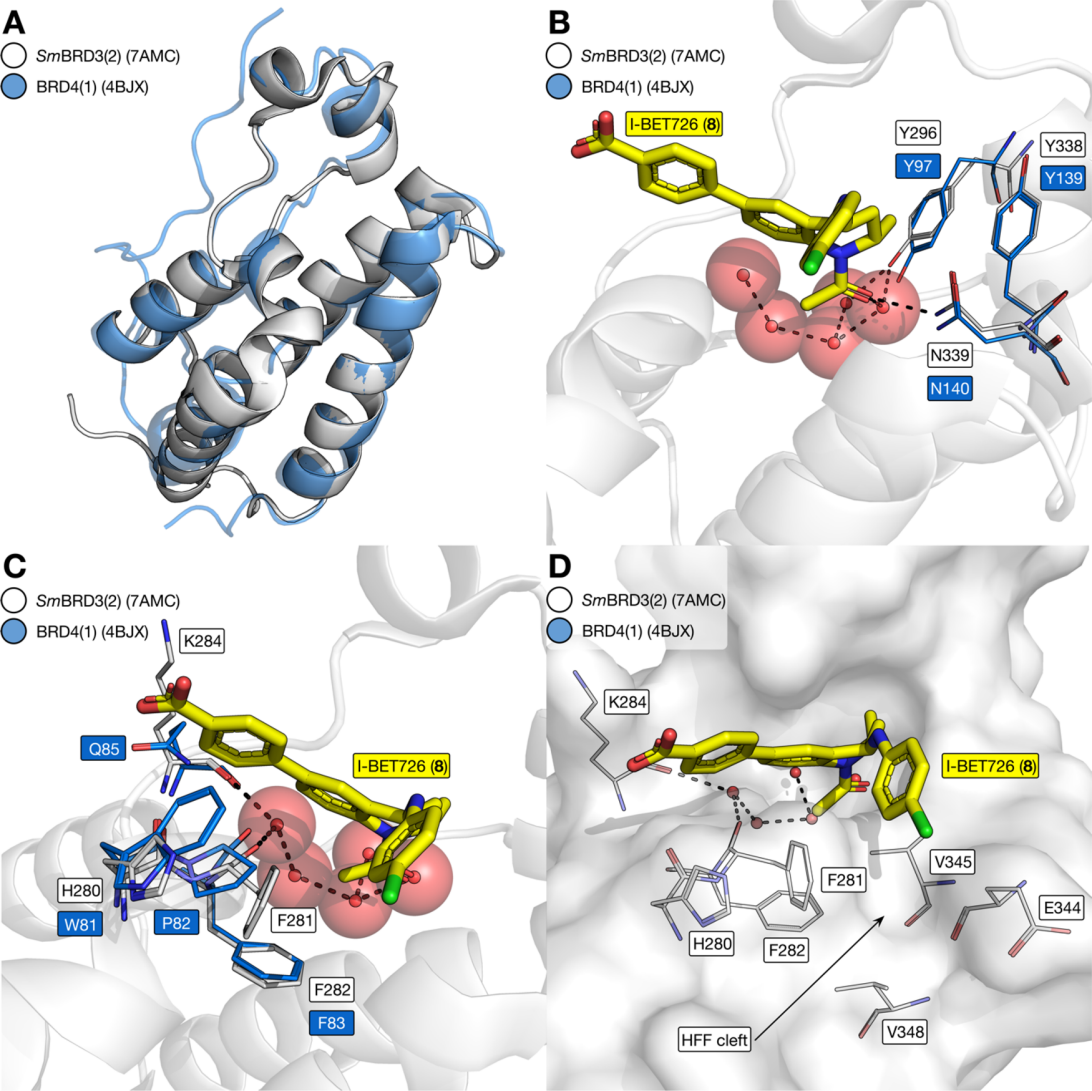
X-ray crystal structure of I-BET726 (**8**) bound to *Sm*BRD3(2) (PDB code: 7AMC). (A) Overall fold of *Sm*BRD3(2) (white cartoon) is similar to that of *HsBRD*4(1) (blue cartoon; PDB code: 4BJX). (B) X-ray crystal structure of I-BET726 (**8**) bound to *Sm*BRD3(2) (cartoon and carbon = white; PDB code: 7AMC) overlaid with the X-ray crystal structure of *HsBRD*4(1) (carbon = blue; PDB code: 4BJX). This comparison shows that the NYY motif, which is important for KAc- and KAc-mimic binding, is conserved between the proteins. (C) WPF shelf region of *HsBRD*4(1) carbon = blue; PDB code: 4BJX) is altered to an HFF motif in *Sm*BRD3(2) (cartoon and carbon = white; PDB code: 7AMC). (D) HFF residues result in a narrow channel, the HFF cleft, that is defined by F281, E344, V345, and V348. The chlorophenyl group of I-BET726 partly occupies the HFF cleft.

Given the similarity of *Sm*BRD3 and the human BET BRDs, we proposed that I-BET726 (**8**) would occupy the KAc binding pocket of *Sm*BRD3(2) when bound. To probe this assumption, we obtained an X-ray crystal structure of I-BET726 (**8**) bound to *Sm*BRD3(2) (Figure 4). This is the first X-ray crystal structure of a BRD from *S. mansoni* (PDB code: 7AMC) and it demonstrates that the overall fold of *Sm*BRD3(2) is very similar to that of *Hs*BRD4(1) (Figure 4A). The Tyr (Y97 and Y139) and Asn (N140) residues that are important for KAc in *Hs*BRD4(1)^24^ are conserved in *Sm*BRD3(2). N339 forms a hydrogen bond with the carbonyl oxygen atom of I-BET726 (**8**) and Y296 forms a water-mediated hydrogen bond with the same atom (Figure 4B). While *Hs*BRD4(1) and *Hs*BRD4(2) possess the WPF shelf, these residues are replaced by H280, F281, and F282 in *Sm*BRD3(2) (Figure 4C). This change results in the formation of a narrow channel, the HFF cleft, which is defined by F281, E344, V345, and V348, and in which the chlorophenyl group of I-BET726 resides (Figure 4D).

### X-ray Crystal Structure of I-BET726 Bound to *Sm*BRD3(2)

**Figure 5:**
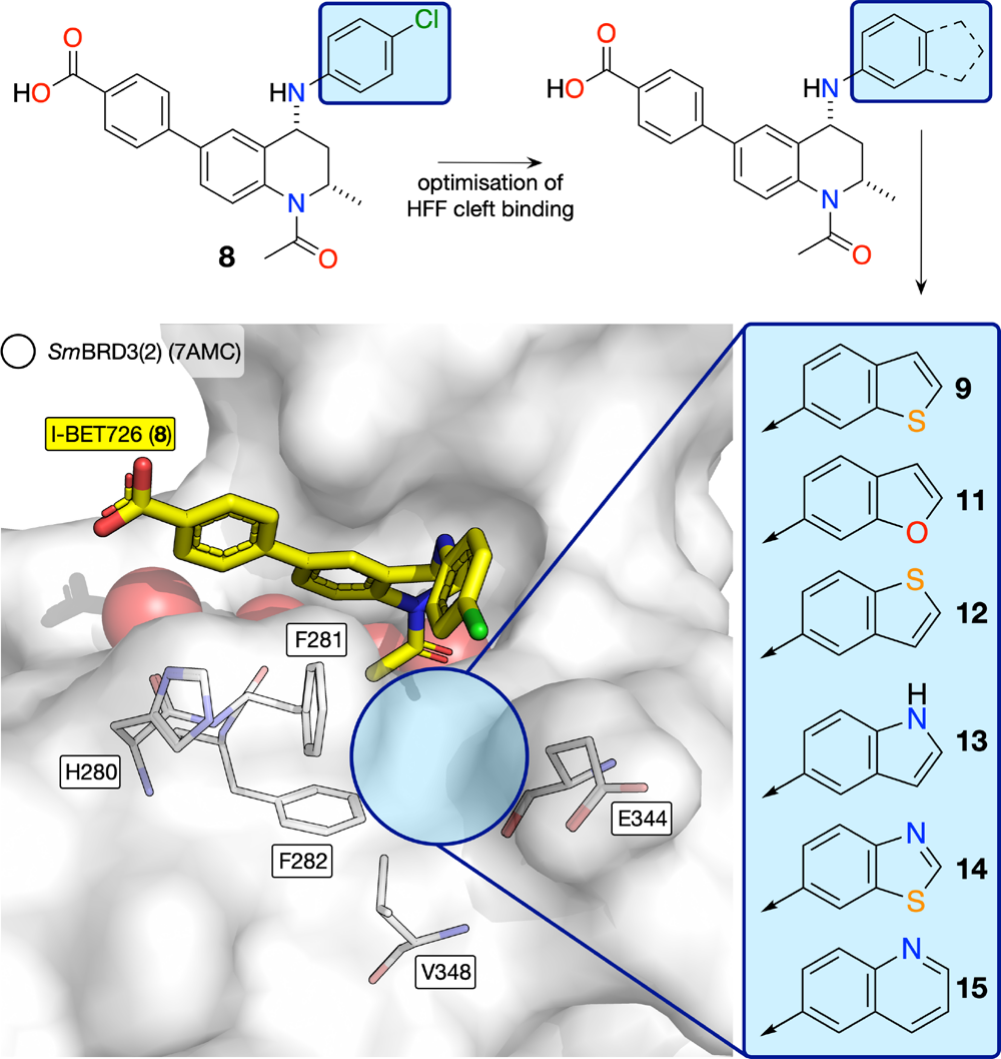
Design strategy that we employed to identify *Sm*BRD3(2) ligands that occupy the HFF cleft more effectively than the chlorophenyl moiety of I-BET726 (**8**). We proposed that a bicyclic group replacing the cholorophenyl group would result in more effective HFF occupancy and therefore higher *Sm*BRD3(2) affinity.

Despite the *Sm*BRD3(2) affinity of I-BET726 (**8**), the occupancy of the HFF cleft by the chlorophenyl group does not appear to be optimal. As the affinity of many BET bromodomain ligands results from the binding of lipophilic groups to the WPF shelf,^26,41^ we reasoned that developing I-BET726 derivatives that can better occupy the HFF cleft would result in compounds with higher affinity for *Sm*BRD3(2). Therefore, compounds **9**–**14** were designed, which incorporate a variety of [6.5]-fused ring systems (Figure 5). The quinoline derivative **15** was designed to probe the *Sm*BRD3 affinity of a [6.6]-fused bicycle. The synthesis of these compounds was based, in part, on the work of Gosmini et al.^38^ and Shadrick et al.^42^ with the appropriate bicyclic bromides employed in the Buchwald–Hartwig coupling. Full details are provided in the Supporting Information.

### Structure-Based Optimization of Small Molecule Ligands for *Sm*BRD3(2)

**Figure 6:**
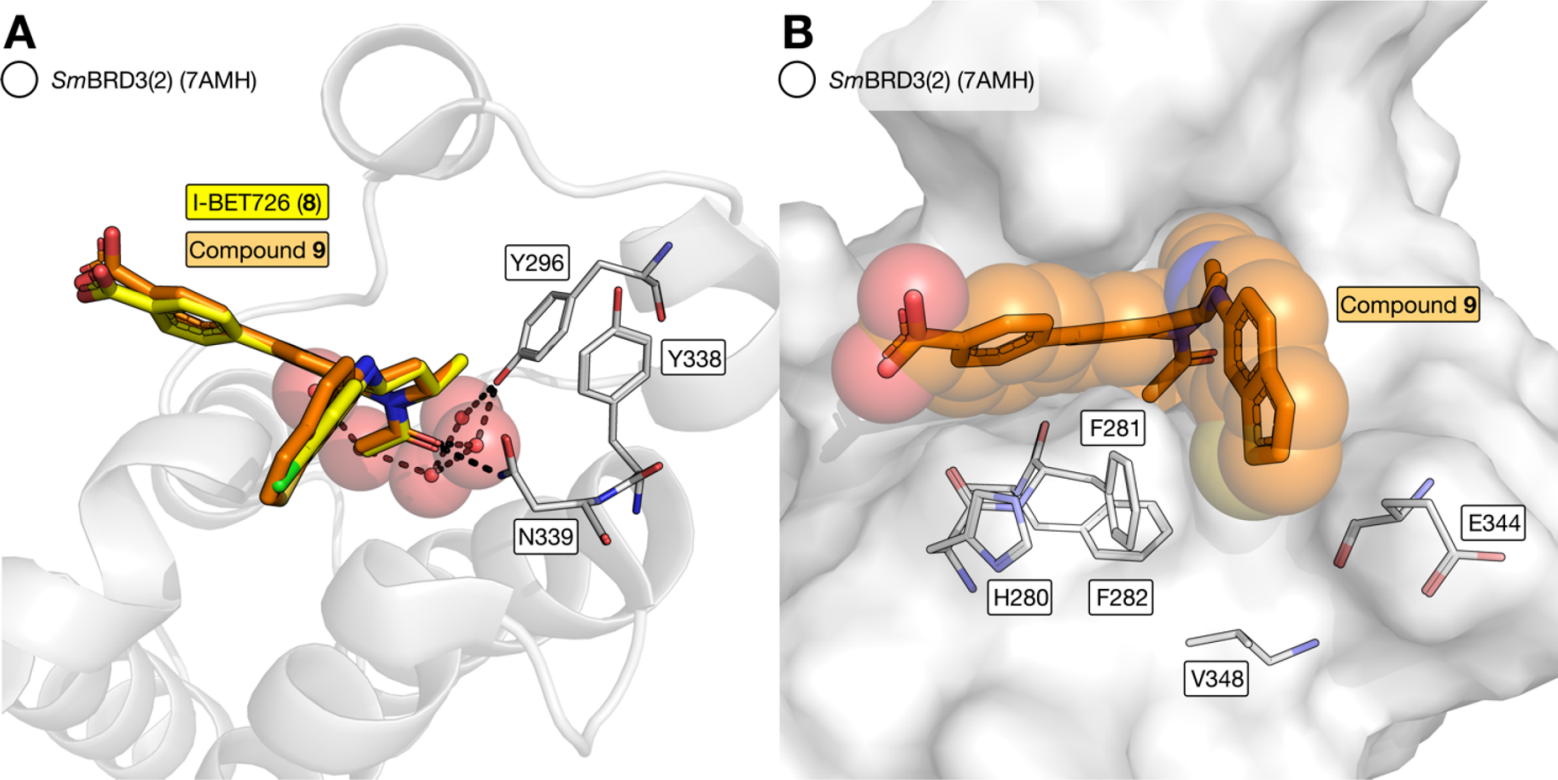
X-ray crystal structure of compound **9** bound to *Sm*BRD3(2) (cartoon and carbon = white, PDB code: 7AMH). (A) Overlay of 7AMH with 7AMC shows that I-BET726 (**8**) and **9** bind in the same orientation to *Sm*BRD3(2) and make the same interactions with Y296, Y338, and N339. (B) Benzothiophene group of **9** occupies the HFF cleft more fully than the chlorophenyl group of I-BET726 (**8**).

The benzothiophene-based compound **9** and its enantiomer, **10**, were synthesized first. Analysis using ITC showed that compound **9** has a *K*_d_ value of 701 ± 64.9 nM, while compound **10** showed no detectable binding (Table 2). This observation is in line with data obtained on human bromodomains, where the enantiomer of an I-BET726 derivative showed reduced binding to *HsBRD*4(1).^38^ To determine whether the increase in *Sm*BRD3(2) affinity displayed by compound **9** resulted from greater occupancy of the HFF cleft, we obtained an X-ray crystal structure of this compound bound to *Sm*BRD3(2) (Figure 6, PDB code: 7AMH).

**Table 2:**
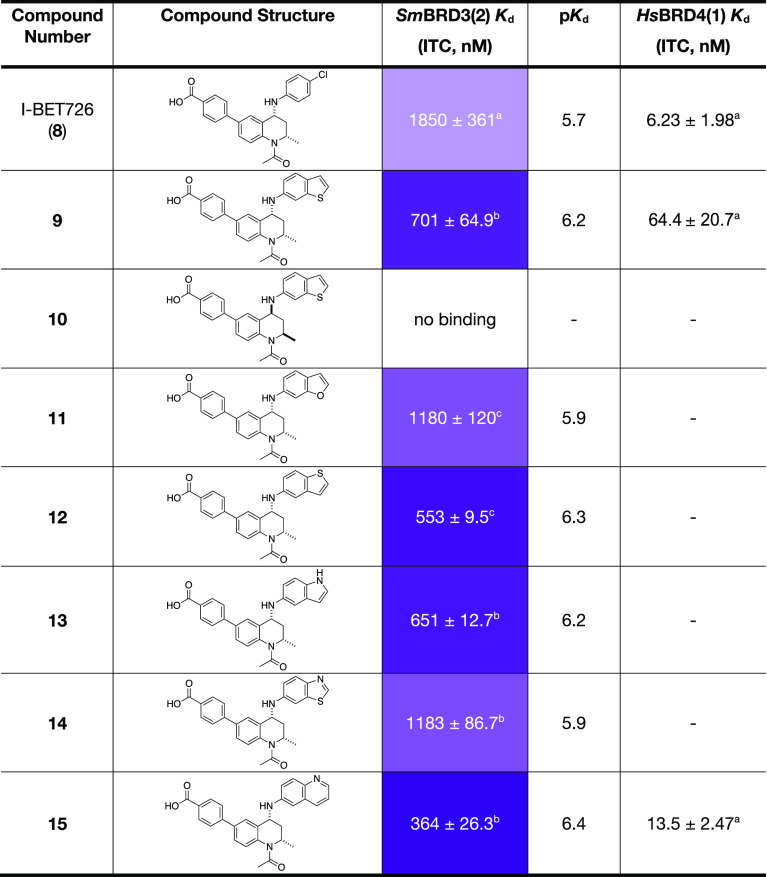
*Sm*BRD3(2) *K*_d_ Values of I-BET726 (**8**) and Compounds **9**–**15** Determined Using ITC^d^

a Single value ± error of the curve fit.

b Mean value of 3 repeats ± s.e.m.

c Mean value of 2 repeats ± s.e.m.

d A colour scale is used with darker purple representing lower *K*_d_ values. ITC traces are shown in Figures S33–S47.

This crystal structure shows that compound **9** binds to *Sm*BRD3(2) in the same orientation to I-BET726 (**8**) and forms the same interactions with N339 and Y296 (Figure 6A). The benzothiophene moiety is observed to occupy the HFF cleft, as proposed (Figure 6B), and this interaction could contribute to the increased *Sm*BRD3(2) affinity exhibited by compound **9**. Encouraged by these data, we synthesized compounds **11**–**15**, designed to probe the structure–activity relationship (SAR) of the HFF cleft, and evaluated their affinity for *Sm*BRD3(2) using ITC. All compounds show higher *Sm*BRD3(2) affinity compared to I-BET726 (**8**), with those compounds containing heteroatoms directed to the solvent (**12**, **13**, **15**) preferred over those with heteroatoms oriented toward the protein (**9**, **11**, **14**). Of the compounds evaluated, the quinoline derivative **15** possessed the highest *Sm*BRD3(2) affinity (ITC *K*_d_ = 364 ± 26.3 nM), which we attribute to effective occupancy of the HFF cleft by the [6.6]-fused ring system. We note that compounds **9** and **15** also show high affinity for *Hs*BRD4(1) (Table 2), which is not a problem for our studies here, but this activity would need to be absent from *Sm*BRD3(2) ligands intended as treatments for schistosomiasis. The ethyl esters of compounds **9**–**15** (compounds **16**–**22**) were also evaluated for their *Sm*BRD3(2) affinity using ITC. In all cases no binding could be detected. To determine whether this observation resulted from low affinity or poor solubility, we analyzed the solubility (phosphate buffered saline at pH 7.4) and chromLogD values of the benzothiophene- and benzothiazole-based acids **9** and **14**, and the benzothiophene- and quinoline-based esters **17** and **22** (Table S4). The acids **10** and **14** showed solubility of >259 μM and >260 μM, respectively, under these conditions; compound **10** has a chromLogD of 2.83 and **14** has a chromLogD of 1.78. The esters **17** and **22**, however, have lower solubility of only 1 and 13 μM, respectively. The chromLogD value for **17** is 7.61 and for **22** the value is 5.95, indicating that these compounds are more lipophilic than the acid derivatives, as expected. These data suggest that we are unable to measure the *Sm*BRD3(2) affinity of the ethyl esters due to poor solubility and we cannot be certain whether these compounds have any affinity for *Sm*BRD3(2).

Encouraged by the above data and the broad expression of *smbrd3* across the parasite’s lifecycle (Figure 1C), we evaluated the effects of these compounds on sexually immature larval schistosomula, sexually mature adult worms, and snail-infective miracidia, using a series of *ex vivo* assays. We were concerned that the carboxylic acid-containing I-BET726 analogues might not show high parasite permeability, and therefore we also evaluated their ethyl ester precursors, which we reasoned could act as prodrugs to release the *Sm*BRD3(2)-binding ligand *in situ*.

### Assessing the Effects of *Sm*BRD3(2) Ligands on Schistosomula

**Figure 7:**
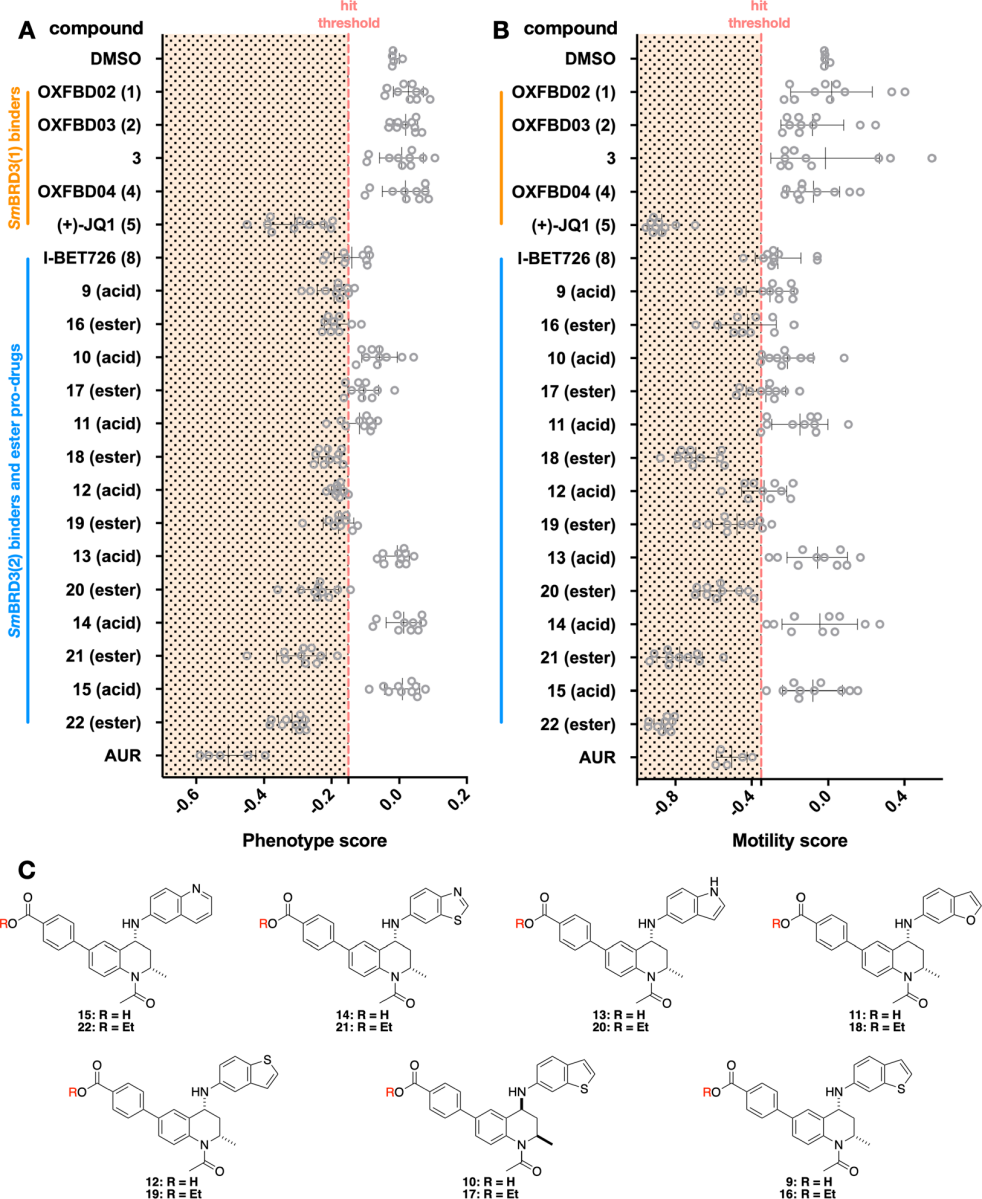
Effect of compounds **1**–**5** and **8**–**22** on the (A) phenotype and (B) motility of schistosomula (at 20 μM in 0.625% DMSO, following 72 h incubation). Negative (0.625% DMSO) and positive (10 μM auranofin in 0.625% DMSO) controls are included in each drug screen (4–5 in total, two technical replicates each). The compound score is shown as gray dots, and whiskers represent the average score and standard deviation across the screens. Hit threshold is delineated by the vertical dashed red lines in the graphs; −0.15 and −0.35 for phenotype and motility scores, respectively. Orange: *Sm*BRD3(1) selective compounds and Blue: *Sm*BRD3(2) selective compounds. (C) Structure of compounds **1**, **2**, and **9**–**22**.

A high-throughput imaging platform that quantifies both phenotype and motility *ex vivo*, was used to screen compounds against schistosomula; their effects were assessed as previously described.^20^ Briefly, the schistosomula stage of *S. mansoni* was obtained by mechanical transformation of cercariae, dispensed into 384-well tissue culture plates, and dosed with 20 μM (Figure 7) or 10 μM (Figure S48) of each compound. Following 72 h coincubation of the schistosomula and compound, the plate was subjected to high content imaging to quantify the effects of each compound. As expected, no carboxylic acid-containing I-BET726 analogues showed substantial activity on schistosomula phenotype and motility metrics, which we attributed to low permeability resulting from higher polarity (determined by chromLogD, see above). However, the majority of their ethyl ester derivatives were classified as hits. The quinoline derivative **22**, and the benzothiazole derivative **21**, showed the greatest effects on schistosomula phenotype and motility, while the benzofuran derivative **18** also had substantial effects on motility (Figure 7 and Figure S48). The carboxylic acid counterparts of these compounds all have affinity for *Sm*BRD3(2). However, compound **10**, which is the enantiomer of **9** showed no binding to *Sm*BRD3(1) or *Sm*BRD3(2), and the corresponding ethyl ester, **17**, did not have substantial effects on either phenotype or motility. This result suggests a link between the phenotypic effects and inhibition of *Sm*BRD3(2) function. In contrast, of the *Sm*BRD3(1) binding compounds evaluated (orange, Figure 7A), only (+)-JQ1 (**2**) showed activity *ex vivo*, which is in line with previous reports.^20^ The phenotype and movement EC_50_ values of the more active compounds, **18** and **20**-**22** are in the low micromolar range (Table 3), but there is only moderate correlation with the *Sm*BRD3(2) *K*_d_ values of the corresponding carboxylic acids. This reflects the range of characteristics that affect the activity of these compounds on the live schistosomula, including permeability and metabolic stability.

**Table 3:**
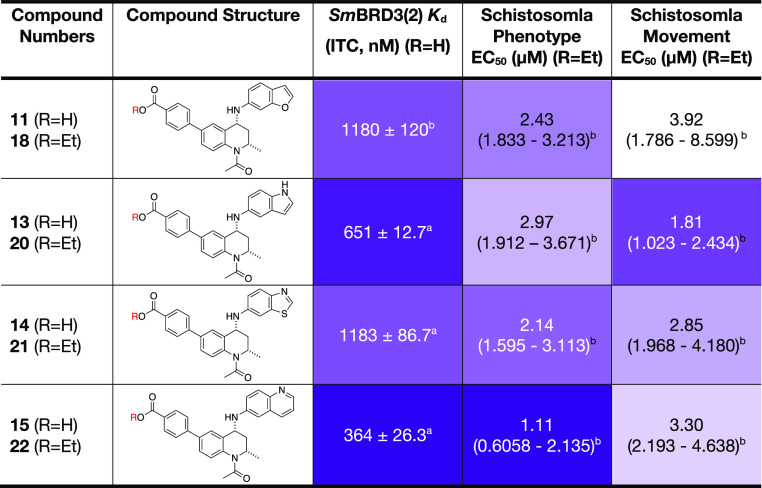
*Sm*BRD3(2) *K*_d_ Values (ITC) for the Carboxylic Acids **11** and **13**–**15** and Schistosomula Phenotype and Movement EC_50_ Values for the Corresponding Ethyl Esters **18** and **20**–**22**^d^

a Mean value of 3 repeats ± s.e.m.

b 95% Confidence Interval (CI).

c Mean value of 2 repeats ± s.e.m.

d A color scale is used with darker purple representing lower *K*_d_ values or lower EC_50_ values.

### Assessing the Effects of *Sm*BRD3(2) Ligands on Adult *S. mansoni* Worms

**Figure 8:**
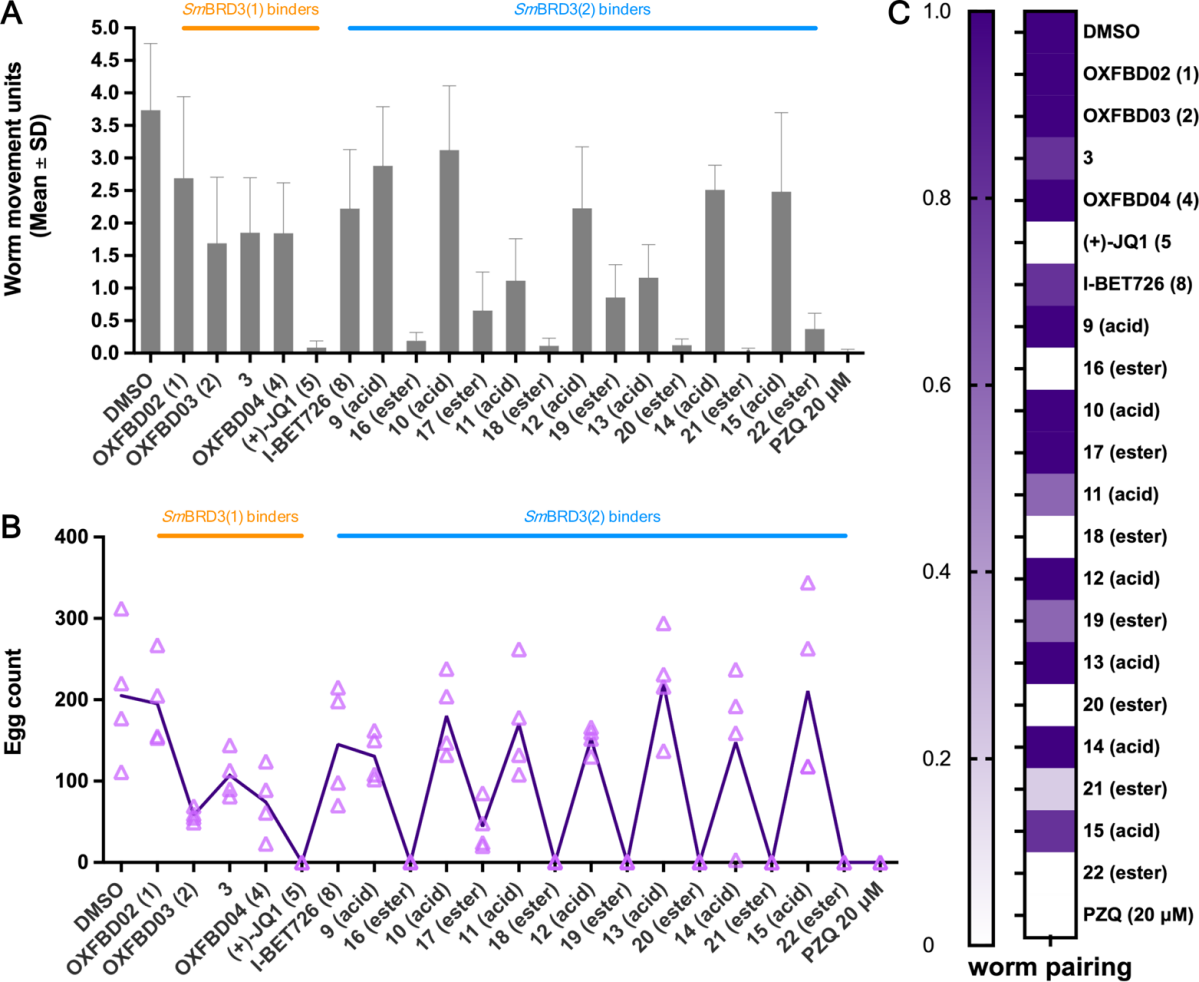
Effect of compounds **1**–**5** and **8**–**22** on adult *S. mansoni* motility, pairing, and egg production. (A) Worm movement recordings following 72 h compound-treatment (20 μM in 0.2% DMSO). Negative (0.2% DMSO) and positive (20 μM Praziquantel – PZQ in 0.2% DMSO) controls are included in each screen (*n* = 2 independent compound screens, 2 technical replicate each). The gray bars represent the mean worm movement (+ standard deviation). (B) At 72 h, *in vitro* laid eggs (IVLEs) were collected and enumerated. For each compound tested, individual egg counts are represented in a scatter plot; the purple line represent the mean trend across the treatments. (C) Effect of compound treatment on worm pairing, with 1 and 0 corresponding to 100% and 0% of parasite pairs following 72 h drug-treatment, respectively, (mean of *n* = 2 compound screens).

The *ex vivo* screens of these small molecules on adult schistosome pairs broadly mirrors the results of the phenotypic and motility assays performed on schistosomula, with the ethyl esters of the I-BET726 derivatives again showing the greatest effects on the adult worms. The benzothiophene (**16**), benzofuran (**18**), indole (**20**), benzothiazole (**21**), and quinoline (**22**) derivatives all showed substantial effects on worm movement (Figure 8A); however, none of the compounds were lethal to the parasite. Compounds that affect worm motility are also associated with reduced oviposition (Figure 8B) and decreased worm pairing (Figure 8C). It is notable that compound **10** does not bind to *Sm*BRD3(2), but its ethyl ester (**17**) showed some modest effects on worm movement and egg count (Figure 8A,B), but not on worm pairing (Figure 8C).

**Figure 9:**
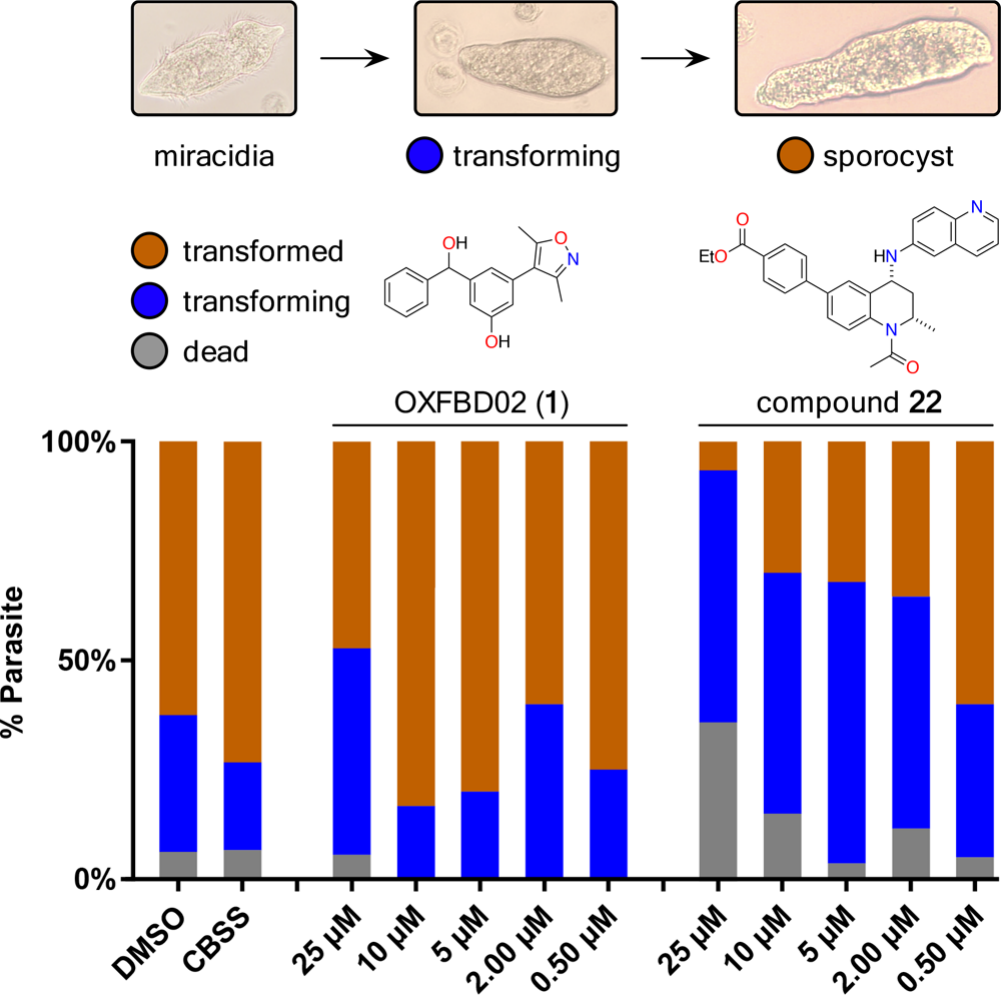
Differential activity of compound OXFBD02 (**1**) and compound **22** on *ex vivo* miracidia to sporocyst transformation. Miracidia were exposed to the selected compounds in a dose–response titration (CBSS containing 25, 10, 5, 2, or 0.5 μM in 1% DMSO). Dead parasites (in gray), transforming miracidia (in blue), and fully transformed sporocysts (in brown) were enumerated after 48 h (scored as percentage of parasite population, % Parasite). Each titration point was assessed in three independent experiments (two technical replicates per data point) and compared to parasites cultured in CBSS with 1% DMSO (controls) at a constant temperature of 26 °C, in the dark.

### Assessing the Effects of *Sm*BRD3(2) Ligands on the Transformation of Miracidia to Sporocysts

As *smbrd3* is most abundantly expressed in miracidia and sporocysts (Figure 1C), we were intrigued to investigate whether our *Sm*BRD3 BRD ligands affect *ex vivo* miracidia-to-sporocyst transformation. This process occurs naturally in the intermediate host snail and is critical for lifecycle progression. Two *Sm*BRD3(2) ligand pro-drugs compounds **22** and **16** showed concentration-dependent inhibition of the miracidia to sporocyst transformation (Figure 9 and Figure S49). Interestingly, compound **22** is the ethyl ester of compound **15**, which has the highest affinity for *Sm*BRD3(2) (Table 2). Most other *Sm*BRD3(2) ligands had no effect on the transformation. The negative control, **17**, and (+)-JQ1 were toxic at the higher concentrations (Figure S49). While OXFBD02 and OXFBD04 showed modest effects on the miracidia to sporocyst transformation at a concentration of 25 μM, the other *Sm*BRD3(1)-selective ligands generally had no effect in this assay (Figure S49).

### Determining the Permeability of Acids and Ethyl Esters in Adult Worms

As the low solubility of the ethyl ester derivatives prevented us from determining their affinity for *Sm*BRD3(2) using ITC, it is possible that these compounds are themselves inhibitors of *Sm*BRD3(2). Alternatively, the ethyl esters could function as pro-drugs that release the corresponding acid in the parasite. To investigate compound permeability, adult worms were treated with 20 μM of the acid **15** or the ethyl ester **22** for 24 h, and then the media exchanged three times. The worms were then lysed and the levels of the acid **15** or the ethyl ester **22** were determined using LCMS (Figure S50). High quantities of the ethyl ester **22** were found in the worms treated with this compound, while lower quantities of the acid **15** were found in the worms treated with **15** (Figure S51). Interestingly, a similar amount of the acid **15** was also observed in the worms treated with the ester **22**, indicating that it can be converted from the ester to the acid in the worm. However, some ester hydrolysis was also seen in media alone (Figure S52). These data confirm the high worm permeability of the ethyl ester **22** and demonstrate that it is possible for this compound to be converted to the corresponding acid **15**. Using this approach, it was also shown that OXFBD03 (**2**) is rapidly deacetylated to give OXFBD02 (**1**) in media in the presence or absence of worms. As both **1** and **2** have similar affinities for *Sm*BRD3(1,2), this does not affect the ability of the compound to interact with this protein once in the parasite.

## Discussion

The complete reliance on praziquantel to treat schistosomiasis represents a global vulnerability in the sustainable control of this neglected disease, especially in the case that praziquantel-resistant schistosomes develop. In the absence of a vaccine, and with very little new chemical matter being progressed into late-stage preclinical investigations, this situation presents a substantial challenge for the teams of medicinal chemists and parasitologists seeking to identify praziquantel replacements as part of collaborative drug discovery initiatives. Historically, the main approach to identifying antischistosomal compounds has relied on phenotypic screening, where the cellular target of the compound is (at least initially) unknown. More recently, efforts have been made to identify *S. mansoni* targets that can be modulated for therapeutic benefit or to repurpose molecules that target a specific class of human proteins.^43^ As a digenetic parasite, *S. mansoni* has adopted a lifecycle comprised of distinct morphological forms to maximize survival in a range of harsh environments, including the intermediate host snail, the definitive mammalian host, and fresh-water bodies. The phenotypic changes that accompany lifecycle progression occur without alteration of its genetic code, suggesting that the parasite’s epigenetic machinery plays a key role in these processes. We reasoned, therefore, that BRD-containing epigenetic regulators could be therapeutically interesting targets for the development of antischistosomal compounds. Having identified 29 BRDs in *S. mansoni*, we have begun a systematic investigation into their function. Our approach of focusing on *Sm*BRD3, which is the *S. mansoni* BRD that is most similar to the human BET BCPs, was validated by the identification of high affinity ligands for both *Sm*BRD3(1) and *Sm*BRD3(2).

Following identification of ligands for both *Sm*BRD3(1) and *Sm*BRD3(2), a structure-based optimization allowed us to develop the quinoline derivative **15**, which has a *K*_d_ value of 364 ± 26.3 nM for *Sm*BRD3(2). The ethyl ester derivatives of this compound, **22**, had strong effects on schistosomula phenotype and movement. It also reduced adult worm movement, pairing, and egg production (the lifecycle stage responsible for schistosomiasis pathology and transmission). Compound **22** additionally showed a substantial and concentration-dependent effect on the ability of miracidia to transform into sporocysts; this was likely due to high expression of *smbrd3* in these lifecycle states (Figure 1C). At concentrations as low as 2.00 μM, the majority of the miracidia were prevented from transforming into sporocysts. It is notable that most *Sm*BRD3(1) ligands did not show effects against schistosomula, miracidia, or adult worms. Given that both compound **22** and OXFBD03 (**2**) can be delivered to worms, these results suggest that *Sm*BRD3(1) and *Sm*BRD3(2) have different functions, and consequently their selective inhibition results in different phenotypes.

## Conclusions

In conclusion, our data strongly indicate that inhibition of the *Sm*BRD3 BRDs has significant effects on three *S. mansoni* life stages, and studies are ongoing to link the detrimental effects on *S. mansoni* lifecycle progression and survival to *Sm*BRD3 inhibition. The high affinity small molecule *Sm*BRD3 ligands we have identified provide a firm foundation for the development of further molecular tools that will enable us to investigate the link between *Sm*BRD3 BRD inhibition and the phenotypes observed, providing unprecedented insight into the role of BRDs and epigenetics in the *S. mansoni* lifecycle. The identification of a new *S. mansoni* target that is ligandable and important for parasite survival is a major advancement in the search for novel antischistosomal targets and drugs.

## Chemistry Experimental Section

Reagents and solvents used were of commercially available reagent grade quality from Sigma-Aldrich, Fluorochem, Alfa Aesar, Merck, Acros Organics, Apollo Scientific, Fisher Scientific, or Fluka and used without purification unless otherwise stated. All nonaqueous reactions requiring anhydrous conditions were carried out in a flame-dried flask under an inert atmosphere of argon or nitrogen unless otherwise stated. Anhydrous solvents were obtained from an MBRAUN MB5 Solvent Purification System and stored over 3 Å molecular sieves under an inert argon atmosphere. Concentration *in vacuo* refers to removal of solvent on a Buchi rotary evaporator under reduced pressure in a water bath at 40 °C. Celite refers to Celite 545 filter aid, treated with sodium carbonate, flux-calcined (Sigma-Aldrich). Brine refers to a saturated aqueous solution of sodium chloride. Petroleum ether refers to the fraction in the boiling point range 40–60 °C.

^1^H NMR spectra were measured on a Bruker AVIII HD 400 (400 MHz), AVII 500 (500 MHz), AVIIIHD 600 (600 MHz), or NEO 600 (600 mHz) spectrometer in the stated solvents as a reference for the internal deuterium lock. The chemical shift data for each signal are given as δ in units of parts per million (ppm) relative to tetramethylsilane (TMS) where δ(TMS) = 0.00. The spectra are calibrated using the solvent peak with the data provided by Fulmer et al.^44^ The multiplicity of each signal is indicated by s (singlet); d (doublet); t (triplet); q (quartet); m (multiplet); sp (septet); or combinations thereof. The number of protons (*n*) for a given resonance signal is indicated by nH. Where appropriate, coupling constants (*J*) are quoted in Hz, recorded to the nearest 0.1 Hz. Spectra were assigned using COSY, HSQC, and HMBC experiments as necessary. Relative stereochemistry was assigned via NOESY experiments.

^13^C NMR spectra were measured on a Bruker AVII 500 (126 MHz), AVIIIHD 600 (151 MHz), or NEO 600 (151 MHz) spectrometer in the stated solvents as a reference for the internal deuterium lock. The chemical shift data for each signal are given as δ in units of parts per million (ppm) relative to tetramethylsilane (TMS) where δ(TMS) = 0.00. The spectra are calibrated using the solvent peak with the data provided by Fulmer et al.^44^ The chemical shift is quoted to 1 decimal places, unless two different shifts are indistinguishable, when the shifts are quoted to 2 decimal places. Spectra were assigned using HSQC and HMBC as necessary.

^11^B NMR spectra were measured on a Bruker AVIII HD 400 (128 MHz) spectrometer in the stated solvents as a reference for the internal deuterium lock. The chemical shift data for each signal are given as δ in units of parts per million (ppm). The spectra are uncalibrated. Coupling constants (*J*) are quoted in Hz, recorded to the nearest 0.1 Hz.

Mass spectra were acquired on either an Agilent 6120 (low resolution), Waters LCT Premier XE benchtop, Waters LCT Premier benchtop orthogonal acceleration time-of-flight LC-MS system (low resolution), or Bruker microToF spectrometer (high resolution) using electrospray ionization (ESI) from solutions of methanol or acetonitrile. *m*/*z* values are reported in Daltons and followed by their percentage abundance in parentheses.

Melting points were determined using a Griffin capillary tube melting point apparatus or Leica Galen III hot stage microscope and are uncorrected. The solvent(s) from which the sample was crystallized is given in parentheses.

Specific optical rotations were measured using a Schmidt Haensch Unipol polarimeter, using a sodium lamp at 589 nm and a path length of 1.0 dm. The concentration (*c*) is expressed in g/100 mL (equivalent to g/0.1 dm^3^). Specific rotations are denoted [α]_D_^T^ and are given in implied units of 10^–1^ deg cm^2^g^–1^ (where *T* = ambient temperature in °C).

### Analytical High-Performance Liquid Chromatography (HPLC)

Method 1 was carried out on a PerkinElmer Flexar system with a Binary LC Pump and UV/vis LC Detector, with detection at 254 nm, using a Dionex Acclaim reverse phase 120 column (C18, 5 μm, 120 Å, 4.6 × 150 mm) which was employed using a flow rate of 1.5 mL min^–1^; [95:5 H_2_O: MeCN → 5:95 H_2_O: MeCN: H_2_O with 0.1% TFA modifier, 10 min; 5 min hold; 1.5 mL min^–1^]. Method 2 was carried out on an Agilent 1260 Infinity II system, with detection at 254 nm, using a Poroshell 120 EC-C18 column [4 μM, 4.6 × 100 mm]; [95:5 H_2_O: MeCN → 5:95 H_2_O: MeCN with 0.1% FA modifier, 10 min; 5 min hold; 1 mL min^–1^]. The purity of all biologically tested compounds was ≥95% as determined using the HPLC methods above.

Semipreparative high-performance liquid chromatography (HPLC) was carried out an a Agilent 1260 Infinity II with an Agilent 5 Prep C18 column [5 μM, 21.2 × 50 mm] [95:5 H_2_O:MeCN % FA modifier (1 min), 95:5 H_2_O:MeCN → 5:95 H_2_O:MeCN with 0.1% FA modifier (10 min), 5 min hold; 20 mL min^–1^].

Chiral high-performance liquid chromatography (HPLC) was carried out on a PerkinElmer Flexar system with a Binary LC Pump and UV/vis LC Detector or on a Thermofisher/Dionex Ultimate 3000 system comprising of a LPG-3400SD pump, WPS-3000SL autosampler, TCC-3000SD column compartment fitted with the appropriate Daicel Chiralpak column (dimensions: 0.46 cm o̷ × 25 cm) and corresponding guard column (0.4 cm o̷ × 1 cm), and a DAD3000 diode array detector, both at 1 mL min^–1^. The solvent system and column used for the compound are stated, where appropriate, and UV absorbance for both methods was measured at 254 nm.

A CAD solubility assay was carried out by Physchem Team, Discovery Analytical, NCE Molecular Discovery at GSK. 5 μL of 10 mM DMSO stock solution was diluted to 100 μL with pH 7.4 phosphate buffered saline, equilibrated for 1 h at room temperature, and filtered through Millipore Multiscreen_HTS_-PCF filter plates (MSSL BPC). The filtrate was quantified using a suitably calibrated charged aerosol detector.^45^

Lipophilicity: a chromLogD assay was carried out by the Physchem Team, Discovery Analytical, NCE Molecular Discovery at GSK. The chromatographic hydrophobicity index (CHI)^46,47^ values were measured using reversed phase HPLC column (50 × 2 mm 3 μM Gemini NX C18, Phenomenex, UK) with fast acetonitrile gradient at starting mobile phase of pHs 2, 7.4, or 10.5. CHI values were derived directly from the gradient retention times using calibration parameters for standard compounds. The CHI value approximates to the volume % organic concentration when the compound elutes. CHI was linearly transformed into a chromLogD^48^ value by least-squares fitting of experimental CHI values to calculated cLogP values for over 20000 research compounds.

#### Isopropyl Carbamate (**S1**)

Using a modified version of the procedure reported by Laurin et al.,^37^ trifluoroacetic acid (TFA) (17.0 mL, 222 mmol, 1.7 equiv) was added to a solution of isopropyl alcohol (10.0 mL, 131 mmol, 1.0 equiv) and NaOCN (12.6 g, 195 mmol, 1.5 equiv) in toluene (40 mL) cooled to 0 °C. The resulting suspension was warmed to rt and stirred for 4 h. After this time, H_2_O (100 mL) was added, and the mixture was extracted with EtOAc (2 × 150 mL). The combined organic components were washed with brine (150 mL), dried over MgSO_4_, filtered, and concentrated *in vacuo* to yield colorless crystals of isopropyl carbamate (**S1**) (10.7 g, 79%), which was used without further purification: *R*_*f*_ 0.31 (20% EtOAc/petroleum ether); mp 82–84 °C (from EtOAc) [lit.^49^ 88–90 °C, lit.^50^ 89–93 °C, lit.^37^ 90–92 °C]; ^1^H NMR (400 MHz, CDCl_3_) δ_H_ 4.89 (1H, sp, *J* 6.3), 4.58 (2H, br s), 1.24 (6H, d, *J* 6.3); LRMS *m*/*z* (ESI^+^) 126 ([M + Na]^+^, 20%). These data are in good agreement with the literature values.^37,49,50^

#### Isopropyl (*E*)-But-2-enoylcarbamate (**S2**)

Using a procedure reported by Shadrick et al.,^42^ crotonoyl chloride (4.8 mL, 50 mmol, 1.1 equiv) was added to a solution of compound **S1** (4.6 g, 45 mmol, 1.0 equiv) in anhydrous THF (43 mL) at −78 °C followed by LiHMDS (1 M in THF, 90 mL, 90 mmol, 2.0 equiv). The reaction mixture was warmed to rt and was stirred for 16 h. After this time, the reaction was quenched by the addition of chilled saturated NH_4_Cl_(aq)_ (50 mL). The resulting solution was extracted with EtOAc (3 × 50 mL). The combined organic components were washed with brine (50 mL), dried over MgSO_4_, filtered, and concentrated *in vacuo*. The crude material was purified using flash column chromatography (0–20% EtOAc/petroleum ether) to give compound **S2** as a colorless solid (4.7 g, 61%): *R*_*f*_ 0.39 (17% EtOAc/petroleum ether); mp 75–78 °C (from EtOAc) [lit.^42^ 91 °C, lit.^37^ 72–74 °C]; *v̅*_max_ (neat)/cm^–1^ 3285 (N–H, w), 2980 (C–H, s), 1765 (C=O, s), 1682 (C=O, w), 1647 (C=C, s); ^1^H NMR (400 MHz, CDCl_3_) δ_H_ 7.23 (1H, s), 7.13 (1H, dq, *J* 15.3, 6.9); 6.87 (1H, dq, *J* 15.3, 1.7), 4.99 (1H, sp, *J* 6.3), 1.94 (3H, dd, *J* 6.9, 1.7), 1.29 (6H, d, *J* 6.3); ^13^C NMR (151 MHz, CDCl_3_) δ_C_ 166.1, 151.5, 146.4, 123.1, 70.4, 21.9, 18.5; LRMS *m*/*z* (ESI^+^) 194 ([M + Na]^+^, 100%); HRMS *m*/*z* (ESI^+^) [found: 194.0786, C_8_H_13_O_3_NNa, requires [M + Na]^+^ 194.0788]. These data are in good agreement with the literature values.^37,38,42^

#### Isopropyl (*S*)-(3-(4′-Bromoanilinyl)butanoyl)carbamate (S3a)

(*R*)-BINAP(OTf)_2_(H_2_O)_2_Pd (728 mg, 0.685 mmol, 0.06 equiv) was added to a solution of compound **S2** (2.00 g, 11.7 mmol, 1.0 equiv) in anhydrous degassed toluene (36 mL), and the resulting suspension was stirred at rt for 20 min. 4-Bromoaniline (3.54 g, 20.6 mmol, 1.8 equiv) was then added and the reaction mixture was stirred for a further 21 h. After this time, the reaction mixture was concentrated *in vacuo* and purified using flash column chromatography (0–20% EtOAc/petroleum ether) to give **S3a** as a colorless solid (3.82 g, 95%): *R*_*f*_ 0.16 (20% EtOAc/petroleum ether); [α]_D_^25^ = −6.7 (*c* 1.0, MeOH) [lit.^37^ −16.1 (*c* 1.0, CHCl_3_)]; mp 146–149 °C (from EtOAc) [lit.^42^ 131 °C, lit.^37^ 128–130 °C]; *v̅*_max_ (neat)/cm^–1^ 3266 (N–H, w), 1748 (C=O, s), 1178 (C–O, m); ^1^H NMR (400 MHz, CDCl_3_) δ_H_ 7.36 (1H, br s), 7.26–7.21 (2H, m), 6.52–6.48 (2H, m), 5.02–4.92 (1H, m), 4.04–3.94 (1H, m), 3.94–3.84 (1H, m), 3.09 (1H, dd, *J* 16.0, 5.9), 2.89 (1H, dd, *J* 16.0, 5.9), 1.30–1.26 (9H, m); ^13^C NMR (151 MHz, CDCl_3_) δ_C_ 172.8, 151.4, 145.9, 132.2, 115.5, 109.5, 70.7, 46.2, 42.0, 21.9, 20.8; LRMS *m*/*z* (ESI^–^) 341 ([M^79^Br–H^+^]^−^, 100%), 343 ([M^81^Br–H^+^]^−^, 81%); HRMS *m*/*z* (ESI^+^) [Found: 343.0647, C_14_H_20_O_3_N_2_^79^Br, requires [M + H]^+^ 343.0652]; Chiral HPLC, Chiral AD-H column (80:20 heptane/ethanol, 0.1% DEA, 1.0 mL min^–1^), retention time = 11.0 min (**S3a**, 96.7% UV), retention time = 16.3 min [opposite enantiomer (**S3b**), 3.3% UV]; 93% e.e. These data are in good agreement with the literature values.^37,38,42^

#### Isopropyl ((2*S*,4*R*)-6-Bromo-2-methyl-1,2,3,4-tetrahydroquinolin-4-yl)carbamate (**S4a**)

Sodium borohydride (229 mg, 6.05 mmol, 0.75 equiv) was added to a solution of compound **S3a** (2.77 g, 8.07 mmol, 1.0 equiv) in EtOH (143 mL), cooled to a temperature below −10 °C followed by addition of MgCl_2_·6H_2_O (1.81 g, 8.89 mmol, 1.1 equiv) in water (14.3 mL). The reaction mixture was stirred at a temperature below 0 °C for 2 h and then warmed to rt and stirred for 1 h. The resulting suspension was poured into a mixture of citric acid (60 mL, 0.5 M in water), HCl_(aq)_ (205 mL, 1 M), and CH_2_Cl_2_ (200 mL) and left to stir for 1 h. After this time, the layers were separated, the aqueous layer was extracted with CH_2_Cl_2_ (3 × 150 mL), and the combined organic components were dried over MgSO_4_, filtered, and concentrated *in vacuo* to give compound **S4a** as a colorless solid (2.60 g, 98%): *R*_*f*_ 0.68 (30% EtOAc/toluene); [α]_D_^25^ = +12.9 (*c* 1.0, MeOH) [lit.^37^ – 14.9 (*c* 1.0, CHCl_3_)], mp 130–136 °C (from toluene) [lit.^42^ 167 °C, lit.^37^ 148–150 °C]; *v̅*_max_ (neat)/cm^–1^ 3309 (N–H, m), 2980 (C–H, s), 1684 (C = O, s); ^1^H NMR (400 MHz, D_6_-DMSO) δ_H_ 7.38 (1H, d, *J* 9.1), 7.02 (1H, dd, *J* 8.6, 2.4), 6.95–6.92 (1H, m), 6.41 (1H, d, *J* 8.6), 5.89 (1H, br s), 4.87–4.79 (1H, m), 4.77–4.67 (1H, m), 3.48–3.39 (1H, m), 1.95–1.85 (1H, m), 1.49–1.40 (1H, m), 1.23 (3H, d, *J* 6.2), 1.20 (3H, d, *J* 6.2), 1.11 (3H, d, *J* 6.2); ^13^C NMR (151 MHz, D_6_-DMSO) δ_C_ 156.4, 144.2, 129.9, 128.6, 124.5, 115.6, 106.7, 66.1, 46.2, 40.1, 36.5, 22.0, 21.5; LRMS *m*/*z* (ESI^+^) 327 ([M+H^+^]^+^, 73%)**;** HRMS *m*/*z* (ESI^+^) [Found: 327.0703, C_14_H_20_O_2_N_2_^79^Br, requires [M + H]^+^ 327.0703]. Chiral AD-H column (90:10 heptane:ethanol, 0.1% DEA, 1.0 mL min^–1^), retention time = 8.1 min (**S4a**, 94.3% UV), retention time = 11.5 min (opposite enantiomer (**S4b**), 5.7% UV); 89% e.e. These data are in good agreement with the literature values.^37,38,42^

#### Isopropyl ((2*S*,4*R*)-1-Acetyl-6-bromo-2-methyl-1,2,3,4-tetrahydroquinolin-4-yl)carbamate (**S5a**)

Compound **S4a** (2.18 g, 6.36 mmol, 1.0 equiv) was dissolved in CH_2_Cl_2_ (60 mL) under N_2_ at room temperature. Pyridine (1.66 mL, 20.5 mmol, 3.2 equiv) was added followed by dropwise addition of acetyl chloride (0.75 mL, 10.5 mmol, 1.7 equiv). The reaction mixture was stirred for 40 min. The reaction mixture was partitioned between EtOAc (250 mL) and saturated NaHCO_3(aq)_ (250 mL). The aqueous layer was extracted with EtOAc (3 × 200 mL) and the combined organic components washed with water and brine, dried over Na_2_SO_4_, filtered, and then concentrated *in vacuo* to give compound **S5a** as a brown solid, which was judged to be pure enough to use in the next step without further purification (2.10 g, 90%). *R*_*f*_ 0.27 (50% EtOAc/petroleum ether); [α]_D_^25^ = +209.9 (*c* 1.0, MeOH) [lit.^37^ + 298.4 (*c* 1.0, CHCl_3_)]; mp 105–106 °C (from EtOAc) [lit.^42^ 163 °C, lit.^37^ 141–143 °C]; *v̅*_max_ (neat)/cm^–1^ 3334 (N–H, w), 1688 (C=O, s), 1660 (C=O, s); ^1^H NMR (400 MHz, D_6_-DMSO) δ_H_ 7.63 (1H, d, *J* 8.6), 7.47 (1H, dd, *J* 8.4, 2.3), 7.30 (1H, d, *J* 8.4), 7.21 (1H, d, *J* 2.3), 4.87–4.79 (1H, m), 4.67–4.56 (1H, m), 4.40–4.31 (1H, m), 2.44 (1H, ddd, *J* 12.7, 8.6, 4.3), 2.05 (3H, s), 1.25 (3H, d, *J* 6.3), 1.22 (3H, d, *J* 6.3), 1.20–1.14 (1H, m), 1.01 (3H, d, *J* 6.3); ^13^C NMR (151 MHz, D_6_-DMSO) δ_C_ 168.4, 155.7, 139.2, 135.5, 129.6, 128.2, 125.6, 117.9, 67.3, 47.0, 46.7, 39.8 (hidden by D_6_-DMSO multiplet, assigned using HSQC), 22.6, 22.0, 21.3; LRMS *m*/*z* (ESI)^+^ 391 ([M^79^Br+Na]^+^, 100%); HRMS *m*/*z* (ESI^+^) [Found: 369.0803, C_16_H_22_O_3_N_2_^79^Br, requires [M + H]^+^ 369.0808]. These data are in good agreement with the literature values.^37,38,42^

#### Ethyl 4-((2*S*,4*R*)-1-Acetyl-4-((isopropoxycarbonyl)amino)-2-methyl-1,2,3,4-tetrahydroquinolin-6-yl)benzoate (**S6a**)

A solution of compound **S5a** (1.10 g, 2.98 mmol, 1.0 equiv), {4-[(ethyloxy)carbonyl]-phenyl}boronic acid (0.609 g, 3.14 mmol, 1.05 equiv), and Pd(Ph_3_)_4_ (57 mg, 0.0493 mmol, 0.017 equiv) in DME (11.7 mL) was treated with an aqueous solution of Na_2_CO_3_ (2 M, 5.87 mL, 11.7 mmol, 3.9 equiv). The mixture was degassed and heated at 105 °C under argon for 2.5 h. After this time the mixture was cooled to rt and partitioned between EtOAc and water. The layers were separated, and the aqueous layer extracted with EtOAc (3 × 100 mL). The combined organic components were washed with water and brine, dried over Na_2_SO_4_, filtered, and concentrated *in vacuo* to give a gray-brown solid. This solid was redissolved in EtOAc and filtered through a silica plug eluting with a 1:1 EtOAc/petroleum ether mixture. The eluents containing the product were concentrated *in vacuo* to give **S6a** as a colorless solid (1.19 g, 91%). *R*_*f*_ 0.31 (25% acetone/petroleum ether); [α]_D_^25^ = +272.6 (*c* 1.0, MeOH); mp 73–75 °C (from acetone); *v̅*_max_ (neat)/cm^–1^ 3324 (N–H, w), 1718 (C=O, s), 1695 (C=O, s), 1659 (C=O, s), 1609 (N–H, m); ^1^H NMR (400 MHz, D_6_-DMSO) δ_H_ 8.08–8.04 (2H, m), 7.83–7.78 (2H, m), 7.70 (1H, d, *J* 8.7), 7.65 (1H, dd, *J* 8.3, 2.1), 7.48–7.44 (2H, m), 4.89–4.81 (1H, m), 4.70–4.61 (1H, m), 4.48–4.40 (1H, m), 4.34 (2H, q, *J* 7.1), 2.48–2.44 (1H, m) (partially hidden by DMSO peak), 2.10 (3H, s), 1.34 (3H, t, *J* 7.1), 1.28–1.21 (6H, m), 1.21–1.16 (1H, m), 1.06 (3H, d, *J* 6.3); ^13^C NMR (151 MHz, D_6_-DMSO) δ_C_ 168.5, 165.5, 155.9, 144.3, 137.1, 136.4, 135.7, 129.9, 128.7, 126.7, 126.6, 125.5, 121.3, 67.2, 60.7, 47.1, 46.9, 40.1, 22.7, 22.04, 22.01, 21.4, 14.2; LRMS *m*/*z* (ESI^+^) 461 ([M+Na^23^]^+^, 100%); HRMS *m*/*z* (ESI^+^) [found: 439.2220, C_25_H_31_O_5_N_2_, requires [M + H]^+^ 439.2227]. The LRMS and ^1^H NMR data are in good agreement with the literature.^38^

#### Ethyl 4-((2*S*,4*R*)-1-Acetyl-4-amino-2-methyl-1,2,3,4-tetrahydroquinolin-6-yl)benzoate (**S7a**)

Compound **S6a** (500 mg, 1.14 mmol, 1.0 equiv) was added to a suspension of AlCl_3_ (760 mg, 5.70 mmol, 5.0 equiv) in anhydrous CH_2_Cl_2_ (9 mL) and the resulting solution stirred for 20 min at 0 °C. A solution of anhydrous NEt_3_ (1.9 mL, 13.7 mmol, 12 equiv) and anhydrous MeOH (2 mL) was added dropwise. The mixture was stirred for 20 min at 0 °C, then diluted with EtOAc (20 mL) and saturated Rochelle salt solution (40 mL), and stirred for 30 min. After this time, the solution was filtered through Celite, eluting with EtOAc, saturated aqueous NaHCO_3_, acetone, and MeOH. The organic components were removed from the filtrate *in vacuo*. The resulting solution was then diluted with EtOAc (50 mL) and the organic and aqueous components were partitioned. The aqueous components were extracted with EtOAc (3 × 50 mL). The organic components were the dried with Na_2_SO_4_, and concentrated *in vacuo*. The crude material was purified using silica gel flash column chromatography (0–10% MeOH/CH_2_Cl_2_) to give **S7a** as a colorless solid (339 mg, 84%). *R*_*f*_ 0.69 (10% MeOH/CH_2_Cl_2_); [α]_*D*_^25^ = +315.0 (*c* 1.0, CHCl_3_); mp 38–45 °C (from EtOAc); *v̅*_max_ (thin film)/cm^–1^ 2978 (C–H, w), 1711 (C = O, s), 1645 (N–H, s); ^1^H NMR (400 MHz, CDCl_3_) δ_H_ 8.14–8.09 (2H, m), 7.77–7.75 (1H, m), 7.72–7.67 (2H, m), 7.53 (1H, dd, *J* 8.2, 1.8), 7.20 (1H, d, *J* 8.2), 4.91–4.78 (1H, m), 4.41 (2H, q, *J* 7.1), 3.80 (1H, dd, *J* 12.1, 4.4), 2.56 (1H, ddd, *J* 12.7, 8.6, 4.4), 2.16 (3H, s), 1.65 (2H, br s), 1.42 (3H, t, *J* 7.1), 1.20–1.11 (1H, m), 1.16 (3H, d, *J* 6.3); ^13^C NMR (151 MHz, CDCl_3_) δ_C_ 169.6, 166.6, 145.0, 140.7, 137.9, 136.5, 130.3, 129.5, 127.0, 126.3, 125.7, 121.6, 61.2, 47.8, 47.7, 44.7, 23.1, 21.6, 14.5; LRMS *m*/*z* (ESI^+^) 337 ([M–NH_2_+H]^+^, 25%); HRMS *m*/*z* (ESI^+^) [found: 353.1861, C_21_H_25_O_3_N_2_, requires [M + H]^+^ 353.1860]. The LRMS data are in good agreement with the literature.^38^

#### Isopropyl (*R*)-(3-(4′-Bromoanilinyl)butanoyl)carbamate (**S3b**)

The preparation of **S3b** was based upon a previously reported procedure.^37,38,42^ Compound **S2** (161 mg, 0.940 mmol, 1.0 equiv) was dissolved in anhydrous, degassed toluene (3 mL). (*S*)-BINAP(OTf)_2_(H_2_O)_2_Pd (50 mg, 0.0470 mmol, 0.050 equiv) was added, and the resulting mixture was stirred for 20 min. 4-Bromoaniline (240 mg, 1.40 mmol, 1.5 equiv) was added then and the resulting solution was stirred for 6 h 25 min. After this time the solution was concentrated *in vacuo* and purified using silica gel flash column chromatography (0–50% EtOAc/petroleum ether) to give **S3b** as a colorless solid (291 mg, 90%): *R*_*f*_ 0.13 (20% EtOAc/petroleum ether); [α]_D_^25^ = +7.45 (*c* 1.0, MeOH); mp 72–88 °C (from EtOAc) [lit.^42^ 131 °C, lit.^37^ 128–130 °C, (opposite enantiomer)]; ^1^H NMR (400 MHz, D_6_-DMSO) δ_H_ 10.47 (1H, s), 7.21–7.16 (2H, m), 6.54–6.48 (2H, m), 5.66 (1H, d, *J* 8.7), 4.84 (1H, sp, *J* 6.3), 3.85–3.71 (1H, m), 2.68 (1H, dd, *J* 15.7, 5.6), 2.45 (1H, dd, *J* 15.7, 7.5), 1.22 (6H, d, *J* 6.3), 1.12 (3H, d, *J* 6.4); LRMS *m*/*z* (ESI^+^) 365 ([M + Na]^+^, 64%); Chiral HPLC, Chiral AD-H column (80:20 heptane:ethanol, 0.1% DEA, 1.0 mL min^–1^), retention time = 11.0 min (opposite enantiomer (**S3a**), 7.7% UV), retention time = 16.3 min (**S3b**, 92.3% UV); 85% e.e. These data are in good agreement with the spectroscopic data for **S3a**.^37,38,42^

#### Isopropyl ((2*R*,4*S*)-6-Bromo-2-methyl-1,2,3,4-tetrahydroquinolin-4-yl)carbamate (**S4b**)

The preparation of **S4b** was based upon a previously reported procedure.^37,38,42^ Compound **S3b** (252 mg, 0.734 mmol, 1.00 equiv) was dissolved in EtOH (13 mL), and the resulting solution was cooled to below −10 °C. Sodium borohydride (21 mg, 0.555 mmol, 0.76 equiv) was added to this solution followed by a solution of MgCl_2_·6H_2_O (164 mg, 0.807 mmol, 1.1 equiv) in water (1.3 mL). The resulting suspension was poured into a mixture of citric acid (5.3 mL, 0.5 M), HCl_(aq)_ (1 M, 18 mL), and CH_2_Cl_2_ (18 mL). The layers were separated and the aqueous components were extracted with CH_2_Cl_2_ (3 × 30 mL), dried over Na_2_SO_4_, filtered, and concentrated *in vacuo* to give **S4b** as a colorless solid (224 mg, 93%): *R*_*f*_ 0.68 (30% EtOAc/petroleum ether); mp 140–143 °C (from CH_2_Cl_2_) [lit.^42^ 167 °C lit.^37^ 148–150 °C, (opposite enantiomer)]; [α]_D_^25^ = −7.8 (*c* 1.0, MeOH); ^1^H NMR (400 MHz, D_6_-DMSO) δ_H_ 7.38 (1H, d, *J* 9.1), 7.02 (1H, dd, *J* 8.6, 2.4), 6.96–6.93 (1H, m), 6.42 (1H, d, *J* 8.6), 4.87–4.79 (1H, m), 4.77–4.66 (1H, m), 3.44–3.39 (1H, m), 1.91 (1H, ddd, *J* 12.4, 5.8, 2.6), 1.49–1.37 (1H, m), 1.23 (3H, d, *J* 6.2), 1.20 (3H, d, *J* 6.2), 1.12 (3H, d, *J* 6.3); LRMS *m*/*z* (ESI^+^) 349 ([M + Na]^+^, 8%); Chiral HPLC, Chiral AD-H column (85:15 heptane/ethanol, 0.1% DEA, 1.0 mL min^–1^), retention time = 6.0 min (opposite enantiomer (**S4a**), 9.9% UV), retention time = 7.9 min (**S4b**, 90.1% UV); 80% e.e. These data are in good agreement with the nonstereospecific spectroscopic data for **S4a**.^37,38,42^

#### Isopropyl ((2*R*,4*S*)-1-Acetyl-6-bromo-2-methyl-1,2,3,4-tetrahydroquinolin-4-yl)carbamate (**S5b**)

The preparation of compound **S5b** was based upon a previously reported procedure.^38,42^ Pyridine (0.114 mL, 1.41 mmol, 3.0 equiv) was added to a solution of compound **S4b** (154 mg, 0.471 mmol, 1.0 equiv) dissolved in anhydrous CH_2_Cl_2_ (4.5 mL) under argon at rt. Acetyl chloride (537 μL, 0.753 mmol, 1.6 equiv) was added dropwise and the reaction mixture was left to stir for 1 h 10 min. After this time the mixture was partitioned with EtOAc (30 mL) and NaHCO_3(aq)_ (30 mL). The aqueous layer was extracted with EtOAc (3 × 30 mL) and the combined organic components washed with H_2_O and brine, dried over Na_2_SO_4_, filtered, and concentrated *in vacuo*. The crude residue was purified using silca gel flash column chromatography (50–100% EtOAc/petroleum ether) to give **S5b** as a brown solid (152 mg, 88%): *R*_*f*_ 0.27 (50% EtOAc/petroleum ether); mp 127–129 °C (from EtOAc) [lit.^42^ 163 °C, lit.^37^ 141–143 °C, (opposite enantiomer)]; [α]_*D*_^25^ = −214.1 (*c* 1.0, MeOH); ^1^H NMR (400 MHz, D_6_-DMSO) δ_H_ 7.63 (1H, d, *J* 8.6), 7.47 (1H, dd, *J* 8.4, 2.2), 7.30 (1H, d, *J* 8.4), 7.21 (1H, d, *J* 2.2), 4.91–4.75 (1H, m), 4.67–4.55 (1H, m), 4.40–4.30 (1H, m), 2.48–2.40 (1H, m), 2.05 (3H, s), 1.25 (3H, d, *J* 6.3), 1.22 (3H, d, *J* 6.3), 1.19–1.13 (1H, m), 1.01 (3H, d, *J* 6.3); LRMS *m*/*z* (ESI^+^) 391 ([M + Na]^+^, 100%). These data are in good agreement with the nonstereospecific spectroscopic data of **S5a**.^37,38,42^

#### Ethyl 4-((2*R*,4*S*)-1-Acetyl-4-((isopropoxycarbonyl)amino)-2-methyl-1,2,3,4-tetrahydroquinolin-6-yl)benzoate (**S6b**)

The preparation of compound **S6b** was based upon a previously reported procedure.^38^ Degassed Na_2_CO_3(aq)_ (2 M, 4.7 mL, 9.40 mmol, 3.93 equiv) was added to a solution of compound **S5b** (879 mg, 2.39 mmol, 1.00 equiv) {4-[(ethyloxy)carbonyl]phenyl}boronic acid (485 mg, 2.50 mmol, 1.05 equiv), and Pd(PPh_3_)_4_ (41 mg, 0.0355 mmol, 0.015 equiv) in degassed DME (9.6 mL). The resulting solution was stirred at 105 °C for 17 h 15 min. After this time, the reaction mixture was cooled to rt and partitioned between EtOAc (20 mL) and water (20 mL). The layers were separated, and the aqueous layer extracted with EtOAc (3 × 50 mL). The combined organic components were washed with water (50 mL) and brine (50 mL), dried over Na_2_SO_4_, filtered, and concentrated *in vacuo*. The resulting gray-brown crude solid was redissolved in EtOAc and filtered through a plug of silica. The silica was washed with EtOAc and the filtrate concentrated *in vacuo* to give **S6b** as a brown solid (843 mg, 80%): *R*_*f*_ 0.31 (acetone:petroleum ether 1:3); mp 146–148 °C (from EtOAc) [opposite enantiomer: 73–75 °C (from acetone)]; [α]_*D*_^25^ = −260.9 (*c* 1.0, MeOH); ^1^H NMR (400 MHz, D_6_-DMSO) δ_H_ 8.08–8.04 (2H, m), 7.83–7.77 (2H, m), 7.69 (1H, d, *J* 8.8), 7.65 (1H, dd, *J* 8.2, 2.2), 7.48–7.43 (2H, m), 4.90–4.80 (1H, m), 4.72–4.60 (1H, m), 4.49–4.39 (1H, m), 4.34 (2H, q, *J* 7.1), 2.48–2.44 (partially hidden by D_6_-DMSO peak) (1H, m), 2.10 (3H, s), 1.34 (3H, t, *J* 7.1), 1.27–1.22 (6H, m), 1.24–1.15 (1H, m), 1.06 (3H, d, *J* 6.4); LRMS *m*/*z* (ESI^+^) 461 ([M + Na]^+^, 100%). The LRMS and ^1^H NMR data are in good agreement with the literature values for **S6a**.^38^

#### Ethyl 4-((2*R*,4*S*)-1-Acetyl-4-amino-2-methyl-1,2,3,4-tetrahydroquinolin-6-yl)benzoate (**S7b**)

The preparation of compound **S7b** was based upon a previously reported procedure.^38^ AlCl_3_ (76 mg, 0.570 mmol, 5.0 equiv) was added to a solution of compound **S6b** (50 mg, 0.114 mmol, 1.0 equiv) dissolved in anhydrous CH_2_Cl_2_ (0.9 mL) at 0 °C, and the resulting suspension was stirred for 20 min. After this time, a solution of anhydrous NEt_3_ (0.2 mL, 1.4 mmol, 12 equiv) in dry MeOH (0.2 mL) was added dropwise and the resulting suspension was stirred for a further 1 h 30 min. The reaction mixture was then diluted with EtOAc (5 mL) and saturated solution of Rochelle’s salt (10 mL), and the solution left to stir for 30 min. The suspension was then filtered through Celite, eluting with EtOAc, saturated NaHCO_3(aq)_, acetone, and MeOH. The organic components were removed *in vacuo* and the aqueous components partitioned between saturated NaHCO_3(aq)_ (10 mL) and EtOAc (10 mL). The aqueous layer was washed with EtOAc (3 × 10 mL) and the combined organic components were dried over Na_2_SO_4_, filtered, and concentrated *in vacuo*. The crude residue was purified using silica gel flash column chromatography (0–10% MeOH/CH_2_Cl_2_) to give **S7b** as a colorless solid (33 mg, 82%). *R*_*f*_ 0.69 (10% MeOH/CH_2_Cl_2_); [α]_D_^25^ = −264.3 (*c* 1.0, CHCl_3_); mp 120–125 °C (from toluene) [opposite enantiomer: 38–45 °C (from EtOAc)]; ^1^H NMR (400 MHz, CDCl_3_) δ_H_ 8.14–8.09 (2H, m), 7.78–7.76 (1H, m), 7.71–7.67 (2H, m), 7.53 (1H, dd, *J* 8.2, 1.9), 7.20 (1H, d, *J* 8.2), 4.91–4.78 (1H, m), 4.41 (2H, q, *J* 7.1), 3.81 (1H, dd, *J* 12.0, 4.4), 2.57 (1H, ddd, *J* 12.8, 8.7, 4.4), 2.16 (3H, s), 1.65 (2H, br s), 1.42 (3H, t, *J* 7.1), 1.23–1.18 (1H, m), 1.17 (3H, d, *J* 6.4); LRMS *m*/*z* (ESI^+^) 375 ([M + Na]^+^, 25%). The ^1^H NMR and LRMS data are in good agreement with the literature value for **S7a**.

#### Ethyl 4-{(2*S*,4*R*)-1-Acetyl-4-[(1-benzothiophen-6-yl)amino]-2-methyl-1,2,3,4-tetrahydroquinolin-6-yl}benzoate (**16**)

Compound **S7a** (100 mg, 0.284 mmol, 1.0 equiv), 6-bromobenzothiophene (121 mg, 0.568 mmol, 2 equiv), BrettPhos (49 mg, 0.0913 mmol, 0.32 equiv), BrettPhos Pd G3 (21 mg, 0.0232 mmol, 0.08 equiv), and NaO^*t*^Bu (33 mg, 0.343 mmol, 1.2 equiv) were added to a dry microwave vial and the vial was purged with argon. Degassed anhydrous toluene (1.7 mL) was added and the resulting suspension was stirred for 15 h at 70 °C. After this time the suspension was filtered through Celite, eluting with toluene and Et_2_O, and the filtrate was concentrated *in vacuo*. The resulting residue was redissolved in EtOAc (50 mL) and washed with H_2_O (40 mL) and brine (40 mL). The organic components were dried over Na_2_SO_4_, filtered, and concentrated *in vacuo*. The crude material was purified using silica gel flash column chromatography (0–60% EtOAc/petroleum ether) to give **16** as a yellow solid (120 mg, 87%, 94.8% purity by HPLC). To achieve a higher purity, 50 mg of **16** was further purified using semipreparative HPLC and lyophilized to give **16** as an off-white solid (>99.9% purity by HPLC)**:***R*_*f*_ 0.43 (10% EtOAc/CH_2_Cl_2_); [α]_D_^25^ = +338.1 (*c* 1.0, CHCl_3_); mp 78–84 °C (from CHCl_3_); *v̅*_max_ (thin film)/cm^–1^ 3350 (N–H, w), 1711 (C=O, s), 1646 (C=O, s), 1606 (s), 1487 (s), 1277 (s); ^1^H NMR (400 MHz, CD_3_CN) δ_H_ 8.09–7.89 (2H, m), 7.66–7.57 (4H, m), 7.56–7.54 (1H, m), 7.37 (1H, d, *J* 8.2), 7.18 (1H, dd, *J* 5.4, 0.8), 7.16–7.12 (2H, m), 6.91 (1H, dd, *J* 8.7, 2.2), 4.97 (1H, d, *J* 8.0), 4.86–4.73 (1H, m), 4.40 (1H, ddd, *J* 12.2, 8.0, 4.2), 4.31 (2H, q, *J* 7.1), 2.71 (1H, ddd, *J* 12.6, 8.5, 4.2), 2.17 (3H, s), 1.33 (3H, t, *J* 7.1), 1.35–1.24 (1H, m), 1.14 (3H, d, *J* 6.3); ^13^C NMR (151 MHz, CD_3_CN) δ_C_ 170.0, 166.9, 146.8, 145.7, 142.8, 139.5, 138.2, 137.6, 132.5, 130.8, 130.4, 127.9, 127.7, 126.5, 125.1, 124.6, 123.3, 122.5, 114.7, 104.6, 61.8, 50.7, 48.6, 41.6, 23.4, 21.7, 14.6; LRMS *m*/*z* (ESI^+^) 485 ([M + H]^+^, 2.6%); HRMS *m*/*z* (ESI^+^) [found: 485.18976, C_29_H_29_O_3_N_2_^32^S, requires [M + H]^+^ 485.18934]; HPLC (Method 1), retention time = 12.4 min, > 99.9%; Chiral HPLC, Chiral AD-H column (40:60 hexane/IPA, 1.0 mL min^–1^), retention time = 7.7 min (**16**, 91.4% UV), retention time = 10.1 min (opposite enantiomer (**17**), 8.6% UV); 83% e.e.

#### 4-{(2*S*,4*R*)-1-Acetyl-4-[(1-benzothiophen-6-yl)amino]-2-methyl-1,2,3,4-tetrahydroquinolin-6-yl}benzoic Acid (**9**)

Compound **16** (55 mg, 0.113 mmol, 1 equiv) was dissolved in EtOH (0.71 mL). NaOH_(aq)_ (2 M, 565 μL, 1.13 mmol, 10 equiv) was added over a period of 1 h and the resulting solution was left to stir at rt for 3 h. The solution was then acidified to ∼pH 3 with 1 M HCl_(aq)_ and diluted with H_2_O (5 mL) and EtOAc (10 mL). The aqueous components were then extracted with EtOAc (3 × 10 mL). The combined organic components were dried over Na_2_SO_4_, filtered, and concentrated *in vacuo* to give **9** as a yellow solid (45 mg, 87%, 94.7% purity by HPLC). To achieve higher purity, **9** was purified using semipreparative HPLC and lyophilized to give **9** as an off-white solid: *R*_*f*_ 0.29 (50% EtOAc/petroleum ether, 1% acetic acid); [α]_D_^25^ = +308.8 (*c* 0.66, CHCl_3_); mp 115–120 °C (from CHCl_3_); *v̅*_max_ (thin film)/cm^–1^ 3362 (N–H, w), 3034 (O–H, w), 1704 (C=O, m), 1606 (s), 1488 (m); ^1^H NMR (400 MHz, CD_3_CN) δ_H_ 8.02–7.94 (2H, m), 7.66–7.58 (4H, m), 7.55 (1H, d, *J* 1.4), 7.38 (1H, d, *J* 8.2), 7.19 (1H, dd, *J* 5.5, 0.7), 7.16–7.11 (2H, m), 6.92 (1H, dd, *J* 8.6, 2.2), 4.86–4.73 (1H, m), 4.41 (1H, dd, *J* 12.1, 4.1), 2.71 (1H, ddd, *J* 12.5, 8.6, 4.1), 2.18 (3H, s), 1.36–1.24 (1H, m), 1.14 (3H, d, *J* 6.3); ^13^C NMR (151 MHz, CD_3_CN) δ_C_ 170.1, 167.6, 146.8, 145.8, 142.8, 139.6, 138.3, 137.6, 132.5, 131.2, 130.0, 127.9, 127.7, 126.6, 125.1, 124.6, 123.3, 122.5, 114.7, 104.6, 50.7, 48.7, 41.6, 23.4, 21.7; LRMS *m*/*z* (ESI^–^) 455 ([M–H]^−^, 100%); HRMS *m*/*z* (ESI^+^) [Found: 457.15808, C_29_H_29_O_3_N_2_^32^S, requires [M + H]^+^ 457.15804]; HPLC (Method 2) retention time = 9.18 min, 99.0%; Chiral HPLC, Chiral AD-H column (50:50 hexane/IPA, 1.0 mL min^–1^), retention time = 7.3 min (**9**, 91.2% UV), retention time = 11.0 min (opposite enantiomer (**10**), 8.8% UV); 82% e.e.

#### Ethyl 4-{(2*R*,4*S*)-1-Acetyl-4-[(1-benzothiophen-6-yl)amino]-2-methyl-1,2,3,4-tetrahydroquinolin-6-yl}benzoate (**17**)

Compound **S7b** (140 mg, 0.398 mmol, 1.0 equiv), 6-bromobenzo[*b*]thiophene (169 mg, 0.793 mmol, 2.0 equiv), BrettPhos (68 mg, 0.127 mmol, 0.32 equiv), BrettPhos Pd G3 (29 mg, 0.032 mmol, 0.080 equiv), and NaO^*t*^Bu (46 mg, 0.479 mmol, 1.2 equiv) were added to a dry microwave vial, which was subsequently purged with argon. Anhydrous, degassed toluene (2.4 mL) was added, and the resulting suspension was stirred for 15 h at 70 °C. After this time, the suspension was filtered through Celite, eluting with toluene and Et_2_O, and the filtrate was concentrated *in vacuo*. The resulting residue was redissolved in EtOAc (50 mL) and washed with H_2_O (40 mL) and brine (40 mL). The organic components were dried over Na_2_SO_4_, filtered, and concentrated *in vacuo*. The crude material was purified using silica gel flash column chromatography (0–60% EtOAc/petroleum ether) to give **17** as a yellow solid (132 mg, 68%, 96.2% purity by HPLC). To achieve higher purity 66 mg of compound **17** was further purified using semipreparative HPLC, and lyophilized to give **17** as an off-white solid (>99.9% purity by HPLC): *R*_*f*_ 0.43 (10% EtOAc/CH_2_Cl_2_); [α]_D_^25^ = −285.9 (*c* 1.0, CHCl_3_); mp 102–105 °C (from CHCl_3_) [opposite enantiomer: 78–84 °C (from CHCl_3_)]; ^1^H NMR (400 MHz, CD_3_CN) δ_H_ 8.03–7.94 (2H, d, *J* 8.0), 7.65–7.57 (4H, m), 7.57–7.54 (1H, m), 7.38 (1H, d, *J* 8.2), 7.19 (1H, dd, *J* 5.4, 0.7), 7.16–7.13 (2H, m), 6.91 (1H, dd, *J* 8.6, 2.2), 4.97 (1H, d, *J* 8.0), 4.87–4.73 (1H, m), 4.41 (1H, ddd, *J* 12.1, 8.0, 4.1), 4.31 (2H, q, *J* 7.1), 2.71 (1H, ddd, *J* 12.5, 8.6, 4.1), 2.18 (3H, s), 1.34 (3H, t, *J* 7.1), 1.35–1.25 (1H, m), 1.14 (3H, d, *J* 6.3); ^13^C NMR (151 MHz, CD_3_CN) δ_C_ 170.0, 167.0, 146.8, 145.7, 142.8, 139.5, 138.3, 137.6, 132.5, 130.9, 130.4, 127.9, 127.7, 126.5, 125.1, 124.6, 123.3, 122.5, 114.7, 104.6, 61.8, 50.7, 48.7, 41.6, 23.4, 21.7, 14.6; LRMS: *m*/*z* (ESI^+^) 507 ([M + Na]^+^, 28%); HPLC (Method 1), retention time = 12.4 min, > 99.9%; Chiral HPLC, Chiral AD-H column (40:60 hexane/IPA, 1.0 mL min^–1^), retention time = 7.2 min (opposite enantiomer (**16**), 5.6% UV), retention time = 10.1 min (**17**, 94.4% UV); 89% e.e.

#### 4-{(2*R*,4*S*)-1-Acetyl-4-[(1-benzothiophen-6-yl)amino]-2-methyl-1,2,3,4-tetrahydroquinolin-6-yl}benzoic Acid (**10**)

Compound **17** (67 mg, 0.138 mmol, 1 equiv) was dissolved in EtOH (0.86 mL), NaOH_(aq)_ (2 M, 695 μL, 1.39 mmol, 10 equiv) was added over 1 h, and the resulting solution was left to stir at rt for 3 h. After this time, the solution was acidified to approximately pH 3 with 1 M HCl_(aq)_ and diluted with H_2_O (5 mL) and EtOAc (10 mL). The aqueous components were then extracted with EtOAc (3 × 10 mL). The combined organic components were dried over Na_2_SO_4_, filtered, and concentrated *in vacuo*. The crude material was purified using semipreparative HPLC, and lyophilized to give **10** as an off-white solid (20 mg, 32%): *R*_*f*_ 0.63 (50% EtOAc/petroleum ether, 1% acetic acid); [α]_D_^25^ = −247.7 (*c* 0.47, CHCl_3_); mp 118–123 °C (from CH_2_Cl_2_) [opposite enantiomer: 115–120 °C]; ^1^H NMR (400 MHz, CD_3_CN) δ_H_ 8.02–7.95 (2H, m), 7.66–7.58 (4H, m), 7.55 (1H, d, *J* 1.4), 7.38 (1H, d, *J* 8.2), 7.19 (1H, d, *J* 5.5, 0.7), 7.16–7.13 (2H, m), 6.92 (1H, dd, *J* 8.6, 2.2), 4.86–4.73 (1H, m), 4.41 (1H, dd, *J* 12.1, 4.1), 2.71 (1H, ddd, *J* 12.5, 8.6, 4.1), 2.18 (3H, s), 1.30 (1H, m), 1.15 (3H, d, *J* 6.3); ^13^C NMR (151 MHz, CD_3_CN) δ_C_ 170.1, 167.6, 146.8, 145.8, 142.8, 139.6, 138.2, 137.7, 132.5, 131.2, 130.0, 127.9, 127.7, 126.6, 125.1, 124.6, 123.3, 122.5, 114.7, 104.6, 50.7, 48.7, 41.6, 23.4, 21.7; LRMS: *m*/*z* (ESI^+^) 479 ([M + Na]^+^, 25%); HPLC (Method 1), retention time = 10.1 min, 99.8%; Chiral HPLC, Chiral AD-H column (hexane/IPA, 1.0 mL min^–1^), retention time = 6.7 min (opposite enantiomer (**9**), 5.5% UV), Retention time = 9.8 min (**10**, 94.5% UV); 89% e.e.

#### Ethyl 4-{(2*S*,4*R*)-1-Acetyl-4-[(1-benzofuran-6-yl)amino]-2-methyl-1,2,3,4-tetrahydroquinolin-6-yl}benzoate (**18**)

Compound **S7a** (133 mg, 0.377 mmol, 1.0 equiv), 6-bromobenzofuran (149 mg, 0.756 mmol, 2.0 equiv), BrettPhos (65 mg, 0.121 mmol, 0.32 equiv), BrettPhos Pd G3 (27 mg, 0.0298 mmol, 0.079 equiv), and NaO^*t*^Bu (44 mg, 0.458 mmol, 1.2 equiv) were added to a dry microwave vial, which was subsequently purged with argon. Degassed, anhydrous toluene (2.2 mL) was added, and the resulting suspension was stirred for 15 h at 70 °C. After this time, the suspension was filtered through Celite, eluting with toluene and Et_2_O, and the filtrate concentrated *in vacuo*. The resulting residue was redissolved in EtOAc (50 mL) and washed with H_2_O (40 mL) and brine (40 mL). The organic components were dried over Na_2_SO_4_, filtered, and concentrated *in vacuo*. The crude material was purified using silica gel flash column chromatography (0–50% EtOAc/petroleum ether) to give **18** as a yellow solid (120 mg, 68%, 94.6% purity by HPLC). To achieve a higher purity, 60 mg of **18** was purified using semipreparative HPLC, and lyophilized to give **18** as a light-green solid: *R*_*f*_ 0.17 (30% EtOAc/petroleum ether); [α]_D_^25^ = +258.7 (*c* 1.0, CHCl_3_); mp 84–87 °C (from CHCl_3_); *v̅*_max_ (thin film)/cm^–1^ 3358 (N–H, w), 1713 (C = O, s), 1629 (s), 1608 (s), 1488 (s); ^1^H NMR (400 MHz, CD_3_CN) δ_H_ 8.04–7.89 (2H, m), 7.65–7.58 (3H, m), 7.57–7.55 (1H, m), 7.48 (1H, d, *J* 2.2), 7.40–7.34 (2H, m), 6.82–6.80 (1H, m), 6.78 (1H, dd, *J* 8.3, 2.1), 6.68 (1H, dd, *J* 2.2, 1.0), 4.93 (1H, d, *J* 8.1), 4.84–4.74 (1H, m), 4.43–4.35 (1H, dd, *J* 9.1, 4.4), 4.32 (2H, q, *J* 7.1), 2.71 (1H, ddd, *J* 12.5, 8.5, 4.1), 2.17 (3H, s), 1.34 (3H, t, *J* 7.1), 1.34–1.24 (1H, m), 1.14 (3H, d, *J* 6.3); ^13^C NMR (151 MHz, CD_3_CN) δ_C_ 170.0, 167.0, 157.7, 147.5, 145.7, 143.9, 139.6, 138.2, 137.6, 130.9, 130.4, 127.8, 127.7, 126.5, 123.4, 122.5, 119.1, 112.4, 107.4, 95.4, 61.8, 50.8, 48.7, 41.6, 23.4, 21.7, 14.6; LRMS *m*/*z* (ESI^+^) 469 ([M + H]^+^, 11%); HRMS *m*/*z* (ESI^+^) [Found: 469.21239, C_29_H_29_O_4_N_2_, requires [M + H]^+^ 469.21218]; HPLC (Method 1), retention time = 11.9 min, 99.7%; Chiral HPLC, Chiral AD-H column (80:20 hexane/IPA, 1.0 mL min^–1^), retention time = 19.7 min (**18**, 91.7% UV), retention time = 28.0 min (opposite enantiomer, 8.3% UV); 83% e.e.

#### 4-((2*S*,4*R*)-1-Acetyl-4-[(1-benzofuran-6-yl)amino]-2-methyl-1,2,3,4-tetrahydroquinolin-6-yl)benzoic Acid (**11**)

Compound **18** (48 mg, 0.102 mmol, 1.0 equiv) was dissolved in EtOH (0.63 mL). NaOH_(aq)_ (2 M, 0.512 mL, 1.02 mmol, 10 equiv) was added portion-wise over 30 min, and the resulting solution was stirred for 1 h. The solution was then acidified to ∼pH 5 using HCl_(aq)_ (1 M), upon which a precipitate formed, and then extracted with EtOAc (3 × 10 mL). The organic components were dried over Na_2_SO_4_, filtered, and concentrated *in vacuo*. The crude material was purified using semipreparative HPLC and the fractions containing **11** were combined and lyophilized to give **11** as a colorless solid (9 mg, 20%): *R*_*f*_ 0.54 (10% MeOH/CH_2_Cl_2_); [α]_D_^25^ = +177.8 (*c* 0.5, MeOH); mp 170–173 °C (from MeOH); *v̅*_max_ (thin film)/cm^–1^ 3404 (N–H, m), 1700 (C=O, m), 1628 (N–H, m); ^1^H NMR (600 MHz, MeOD) δ_H_ 8.06–7.95 (2H, m), 7.68–7.62 (2H, m), 7.59 (2H, d, *J* 8.4), 7.47 (1H, d, *J* 2.2), 7.40 (1H, d, *J* 8.1), 7.36 (1H, d, *J* 9.1), 6.83–6.72 (2H, m), 6.65 (1H, d, *J* 2.1), 4.93–4.81 (partially hidden by H_2_O peak) (1H, m), 4.33 (1H, dd, *J* 12.3, 4.1), 2.74 (1H, ddd, *J* 12.6, 8.6, 4.2), 2.26 (3H, s), 1.41–1.33 (1H, m), 1.20 (3H, d, *J* 6.4); ^13^C NMR (151 MHz, MeOD) δ_C_ 172.2, 170.4, 158.3, 147.8, 145.9, 143.8, 140.5, 139.3, 137.5, 131.7, 131.3, 127.9, 127.7, 126.8, 124.1, 122.3, 119.6, 112.3, 107.3, 95.7, 51.3, 49.6, 41.7, 23.0, 21.5; LRMS *m*/*z* (ESI^–^) 439 ([M-H]^−^, 77%); HRMS *m*/*z* (ESI^+^) [Found: 441.1812, C_27_H_25_O_4_N_2_, requires [M + H]^+^ 441.1809]; HPLC (Method 1), retention time = 9.7 min, 98.9%; Chiral HPLC, Chiral IG-3 column (70:30 hexane/IPA, 0.1% TFA, 1.0 mL min^–1^), retention time = 13.8 min (**11**, 92.5% UV), retention time = 18.4 min (opposite enantiomer, 7.5% UV); 85% e.e.

#### Ethyl 4-{(2*S*,4*R*)-1-Acetyl-4-[(1-benzothiophen-5-ylamino]-2-methyl-1,2,3,4-tetrahydroquinolin-6-yl}benzoate (**19**)

Compound **S7a** (100 mg, 0.284 mmol, 1.0 equiv), 5-bromobenzothiophene (121 mg, 0.568 mmol, 2.0 equiv), BrettPhos (49 mg, 0.0913 mmol, 0.32 equiv), BrettPhos Pd G3 (21 mg, 0.0232 mmol, 0.082 equiv), and NaO^*t*^Bu (33 mg, 0.343 mmol, 1.2 equiv) were added to a dry microwave vial, and the vial was purged with argon. Degassed anhydrous toluene (1.7 mL) was added and the resulting suspension was stirred for 15 h at 70 °C. After this time, the suspension was filtered through Celite, eluting with toluene and Et_2_O, and the filtrate was concentrated *in vacuo*. The resulting residue was redissolved in EtOAc (50 mL) and washed with H_2_O (40 mL) and brine (40 mL). The organic components were dried over Na_2_SO_4_, filtered, and concentrated *in vacuo*. The crude material was purified using silica gel flash column chromatography (0–60% EtOAc/petroleum ether) to give **19** as a yellow solid (88 mg, 66%, 94.7% purity by HPLC). To achieve a higher purity, 44 mg of **19** was further purified using semipreparative HPLC and the fractions containing **19** lyophilized to give **19** as an off-white solid**:***R*_*f*_ 0.34 (50% EtOAc/petroleum ether); [α]_D_^25^=+324.5 (*c* 1.0, CHCl_3_); mp 95–99 °C (from CHCl_3_); *v̅*_max_ (thin film)/cm^–1^ 3360 (N–H, w), 1713 (C = O, s), 1647 (N–H, s), 1606 (s), 1488 (s), 1278 (s); ^1^H NMR (400 MHz, CD_3_CN) δ_H_ 8.04–7.91 (2H, m), 7.68 (1H, d, *J* 8.7), 7.63–7.54 (4H, m), 7.43 (1H, d, *J* 5.4), 7.38 (1H, d, *J* 8.0), 7.13 (1H, d, *J* 5.5, 0.8), 7.10–7.08 (1H, m), 6.93 (1H, dd, *J* 8.7, 2.3), 4.87 (1H, d, *J* 8.0), 4.83–4.74 (1H, m), 4.40 (1H, ddd, *J* 12.1, 8.0, 4.1), 4.31 (2H, q, *J* 7.1), 2.72 (1H, ddd, *J* 12.5, 8.6, 4.1), 2.18 (3H, s), 1.34 (3H, t, *J* 7.1), 1.35–1.22 (1H, m), 1.14 (3H, d, *J* 6.3); ^13^C NMR (151 MHz, CD_3_CN) δ_C_ 170.0, 167.0, 146.7, 145.7, 142.2, 139.7, 138.2, 137.6, 130.9, 130.4, 130.0, 128.1, 127.9, 127.7, 126.5, 124.4, 123.8, 123.4, 115.1, 106.1, 61.8, 50.8, 48.7, 41.6, 23.4, 21.7, 14.6; LRMS *m*/*z* (ESI^+^) 485 ([M + H]^+^, 2.6%); HRMS *m*/*z* (ESI^+^) [Found: 485.18976, C_29_H_29_O_3_N_2_^32^S, requires [M + H]^+^ 485.18934]; HPLC (Method 1), retention time = 12.3 min, > 99.9%; Chiral HPLC, Chiral AD-H column (50:50 hexane/EtOH, 1.0 mL min^–1^), retention time = 10.9 min (opposite enantiomer, 6.7% UV), retention time = 13.9 min (**19**, 93.3% UV); 87% e.e.

#### 4-((2*S*,4*R*)-1-Acetyl-4-[(1-benzothiophen-5-yl)amino]-2-methyl-1,2,3,4-tetrahydroquinolin-6-yl)benzoic Acid (**12**)

Compound **19** (36 mg, 0.0769 mmol, 1 equiv) was dissolved in EtOH (0.46 mL). NaOH(aq) (2 M, 0.371 mL, 0.742 mmol, 10 equiv) was added over the course of 30 min, and the resulting solution was left to stir for 3 h at rt. After this time the solution was acidified to ∼pH 5, upon which a precipitate formed. EtOAc (10 mL) was added, upon which the precipitate redissolved, and the aqueous and organic components were separated. The aqueous components were extracted with EtOAc (3 × 10 mL), and the combined organic components were dried over Na_2_SO_4_, filtered, and concentrated *in vacuo*. The crude material was purified using semipreparative HPLC to give **12** as an off-white solid (14 mg, 41%): *R*_*f*_ 0.61 (50% EtOAc/petroleum ether, 1% acetic acid); [α]_D_^25^=+170.1 (*c* 0.37, CHCl_3_); mp 137–143 °C (from CHCl_3_); *v̅*_max_ (thin film)/cm^–1^ 3352 (N–H, w), 1710 (C=O, m), 1638 (m), 1605 (s), 1513 (m); ^1^H NMR (400 MHz, CD_3_CN) δ_H_ 8.03–7.95 (2H, m), 7.68 (1H, d, *J* 8.7), 7.64–7.59 (3H, m), 7.59–7.57 (1H, m), 7.44 (1H, d, *J* 5.4), 7.39 (1H, d, *J* 8.1), 7.14 (1H, dd, *J* 5.4, 0.8), 7.10–7.08 (1H, m), 6.94 (1H, dd, *J* 8.7, 2.3), 4.88–4.73 (1H, m), 4.40 (1H, dd, *J* 12.1, 4.1), 2.72 (1H, ddd, *J* 12.5, 8.5, 4.1), 2.18 (3H, s), 1.35–1.24 (1H, m), 1.15 (3H, d, *J* 6.3); ^13^C NMR (151 MHz, CD_3_CN) δ_C_ 170.1, 167.7, 146.7, 145.7, 142.2, 139.7, 138.2, 137.7, 131.2, 130.2, 130.0, 128.1, 127.9, 127.7, 126.5, 124.4, 123.8, 123.4, 115.1, 106.1, 50.8, 48.7, 41.6, 23.4, 21.7; LRMS: *m*/*z* (ESI^+^) 457 ([M + H]^+^, 100%); HRMS *m*/*z* (ESI^–^) [found: 455.14422, C_27_H_23_O_3_^32^S, requires [M–H]^−^ 455.14349]; HPLC (Method 1), retention time = 9.97 min, > 99.9%; Chiral HPLC, Chiral IG-3 column (70:30 hexane/IPA, 0.1% TFA, 1.0 mL min^–1^), retention time = 15.0 min (**12**, 92.35% UV), retention time = 18.5 min (opposite enantiomer, 7.65% UV); 85% e.e.

#### (1-(*tert*-Butoxycarbonyl)-1*H*-indol-5-yl)boronic Acid (**S8**)

Di-*tert*-butyl dicarbonate (542 mg, 2.48 mmol, 4.0 equiv) and DMAP (15 mg, 0.123 mmol, 0.2 equiv) were added to a solution of 5-indoylboronic acid (100 mg, 0.621 mmol, 1.0 equiv) in MeCN (2.8 mL). The resulting solution was stirred at rt for 1 h, after which time a solution of aqueous citric acid (0.5 M, 50 mL) was added, and the aqueous components were extracted with EtOAc (3 × 50 mL). The organic components were washed with brine (100 mL), dried over Na_2_SO_4_, filtered, and concentrated *in vacuo*. The crude material was purified using silica gel flash column chromatography (0–50% EtOAc/petroleum ether) to give **S8** as an off-white solid (80 mg, 49%). *R*_*f*_ 0.48 (50% EtOAc/petroleum ether); mp 82–85 °C (from EtOAc); *v̅*_max_ (thin film)/cm^–1^ 2979 (C–H, w), 1734 (C=O, m), 1331 (m); ^1^H NMR (600 MHz, CDCl_3_) δ_H_ 8.55–8.54 (1H, m), 8.32–8.26 (1H, m), 8.26–8.23 (1H, m), 7.67 (1H, d, *J* 3.7), 6.74 (1H, d, *J* 3.7), 1.72 (9H, s); ^13^C NMR (151 MHz, CDCl_3_) δ_C_ 149.9, 138.2, 131.5, 130.6, 129.6, 126.4, 124.5, 114.9, 107.9, 84.1, 28.4; ^11^B NMR (128 MHz, CDCl_3_) δ_B_ 4.31; LRMS *m*/*z* (ESI^+^) 284 ([M + Na]^+^, 87%). The *R*_*f*_, ^1^H and ^13^C data are in agreement with the literature values.^51^

#### *tert*-Butyl 5-{(2*S*,4*R*)-(1-Acetyl-6-[4-(ethoxycarbonyl)phenyl]-2-methyl-1,2,3,4-tetrahydroquinolin-4-yl)amino}-1*H*-indole-1-carboxylate (**S9**)

Compound **S7a** (47 mg, 0.133 mmol, 1 equiv) and boronic acid **S8** (69 mg, 0.264 mmol, 2 equiv) were dissolved in anhydrous CH_2_Cl_2_ (1.0 mL) in a round-bottom flask containing activated 3 Å molecular sieves, connected to a drying tube containing P_2_O_5_, open to the atmosphere. NEt_3_ (55 μL, 0.395 mmol, 3 equiv) was added and the resulting solution was left to stir at rt for 15 h. The molecular sieves were removed using filtration, and the filtrate was concentrated *in vacuo*. The resulting residue was purified using silica gel flash column chromatography column (0–15% EtOAc/CH_2_Cl_2_) to give **S9** as a colorless solid (40 mg, 53%): *R*_*f*_ 0.20 (10% EtOAc/CH_2_Cl_2_); [α]_D_^25^ = +244.3 (*c* 1.0, CHCl_3_); mp 117–121 °C (from CH_2_Cl_2_); *v̅*_max_ (thin film)/cm^–1^ 3347 (N–H, w), 2978 (C–H, w), 1720 (C=O, s), 1653 (N–H, m); ^1^H NMR (400 MHz, CDCl_3_) δ_H_ 8.06–8.01 (2H, m), 8.00–7.91 (1H, m), 7.68–7.64 (1H, m), 7.58–7.49 (4H, m), 7.28–7.21 (1H, m), 6.80–6.79 (1H, m), 6.79–6.73 (1H, m), 6.40 (1H, d, *J* 3.6), 4.99–4.82 (1H, m), 4.37 (2H, q, *J* 7.1), 4.31 (1H, dd, *J* 12.0, 4.2), 3.84 (1H, br s), 2.72 (1H, ddd, *J* 12.5, 8.6, 4.2), 2.26 (3H, s), 1.65 (9H, s), 1.38 (3H, t, *J* 7.1), 1.36–1.27 (1H, m), 1.21 (3H, d, *J* 6.3);^13^C NMR (126 MHz, CDCl_3_) δ_C_ 169.6, 166.5, 149.9, 144.7, 143.2, 138.8, 137.9, 136.6, 131.9, 130.2, 129.4, 129.1, 127.0, 126.5, 126.0, 123.0, 116.1, 112.7, 107.0, 103.7, 83.4, 61.1, 51.0, 47.8, 41.4, 28.4, 23.3, 21.5, 14.5; LRMS *m*/*z* (ESI^+^) 568 ([M + H]^+^, 37%); HRMS *m*/*z* (ESI^+^) [found: 568.2803, C_34_H_38_O_5_N_3_, requires [M + H]^+^ 568.2806]; HPLC (Method 1), retention time = 13.1 min, 98.1%; Chiral HPLC, Chiral IG-3 column (50:50 hexane:IPA, 1.0 mL min^–1^), retention time = 11.2 min (**S9**, 92.0% UV), retention time = 17.0 (opposite enantiomer, 8.0% UV); 84% e.e.

#### Ethyl 4-{(2*S*,4*R*)-1-Acetyl-4-[(1*H*-indol-5-yl)amino]-2-methyl-1,2,3,4-tetrahydroquinolin-6-yl}benzoate (**20**)

TFA (0.8 mL) was added dropwise to a solution of **S8** (85 mg, 0.150 mmol, 1 equiv) in CH_2_Cl_2_ (0.8 mL), and the resulting solution was stirred at room temperature for 30 min. After this time, the solution was diluted with EtOAc (15 mL) and neutralized with saturated NaHCO_3(aq)_ (10 mL). The organic and aqueous components were separated, and the aqueous components were extracted with EtOAc (3 × 10 mL). The organic components were combined, dried over Na_2_SO_4_, filtered, and concentrated *in vacuo*. The crude material was purified using silica gel flash column chromatography (0–50% EtOAc/petroleum ether) to give **20** as a yellow solid (32 mg, 46%): *R*_*f*_ 0.28 (50% EtOAc/pentane); [α]_D_^25^ = +280.4 (*c* 1.0, CHCl_3_); mp 104–108 °C (from EtOAc); *v̅*_max_ (thin film)/cm^–1^ 2975 (C–H, m), 1709 (C = O, m), 1647 (N–H, m); ^1^H NMR (600 MHz, CD_3_CN) δ_H_ 9.00 (1H, s), 8.00 (2H, d, *J* 8.2), 7.67–7.66 (1H, m), 7.63 (2H, d, *J* 8.2), 7.60 (1H, dd, *J* 8.2, 2.2), 7.37 (1H, d, *J* 8.2), 7.26 (1H, d, *J* 8.6), 7.13–7.10 (1H, m), 6.85–6.82 (1H, m), 6.75 (1H, dd, *J* 8.6, 2.3), 6.23–6.21 (1H, m), 4.81–4.74 (1H, m), 4.38–4.29 (3H, m), 2.71 (1H, ddd, *J* 12.4, 8.5, 4.0), 2.17 (3H, s), 1.34 (3H, t, *J* 7.1), 1.30–1.22 (1H, m), 1.14 (3H, d, *J* 6.3); ^13^C NMR (151 MHz, CD_3_CN) δ_C_ 170.0, 167.0, 145.8, 142.4, 140.5, 138.2, 137.5, 131.4, 130.9, 130.4, 130.0, 127.7, 126.3, 125.9, 123.7, 112.9, 112.9, 103.4, 101.6, 61.8, 51.6, 48.8, 41.7, 23.3, 21.8, 14.6; LRMS *m*/*z* (ESI^+^) 468 ([M + H]^+^, 13%); HRMS *m*/*z* (ESI^+^) [found: 468.2281, C_29_H_30_O_3_N_3_ requires [M + H]^+^ 468.2282]; HPLC (Method 1), retention time = 8.8 min, 97.5%; Chiral HPLC, Chiral IF-3 column (70:30 hexane/IPA, 1.0 mL min^–1^), retention time = 14.0 min (**20**, 95.45% UV), retention time = 16.7 (opposite enantiomer, 4.55% UV); 91% e.e.

#### 4-{(2*S*,4*R*)-1-Acetyl-4-[(1*H*-indol-5-yl)amino]-2-methyl-1,2,3,4-tetrahydroquinolin-6-yl}benzoic Acid (**13**)

Compound **20** (27 mg, 0.0577 mmol, 1.00 equiv) was dissolved in EtOH (0.36 mL). NaOH_(aq)_ (2 M, 289 μL, 0.577 mmol, 10.0 equiv) was added portion wise. A precipitate developed, which was redissolved with addition of further EtOH (0.76 mL), and the resulting solution was stirred for 3 h. Further NaOH_(aq)_ (2 M, 578 μL, 1.16 mmol, 20.0 equiv) was added over a period of 1.5 h, after which time the reaction solution was diluted with CH_2_Cl_2_ (5 mL) and H_2_O (5 mL), and the aqueous and organic layers were separated. The aqueous components were acidified to approximately pH 3, then and extracted with EtOAc (3 × 10 mL). The organic components were dried over Na_2_SO_4_, filtered, and concentrated *in vacuo*. The crude material was purified using silica gel flash column chromatography (reverse phase, 5–40% MeCN/H_2_O) and the pure fractions lyophilized to give **13** as a yellow solid (8.2 mg, 32%): *R*_*f*_ 0.24 (50% MeCN/H_2_O; reverse phase); [α]_D_^25^ = +197.0 (*c* 0.29, MeOH); mp 176–179 °C (lyophilized from MeCN/H_2_O); *v̅*_max_ (thin film)/cm^–1^ 3401 (N–H, br, m), 2924 (C–H, w), 1703 (C=O, s), 1631 (C=O, s);^1^H NMR (400 MHz, MeOD) δ_H_ 8.45 (1H, s), 8.01–7.96 (2H, m), 7.77–7.75 (1H, m), 7.63 (1H, dd, *J* 8.2, 2.2), 7.61–7.57 (2H, m), 7.38 (1H, d, *J* 8.2), 7.25 (1H, d, *J* 8.6), 7.11 (1H, d, *J* 3.1), 6.90–6.88 (1H, m), 6.79 (1H, dd, *J* 8.6, 2.2), 6.25 (1H, dd, *J* 3.1, 0.9), 4.90–4.78 (1H, hidden by H_2_O peak), 4.30 (1H, dd, *J* 12.2, 4.1), 2.74 (1H, ddd, *J* 12.5, 8.6, 4.1), 2.25 (3H, s), 1.37–1.25 (1H, m), 1.19 (3H, d, *J* 6.3); ^13^C NMR (151 MHz, MeOD) δ_C_ 172.2, 170.7, 145.8, 142.3, 141.1, 139.2, 137.5, 132.4, 131.9, 131.3, 130.3, 127.7, 126.5, 125.8, 124.3, 113.2, 112.8, 104.5, 101.5, 52.3, 49.6, 41.8, 23.0, 21.5; LRMS *m*/*z* (ESI^–^) 438 ([M–H]^−^, 8%); HRMS *m*/*z* (ESI^–^) [found: 438.1832, C_27_H_24_0_3_N_3_ requires [M–H]^−^ 438.1823]; HPLC (Method 1) Retention time = 6.9 min, 97.9%; Chiral HPLC, Chiral IG-3 column (70:30 hexane:IPA, 0.1% TFA, 1.0 mL min^–1^) Retention time = 15.4 min (**13**, 89.6% UV), Retention time = 19.8 (opposite enantiomer, 10.4% UV); 79% e.e.

#### Ethyl 4-{(2*S*,4*R*)1-Acetyl-4-[(1,3-benzothiazol-6-yl)amino]-2-methyl-1,2,3,4-tetrahydroquinolin-6-yl}benzoate (**21**)

Compound **S7a** (60 mg, 0.170 mmol, 1.0 equiv), 6-bromobenzothiazole (72 mg, 0.340 mmol, 2.0 equiv), BrettPhos (29 mg, 0.0540 mmol, 0.32 equiv), NaO^*t*^Bu (20 mg, 0.208 mmol, 1.2 equiv), and BrettPhos Pd G3 (12 mg, 0.0132 mmol, 0.078 equiv) were added to a dry microwave vial under Ar. Degassed, anhydrous toluene (1 mL) was added, and the resulting suspension was stirred at 70 °C for 16 h. After this time, the reaction mixture was cooled to rt, and filtered through Celite, eluting with toluene, Et_2_O, and EtOAc. The filtrate was concentrated *in vacuo*, and then redissolved in EtOAc and satd. NaHCO_3(aq)_. The organic and aqueous components were separated, and the aqueous components were extracted with EtOAc (3 × 10 mL). The combined organic components were dried over Na_2_SO_4_, filtered, and concentrated *in vacuo*. The crude material was purified using silica gel flash column chromatography (0–70% EtOAc/pentane) to give **21** as a yellow solid (40 mg, 48%). *R*_*f*_ 0.15 (50% EtOAc/pentane); [α]_D_^25^ = +263.8 (*c* 1.0, CHCl_3_); mp 114–117 °C (from MeCN); *v̅*_max_ (thin film)/cm^–1^ 3341 (N–H, w), 2980 (C–H, w), 1711 (C=O, m), 1642 (C=O, m); ^1^H NMR (500 MHz, CD_3_CN) δ_H_ 8.70 (1H, s), 8.00–7.96 (2H, m), 7.83 (1H, d, *J* 8.9), 7.63–7.59 (3H, m), 7.56–7.53 (1H, m), 7.38 (1H, d, *J* 8.1), 7.22 (1H, d, *J* 2.4), 7.02 (1H, dd, *J* 8.9, 2.4), 5.13 (1H, d, *J* 8.0), 4.86–4.74 (1H, m), 4.41 (1H, ddd, *J* 12.2, 8.0, 4.2), 4.31 (2H, q, *J* 7.1), 2.72 (1H, ddd, *J* 12.5, 8.5, 4.2), 2.17 (3H, s), 1.37–1.25 (4H, m), 1.14 (3H, d, *J* 6.3); ^13^C NMR (126 MHz, CD_3_CN) δ_C_ 170.0, 166.9, 150.6, 147.5, 147.0, 145.6, 139.3, 138.2, 137.6, 136.9, 130.8, 130.4, 127.9, 127.7, 126.6, 124.5, 123.2, 115.8, 103.6, 61.8, 50.7, 48.6, 41.5, 23.4, 21.7, 14.6; LRMS *m*/*z* (ESI^+^) 486 ([M + H]^+^, 100%); HRMS *m*/*z* (ESI^+^) [found: 486.1844, C_28_H_28_O_3_N_3_S, requires [M+H]^+^ 486.1846]; HPLC (Method 1), retention time = 10.7 min, 96.0%; Chiral HPLC, Chiral AD-H column (50:50 hexane/IPA, 1.0 mL min^–1^), retention time = 8.0 min (opposite enantiomer, 7.5% UV), retention time = 10.6 (**21**, 92.5% UV); 85% e.e.

#### 4-{(2*S*,4*R*)-1-Acetyl-4-[(1,3-benzothiazol-6-yl)amino]-2-methyl-1,2,3,4-tetrahydroquinolin-6-yl}benzoic acid (**14**)

Compound **21** (61 mg, 0.126 mmol, 1 equiv) was dissolved in EtOH (0.79 mL) and to this solution, NaOH_(aq)_ (2 M, 0.63 mL, 1.26 mmol, 10 equiv) was added portion wise. The solution was stirred for 1 h 20 min, after which time, HCl_(aq)_ (1 M) was added until a red precipitate formed. The precipitate was redissolved in EtOAc (10 mL) and the solution was diluted with H_2_O (5 mL). The aqueous and organic components were separated, and the aqueous component was extracted with EtOAc (3 × 10 mL). The combined organic components were dried over Na_2_SO_4_, filtered, and concentrated *in vacuo*. The crude material was purified using silica gel flash column chromatography (reverse phase, 5–31% MeCN/H_2_O) and lyophilized to give **14** as an orange-brown powder (40 mg, 69%): *R*_*f*_ 0.23 (50% MeCN/H_2_O); [α]_D_^25^ = +318.2 (*c* 1.0, MeOH); mp 168–172 °C (lyophilized from MeCN/H_2_O); *v̅*_max_ (thin film)/cm^–1^ 3358 (N–H, br, w), 3074 (O–H, br, w), 1702 (C=O, m), 1636 (N–H, m); ^1^H NMR (400 MHz, MeOD) δ_H_ 8.82 (1H, s), 8.01–7.98 (2H, m), 7.83 (1H, d, *J* 8.9), 7.65 (1H, dd, *J* 8.2, 2.2), 7.61–7.57 (3H, m), 7.42 (1H, d, *J* 8.2), 7.22 (1H, d, *J* 2.3), 7.07 (1H, dd, *J* 8.9, 2.3), 4.96–4.83 (1H, m) (partially hidden by H_2_O peak), 4.41 (1H, dd, *J* 12.2, 4.2), 2.75 (1H, ddd, *J* 12.5, 8.5, 4.2), 2.27 (3H, s), 1.44–1.36 (1H, m), 1.20 (3H, d, *J* 6.3); ^13^C NMR (151 MHz, MeOD) δ_C_ 172.2, 169.8, 151.7, 148.4, 146.4, 146.1, 139.9, 139.2, 137.7, 137.1, 131.4, 131.1, 128.0, 127.8, 126.9, 124.1, 123.8, 116.2, 103.4, 50.9, 49.6, 41.7, 23.0, 21.5; LRMS *m*/*z* (ESI^+^) 458 ([M + H]^+^, 42%); HRMS *m*/*z* (ESI^–^) [found: 456.1386, C_26_H_23_N_3_O_3_S, requires [M – H]^−^ 456.1387]; HPLC (Method 1), retention time = 8.2 min, 99.1%; Chiral HPLC, Chiral AD-H column (40:60 hexane:IPA, 1.0 mL min^–1^), retention time = 5.9 min (opposite enantiomer, 7.8% UV), retention time = 7.8 min (**14**, 92.2% UV); 84% e.e.

#### Ethyl 4-{(2*S*,4*R*)-1-Acetyl-2-methyl-4-[(quinolin-6-yl)amino]-1,2,3,4-tetrahydroquinolin-6-yl}benzoate (**22**)

Compound **S7a** (56 mg, 0.159 mmol, 1.2 equiv), 6-bromoquinoline (18 μL, 0.132 mmol, 1.0 equiv), CyJohnPhos (5 mg, 0.0143 mmol, 0.11 equiv), Pd(OAc)_2_ (2 mg, 0.00891 mmol, 0.067 equiv), and NaO^*t*^Bu (15 mg, 0.156 mmol, 1.2 equiv) were added to a flame-dried microwave vial, and the vial was purged with Ar. Anhydrous, degassed toluene (0.32 mL) was added to the vial, and the resulting solution was stirred at 100 °C for 15 h. After this time, the solution was cooled to rt and filtered through Celite, eluting with toluene, Et_2_O, and EtOAc. The washings were partially concentrated *in vacuo* to remove the Et_2_O, diluted with EtOAc (10 mL), and washed with H_2_O (20 mL) and then HCl_(aq)_ (1 M, 20 mL). The aqueous components were extracted with EtOAc (3 × 20 mL). The aqueous components were then neutralized with NaHCO_3(aq)_ and further extracted with EtOAc (3 × 40 mL). The combined organic components were washed with brine, dried over Na_2_SO_4_, filtered, and concentrated *in vacuo*. The crude material was purified using silica gel flash column chromatography (0–80% EtOAc/petroleum ether, 1% NEt_3_) to give compound **22** as a yellow solid (19 mg, 30%): *R*_*f*_ 0.16 (60% EtOAc/petroleum ether, 1% NEt_3_); [α]_D_^25^ = +405.8 (*c* 1.0, CHCl_3_); mp 82–86 °C (from CHCl_3_); *v̅*_max_ (thin film)/cm^–1^ 3324 (N–H, m), 2981 (C–H, m), 1712 (C=O, s), 1626 (s), 1608 (s); ^1^H NMR (400 MHz, CD_3_CN) δ_H_ 8.53 (1H, dd, *J* 4.2, 1.7), 7.98–7.92 (2H, m), 7.89 (1H, dd, *J* 8.4, 1.7), 7.84 (1H, d, *J* 9.1), 7.65–7.57 (3H, m), 7.57–7.53 (1H, m), 7.39 (1H, d, *J* 8.2), 7.35 (1H, dd, *J* 9.1, 2.6), 7.24 (1H, dd, *J* 8.4, 4.2), 6.85 (1H, d, *J* 2.6), 5.26 (1H, d, *J* 7.9), 4.90–4.76 (1H, m), 4.49 (1H, ddd, *J* 12.1, 7.9, 4.1), 4.30 (2H, q, *J* 7.1), 2.74 (1H, ddd, *J* 12.5, 8.5, 4.1), 2.19 (3H, s), 1.41–1.29 (1H, m), 1.32 (3H, t, *J* 7.1), 1.16 (3H, d, *J* 6.3); ^13^C NMR (151 MHz, CD_3_CN) δ_C_ 170.1, 166.9, 147.1, 147.0, 145.6, 144.1, 139.1, 138.3, 137.6, 134.5, 131.15, 131.12, 130.8, 130.4, 127.9, 127.7, 126.6, 123.2, 122.5, 122.4, 104.5, 61.8, 50.5, 48.6, 41.4, 23.4, 21.7, 14.6; LRMS *m*/*z* (ESI^+^) 480 ([M+H^+^]^+^, 100%); HRMS *m*/*z* (ESI^+^) [found: 480.2278, requires [M + H]^+^ 480.2282)]; HPLC (Method 1), retention time = 8.6 min, 95.8%; Chiral HPLC, Chiral IG-3 column (40:60 hexane:IPA, 1.0 mL min^–1^), retention time = 9.8 min (**22**, 93.1% UV), retention time = 18.4 (opposite enantiomer, 6.9% UV); 86% e.e.

#### 4-{(2*S*,4*R*)-1-Acetyl-2-methyl-4-[(quinolin-6-yl)amino]-1,2,3,4-tetrahydroquinolin-6-yl}benzoic Acid (**15**)

Compound **22** (51 mg, 0.106 mmol, 1 equiv) was dissolved in EtOH (0.66 mL), NaOH_(aq)_ (2 M, 0.53 mL, 1.06 mmol, 10 equiv) was added dropwise, and the resulting solution was left to stir for 2.5 h. After this time, the solution was acidified to approximately pH 5 using HCl_(aq)_ (1 M), and the aqueous solution was extracted with EtOAc (5 × 10 mL). The combined organic components were dried over Na_2_SO_4_, filtered, and concentrated *in vacuo*. The crude material was purified using silica gel flash column chromatography (reverse phase, 5–36% MeCN/H_2_O) and lyophilized to give **15** as a yellow-green solid (17 mg, 36%, 94.7% purity by HPLC). To achieve a higher purity, **15** was further purified by semipreparative HPLC and lyophilized (>99.9% by HPLC). *R*_*f*_ 0.38 (50% MeCN/H_2_O; reverse phase); [α]_D_^25^ = +369.7 (*c* 0.12, MeOH); mp 194–197 °C (from MeOH); *v̅*_max_ (thin film)/cm^–1^ 3342 (N–H, br, w), 2970 (O–H, br, w), 1703 (C=O, m), 1625 (N–H, s); ^1^H NMR (600 MHz, MeOD) δ_H_ 8.47 (1H, dd, *J* 4.3, 1.6), 8.07–8.02 (1H, m), 7.98 (2H, d, *J* 8.1), 7.83 (1H, d, *J* 9.1), 7.66 (1H, dd, *J* 8.2, 2.2), 7.60–7.58 (1H, m), 7.57 (2H, d, *J* 8.1), 7.47–7.40 (2H, m), 7.32 (1H, dd, *J* 8.3, 4.3), 6.86 (1H, d, *J* 2.6), 4.97–4.87 (1H, m), 4.49 (1H, dd, *J* 12.1, 4.2), 2.79 (1H, ddd, *J* 12.5, 8.5, 4.2), 2.29 (3H, s), 1.49–1.39 (1H, m), 1.22 (3H, d, *J* 6.3); ^13^C NMR (151 MHz, MeOD) δ_C_ 172.2, 169.7, 148.3, 146.0, 145.5, 142.2, 139.4, 139.2, 137.7, 137.1, 132.2, 131.4, 131.0, 128.9, 128.1, 127.8, 127.0, 123.8, 123.7, 122.7, 104.2, 50.6, 49.6, 41.6, 23.1, 21.5; LRMS *m*/*z* 452 (ESI^+^) ([M + H]^+^, 100%); HRMS *m*/*z* (ESI^+^) [Found: 452.1972, C_28_H_26_N_3_O_3_, requires [M + H]^+^ 452.1969]; HPLC (Method 1), retention time = 6.8 min, > 99.9%; Chiral HPLC, Chiral IG-3 column (70:30 hexane:IPA, 1.0 mL min^–1^), retention time = 17.2 min (**15**, 91.3% UV), retention time = 23.9 (opposite enantiomer, 8.7% UV); 83% e.e.

## Biological Methods

### Identification of *Sm*BCPs

The identification of *S. mansoni* BCPs (stably found in both v7 and v10 genome assemblies) was performed using a bioinformatic approach previously described.^13^ Briefly, a BLASTp search was performed to identify *S. mansoni* proteins (Smps) sharing high similarity (*E* value ≤1 × 10^–5^) to representative *H. sapiens* BCPs derived from literature data.^16,52−55^ The results of this method were validated and further expanded using a WormBase ParaSite^56^-based BioMart search looking for *S. mansoni* proteins containing annotated Bromo domain (BRD) or BRD associated domains. Interpro, Pfam, PROSITE, and SMART signatures were used for this search.

### Classification of *Sm*BCP Domains

The amino acid positions of the catalytic domain of each *S. mansoni* BCP (Bromo domain - BRD) were extracted from WormBase ParaSite. The architecture of these proteins was then populated with other N-terminal and/or C-terminal domains known from the literature to be associated with the catalytic domain. These additional domains (Figure 1B) were defined as follows: DNA binding homeobox and Different Transcription factors (DDT), Plant homeodomain (PHD), N-terminal Extra Terminal (ET) domain (NET), Transcription adaptor putative zinc finger (TAZ), KID-interacting domain (KIX), CREB-binding protein (CREBBP), ZZ-type zinc finger domain (ZZ), Histone acetyltransferase (HAT), Trp-Asp motif (WD40), Domain of unknown function 3512 (DUF3512), AAA- ATPase - ATPases Associated with various cellular Activities (AAA), Enhanced and polycomb-like (EPL), Pro-Trp-Trp-Pro domain (PWWP), Zinc finger MYND-type (MYND), Transcription initiation factor TFIID subunit 1 (TFIID), Bromo-adjacent homology domain (BAH), High mobility group box (HMG Box), Gln-Leu-Gln (Glutamine-Leucine-Glutamine) domain (QLQ), SNF2-related, N-terminal domain (SNF2-N), Helicase (Hel), Brahma and kismet domain (BRK), Helicase/SANT-associated domain (HAS), B-box-type zinc finger domain (B-BOX), and Forkhead-associated (FHA) domain (FHA). Graphical representation was prepared using Illustrator for Biological Sequences.^57^

### Gene Expression Profiling of *Sm*BCPs

The normalized gene expression values for each *smbcp* were derived by the RNA-Seq meta database curated by Lu et al.^58^ and accessible through the ‘schisto_xyz’ search engine (http://schisto.xyz/). This database was obtained by normalizing gene expression values derived RNA-Seq data published by Anderson et al. for egg,^59^ Wang et al. for miracidia (indicated as Mir in the heat map) and sporocysts (Spo),^60^ Protasio et al. for cercariae (Cerc), 3h and 24h schistosomula (Som_3h and Som_24h),^61^ Protasio et al. for 21 day juvenile male and female (Male_21days and Female_21days),^62^ and Lu et al. for 42 day adult male and female (Male_42days and Female_42days).^63^ The gene expression values for the 22 BCPs were extracted for each of the 10 life cycle stages and plotted as a heat map in R studio.

### Ethics Statement

All mouse procedures performed at Aberystwyth University adhered to the United Kingdom Home Office Animals (Scientific Procedures) Act of 1986 (project license P3B8C46FD) as well as the European Union Animals Directive 2010/63/EU and were approved by Aberystwyth University’s Animal Welfare and Ethical Review Body (AWERB).

### *Ex Vivo* Schistosomula Screening

*S. mansoni* schistosomula were obtained by mechanical transformation from cercariae as previously described,^64^ distributed in 384-well tissue culture plates (PerkinElmer, catalogue number 6007460) at 120 parasites/well and dosed with the selected compounds as previously reported.^65−68^ For this study, each compound (as 10 mM DMSO stock solution) was initially tested at two concentration points (20 and 10 μM final concentration, in duplicates) in at least four independent screens (*Z*′ scores above 0.35).^69^ Each screen contained both positive (10 μM auranofin (AUR) in 0.625% DMSO) and negative (0.625% DMSO) controls. For those compounds active at 10 μM (18, 20, 21, and 22), a further 2-fold titration (from 10 to 0.312 μM) was performed to determine the extent of their antischistosomula activity (Figure S48). Following 72 h schistosomula/compound coincubation, the plate was analyzed by the Roboworm platform.^70^ EC_50_ values were calculated from the titrated concentrations by nonlinear regression, after log transformation of concentrations and data normalization using GraphPad Prism 7.02.

### *Ex Vivo* Adult Worm Screening

*S. mansoni* adult worms were recovered by hepatic portal vein perfusion from TO mice (*Mus musculus* HsdOLa:TO - Tuck Ordinary; Envigo, UK) that were percutaneously infected 7 weeks earlier with 180 cercariae.^65,71^ Following perfusion, schistosomes were washed and processed as previously described.^65,66^ Subsequently, schistosome pairs were seeded into 48 well tissue culture plates (1 worm pair/well, in duplicate) and dosed with the compounds (20 μM in 0.2% DMSO). DMSO (0.2%) and praziquantel (10 μM in 0.1% DMSO) were also included as negative and positive controls, respectively. Parasite motility was assessed by a digital image processing-based system (WormassayGP2),^66^ modified after Wormassay.^72^*In vitro* laid eggs (IVLEs) were enumerated and the presence or absence of paired worms was noted.

### *Ex Vivo* Miracidia Screening

*S. mansoni* miracidia were obtained by hatching of schistosome eggs as previously described.^73^ The resulting miracidial suspension was collected, washed with Chernin’s balanced salt solution (CBSS),^73^ and enumerated prior to being used for *ex vivo* miracidia to sporocyst screens as previously reported.^66,73^ Each compound was tested at 0.5, 2.0, 5.0, 10, and 25 μM final concentrations (in 1% DMSO). Each treatment was set up in duplicate (15–25 miracidia/well). Each titration was performed in three independent experiments (two technical replicates per data point). Parasites cultured in CBSS (containing 1% DMSO) were included as negative controls. After 48 h incubation at 26 °C in the dark, dead, fully transformed and partially transformed miracidia were enumerated as previously described.^74,75^

## Supporting Information Available

NMR spectra, HPLC traces for biologically tested compounds; raw data; Δ*T*_M_ data and ITC traces (PDF)
X-ray crystallography data under accession codes 7AMC and 7AMH (PDB)
Molecular formula strings (CSV)

## Supplementary Material

jm3c01321_si_001.pdf

jm3c01321_si_002.pdb

## Abbreviations

BCPs: bromodomain-containing proteins;
BRDs: bromodomains;
Cer: cercaria;
Δ*T*_m_: protein thermal shift;
*Hs*BRD3/4: human bromodomain containing protein 3/4;
ITC: isothermal titration calorimetry;
KAc: acetylated lysine;
Mir: miracidia;
MUSCLE: multiple sequence comparison by log-expectation;
PDB: protein data bank;
PZQ: praziquantel;
*Sm*BRD3: *Schistosoma mansoni* bromodomain containing protein 3;
*Sm*BRD3(1/2): the first/second bromodomain of *Sm*BRD3;
*Sm*CBP1/2: cyclic AMP response element binding protein binding protein1/2 from *S. mansoni*;
*Sm*GCN5: general control nondepressible 5 from *S. mansoni*;
Som: schistosomula;
*T. cruzi*: *Trypanosoma cruzi*
