# Supplementary material for: Small Molecule Ligands of the BET-like Bromodomain, SmBRD3, Affect Schistosoma mansoni Survival, Oviposition, and Development
Source: J Med Chem. 2023 Dec 4;66(23):15801–22. doi: 10.1021/acs.jmedchem.3c01321 (PMC10726355; doi:10.1021/acs.jmedchem.3c01321)
Supplement: Supplementary file 1 — jm3c01321_si_001.pdf [file jm3c01321_si_001.pdf]

Supporting Information

Small molecule ligands of the BET-like bromodomain, *Sm*BRD3, affect *Schistosoma mansoni* survival, oviposition, and development

Matthias Schiedel,[1,†] Darius J. B. McArdle,[1,†] Gilda Padalino,[2,†,‡] Anthony K. N. Chan,[1] Josephine Forde-Thomas,[2] Michael McDonough,[1] Helen Whiteland,[2] Manfred Beckmann,[2] Rosa Cookson,[3] Karl F. Hoffmann,[2,] Stuart J. Conway[1,4,*]*

[1]Department of Chemistry, Chemistry Research Laboratory, University of Oxford, Mansfield Road, Oxford OX1 3TA, UK

[2]The Institute of Biological, Environmental and Rural Sciences (IBERS), Aberystwyth University, SY23 3DA, Wales, UK

[3]GlaxoSmithKline R&D, Stevenage, Hertfordshire, SG1 2NY, UK

[4]Department of Chemistry & Biochemistry, University of California Los Angeles, 607 Charles E. Young Drive East, P. O. Box 951569, Los Angeles, California, 90095-1569, USA

*To whom correspondence should be addressed:

krh@aber.ac.uk and stuartconway@chem.ucla.edu

Table of contents

Content	Pages
Supplementary figures, tables, and schemes	S3–S52
General biology experimental and biology methods	S53–S64
NMR spectra of novel compounds	S65–S94
HPLC traces of biologically tested compounds	S95–S112
References	S113–S114

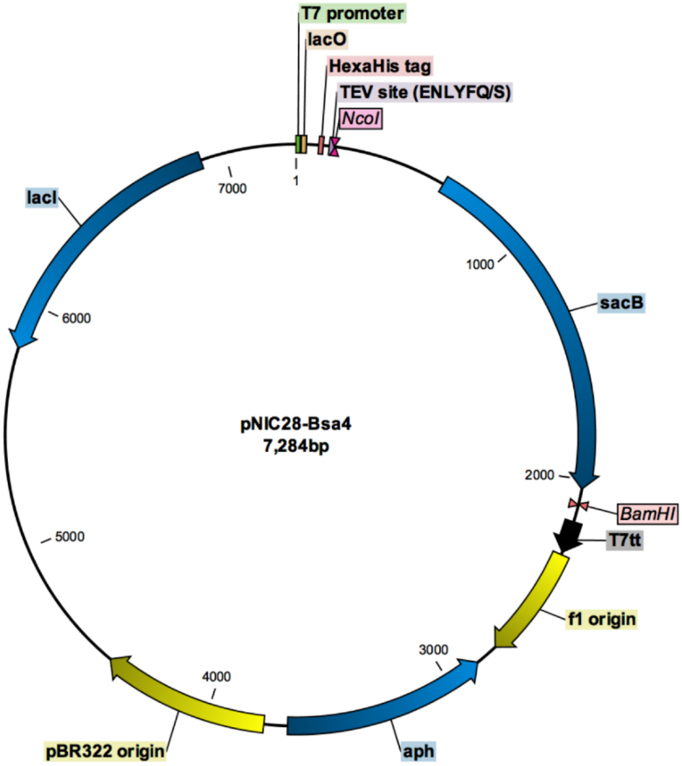

Figure S-1. A visual representation of the plasmid cloning and expression vector, pNIC28-Bsa4,[1] which is drawn to scale. All other expression plasmids used in this study share the same vector backbone. The vector contains in-frame hexa-histidine (His_6) codons, located directly after the initiation start (ATG), for protein purification by immobilized metal affinity chromatography. The T7 promoter is used for transcribing target gene, and T7tt is the T7 transcriptional terminator for terminating gene transcription. The *Nco*I and *Bam*HI restriction sites for linearizing the vector for Gibson assembly 2 are shown. The *sac*B gene allows negative selection of plasmid without gene insert on 5% sucrose. Protein expression can be initiated by addition of IPTG (isopropyl β-D-1-thiogalactopyranoside), which removes transcriptional repression exerted by the LacI repressor (encoded by the constitutively expressed *lacI* gene). The locations of the *lacO* (lac operator, the site bound by the LacI repressor) and TEV site (protease cleavage site for removal of the His_6 tag) are indicated. DNA replication origins, pBR322 origin (for double-stranded DNA replication of plasmid in *E. coli*) and f1 (for single stranded replication in presence of a helper phage), are shown. The kanamycin selectable marker gene, *aph*, is labelled, which encodes aminoglycoside 3'-phosphotransferase. The plasmid map was generated and annotated using CLC Main Workbench 7.6.4.

Table S-1. Primers for the production of H6-SmBRD3(9..378aa) and H6-SmBRD3(241..368aa):

Primer	Direction	Sequence: (5' to 3')	Tm (°C)
GA_f	Forward	AGAACCTGTACTTCCAATCC	61
GA_r	Reverse	CGGAGCTCGAATTCG	60
SmBRD3(2)_f1	Forward	AGAACCTGTACTTCCAATCCCCGAAACGTGAATATGAAGAAC	61
SmBRD3(2)_r1	Reverse	CGGAGCTCGAATTCGGATCCTTATCAGCTTTCATCATCCGGC	61

Cloning of pSmBRD3(9..378aa):

Vector: pNIC28-Bsa4 (7,283 bp) → *NcoI*-HF + *BamHI*-HF (CutSmart) → linearized vector (5,337 bp)

Synthetic fragment: SmBRD3_9_to_378aa (1,156 bp)

PCR: SmBRD3_9_to_378aa (1,156 bp)+ GA_f + GA_r → p2 (1,156 bp)

Gibson assembly: Linearized vector + p2 → **pSmBRD3(9..378aa)** (6,455 bp)

Sequencing primer(s): T7F or T7R

Remark: The synthetic gene (i.e SmBRD3_9_to_378aa with codons optimized for protein expression in *E. coli*) encodes the SmBRD3(9..378aa) fragment (GenBank ID: CCD76183.1).

>H6-SmBRD3(9..378aa)

MHHHHHHSSGVDLGTENLYFQSSEANLDSSNSIKSEPHAAKSHSSKITTNQLEYIKKEVVGRLFKE
KIVWPFTKPVDHQRLNLPDYPKIIKHPMDLGTIKQRLNLKFYHSSSECLDDLFTMFRNCYIFNKPGD
DVVAMAMKLEQIARERLKFMPTPETEICPQKTPKSIRPIGAPLQVHPPIEPIHTAASTNHTEGLNGS
AVSVDQTTLPFRPSVTSTSTKKASKKKSDSTIDELPSTPQSYDDLSRDRRQIKKPKREYEERNVGK
RLRLSEALKACSNILKDISSQRYRDLNHFFLKPVDVVALGLHDYYDVVKKAMDLSTIKTKLESGQYH
TKYDFADDVRLMFNNCYKYNGEDSEVARVGKQLQAIFDENFAKVPDDESDPAASPDGRP

Cloning of pSmBRD3(241..368aa):

Vector: pNIC28-Bsa4 (7,283 bp) *NcoI*-HF + *Bam*HI-HF (CutSmart) → linearized vector (5,337 bp)

PCR: pSmBRD3(9..378aa) (6,455 bp) + SmBRD3(2)_f1 + SmBRD3(2)_r1 → p24 (430 bp)

Gibson assembly: Linearized vector + p24 → **pSmBRD3(241..368aa)** (5,729 bp)

Sequencing primer(s): T7F or T7R

Remark: Native SmBRD3 (GenBank ID: CCD76183.1).

>H6-SmBRD3(241..368aa)

MHHHHHHSSGVDLGTENLYFQSPKREYEERNVGKRLRLSEALKACSNILKDISSQRYRDLNHFFL
KPVDVVALGLHDYYDVVKKAMDLSTIKTKLESGQYHTKYDFADDVRLMFNNCYKYNGEDSEVARV
GKQLQAIFDENFAKVPDDES

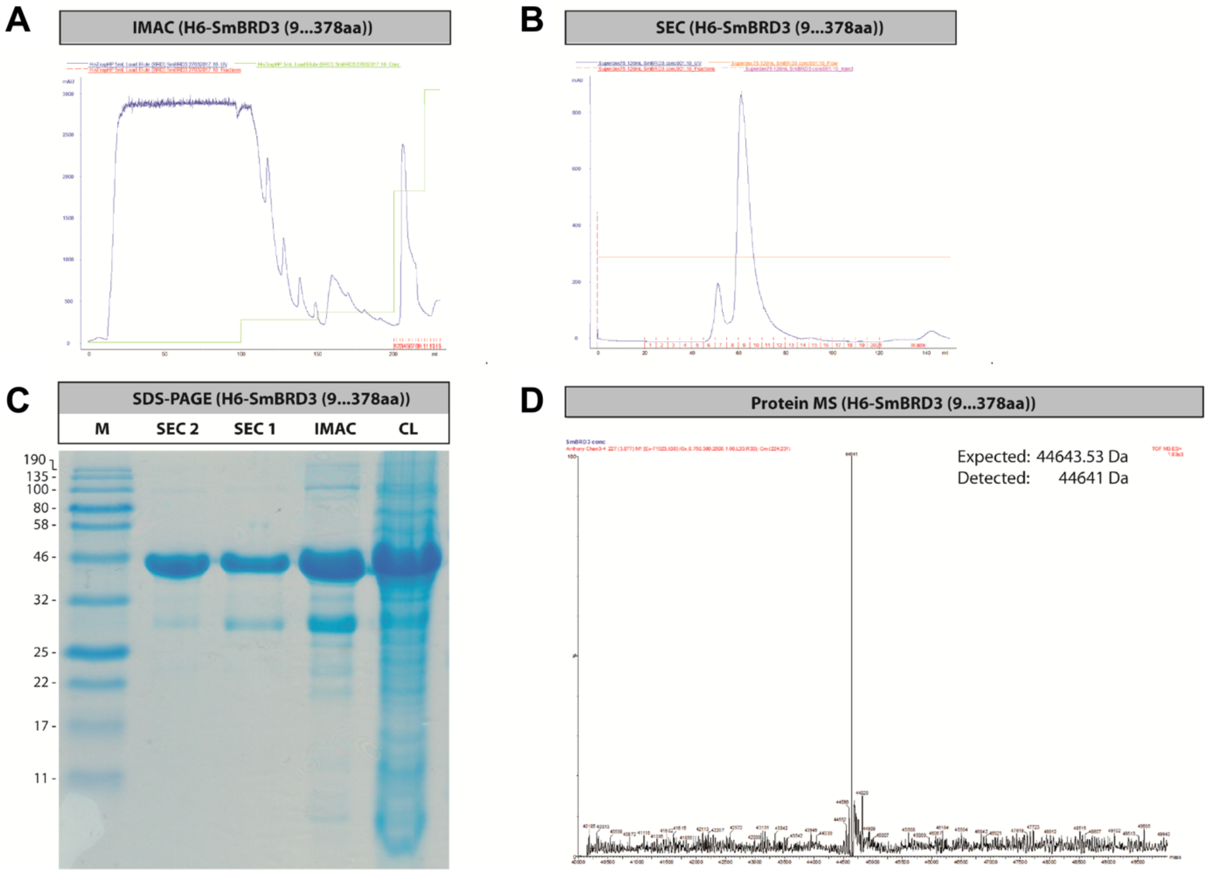

Figure S-2. Purification of H6-*Sm*BRD3(1,2) (9..378aa). **A.** IMAC profile; **B.** SEC profile. **C.** SDS-PAGE gel (M = marker, SEC1/2 = different fractions from size exclusion chromatography, IMAC = eluate of immobilized metal ion affinity chromatography, CL = cell lysate); **D**. Protein mass spectrum.

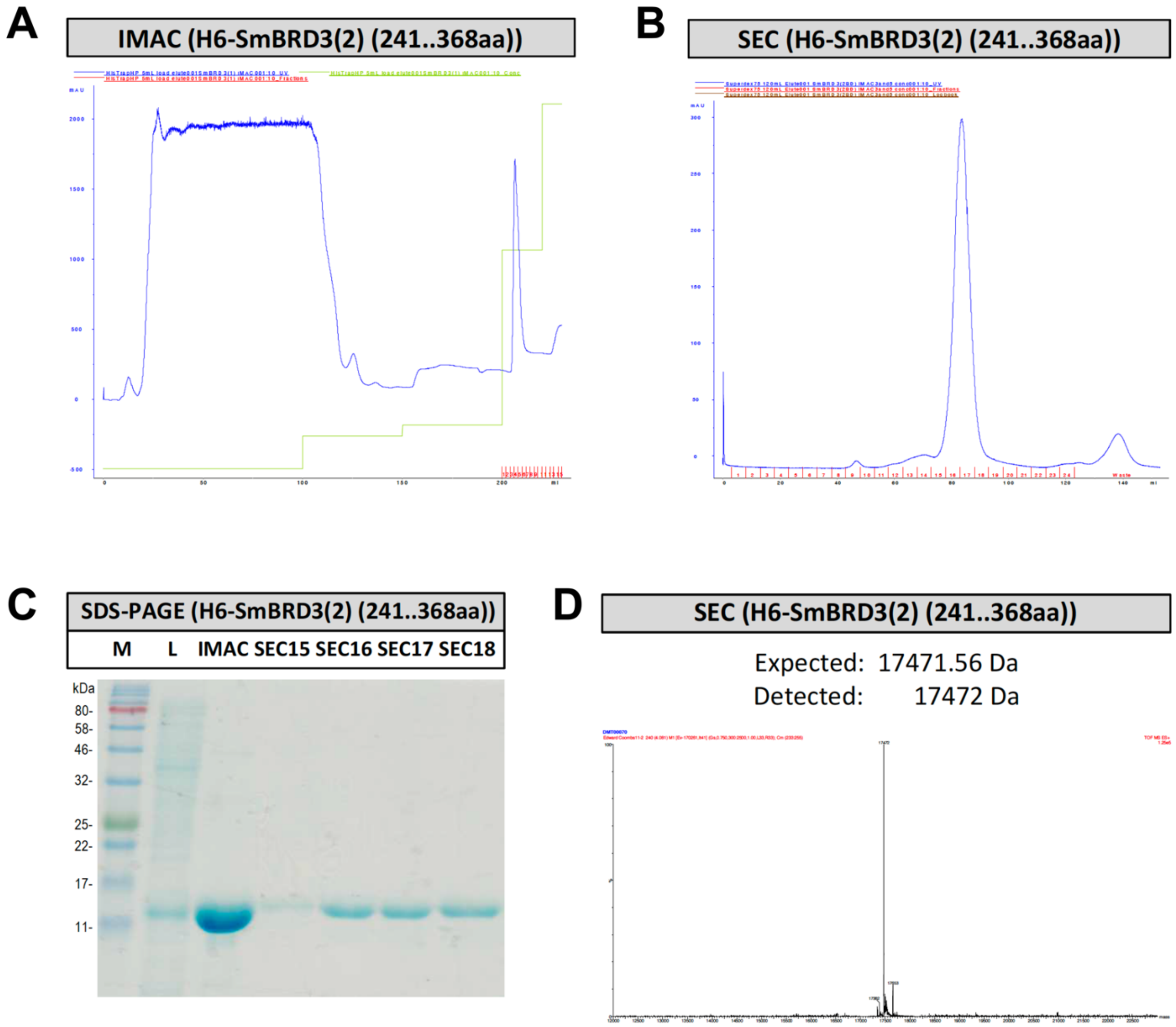

Figure S-3. Purification of H6-*Sm*BRD3(2) (241..368aa). **A.** IMAC profile; **B.** SEC profile; **C.** SDS-PAGE gel (M = marker, L = lysate, IMAC = collected eluate from immobilized metal ion affinity chromatography, SEC = collected eluate from size exclusion chromatography); **D.** Protein mass spectrum.

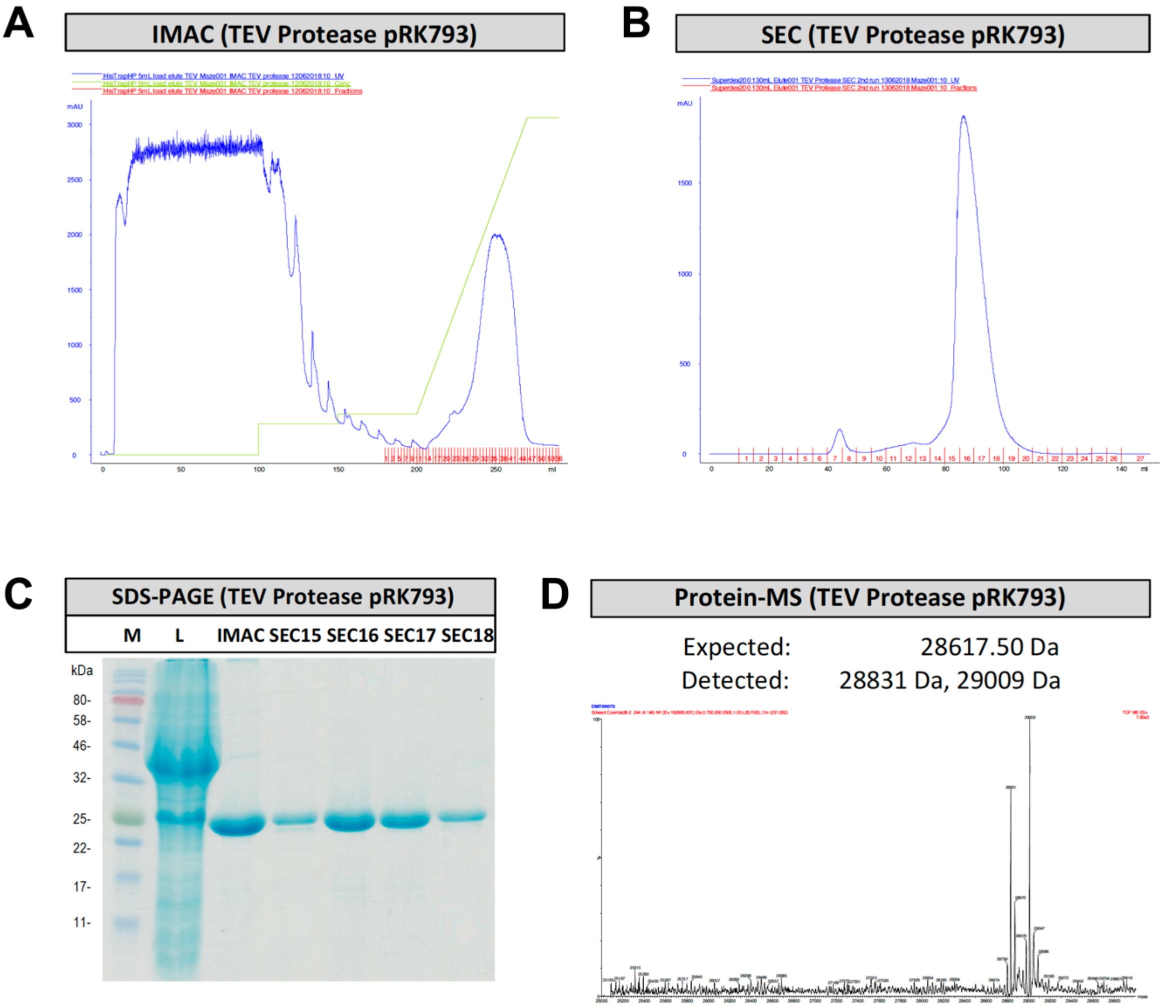

Figure S-4. Purification of TEV Protease (pRK793). **A.** IMAC profile; **B.** SEC profile; **C.** SDS-PAGE gel (M = marker, L = lysate, IMAC = collected eluate from immobilized metal ion affinity chromatography, SEC = collected eluate from size exclusion chromatography); **D.** Protein mass spectrometry.

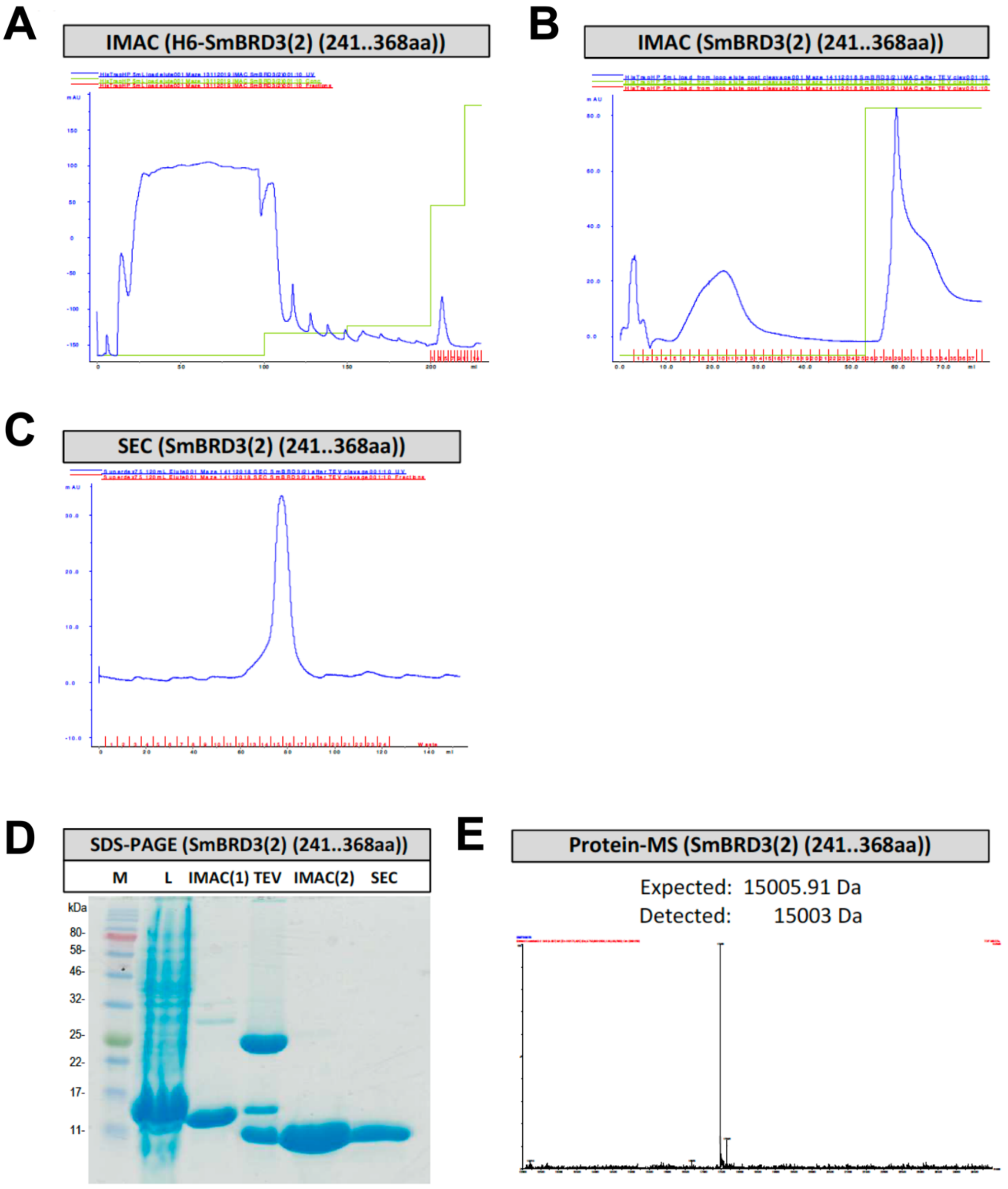

Figure S-5. Purification of *Sm*BRD3(2) (241..368aa). **A.** IMAC profile before TEV cleavage; **B.** IMAC profile after TEV cleavage; **C.** SEC profile; **D.** SDS-PAGE gel (M = marker, L = lysate, IMAC(1) = collected eluate from immobilized metal ion affinity chromatography prior to TEV cleavage, TEV = incubation with TEV protease (pRK793, Mr = 28617.5 Da), substrate/TEV ratio = 2:1, 4 °C, 16 h), IMAC(2) = collected eluate of immobilized metal ion affinity chromatography after TEV cleavage, SEC = collected eluate of size exclusion chromatography); **E.** Protein mass spectrum.

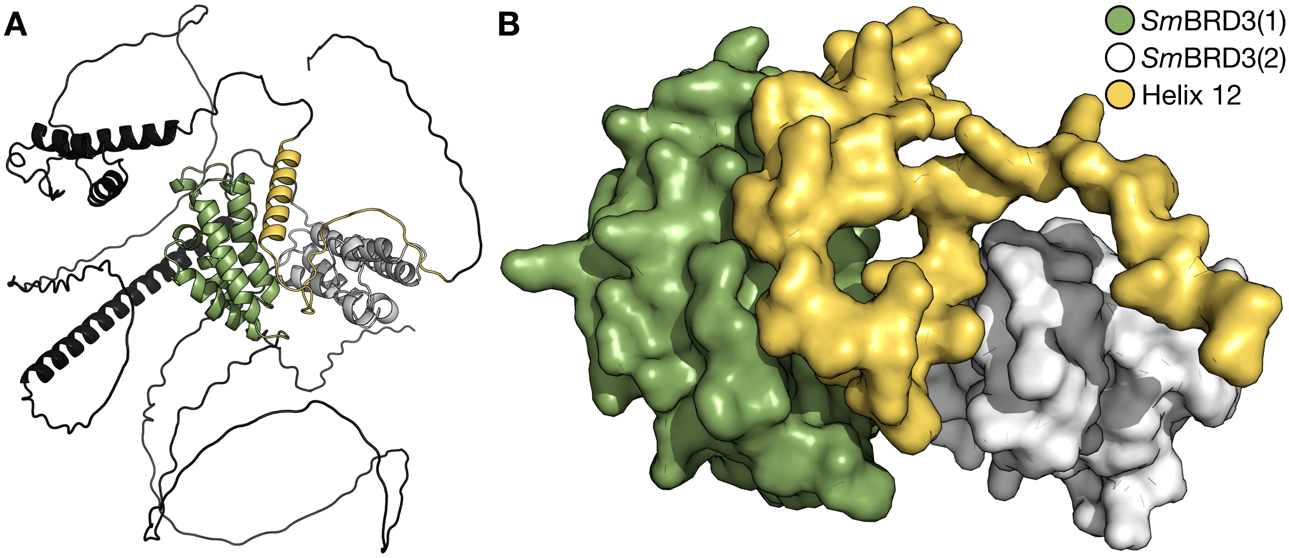

Figure S-6.A. The structure of the full length *Sm*BRD3 protein (Uniprot ID: A0A5K4EQL3) predicted by AlphaFold.[2] Helix 12 (gold) is located between *Sm*BRD3(1) (green) and *Sm*BRD3(2). **B.** It is proposed that the absence of the hydrophobic intraprotein interactions between *Sm*BRD3(1) (green) and helix 12 (gold) are responsible for difficulties in identifying a stable construct for the individual *Sm*BRD3(1) bromodomain. We suggest that these hydrophobic interactions are compensated for in the *Sm*BRD3(1,2) construct. As *Sm*BRD3(2) (white) forms fewer hydrophobic interactions with helix 12 (gold) this bromodomain can be produced as a stable construct.

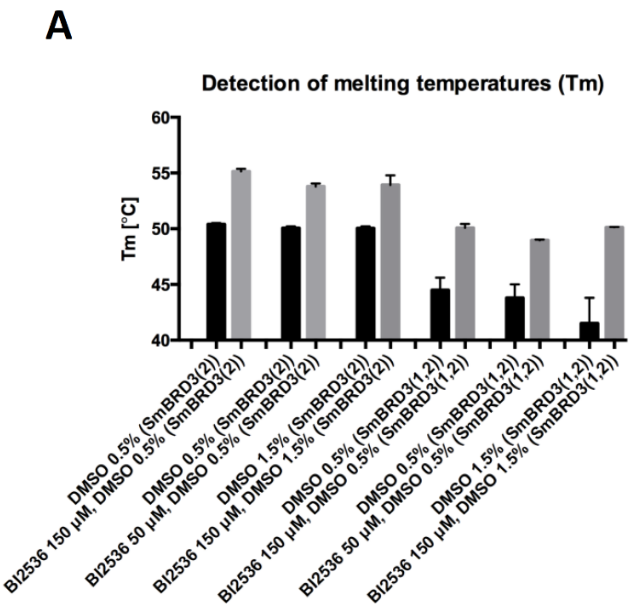

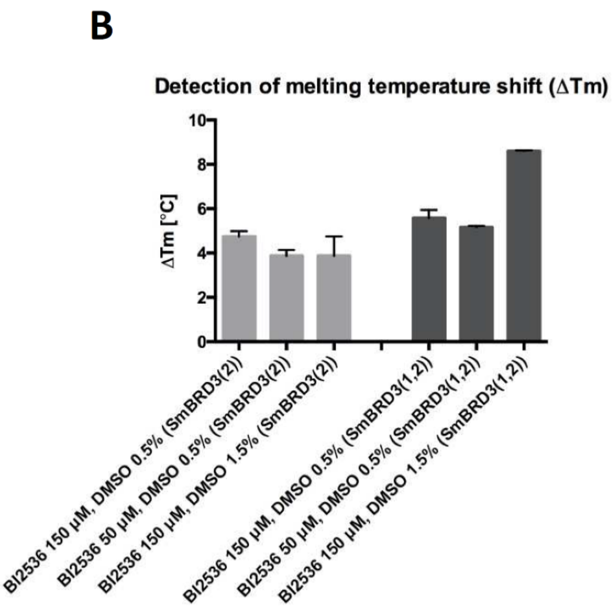

Figure S-7. The detection of binding of BI2536 (**7**) to *Sm*BRD3(2) and *Sm*BRD3(1,2) using a differential scanning fluorimetry (DSF) assay at concentrations of 50 µM or 150 µM, and DMSO at percentages of 0.5% or 1.5%. **A**. Detection of melting temperatures (Tm) of *Sm*BRD3(2) and *Sm*BRD3(1,2) in the presence of BI2536 (**7**) (50 µM or 150 µM, 0.5% or 1.5% DMSO) or DMSO (0.5% or 1.5%) (n=3). **B.** Thermal shifts of the melting temperatures (ΔT_m) of *Sm*BRD3(2) and *Sm*BRD3(1,2) in the presence of BI2536 (**7**) (50 µM or 150 µM, 0.5% or 1.5% DMSO) compared to DMSO (0.5% or 1.5%) (n=3).

Differential Scanning Fluorimetry (Thermal Shift) Screening Against *Sm*BRD3(2)

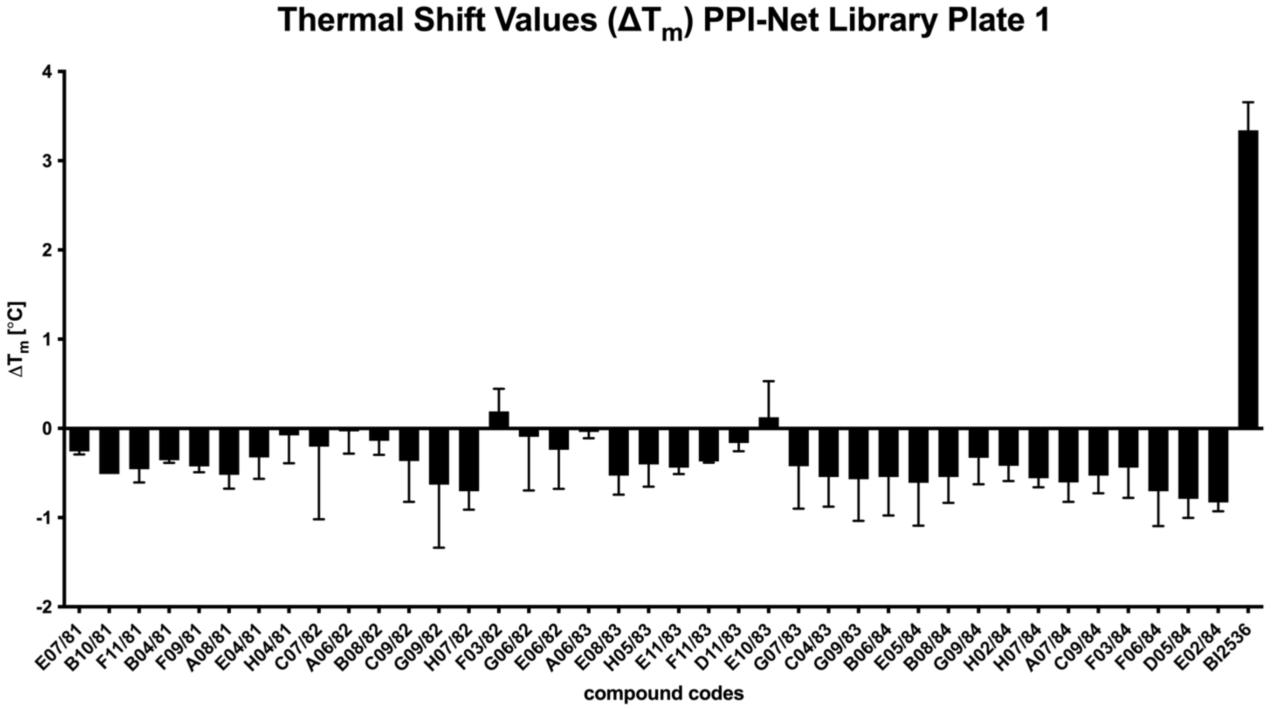

Figure S-8. Plate 1. Binding of compounds (50 µM) of the PPInet library to *Sm*BRD3(2) monitored using a differential scanning fluorimetry (DSF) assay (n=2). BI2536 (**7**, 50 µM) was used as a positive control. Mean values ± standard deviation are shown.

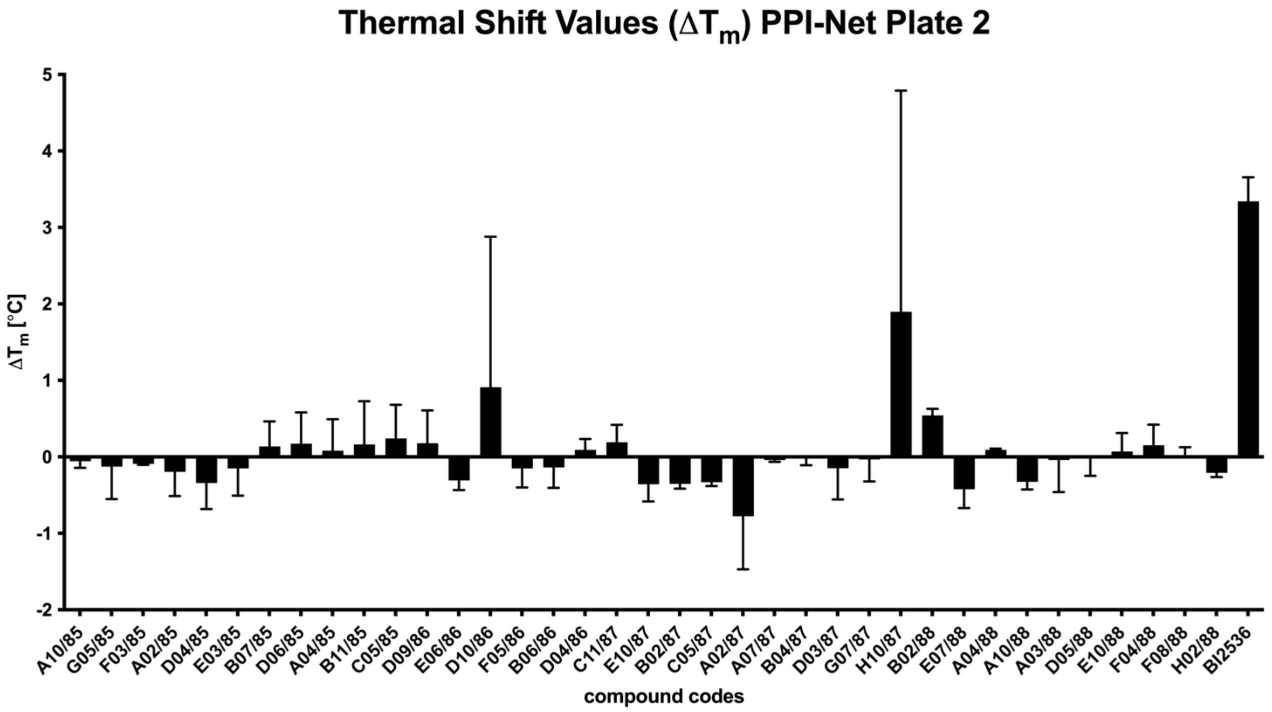

Figure S-9. Plate 2. Binding of compounds (50 µM) of the PPInet library to *Sm*BRD3(2) monitored using a differential scanning fluorimetry (DSF) assay (n=2). BI2536 (**7**, 50 µM) was used as a positive control. Mean values ± standard deviation are shown.

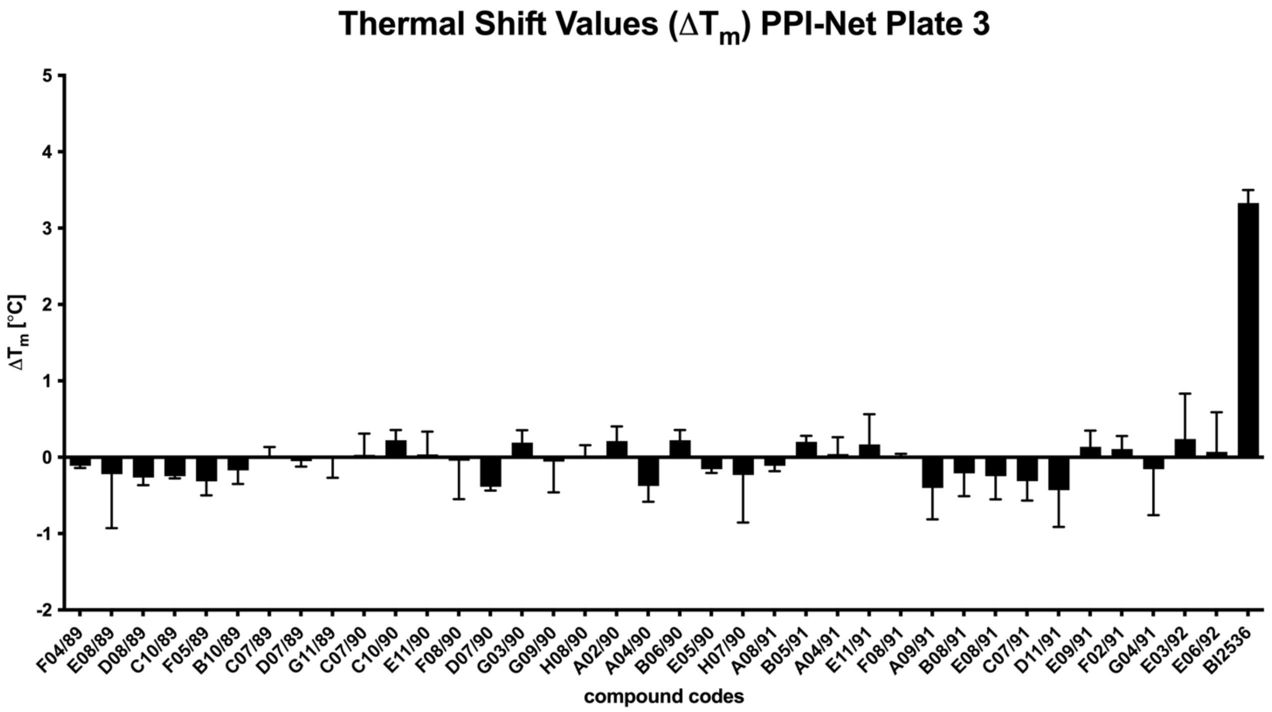

Figure S-10. Plate 3. Binding of compounds (50 µM) of the PPInet library to *Sm*BRD3(2) monitored using a differential scanning fluorimetry (DSF) assay (n=2). BI2536 (**7**, 50 µM) was used as a positive control. Mean values ± standard deviation are shown.

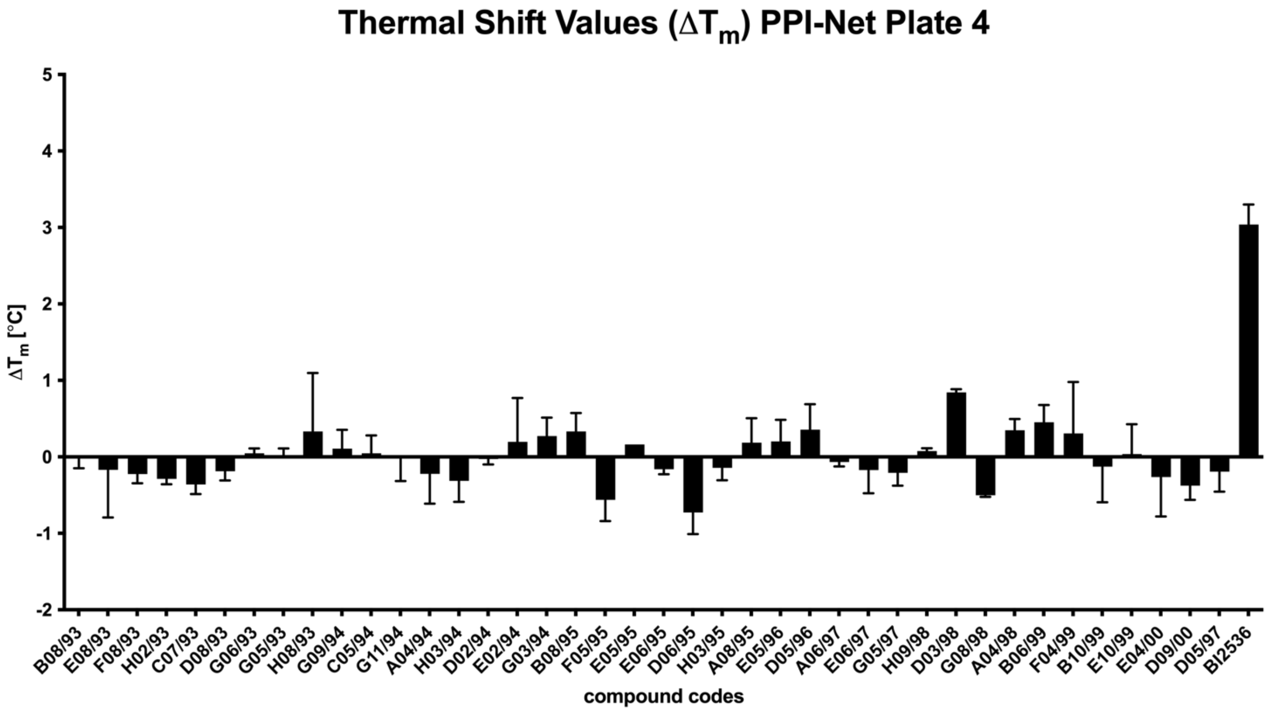

Figure S-11. Plate 4. Binding of compounds (50 µM) of the PPInet library to *Sm*BRD3(2) monitored using a differential scanning fluorimetry (DSF) assay (n=2). BI2536 (**7**, 50 µM) was used as a positive control. Mean values ± standard deviation are shown.

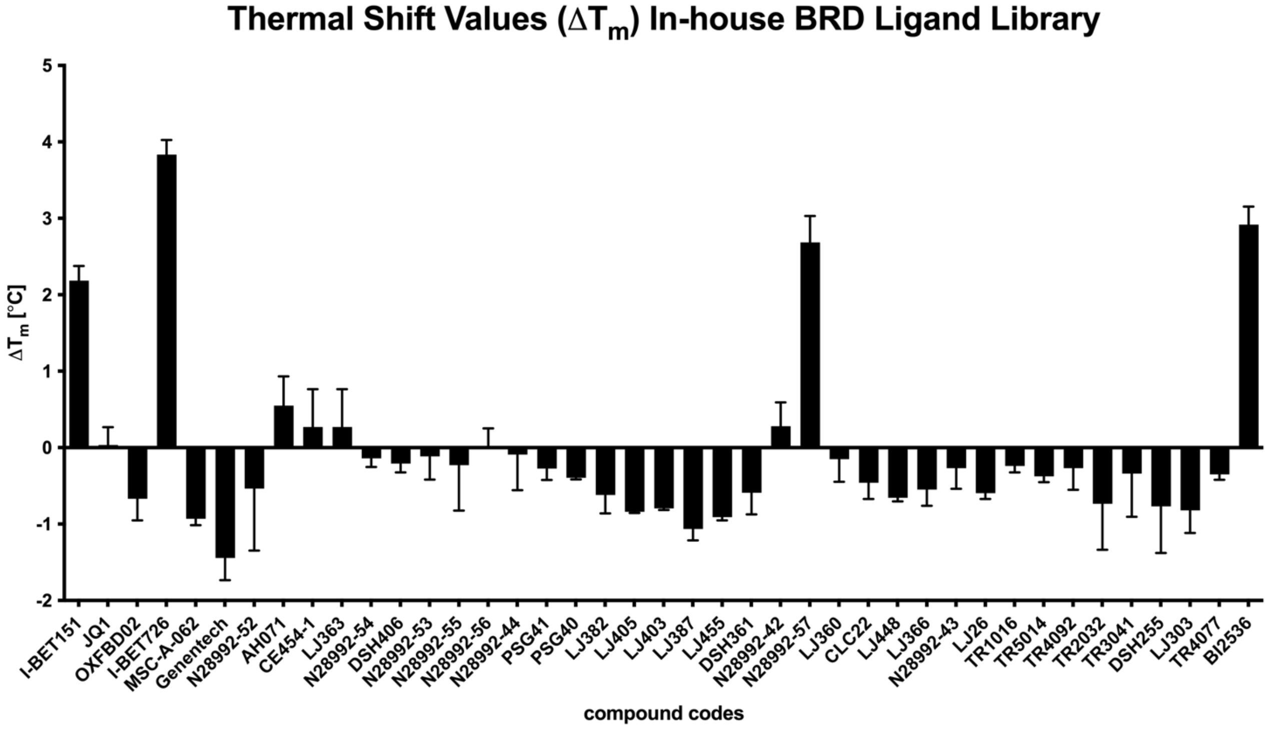

Figure S-12. Plate 5. Binding of compounds (50 µM) of our in-house library to *Sm*BRD3(2) monitored by means of a differential scanning fluorimetry (DSF) setup (n = 2). BI2536 (**7**, 50 µM) was used as a positive control. Mean values ± standard deviation are shown.

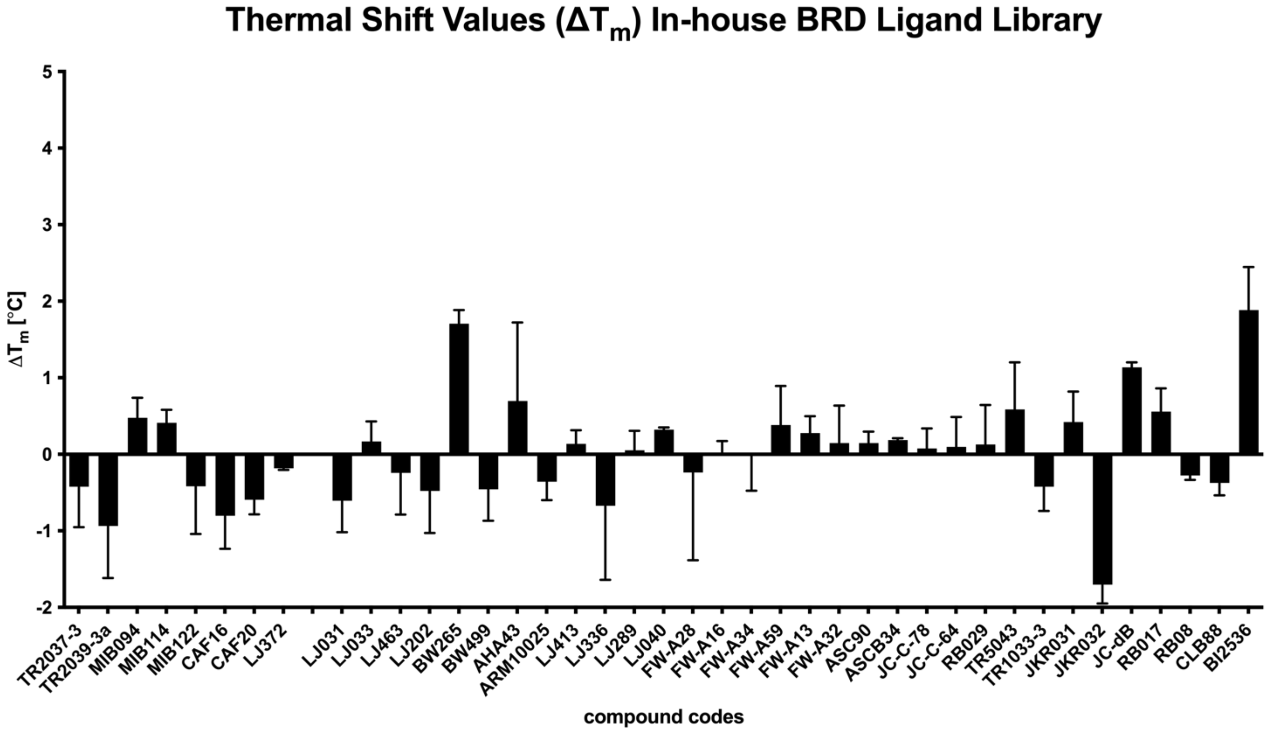

Figure S-13. Plate 6. Binding of compounds (50 µM) of our in-house library to *Sm*BRD3(2) monitored by means of a differential scanning fluorimetry (DSF) setup (n = 2). BI2536 (**7**, 50 µM) was used as a positive control. Mean values ± standard deviation are shown.

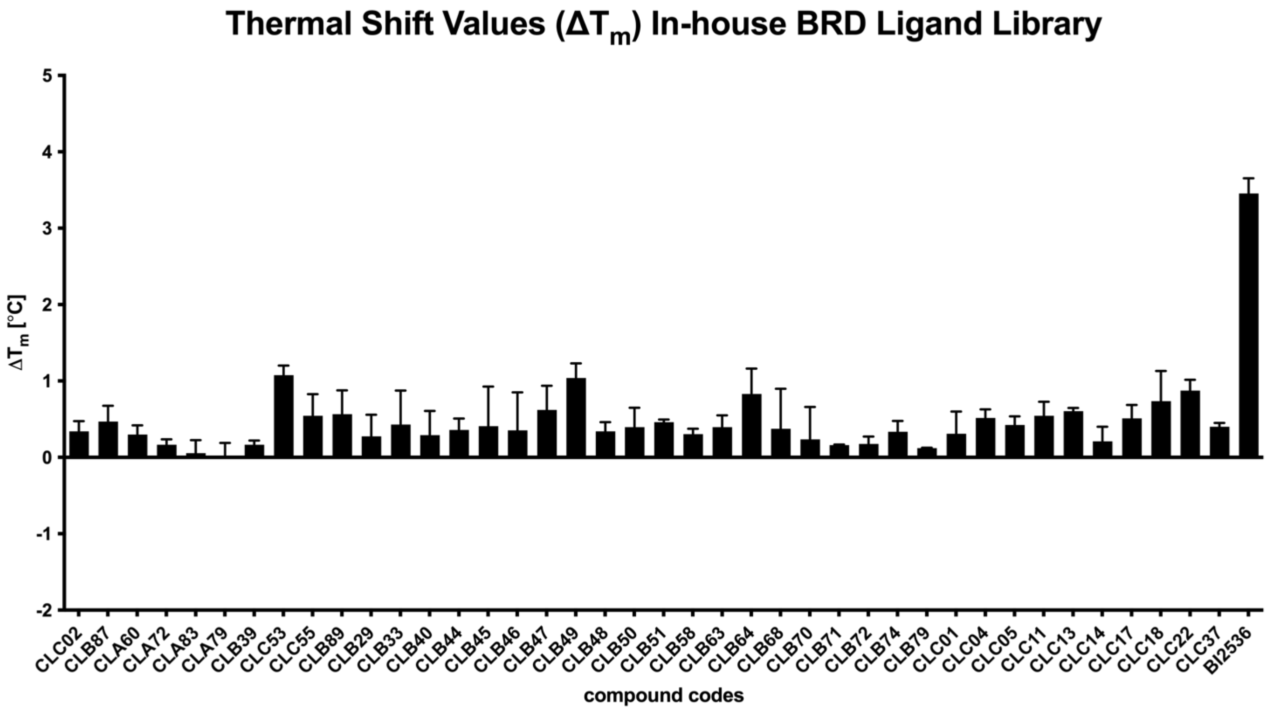

Figure S-14. Plate 7. Binding of compounds (50 µM) of our in-house library to *Sm*BRD3(2) monitored by means of a differential scanning fluorimetry (DSF) setup (n = 2). BI2536 (**7**, 50 µM) was used as a positive control. Mean values ± standard deviation are shown.

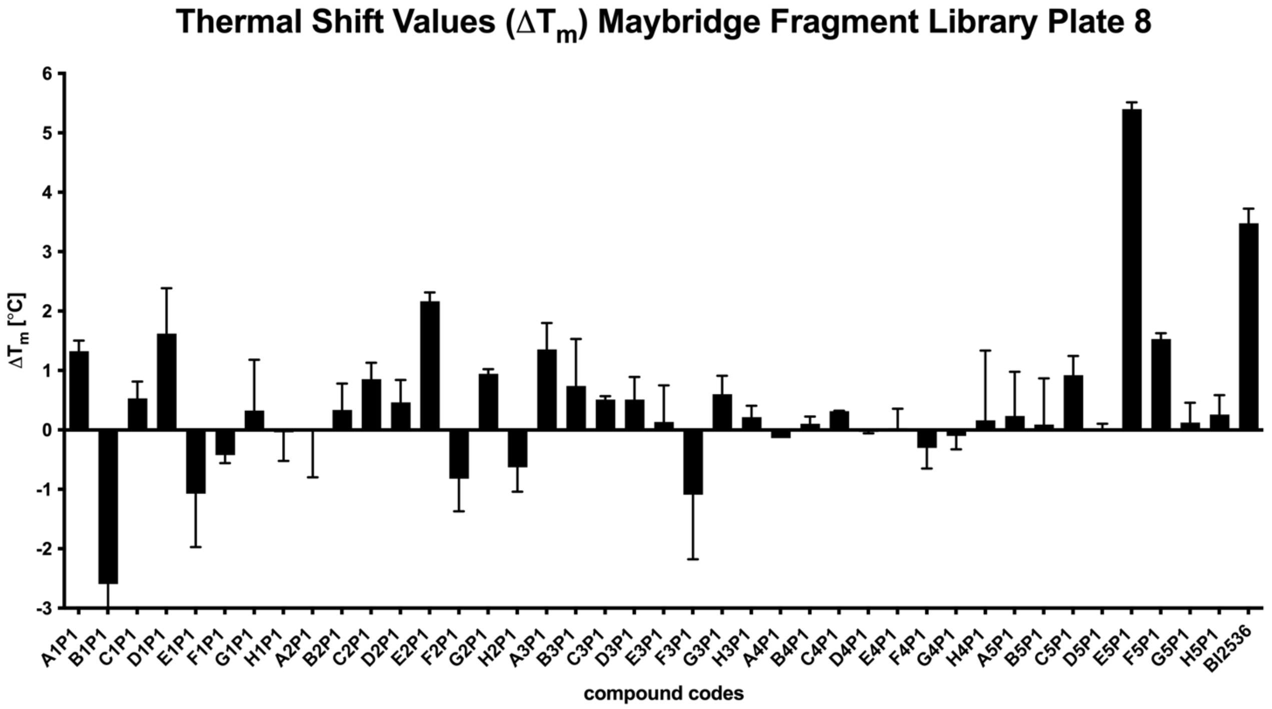

Figure S-15. Plate 8. Binding of compounds (150 µM) from the Maybridge Fragment Library to *Sm*BRD3(2) monitored by means of a differential scanning fluorimetry (DSF) setup (n = 2). BI2536 (**7**, 150 µM) was used as a positive control. Mean values ± standard deviation are shown.

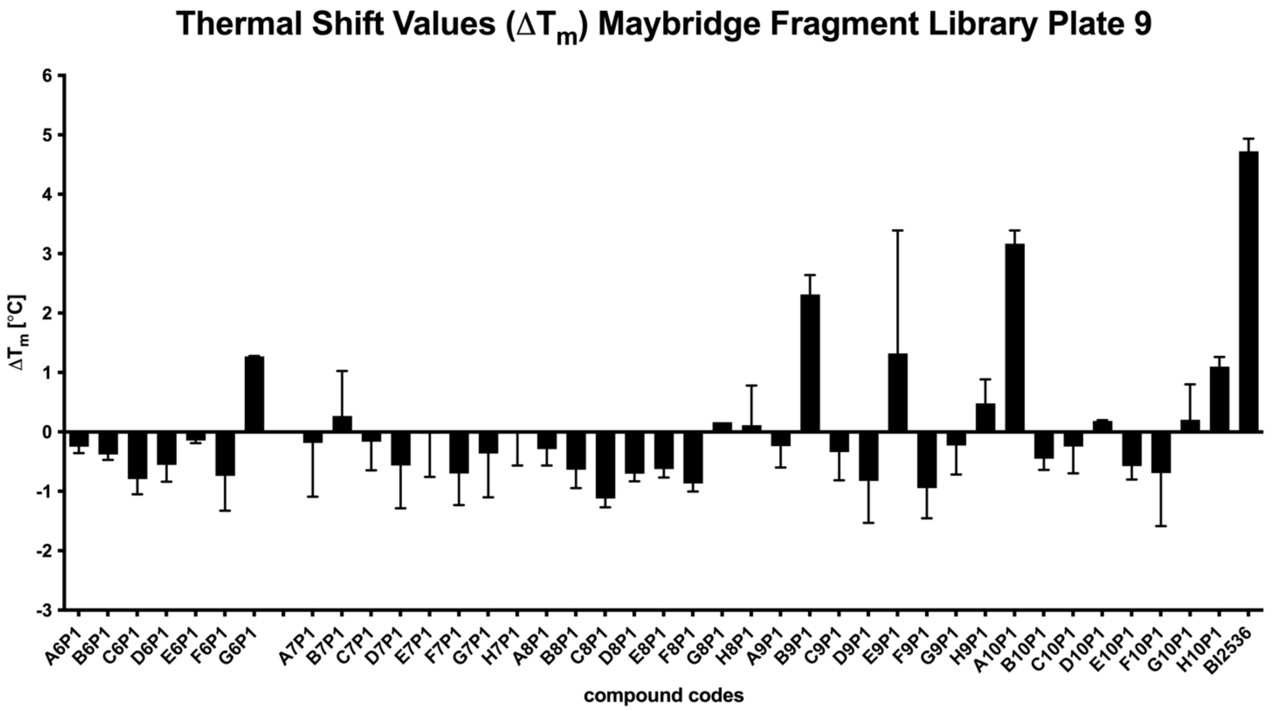

Figure S-16. Plate 9. Binding of compounds (150 µM) from the Maybridge Fragment Library to *Sm*BRD3(2) monitored by means of a differential scanning fluorimetry (DSF) setup (n = 2). BI2536 (**7**, 150 µM) was used as a positive control. Mean values ± standard deviation are shown.

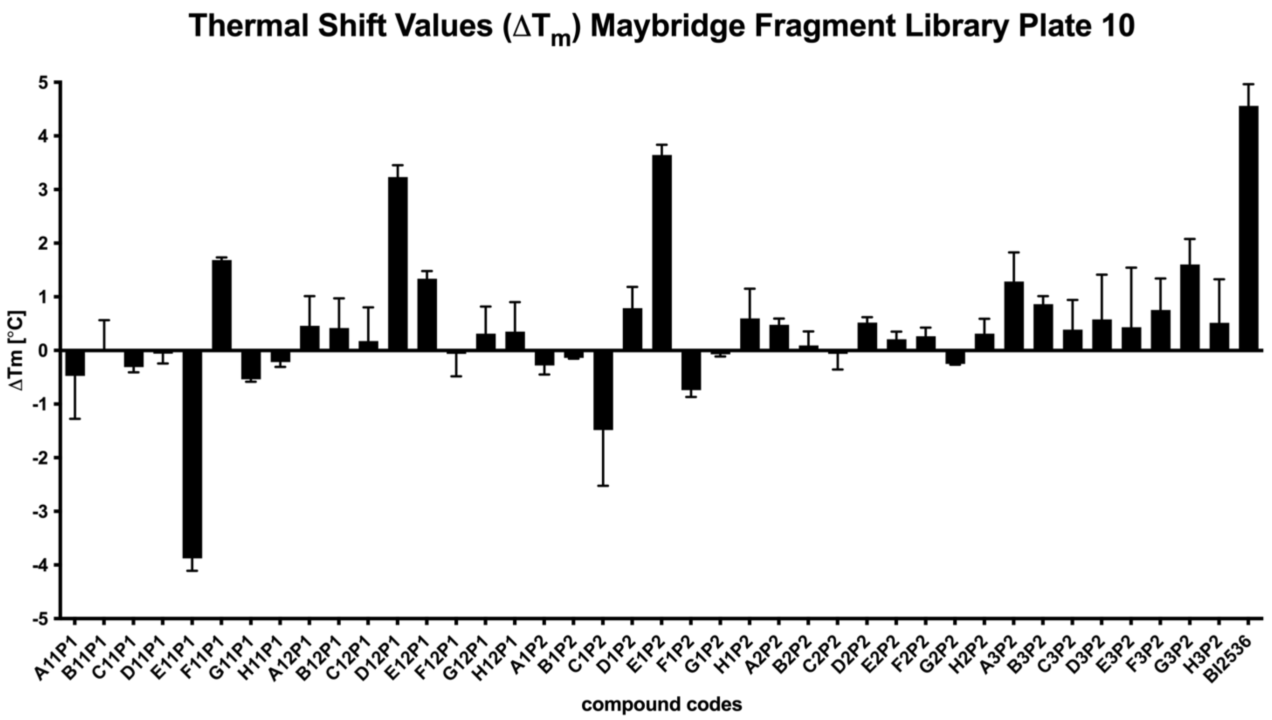

Figure S-17. Plate 10. Binding of compounds (150 µM) from the Maybridge Fragment Library to *Sm*BRD3(2) monitored by means of a differential scanning fluorimetry (DSF) setup (n = 2). BI2536 (**7**, 150 µM) was used as a positive control. Mean values ± standard deviation are shown.

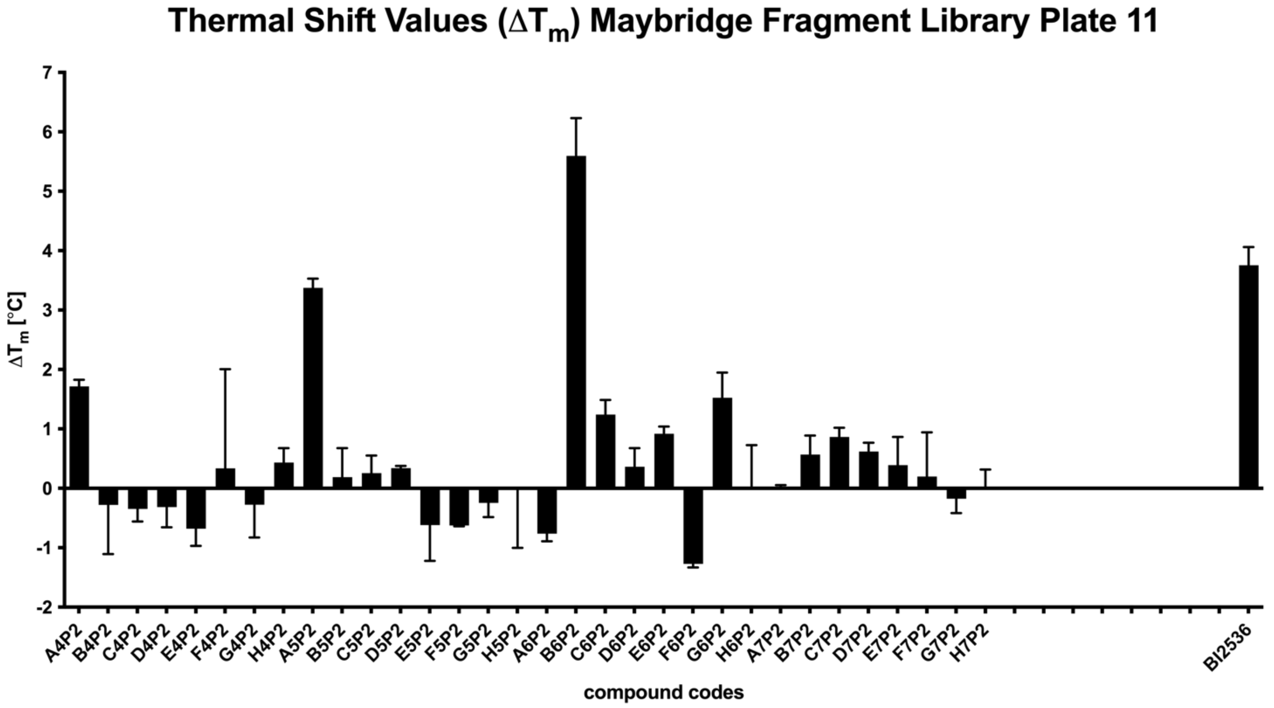

Figure S-18. Plate 11. Binding of compounds (150 µM) from the Maybridge Fragment Library to *Sm*BRD3(2) monitored by means of a differential scanning fluorimetry (DSF) setup (n = 2). BI2536 (**7**, 150 µM) was used as a positive control. Mean values ± standard deviation are shown.

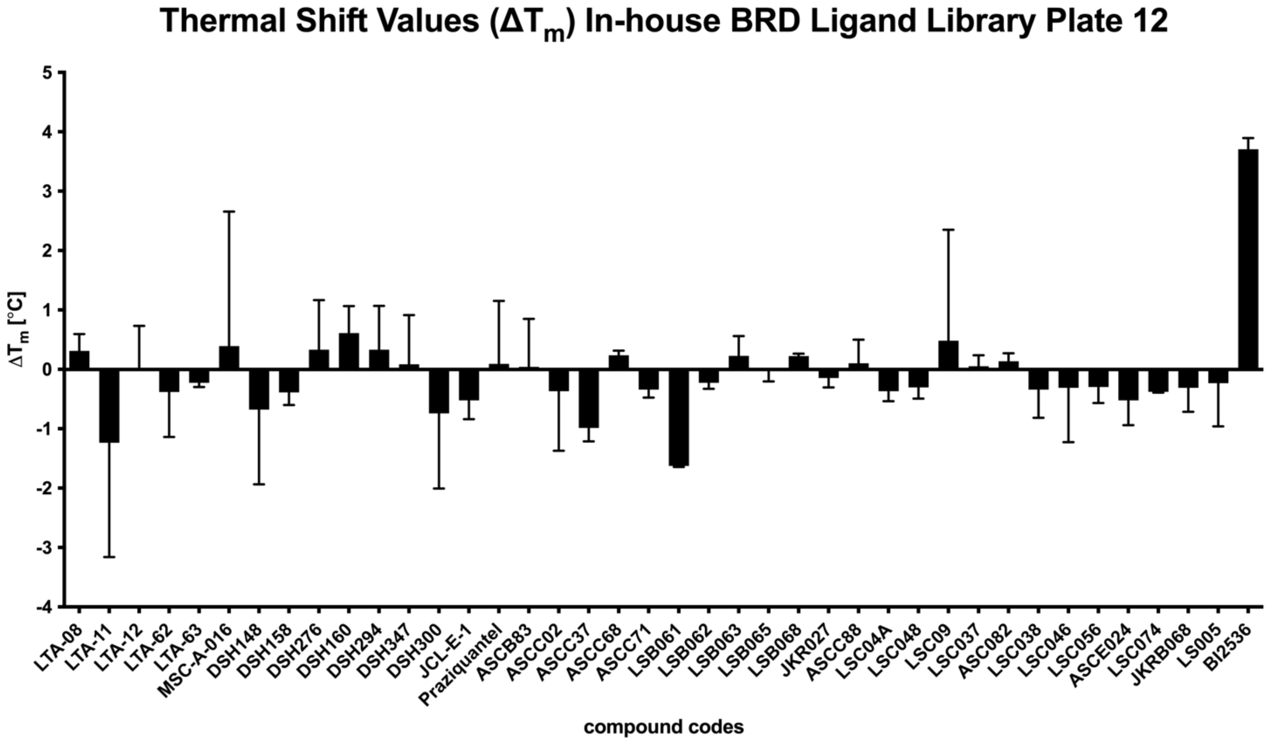

Figure S-19. Plate 12. Binding of compounds (50 µM) of our in-house library to *Sm*BRD3(2) monitored by means of a differential scanning fluorimetry (DSF) setup (n = 2). BI2536 (**7**, 50 µM) was used as a positive control. Mean values ± standard deviation are shown.

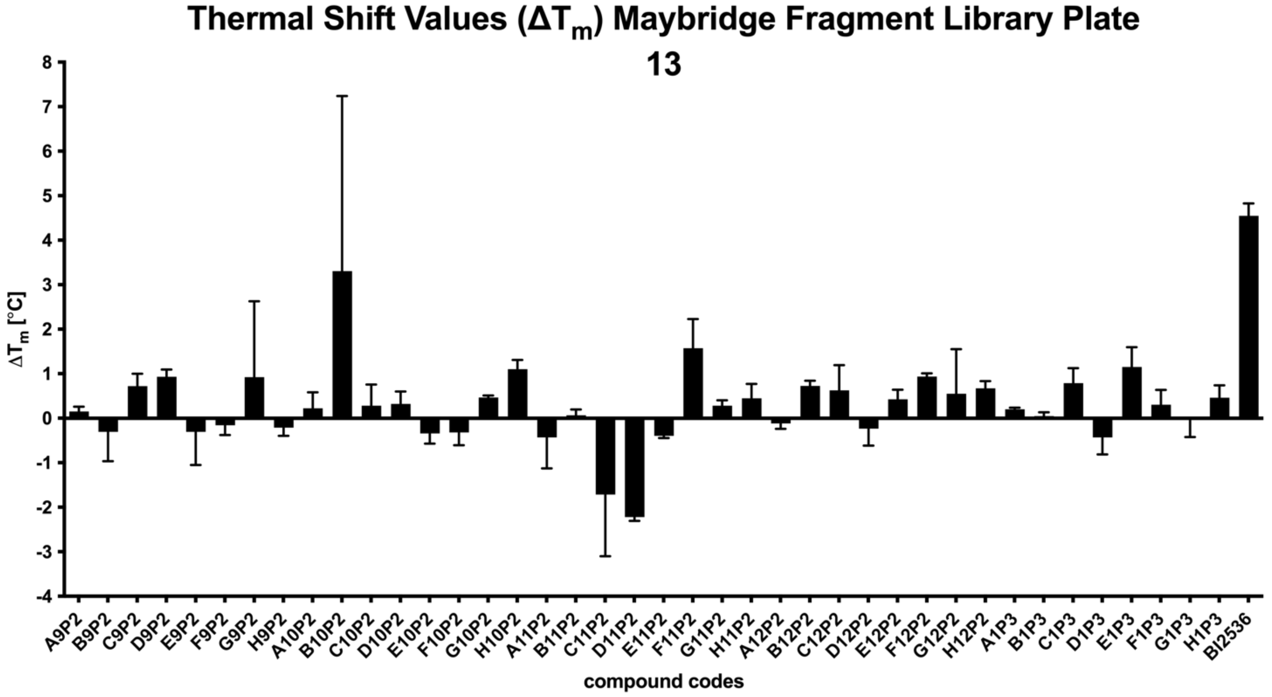

Figure S-20. Plate 13. Binding of compounds (150 µM) from the Maybridge Fragment Library to *Sm*BRD3(2) monitored by means of a differential scanning fluorimetry (DSF) setup (n = 2). BI2536 (**7**, 150 µM) was used as a positive control. Mean values ± standard deviation are shown.

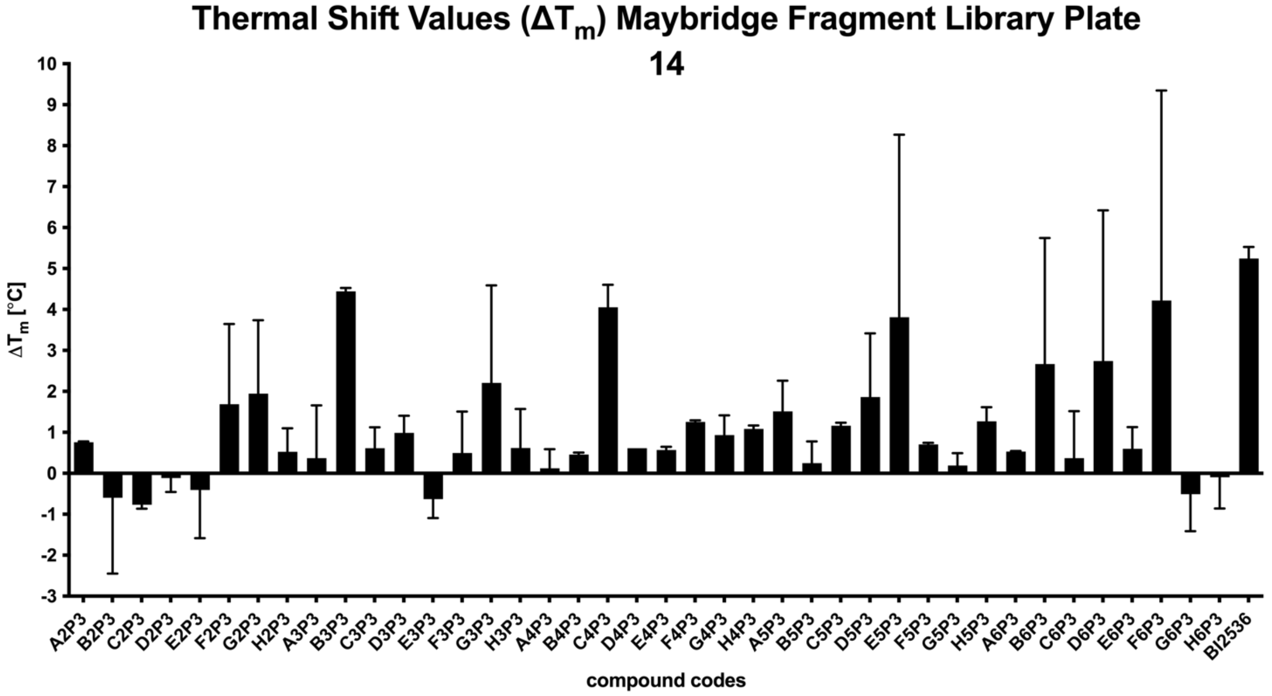

Figure S-21. Plate 14. Binding of compounds (150 µM) from the Maybridge Fragment Library to *Sm*BRD3(2) monitored by means of a differential scanning fluorimetry (DSF) setup (n = 2). BI2536 (**7**, 150 µM) was used as a positive control. Mean values ± standard deviation are shown.

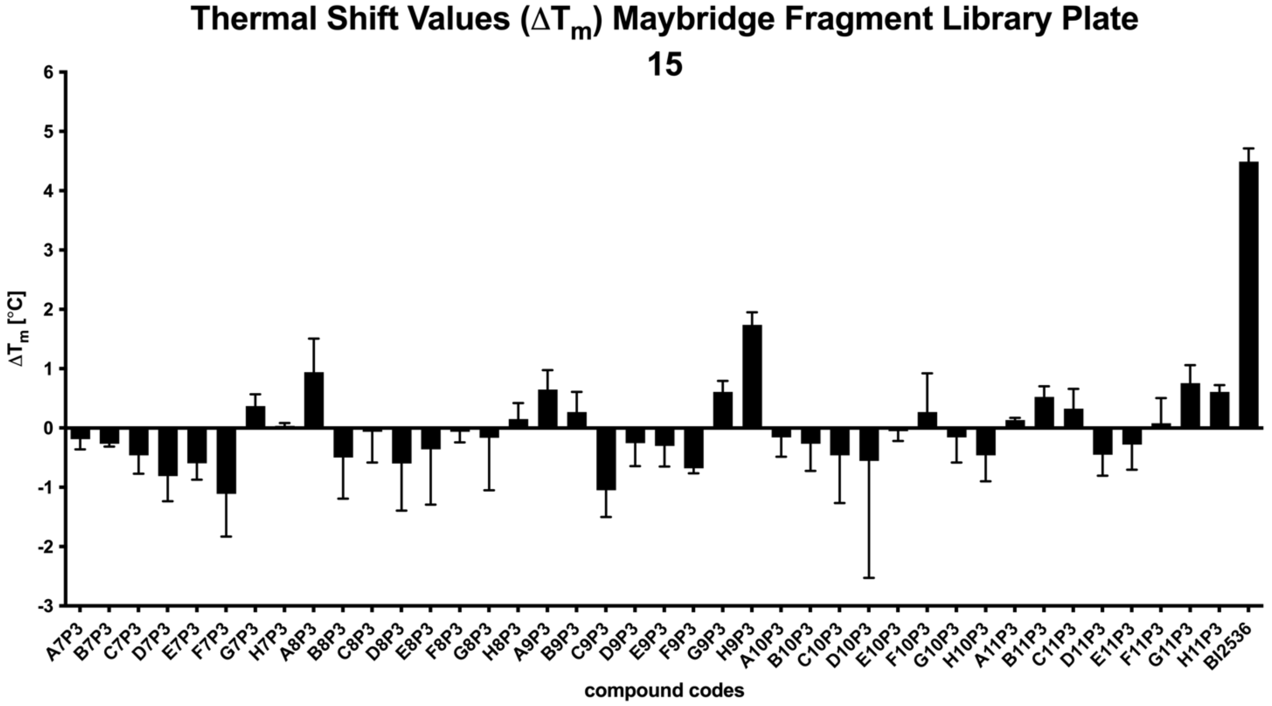

Figure S-22. Plate 15. Binding of compounds (150 µM) from the Maybridge Fragment Library to *Sm*BRD3(2) monitored by means of a differential scanning fluorimetry (DSF) setup (n = 2). BI2536 (**7**, 150 µM) was used as a positive control. Mean values ± standard deviation are shown.

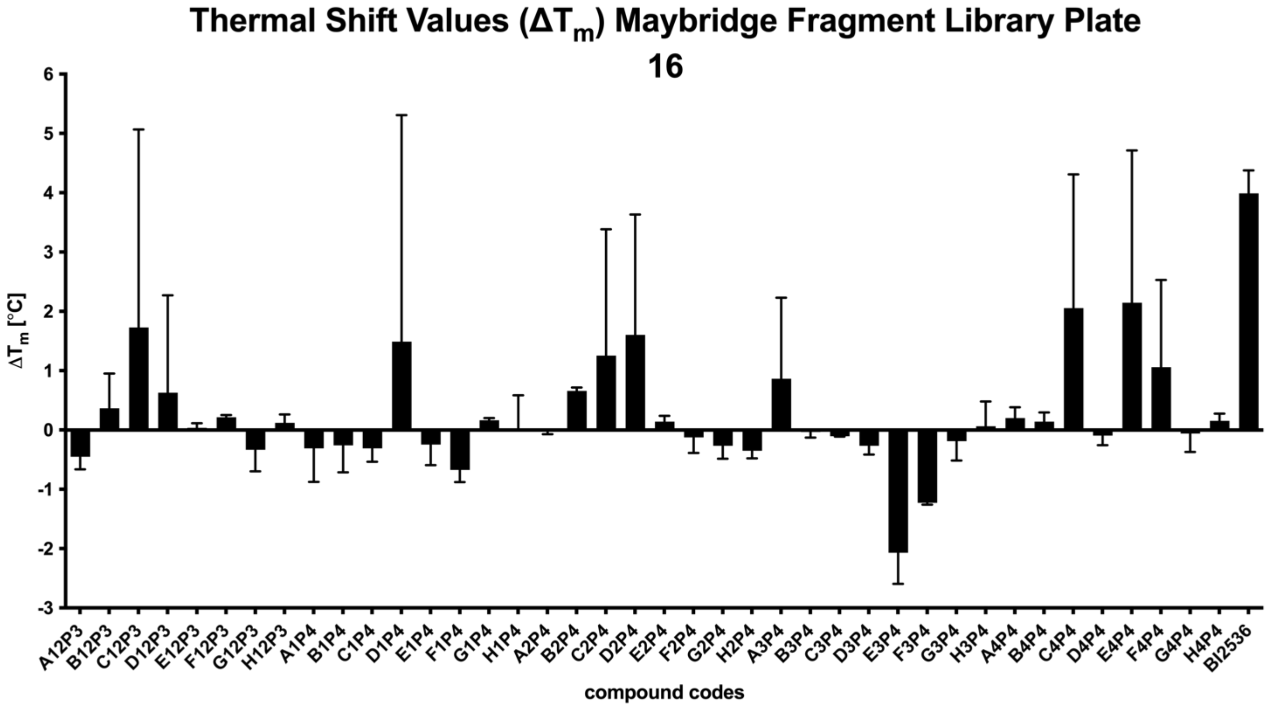

Figure S-23. Plate 16. Binding of compounds (150 µM) from the Maybridge Fragment Library to *Sm*BRD3(2) monitored by means of a differential scanning fluorimetry (DSF) setup (n = 2). BI2536 (**7**, 150 µM) was used as a positive control. Mean values ± standard deviation are shown.

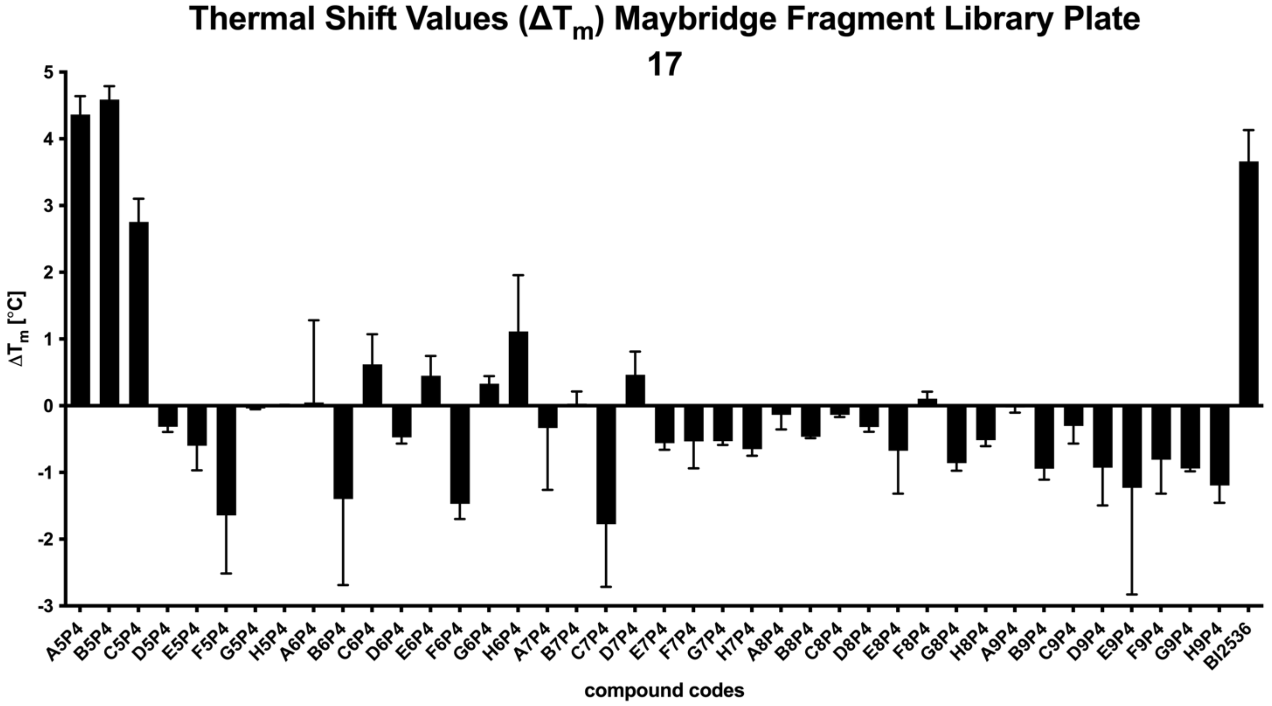

Figure S-24. Plate 17. Binding of compounds (150 µM) from the Maybridge Fragment Library to *Sm*BRD3(2) monitored by means of a differential scanning fluorimetry (DSF) setup (n = 2). BI2536 (**7**, 150 µM) was used as a positive control. Mean values ± standard deviation are shown.

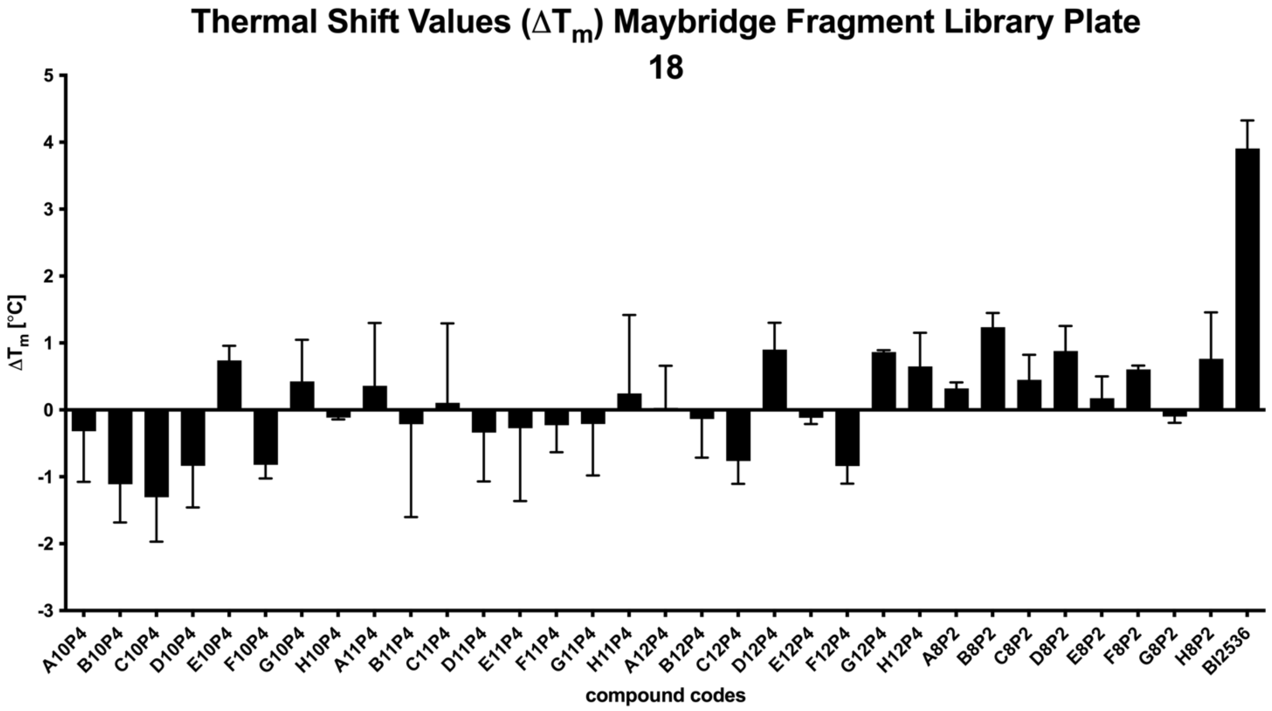

Figure S-25. Plate 18. Binding of compounds (150 µM) from the Maybridge Fragment Library to *Sm*BRD3(2) monitored by means of a differential scanning fluorimetry (DSF) setup (n = 2). BI2536 (**7**, 150 µM) was used as a positive control. Mean values ± standard deviation are shown.

Table S-2. K_d values and N numbers of compounds tested for binding to *Sm*BRD3(1,2) and *Sm*BRD3(2) using Isothermal Titration Calorimetry (ITC). Values are n=1 ± error of the curve of fit.

	**Binding to *Sm*BRD3(1,2)**	
**Compound Name / Number**	**K_d (nM)**	**N**
OXFBD02 (**1**)	683 ± 166	1.61 ± 3.3e-2
OXFBD03 (**2**)	675 ± 97.6	1.17 ± 1.7e-2
**3**	1750 ± 301	1.05 ± 3.9e-2
OXFBD04 (**4**)	2950 ± 261	1.39 ± 2.0e-2
(+)-JQ1 (**5**)	445 ± 106	3.87 ± 7.0 e-2
I-BET151 (**6**)	2830 ± 283	2.63 ± 3.8e-2
BI2536 (**7**)	1990 ± 277	3.39 ± 6.3e-2
I-BET726 (**8**)	1520 ± 520	1.66 ± 6.7e-2
**10**	No binding	-

Table S-3. K_d values and N numbers of compounds tested by Isothermal Titration Calorimetry (ITC) for binding to *Sm*BRD3(1,2) and *Sm*BRD3(2). s.e.m. = standard error of the mean.

Binding to *Sm*BRD3(2)										
	**Repeat 1**		**Repeat 2**		**Repeat 3**		**Mean**		**s.e.m.**	
**Compound Name/Number**	**K_d (nM)**	**N**	**K_d (nM)**	**N**	**K_d (nM)**	**N**	**K_d (nM)**	**N**	**K_d**	**N**
OXFBD02 (**1**)	No binding	-	-	-	-	-	No binding	n/a	n/a	n/a
OXFBD03 (**2**)	No binding	-	-	-	-	-	No binding	n/a	n/a	n/a
**3**	No binding	-	-	-	-	-	No binding	n/a	n/a	n/a
OXFBD04 (**4**)	No binding	-	-	-	-	-	No binding	n/a	n/a	n/a
(+)-JQ1 (**5**)	No binding	-	-	-	-	-	No binding	n/a	n/a	n/a
I-BET151 (**6**)	6160 ± 785	1.43 ± 2.7e-2	-	-	-	-	6160 ± 785	1.43 ± 2.7e-2	n/a	n/a
BI2536 (**7**)	3810 ± 576	1.46 ± 2.9e-2	-	-	-	-	3810 ± 576	1.46 ± 2.9e-2	n/a	n/a
I-BET726 (**8**)	1850 ± 361	1.12 ± 4.2e-2	-	-	-	-	1850 ± 361	1.12 ± 4.2e-2	n/a	n/a
**9**	572 ± 103	1.52 ± 2.2e-2	778 ± 179	1.57 ± 3.4e-2	753 ± 176	1.64 ± 3.3e-2	701	1.58	64.9	3.50e-2
**10**	No binding	-	-	-	-	-	No binding	n/a	n/a	n/a
**11**	1060 ± 164	1.54 ± 2.5e-2	1300 ± 236	1.49 ± 3.0e-2	-	-	1180	1.52	120	5.00e-3
**12**	562 ± 79.1	1.51 ± 1.8e-2	543 ± 60.3	1.55 ± 1.4e-2	-	-	553	1.53	9.5	2.00e-2
**13**	628 ± 94.9	1.52 ± 2.0e-2	652 ± 78.5	1.71 ± 1.8e-2	672 ± 76.0	1.60 ± 1.6e-2	651	1.61	12.7	5.50e-2
**14**	1010 ± 160	1.42 ± 2.4e-2	1270 ± 779	1.54 ± 0.101	1270 ± 349	1.49 ± 5.4e-2	1183	1.48	86.7	3.50e-2
**15**	320 ± 82.8	1.48 ± 2.5e-2	361 ± 70.2	1.51 ± 2.0e-2	411 ± 60.4	1.69 ± 1.8e-2	364	1.56	26.3	6.60e-2
**16**	No binding	-	-	-	-	-	No binding	n/a	n/a	n/a
**17**	No binding	-	-	-	-	-	No binding	n/a	n/a	n/a
**18**	No binding	-	-	-	-	-	No binding	n/a	n/a	n/a
**19**	No binding	-	-	-	-	-	No binding	n/a	n/a	n/a
**20**	No binding	-	-	-	-	-	No binding	n/a	n/a	n/a
**21**	No binding	-	-	-	-	-	No binding	n/a	n/a	n/a
**22**	No binding	-	-	-	-	-	No binding	n/a	n/a	n/a

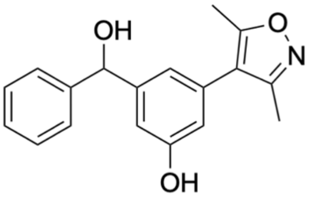

OXFBD02 (**1**)

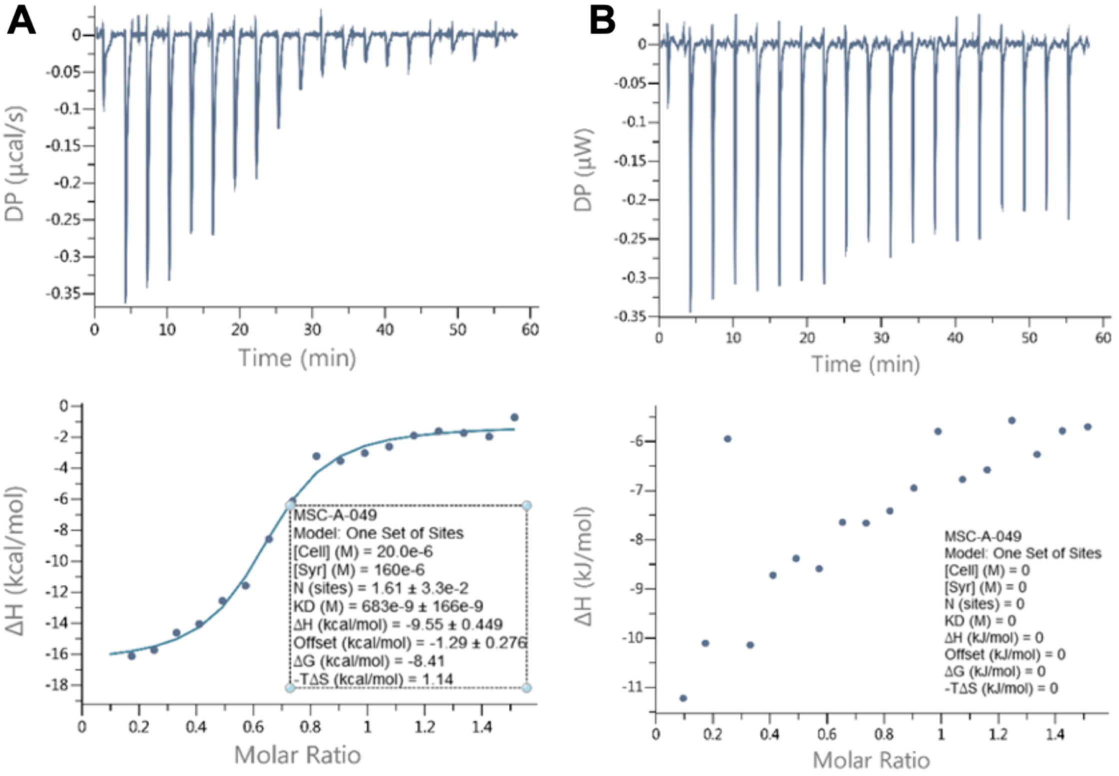

Figure S-26. A. Isothermal Titration Calorimetry (ITC) trace of OXFBD02 (**1**) and *Sm*BRD3(1,2) (K_d = 683 ± 166 nM). **B.** ITC trace of OXFBD02 (**1**) and *Sm*BRD3(2) (No detectable binding).

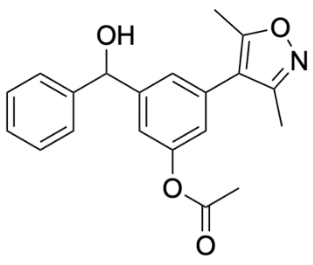

OXFBD03 (**2**)

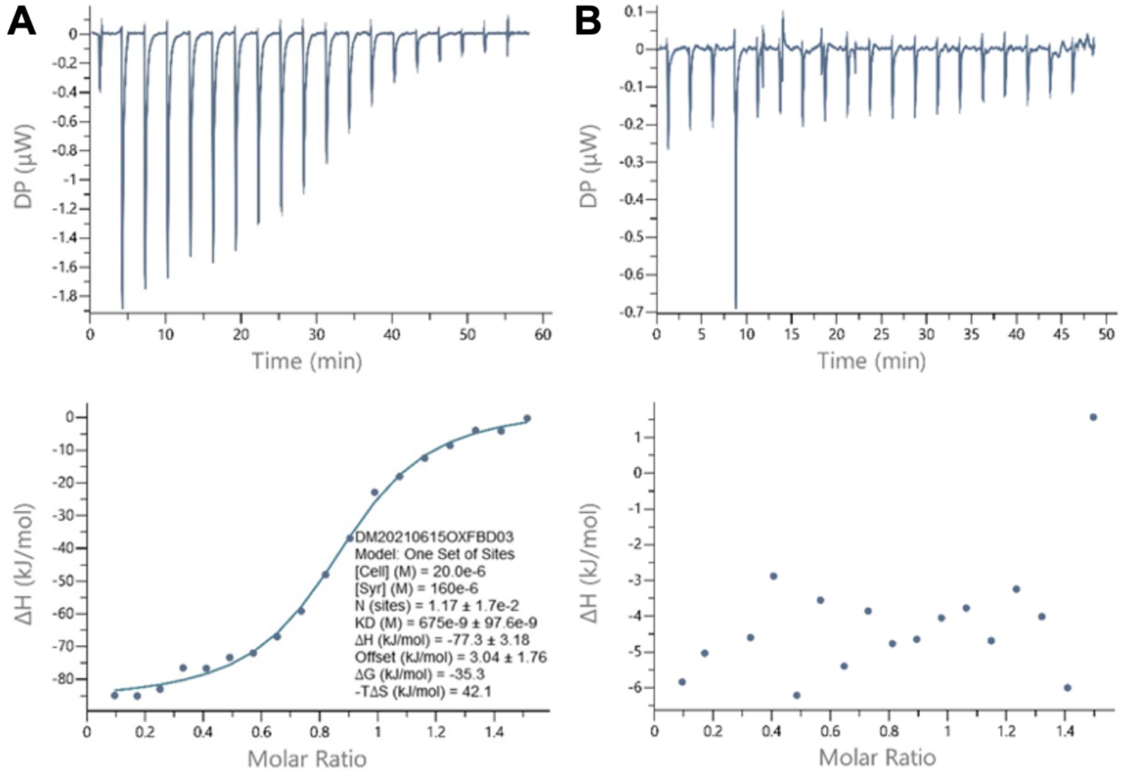

Figure S-27. A. Isothermal Titration Calorimetry (ITC) trace of OXFBD03 (**2**) and *Sm*BRD3(1,2) (K_d = 675 ± 97.6 nM). **B.** ITC trace of OXFBD03 (**2**) and *Sm*BRD3(2) (No detectable binding).

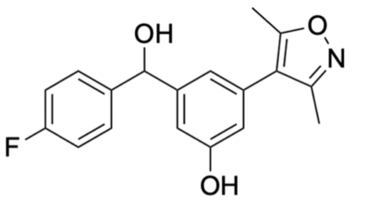

3

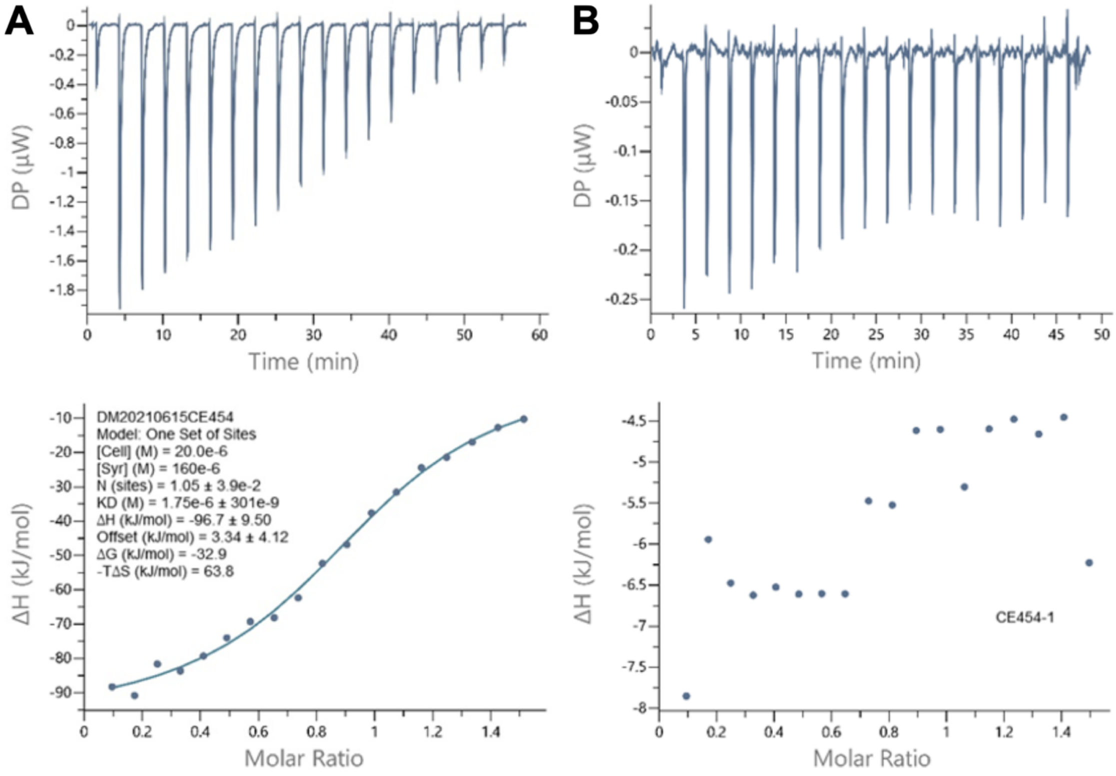

Figure S-28. A. Isothermal Titration Calorimetry (ITC) trace of **3** and *Sm*BRD3(1,2) (K_d = 1750 ± 301 nM). **B.** ITC trace of **3** and *Sm*BRD3(2) (No detectable binding).

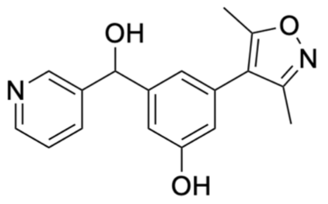

OXFBD04 (**4**)

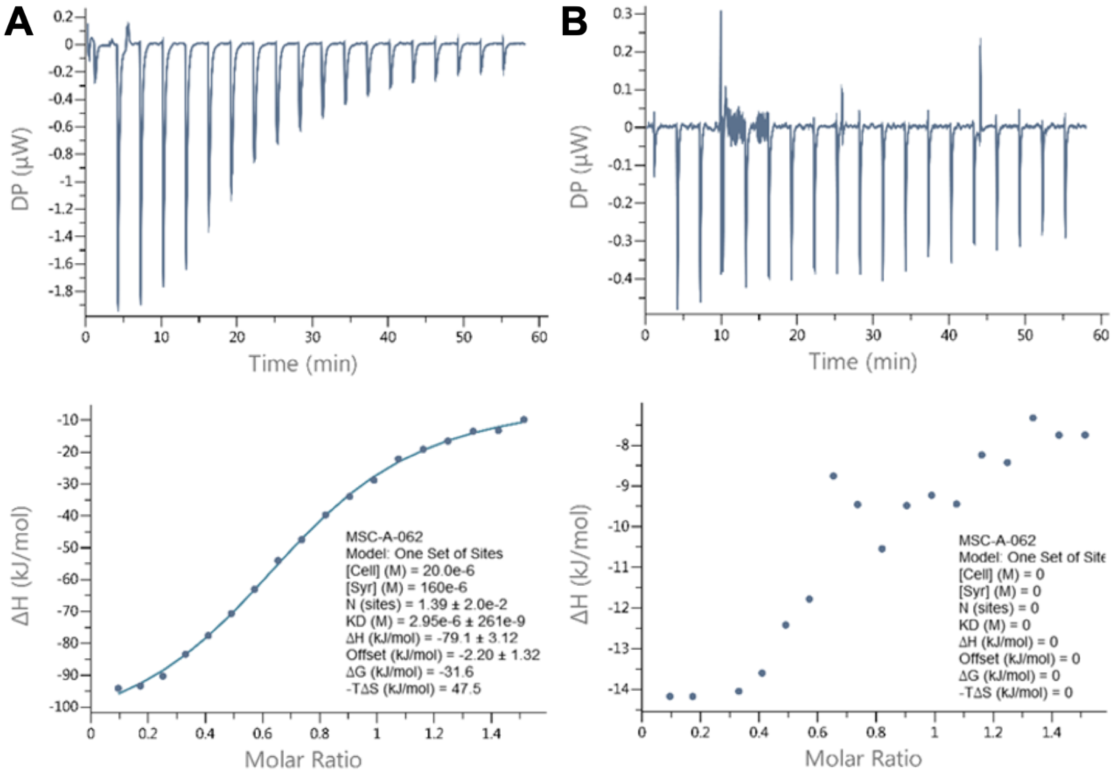

Figure S-29. A. Isothermal Titration Calorimetry (ITC) trace of OXFBD04 (**4**) and *Sm*BRD3(1,2) (K_d = 2950 ± 261 nM). **B.** ITC trace of OXFBD04 (**4**) and *Sm*BRD3(2) (No detectable binding).

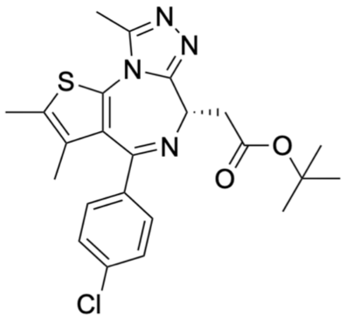

(+)-JQ1 (**5**)

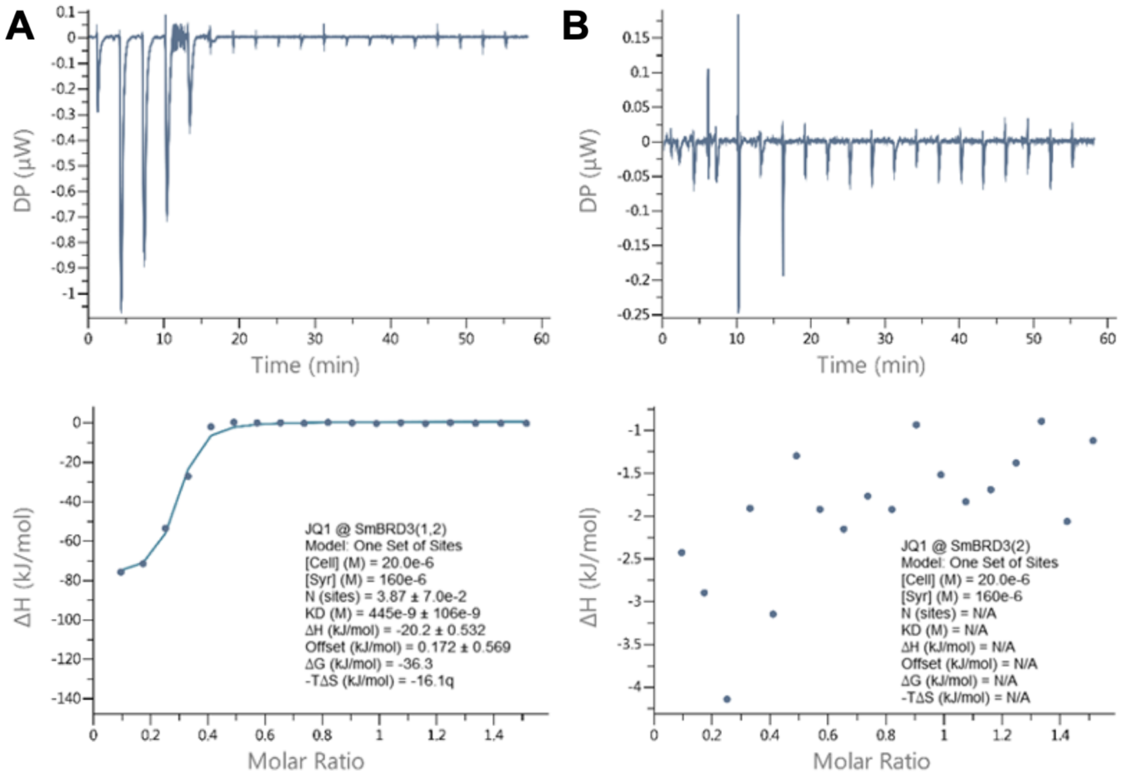

Figure S-30. A. Isothermal Titration Calorimetry (ITC) trace of (+)-JQ1 (**5**) and *Sm*BRD3(1,2) (K_d = 445 ± 106 nM) **B.** ITC trace of (+)-JQ1 (**5**) and *Sm*BRD3(2) (No detectable binding).

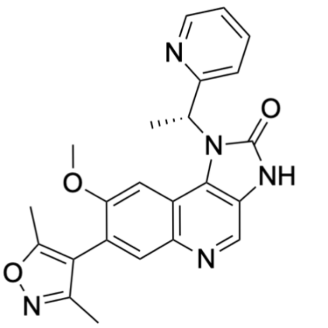

I-BET151 (**6**)

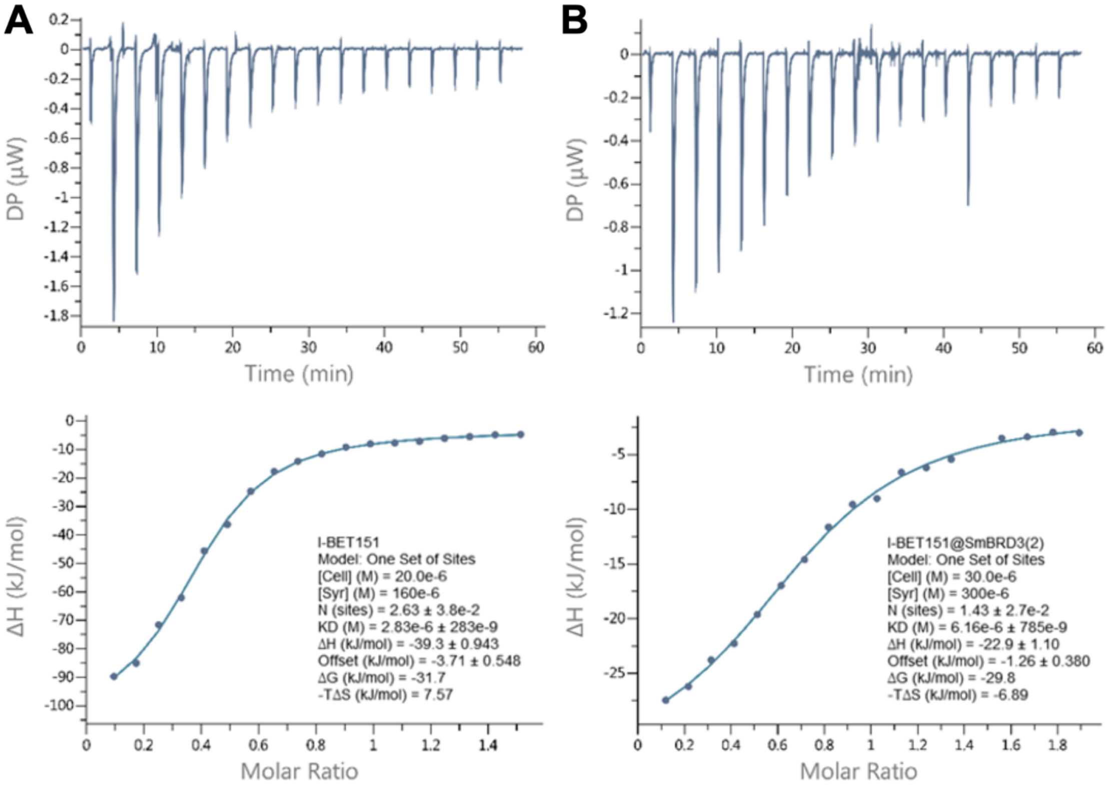

Figure S-31. A. Isothermal Titration Calorimetry (ITC) trace of I-BET151 (**6**) and *Sm*BRD3(1,2) (K_d = 2830 ± 283 nM). **B.** ITC trace of I-BET151 (**6**) and *Sm*BRD3(2) (K_d = 6160 ± 785 nM).

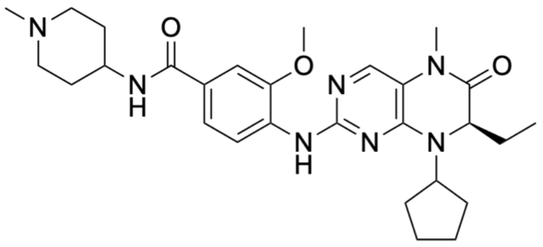

BI-2536 (**7**)

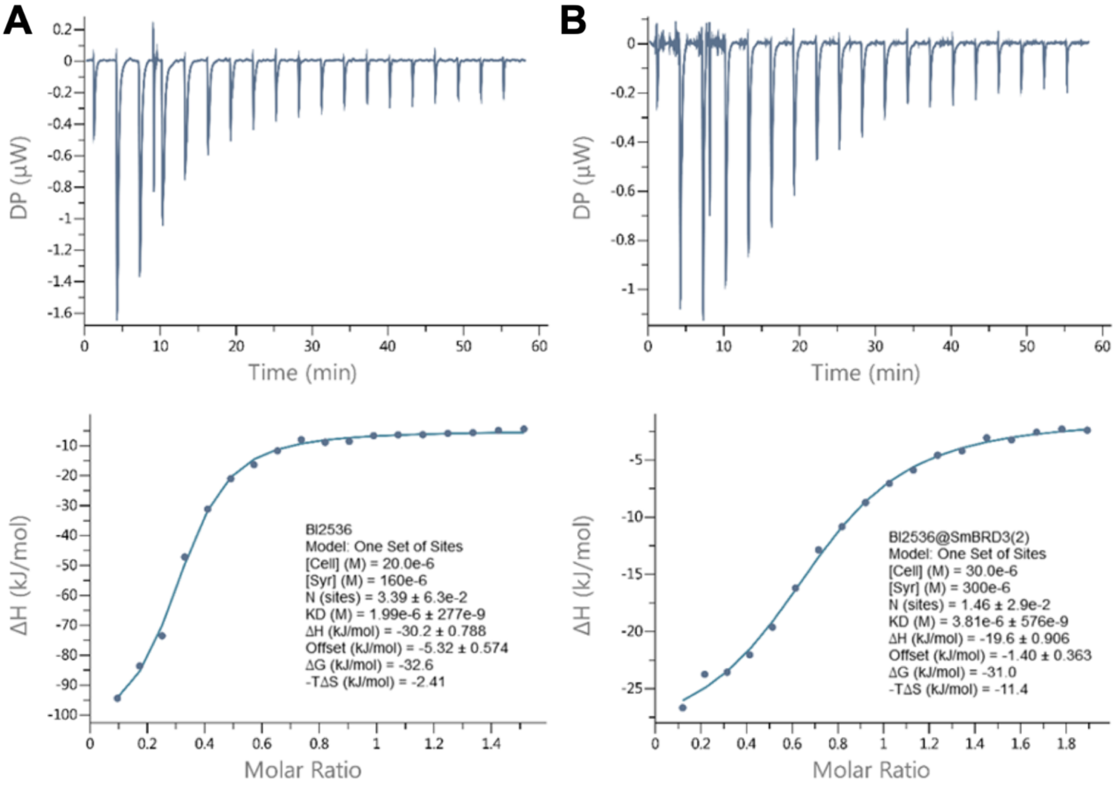

Figure S-32. A. Isothermal Titration Calorimetry (ITC) trace of BI-2536 (**7**) and *Sm*BRD3(1,2) (K_d = 1990 ± 277 nM). **B.** ITC trace of BI-2536 (**7**) and *Sm*BRD3(2) (K_d = 3810 ± 576 nM).

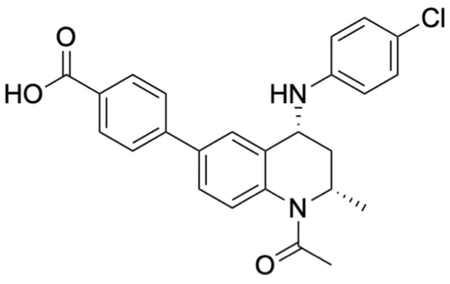

I-BET726 (**8**)

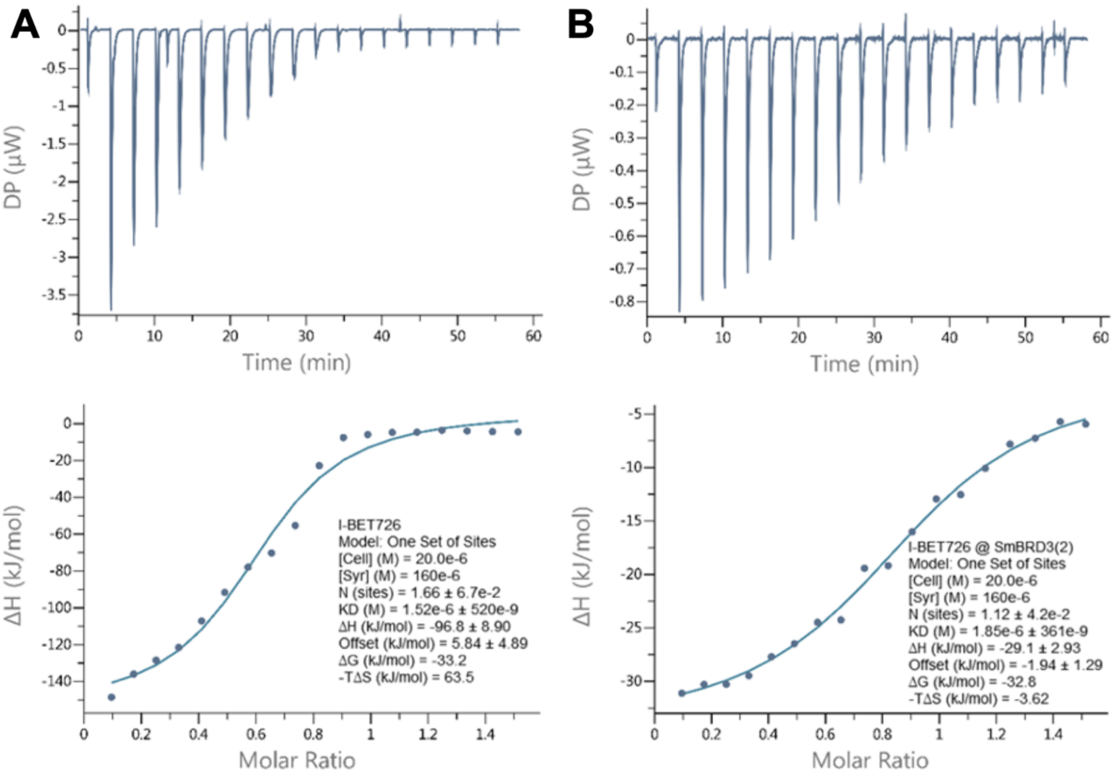

Figure S-33. A. Isothermal Titration Calorimetry (ITC) trace of I-BET726 (**8**) and *Sm*BRD3(1,2) (K_d = 1520 ± 520 nM). **B.** ITC trace of I-BET726 (**8**) and *Sm*BRD3(2) (K_d = 1850 ± 361 nM).

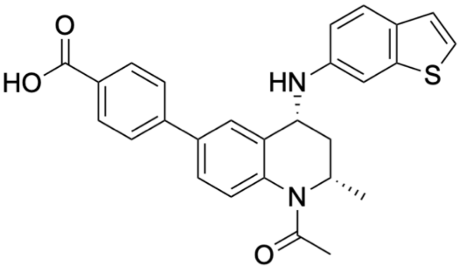

9

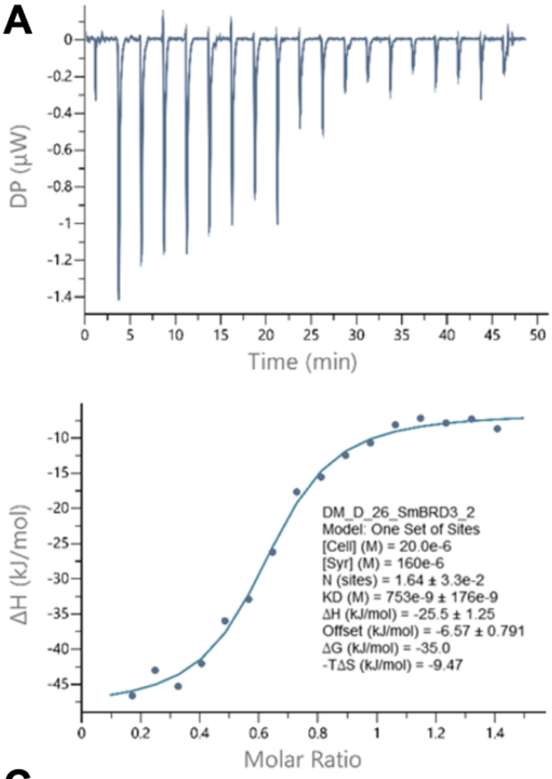

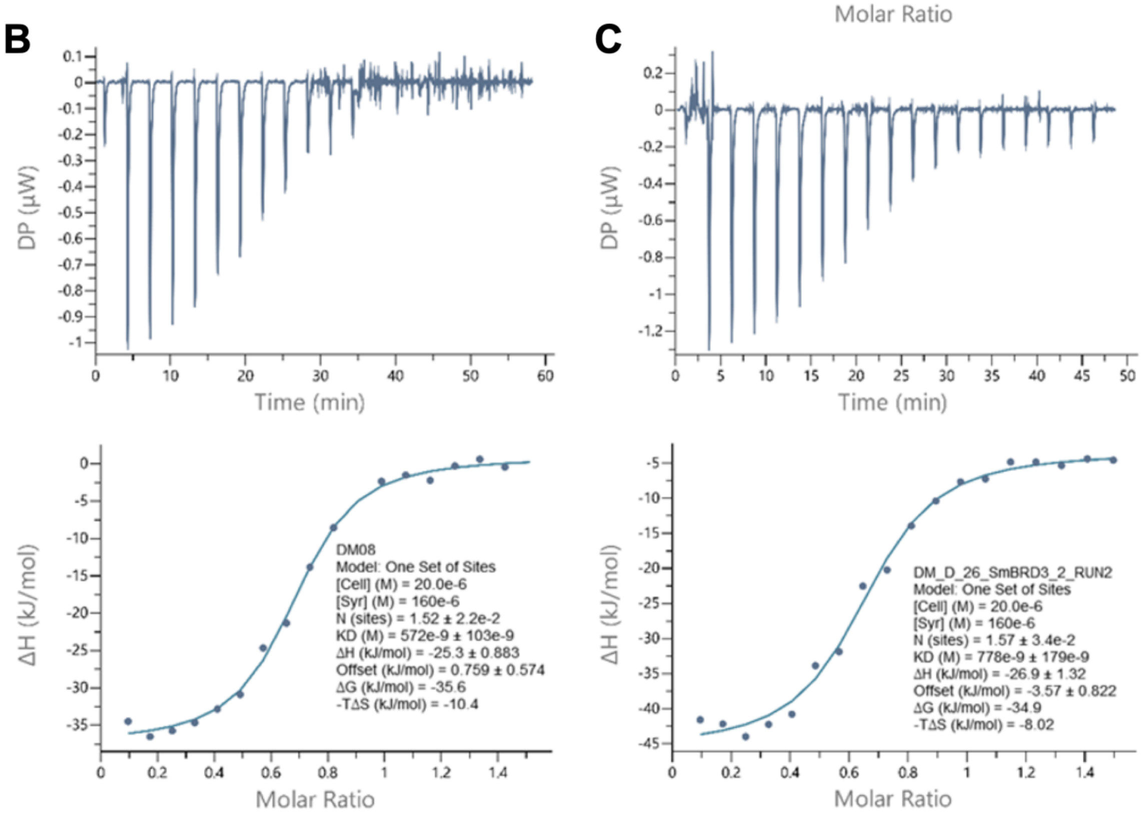

Figure S-34. A. Isothermal Titration Calorimetry (ITC) trace of **9** and *Sm*BRD3(2) (K_d = 753 ± 176 nM). **B.** ITC trace of **9** and *Sm*BRD3(2) (K_d = 572 ± 103 nM). **C.** ITC trace of **9** and *Sm*BRD3(2) (K_d = 778 ± 179 nM).

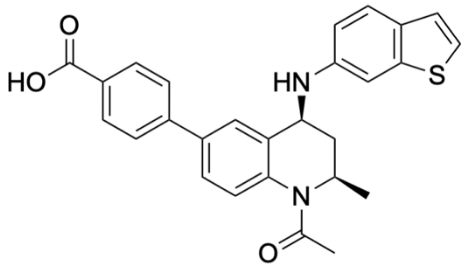

10

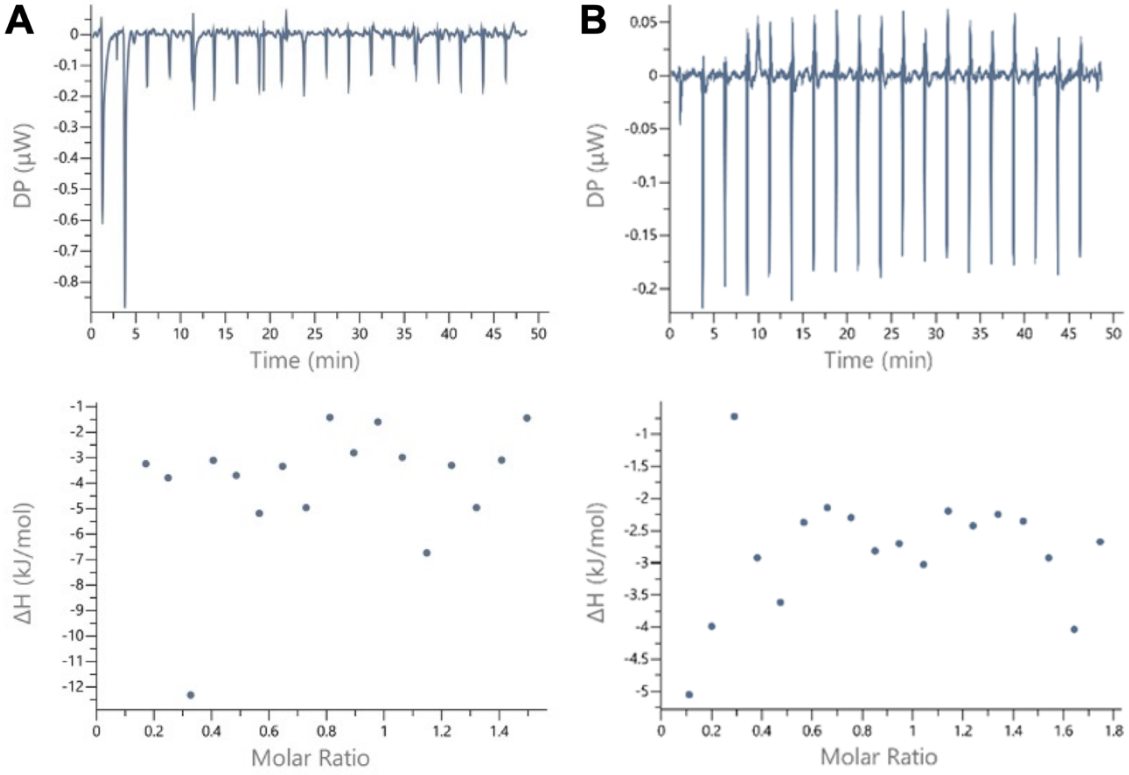

Figure S-35. A. Isothermal Titration Calorimetry (ITC) trace of **10** and *Sm*BRD3(1,2) (No detectable binding). **B.** Isothermal Titration Calorimetry (ITC) trace of **10** and *Sm*BRD3(2) (No detectable binding).

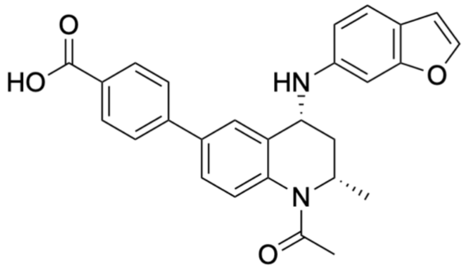

11

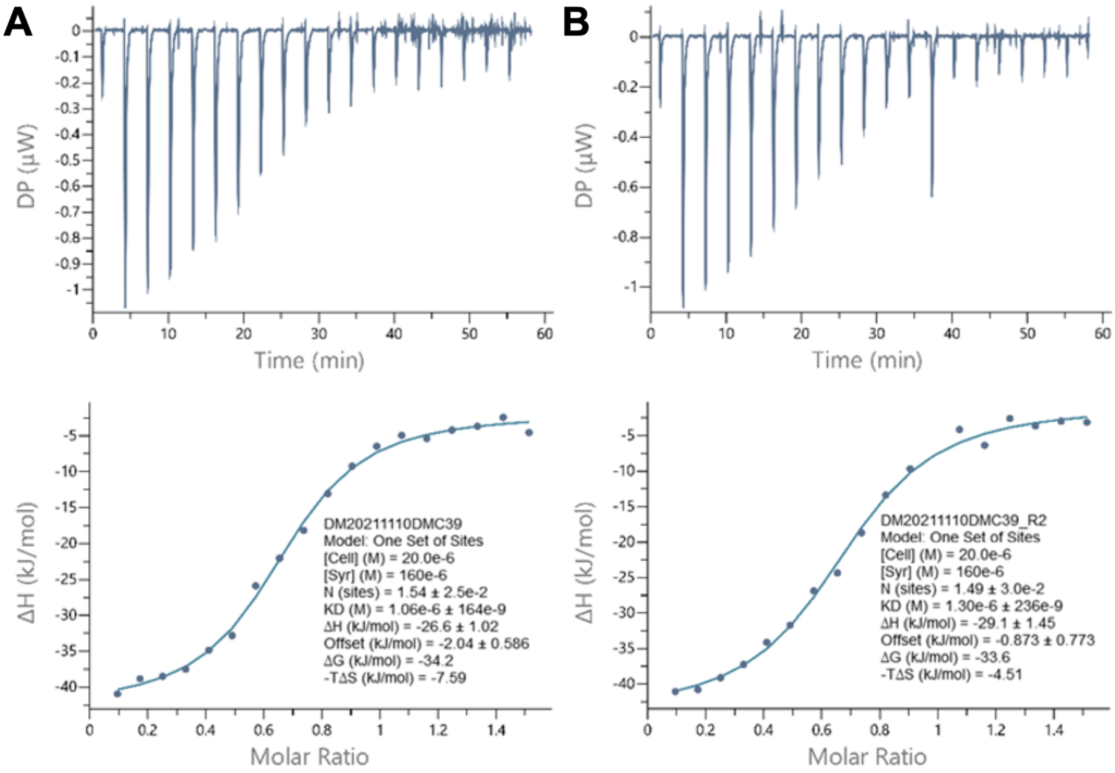

Figure S-36. A. Isothermal Titration Calorimetry (ITC) trace of **11** and *Sm*BRD3(2) (K_d = 1060 ± 164 nM). **B.** ITC trace of **11** and *Sm*BRD3(2) (K_d = 1300 ± 236 nM).

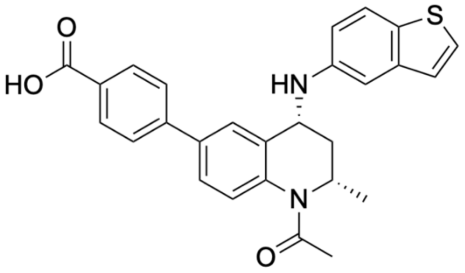

12

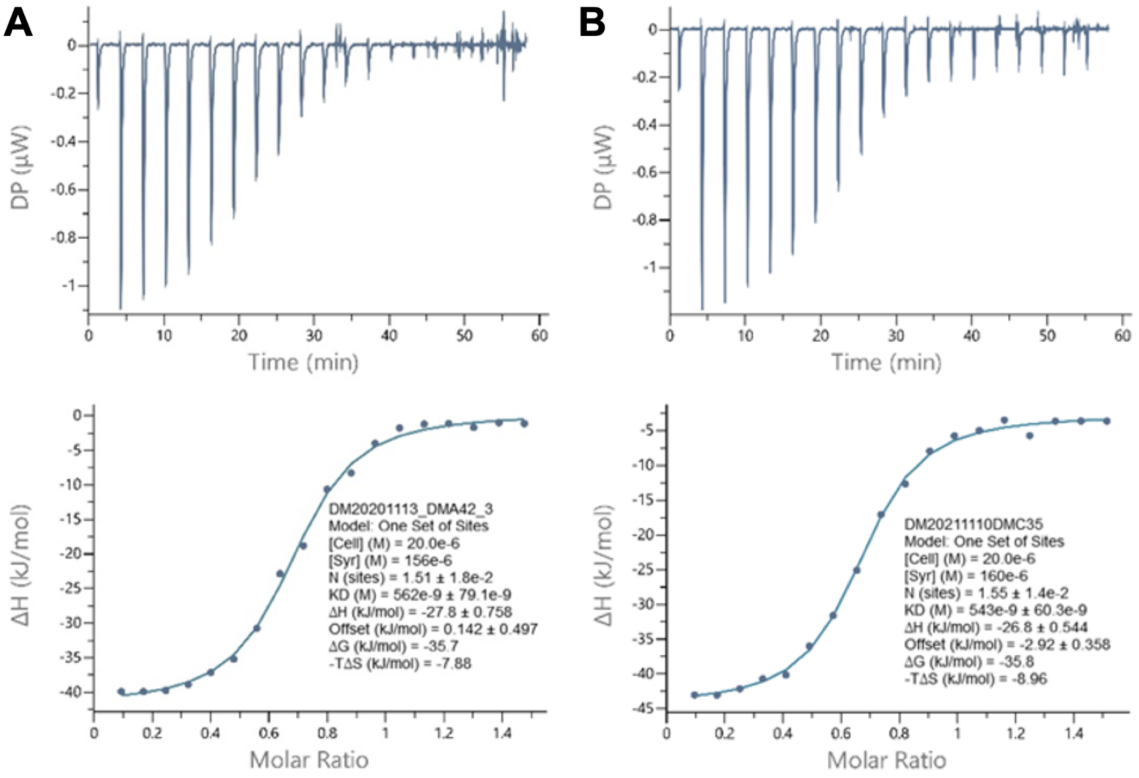

Figure S-37. A. Isothermal Titration Calorimetry (ITC) trace of **12** and *Sm*BRD3(2) (K_d = 562 ± 79.1 nM). **B.** ITC trace of **12** and *Sm*BRD3(2) (K_d = 543 ± 60.3 nM).

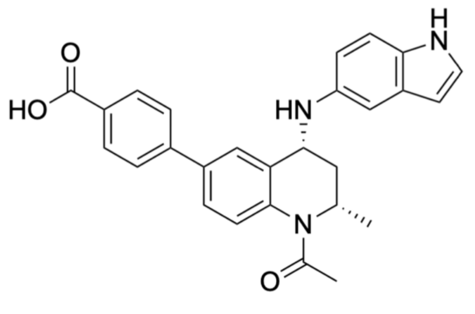

13

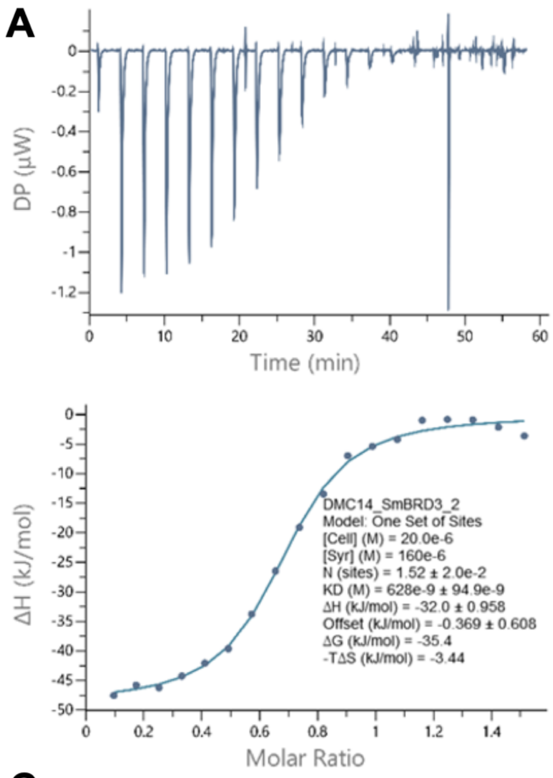

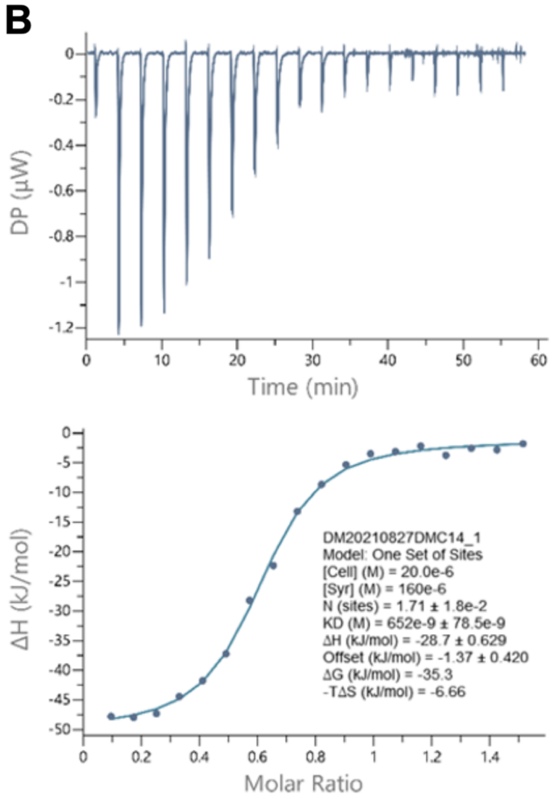

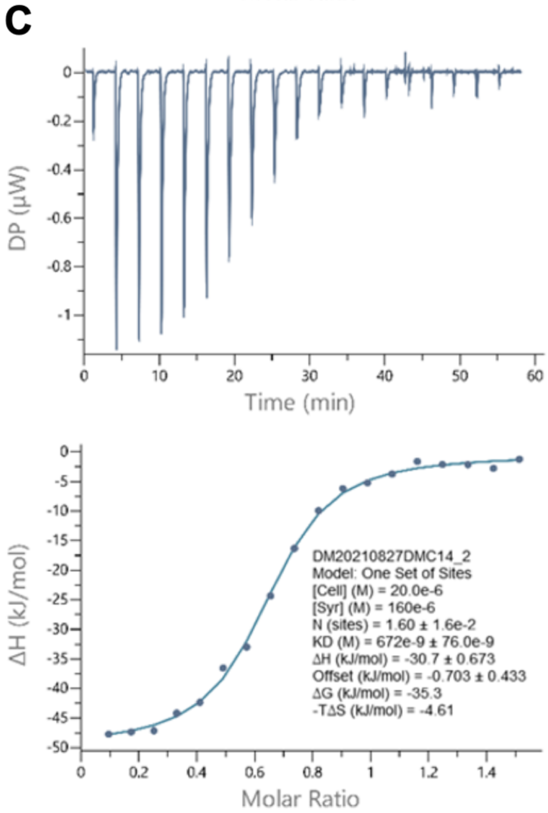

Figure S-38. A. Isothermal Titration Calorimetry (ITC) trace of **13** and *Sm*BRD3(2) (K_d = 628 ± 94.9 nM). **B.** ITC trace of **13** and *Sm*BRD3(2) (K_d = 652 ± 78.5 nM). **C.** ITC trace of **13** and *Sm*BRD3(2) (K_d = 672 ± 76.0 nM).

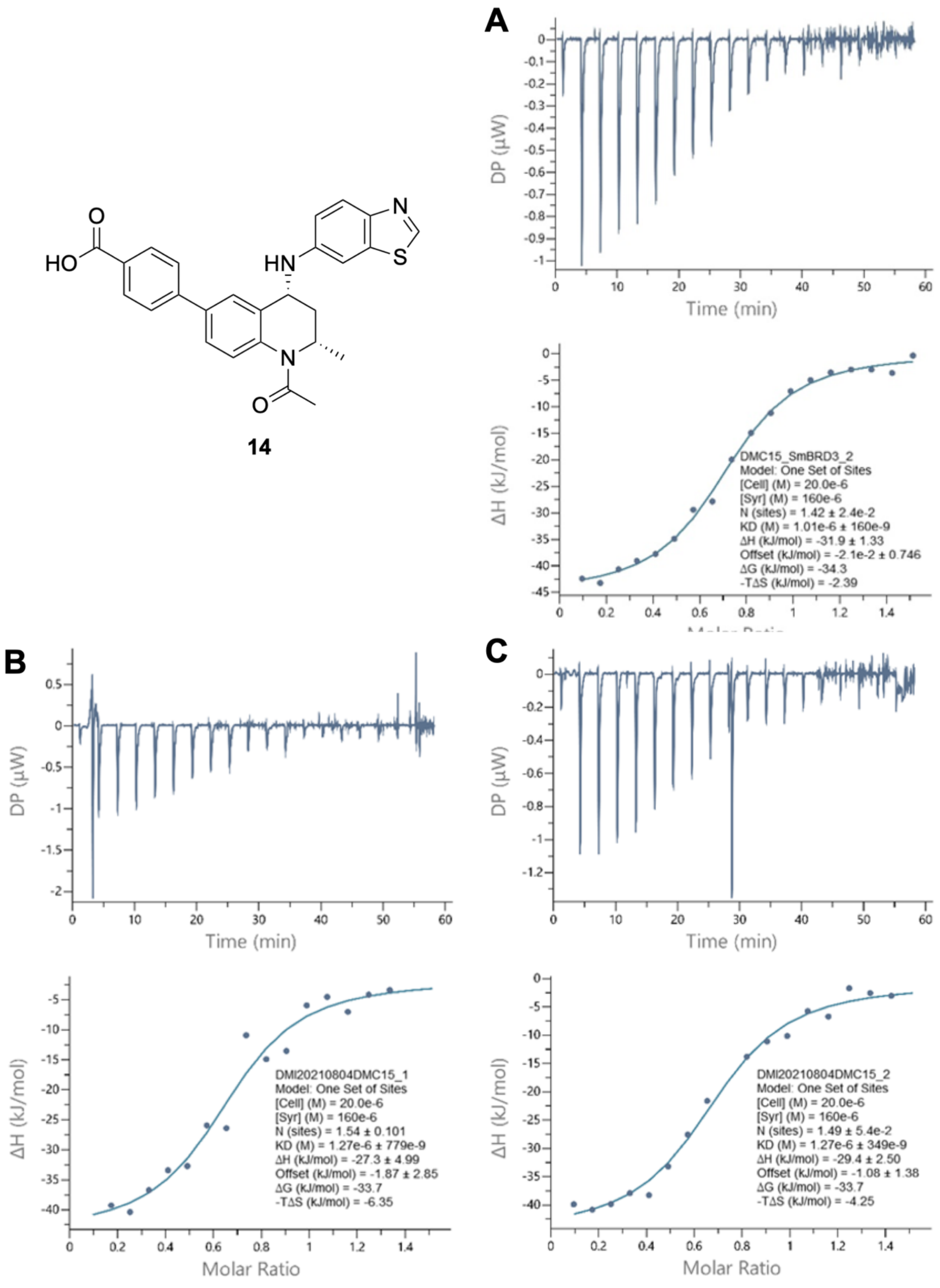

Figure S-39. A. Isothermal Titration Calorimetry (ITC) trace of **14** and *Sm*BRD3(2) (K_d = 1010 ± 160 nM). **B.** ITC trace of **14** and *Sm*BRD3(2) (K_d = 1270 ± 779 nM). **C.** ITC trace of **14** and *Sm*BRD3(2) (K_d = 1270 ± 349 nM).

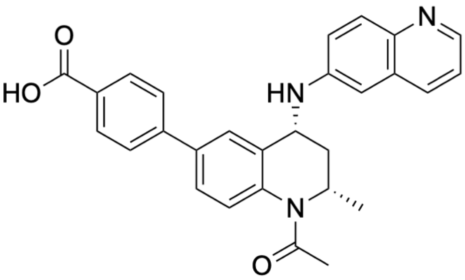

15

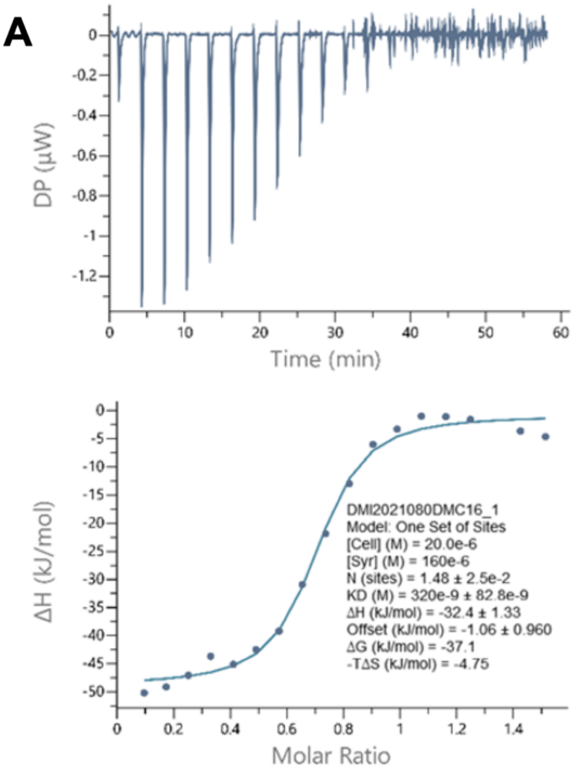

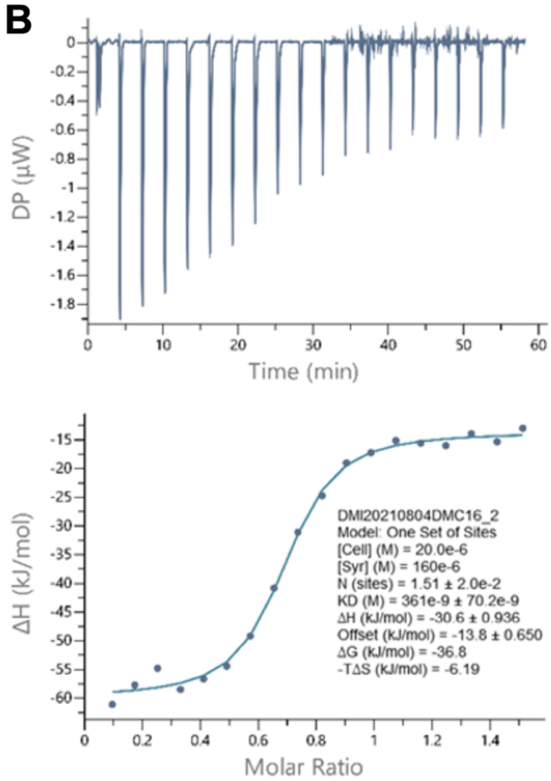

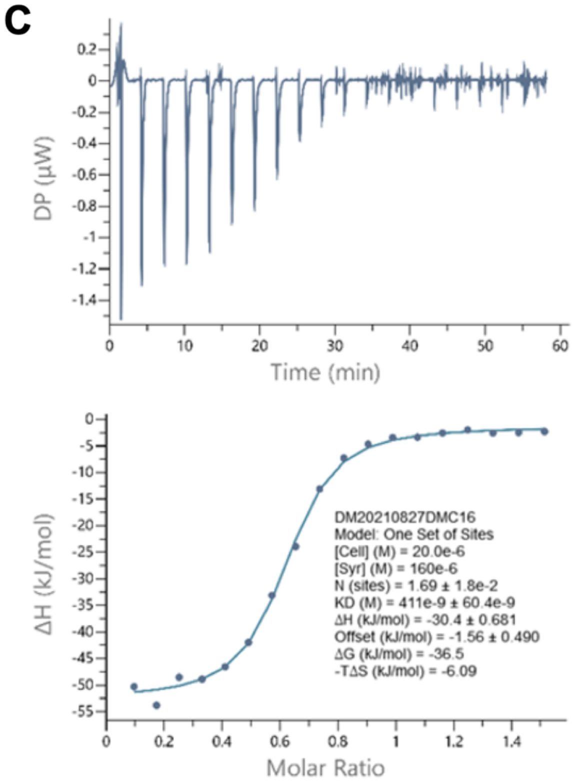

Figure S-40. A. Isothermal Titration Calorimetry (ITC) trace of **15** and *Sm*BRD3(2) (K_d = 320 ± 82.8 nM). **B.** ITC trace of **15** and *Sm*BRD3(2) (K_d = 361 ± 70.2 nM). **C.** ITC trace of **15** and *Sm*BRD3(2) (K_d = 411 ± 60.4 nM).

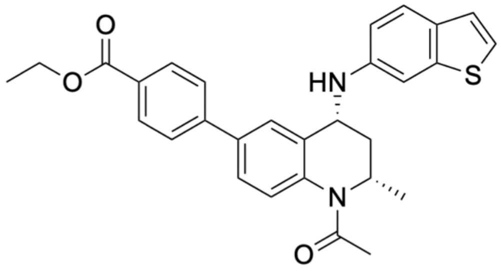

16

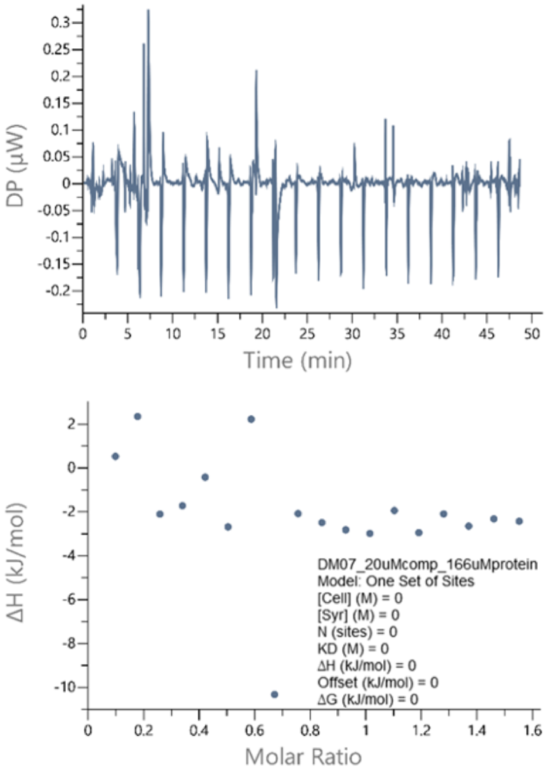

Figure S-41. Isothermal Titration Calorimetry (ITC) trace of **16** and *Sm*BRD3(2) (No detectable binding).

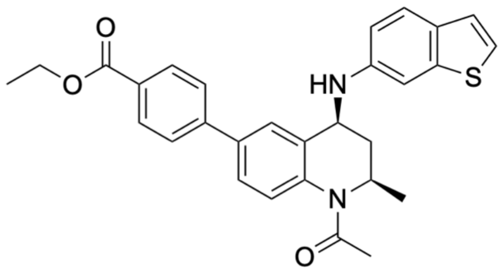

17

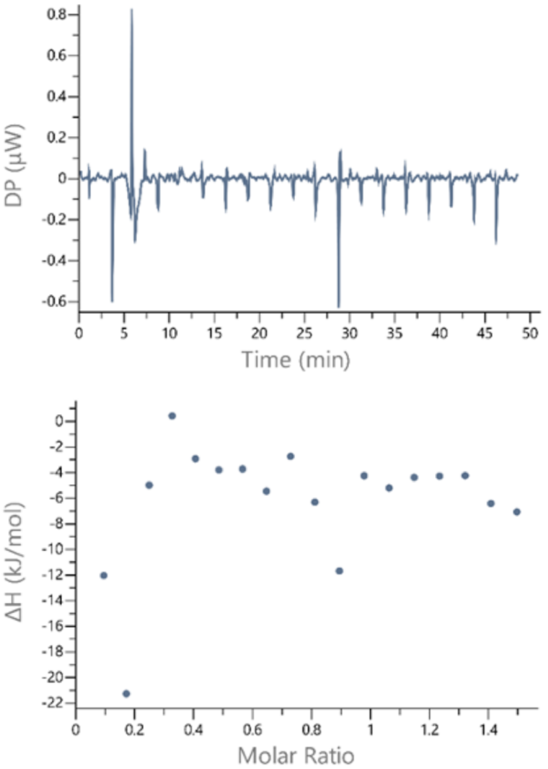

Figure S-42. Isothermal Titration Calorimetry (ITC) trace of **17** and *Sm*BRD3(2) (No detectable binding).

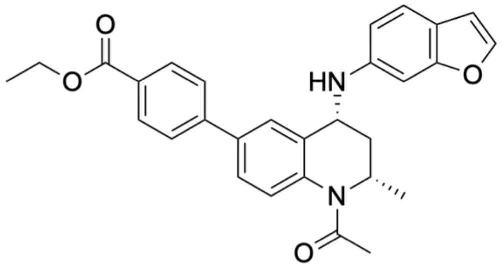

18

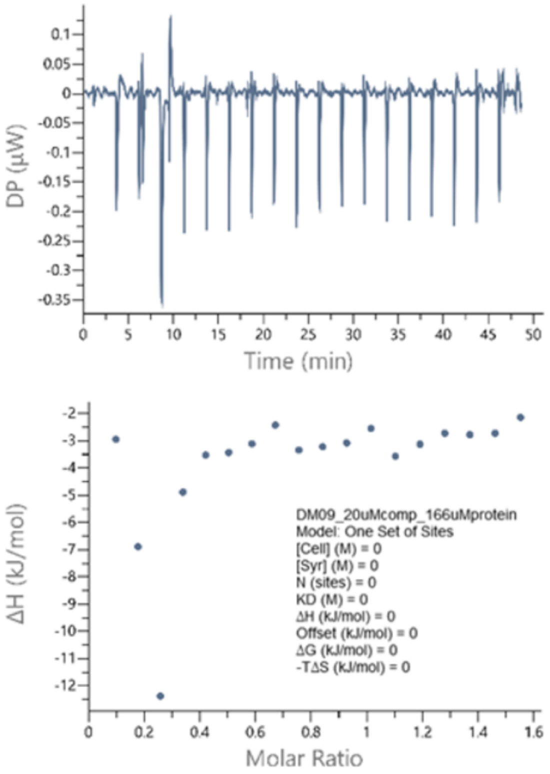

Figure S-43. Isothermal Titration Calorimetry (ITC) trace of **18** and *Sm*BRD3(2) (No detectable binding).

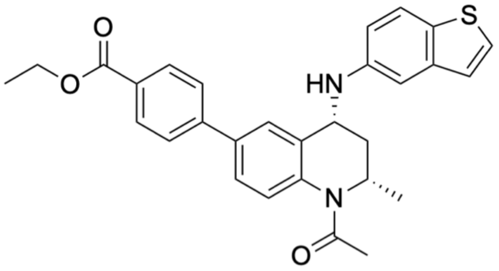

19

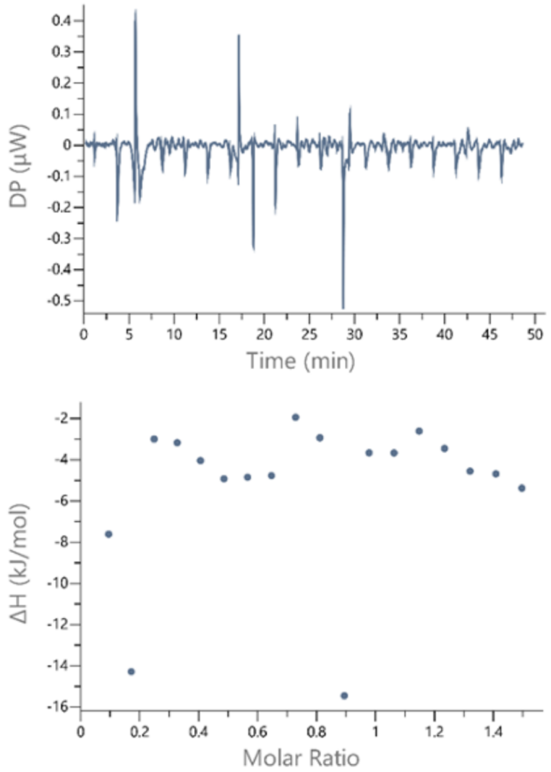

Figure S-44. Isothermal Titration Calorimetry (ITC) trace of **19** and *Sm*BRD3(2) (No detectable binding).

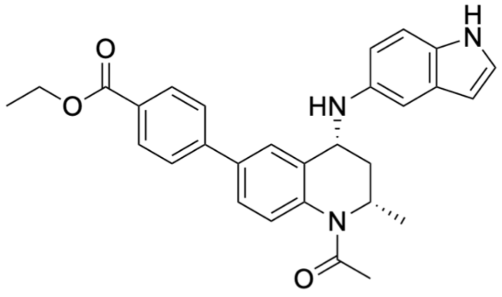

20

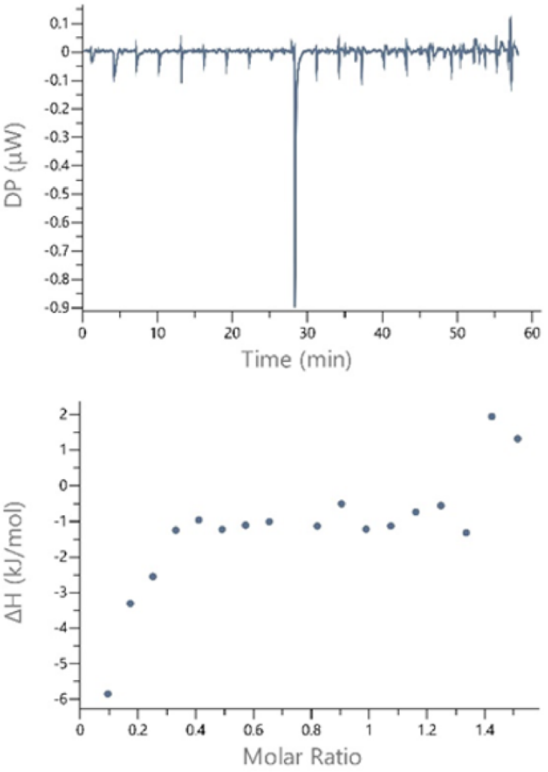

Figure S-45. Isothermal Titration Calorimetry (ITC) trace of **20** and *Sm*BRD3(2) (No detectable binding).

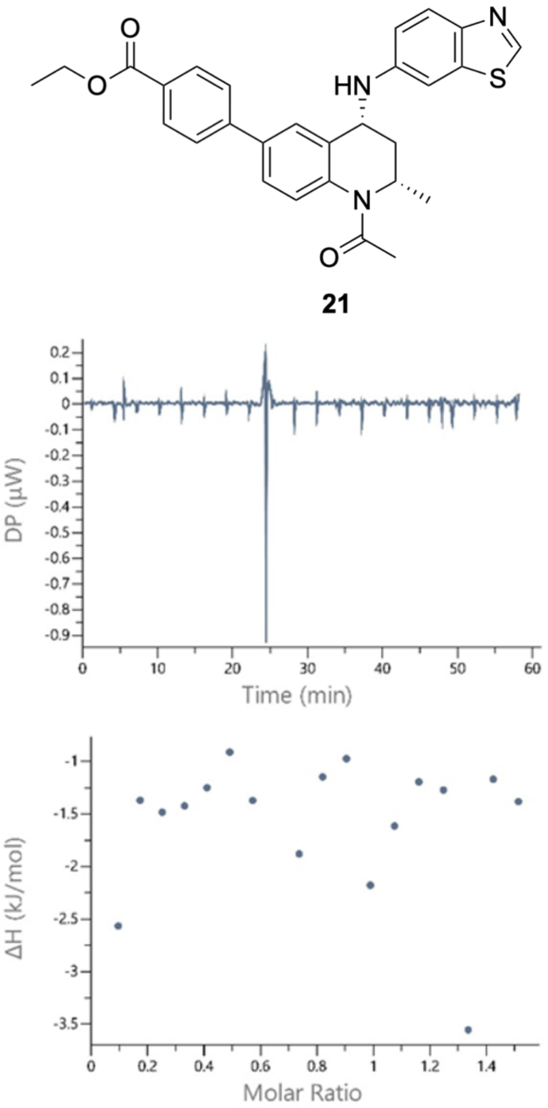

Figure S-46. Isothermal Titration Calorimetry (ITC) trace of **21** and *Sm*BRD3(2) (No detectable binding).

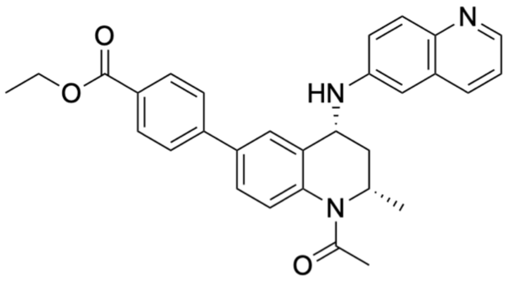

22

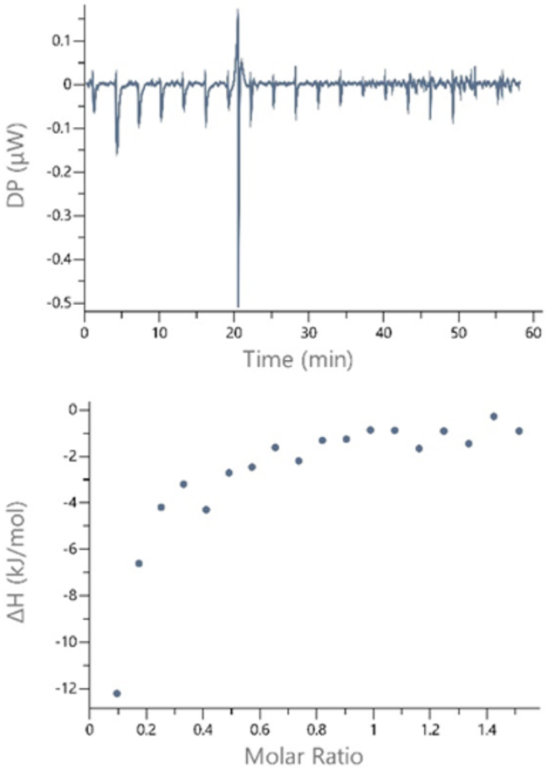

Figure S-47. Isothermal Titration Calorimetry (ITC) trace of **22** and *Sm*BRD3(2) (No detectable binding).

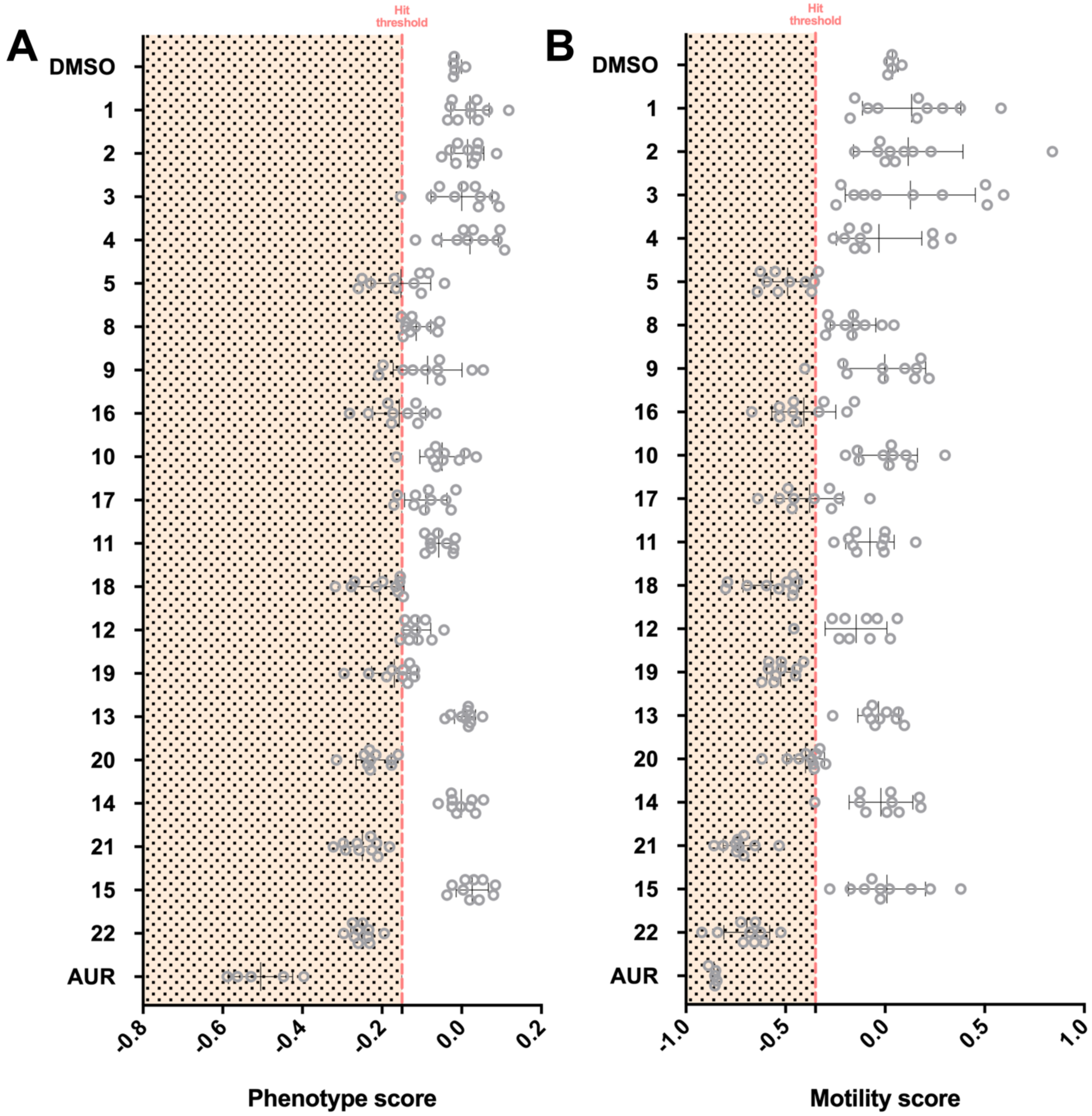

Figure S-48. The effect of compounds **1–5** and **8–22** on the (**A**) phenotype and (**B**) motility of schistosomula tested at 10 μM in 0.625% DMSO, following 72 h incubation. Negative (0.625% DMSO) and positive (10 μM auranofin in 0.625% DMSO) controls are included in each drug screen (4-5 in total, two technical replicates each). The compound score is shown as grey dot and whiskers represent the average score and standard deviation across the screens. Hit threshold is delineated by the vertical dashed red lines in the graphs; −0.15 and −0.35 for phenotype and motility scores, respectively.

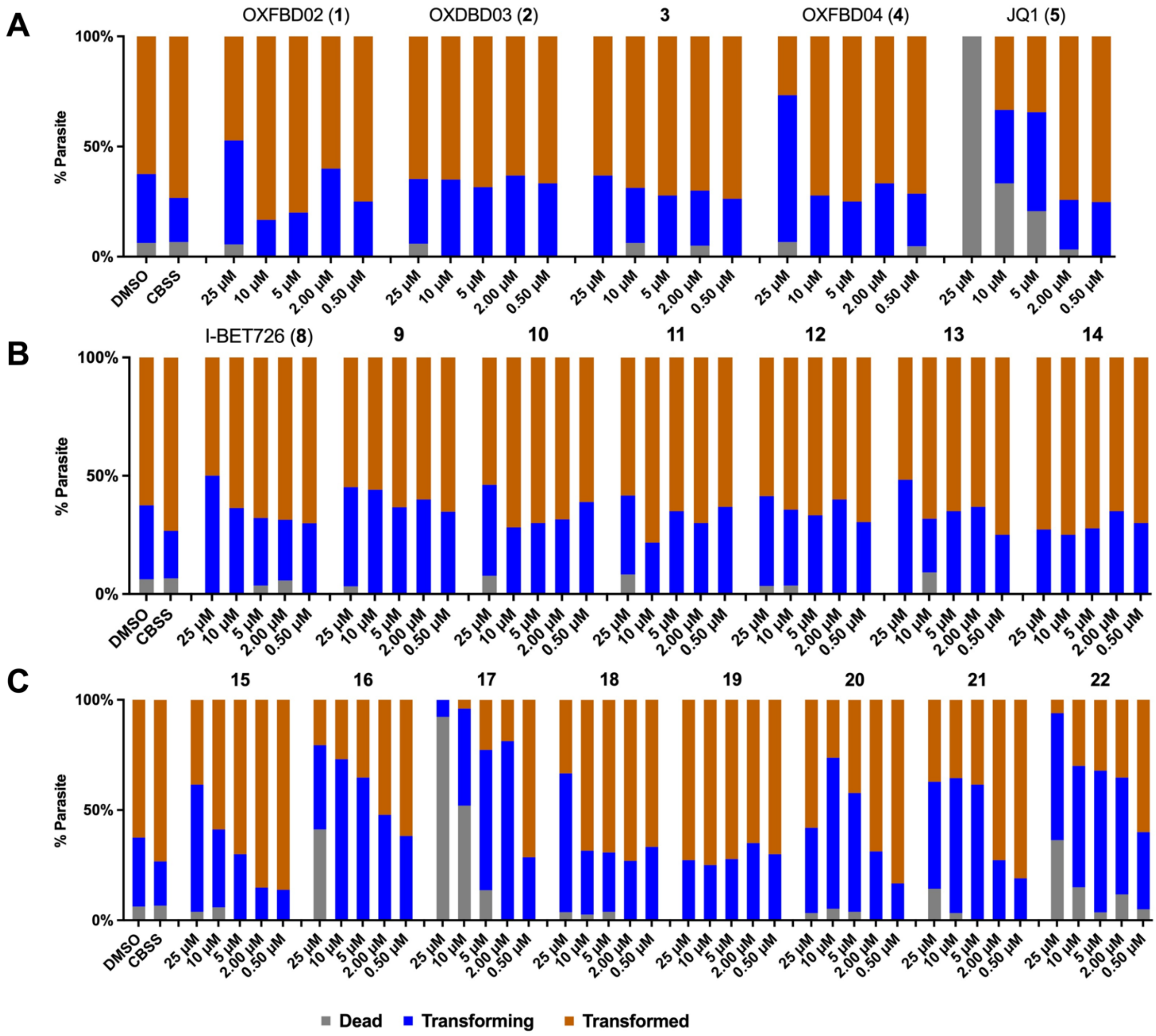

Figure S-49. Differential activity of compounds **1**–**5** (**A**), **8**–**14** (**B**), and **15**–**22** (**C**) on *ex vivo* miracidia to sporocyst transformation. Miracidia were exposed to the selected compounds in a dose response titration (CBSS containing 25, 10, 5, 2, or 0.5 µM in 1% DMSO). Dead parasites (in grey), transforming miracidia (in blue) and fully transformed sporocysts (in brown) were enumerated after 48 h (scored as percentage of parasite population - % Parasite). Each titration point was measured in triplicate and compared to parasites cultured in CBSS with 1% DMSO (controls) at a constant temperature of 26 °C, in the dark.

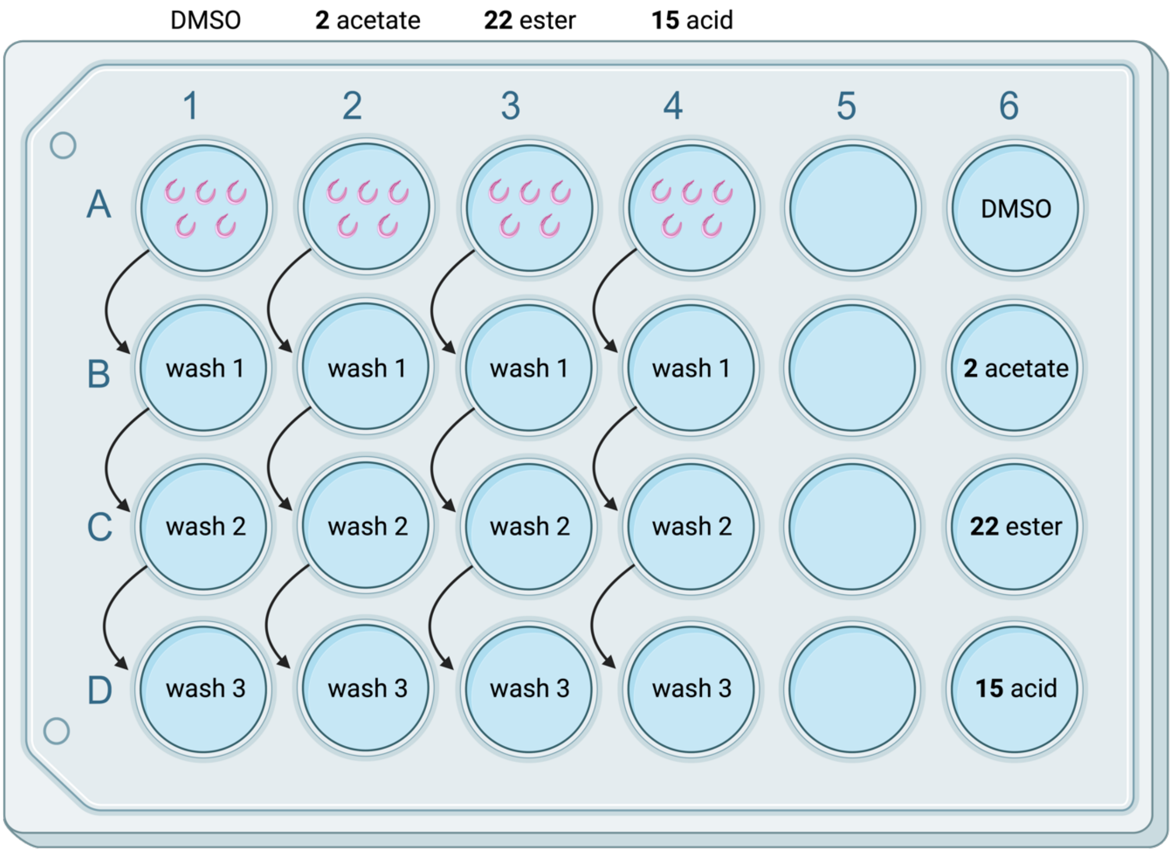

Figure S-50. Graphical display of the experimental set up for the compound accumulation assay: 5 male worms per treatment were incubated with 20 μM of **1**, **2**, **15,** or **22**, in 0.2% DMSO or 0.2% DMSO only, as indicated, in 2 mL of media for 24 hours. Wells containing media and compound only (no worms) were also incubated. Following incubation, the worms were washed 3 times (by sequential transfer) in wells containing 2 mL of fresh media only (no compound). Worms were then transferred to a 1.5 mL Eppendorf and frozen. 1 mL aliquots of the media from all wells were also collected.

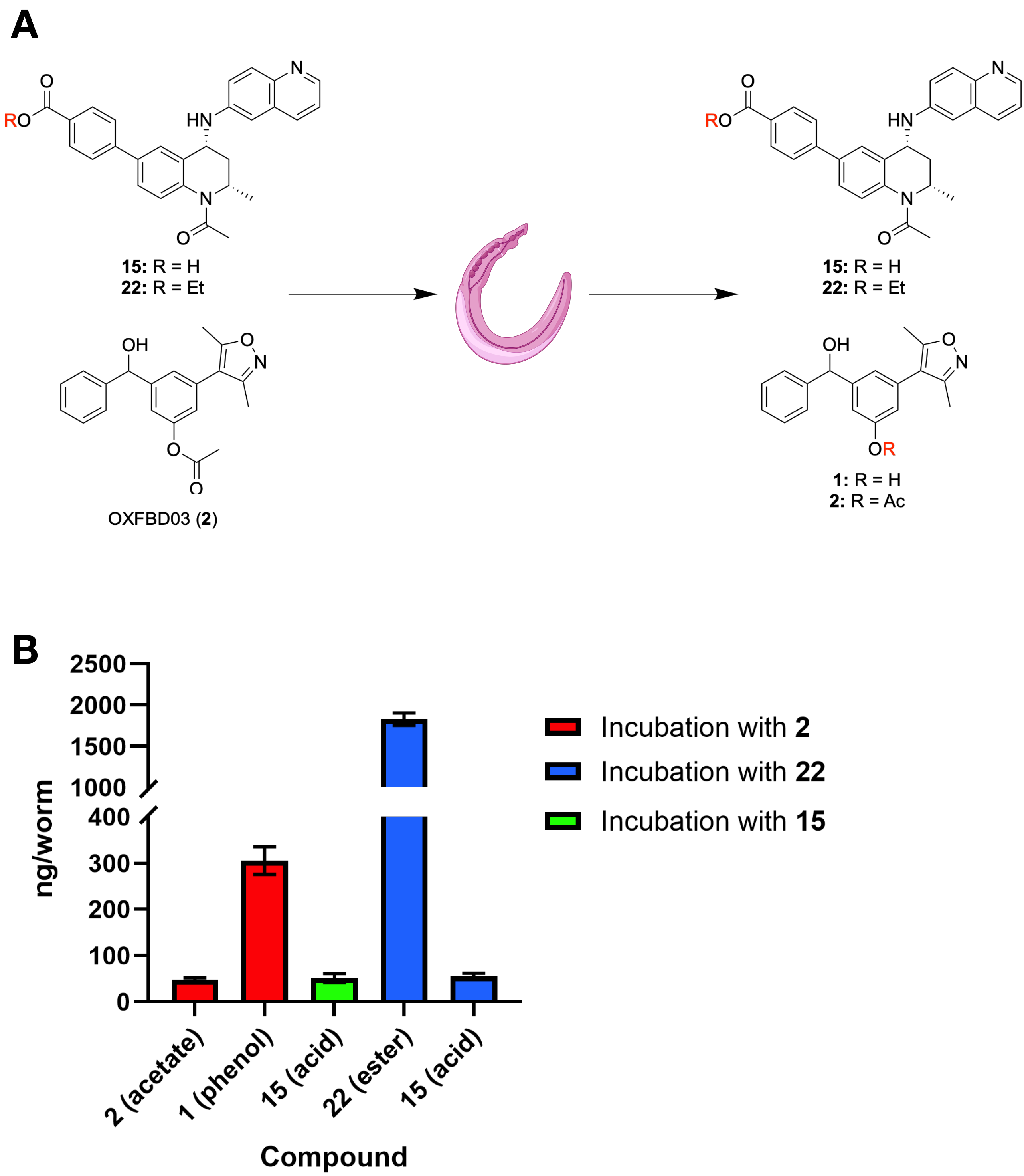

Figure S-51A. Schematic representation of the worm permeability experiments conducted. **B**. Estimation of the amounts of compounds **1, 2**, **15** or **22** found in the lysate of adult male *S. mansoni* lysate following 24 h incubation with compounds **2** (red), **15** (green) and **22** (blue), using the experimental set up shown in Figure S50. The estimated amounts were calculated as a mean (n = 3) from the analysis of lysate from male adult worms, 5 worms per well, with error bars representing the s.e.m.

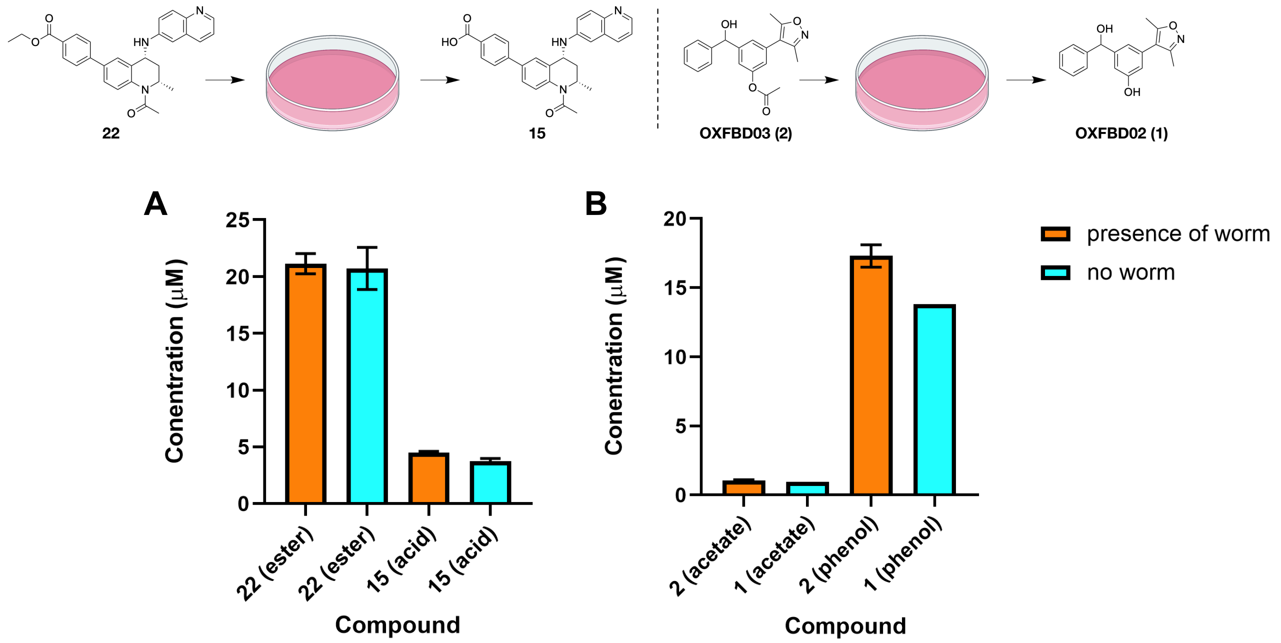

Figure S-52. A. Estimation of the concentration of **15** and **22** in media after incubation with **22** in the presence and absence of *S. mansoni*. The concentration estimations were calculated as an average (presence of worm n=3, no worm n=2) from the analysis media in the presence (5 worms per well) or absence of worms, with error bars representing the s.e.m. **B**. Estimation of the concentration of **1** and **2** in media after incubation with **2** in the presence and absence of *S. mansoni*. The concentration estimations were calculated as an average (presence of worm n=2, no worm n=1) from the analysis of media in the presence (5 worms per well) or absence of worms with error bars representing the s.e.m.

Table S-4. Aqueous solubility in phosphate buffered saline at pH 7.4 and chromLogD values for compounds **9**, **14**, **16** and **22.** Details of the solubility and chromLog D assays can be found in the General Chemistry Experimental.

Compound	Structure	Aqueous Solubility by CAD (µM)	chromLogD (pH 7.4)
**9**	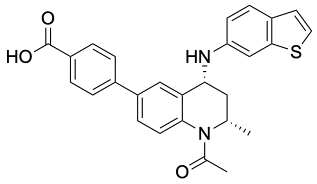	**≥259**	**2.83**
**14**	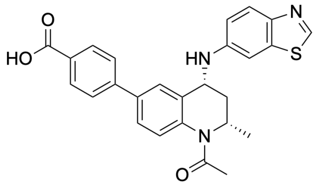	**≥260**	**1.78**
**17**	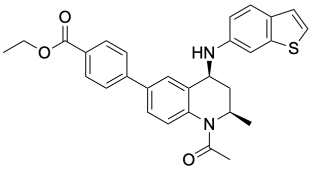	**1**	**7.61**
**22**	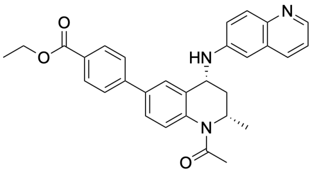	**13**	**5.95**

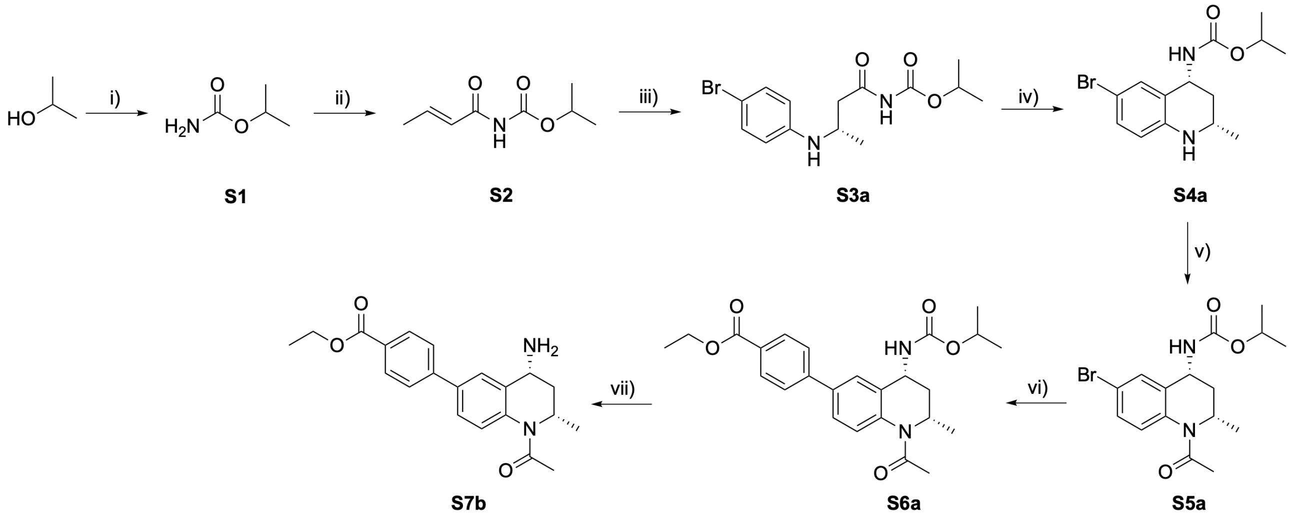

Scheme S-1. Synthesis of primary amine intermediate **S7a**, described by Gosmini *et al.*[3] and using work form Shadrick *et al.*[4] *Reagents and conditions:* i) TFA, ^{i}PrOH, NaOCN, rt, 79%; ii) Crotonoyl chloride, LiHMDS, THF, −78 °C, 61%; iii) (*R*)-BINAP(OTf)$_2$(H$_2$O)$_2$Pd, 4-bromoaniline, toluene, rt, 95%; iv) NaBH$_4$, EtOH, MgCl$_2$·6H$_2$O, H$_2$O, <0 °C to rt, 98%; v) Pyridine, AcCl, CH$_2$Cl$_2$, 90%; vi) {4-[(Ethyloxy)carbonyl]-phenyl}boronic acid, Pd(Ph$_3$)$_4$, Na$_2$CO$_{3(aq)}$, DME, 105 °C, 91%; vii) AlCl$_3$, CH$_2$Cl$_2$, 0 °C, 84%.

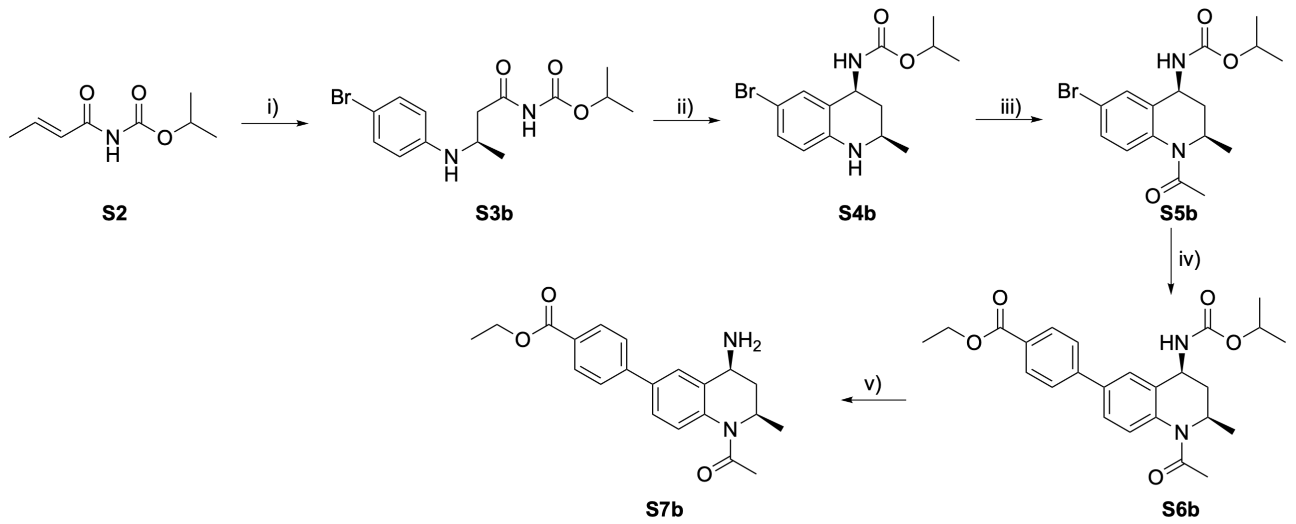

Scheme S-2. Synthesis of primary amine intermediate **S7b**. *Reagents and conditions:* i) (*S*)-BINAP(OTf)$_2$(H$_2$O)$_2$Pd, 4-bromoaniline, toluene, rt, 90%; ii) NaBH$_4$, EtOH, MgCl$_2$·6H$_2$O, H$_2$O, <−10 °C, 93%; iii) Pyridine, AcCl, CH$_2$Cl$_2$, 88%; iv) {4-[(Ethyloxy)carbonyl]-phenyl}boronic acid, Pd(Ph$_3$)$_4$, Na$_2$CO$_{3(aq)}$, DME, 105 °C, 80%; v) AlCl$_3$, CH$_2$Cl$_2$, 0 °C, 82%.

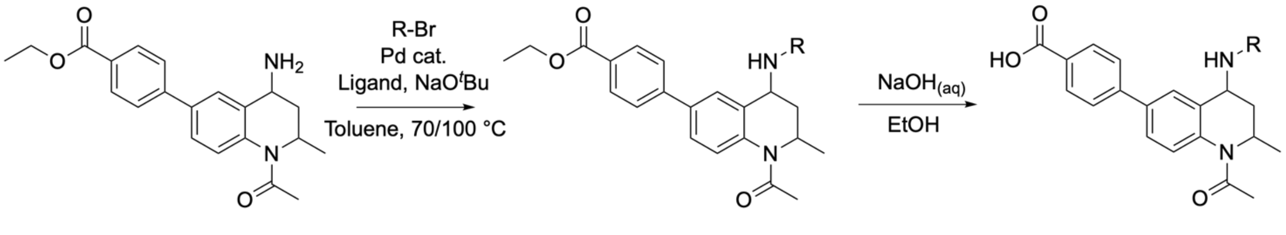

Scheme S-3. General synthetic scheme for compounds **9–12, 14–19** and **21–22**; The ligand, R-Br, catalyst, ligand, temperature and yields for the synthesis of compounds **16–19**, **21** and **22** are shown in Table S5**.** Synthesis of **9–12**, **14** and **15**. *Reagents and conditions*: $NaOH_{(aq)}$, EtOH, rt, 20–87%.

Table S-5. The ligand, R-Br, catalyst, ligand, temperature and yields for the syntheses of compounds **16–19**, **21** and **22.** NaO^{t}Bu was used as the base for all reactions for the syntheses of **16–19**, **21** and **22.**

Compound	R-Br	Pd Cat.	Ligand	Temperature (°C)	Yield (%)
**16**	6-Bromobenzothiophene	BrettPhos Pd G3	BrettPhos	70	87
**17**	6-Bromobenzotiophene	BrettPhos Pd G3	BrettPhos	70	69
**18**	6-Bromobenzofuran	BrettPhos Pd G3	BrettPhos	70	68
**19**	5-Bromobenzothiophene	BrettPhos Pd G3	BrettPhos	70	66
**21**	6-Bromobenzothiazole	BrettPhos Pd G3	BrettPhos	70	48
**22**	6-Bromoquinoline	$Pd(OAc)_2$	CyJohnPhos	100	30

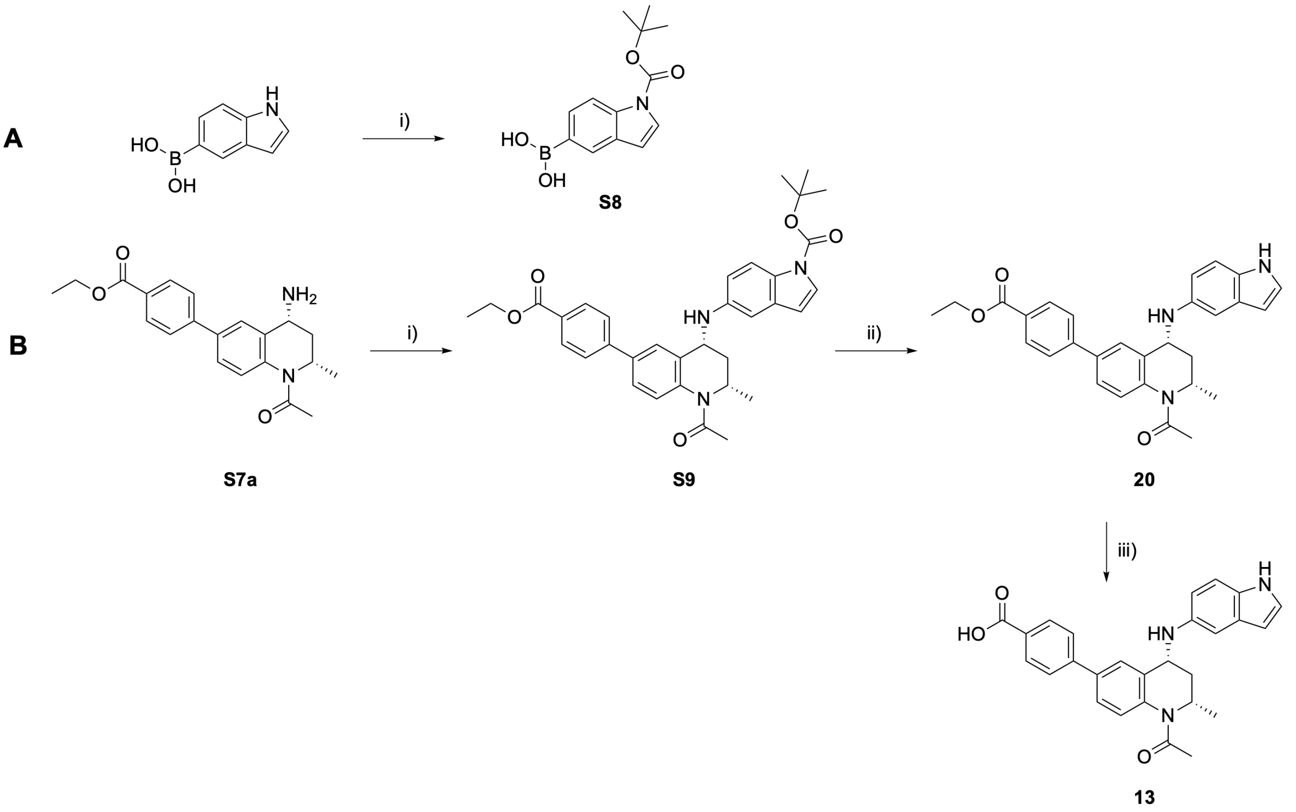

Scheme S-4. A. Synthesis of intermediate **S8**. *Reagents and conditions*: i) $(Boc)_2O$, DMAP, MeCN, rt, 49%; **B.** Synthesis of compounds **20** and **13**. *Reagents and conditions*: **S8**, $Cu(OAc)_2$, NEt_3, CH_2Cl_2, 3 Å molecular sieves, rt, 53%; ii) TFA, CH_2Cl_2, rt, 46%; iii) $NaOH_{(aq)}$, EtOH, 32%.

General biology experimental and biology methods

Biologically tested compounds: compounds **9-22** were synthesised by Darius McArdle using procedures outlined above. OXFBD02 (**1**), OXFBD03 (**2**), OXFBD02-F (**3**) and OXFBD04 (**4**) were synthesised by Lily-Latimer Smith, Oliver Stratton, Charles Evans, and Darius McArdle, respectively, using previously published procedures.[5,6] I-BET726 (**8**) was purchased from Cambridge Bioscience Ltd, BI-2536 (**7**) and I-BET151 (**6**) from MedChemExpress and (+)-JQ1 (**5**) from Stratech Scientific Ltd. The Maybridge Fragment library was obtained from Fisher Scientific. We wish to thank the EPSRC funded network, PPI-net (EP/I037210/1 and EP/I037172/1) and GSK for making the PPI-net screening collection available.

All other biological materials were obtained from commercial sources.

All biological solutions are aqueous, unless otherwise stated.

Purified (Milli-Q) water: obtained from a Millipore Elix® Reverse Osmosis system which was further purified by a Millipore Milli-Q®. Synthesis system with a 0.22 µm filter on the outlet. Reagent quantities given as weight per volume (*w/v*) correspond to g/mL unless otherwise stated.

pH measurements: performed using an VWR pH 100 pH meter or a Jenway pH meter 3305, with an Aldrich glass/calomel combination electrode or an Oakton PH 550 Benchtop pH Meter Kit, with an Oakton pH electrode with temperature sensor. Calibration was carried out between pH 4.0–7.0 or 7.0–10.0 immediately before use, with buffer solutions of phthalate (pH 4.0), phosphate (pH 7.0) and borate (pH 10.0) from Fischer Scientific, or using pH 4.0, pH 7.0, pH 10.0 buffer solutions from Cole-Palmer. Electrodes were stored in a 4 M aqueous potassium chloride solution.

Protein concentration was estimated by measuring the absorbance at 280 nm using a Nanodrop® ND-1000 spectrophotometer (Nanodrop® Technologies Inc.) with the 'Protein A280' program module according to the manufacturer's instructions, or NanoDrop Lite spectrophotometer (Nanodrop® Technologies Inc.), using the Beer-Lambert law and corresponding extinction coefficients.

Protein molecular weight and molar extinction coefficient were calculated using ProtParam on the ExPASy Bioinformatics Resource Portal.

Centrifugation: Protein samples of up to 10.0 mL were centrifuged in a Beckman Allegra-X30R Centrifuge (3082 ×g) at 4 °C. Cell growth media and cell lysates were centrifuged in a Beckman Coulter J-25 (11,325 ×*g*: JSP F500 rotor; 9605 ×*g*: JLA-16.250 rotor and 7741 ×*g*: JA-25.50 rotor, respectively).

Gibson Assembly (GA):

Cloning was performed using a one-step isothermal *in vitro* recombination reaction consisting of T5 exonuclease, Phusion DNA polymerase and Taq DNA ligase. Linear vectors (~0.03 pmol) and gene inserts (~0.1 pmol) with complementary regions were incubated at 50 °C for 15-60 min in a 1:3 ratio with Gibson Assembly master mix (NEB): 20 µL reaction volume, 10 µL 2 × GA master mix. 2 µL of

the reaction mixture was used directly to transform NEB 5-alpha (#C2987) competent cells, or diluted 1:3 to transform BL21 (DE3) Gold competent cells.

Competent cells: *Escherichia coli* (*E. coli*) strains of the following genotypes were used:

- BL21 (DE3) Gold: F- ompT hsdSB (rB-mB-) gal dcm (DE3). DE3 denotes a chromosomal copy of the T7 RNA polymerase gene.

Competent cells were thawed on ice for 20 min before 1-5 µL of plasmid DNA (from an 80–140 ng/µL stock solution) was added to 20 µL of the competent cells in pre-chilled 1.5 mL Eppendorf tubes. The tubes were left on ice for 30 min before incubation at 42 °C for 30 sec. The tubes were returned to ice for 5 min, 500 µL of 2-TY media was added gently to each tube, which was then incubated at 37 °C for 1 h before 100-200 µL of the transformation mixture was plated onto agar plates containing kanamycin (50 µg/mL) and incubated overnight.

Fermentation and purification conditions for bromodomain containing proteins

For the expression and purification of the human and schistosomal bromodomain containing proteins we followed a previously published procedure.[7] Single colonies of transformed *E. coli* BL21(DE3) were transferred under aseptic conditions to 100 mL of 2× TY medium containing a final concentration of 50 µg/mL of kanamycin. The cultures were incubated overnight at 37 °C and 180 rpm. 10 mL of the overnight cultures were added to 1 L of 2× TY medium containing a final concentration of 50 µg/mL of kanamycin. The cultures were incubated overnight at 37 °C and 180 rpm. When the OD_{600} reached a value of 0.6, the cultures were cooled down to 16 °C and IPTG was added to the culture at a final concentration of 0.1 mM. OD_{600} values were measured in 1.6 mL cuvettes against a reference of 2× TY medium, using a Novaspec® II spectrometer measuring at 600 nm. Subsequently, the cells were fermented at 16 °C, 180 rpm, overnight. Cells were harvested by centrifugation (8000 rpm, 4 °C, 10 min). The cell pellet was re-suspended in Extraction buffer (100 mL per 20 g cell mass), 25 Units (U) of Benzoase was added, then the cells were lysed by sonication using thirty 5 sec bursts interrupted by 5 to 10 sec pauses. Polyethyleneimine (PEI, 0.15% *v/v*) was added, and the cells were incubated on ice for 15 min. The lysate was clarified using centrifugation and the supernatant decanted, filtered through a 0.45 µm filter, and applied to a purification column. Immobilised metal affinity chromatography (IMAC) was performed with a HisTrap™ (5 mL) column (GE Healthcare) charged with 4 CV of 100 mM $NiSO_4$ and washed with 10 CV of binding buffer prior to loading the cell lysate at 1 mL/min. The column was then washed with approximately 20 CV binding buffer. When all residual products of bacterial fermentation were eluted from the column by binding buffer, the protein of interest was batch-eluted with elution buffer (step gradient elution, 5 – 500 mM imidazole). Eluted protein was collected and fractionated; fractions containing the highest levels of pure protein (as determined by SDS-PAGE gels and UV trace) were concentrated using 5k or 10k concentrators (GE Healthcare) in a Beckman Allegra™ 21R centrifuge. Further purification was achieved by gel filtration chromatography. Concentrated fractions (<2 mL)

were loaded on a 120/300 mL Superdex 75 size exclusion chromatography column (Amersham). Eluted protein was collected and fractionated; fractions containing the highest levels of pure protein (as determined by SDS-PAGE gels) were concentrated as described before.

Table S-6. Components of 2× TY media

Buffer	Reagent	Mass per litre (gL^{-1})
2× TY	Trypton	16.0
	Yeast Extract	10.0
	NaCl	5.0

Table S-7. Buffer type and composition for purification of H6-*Sm*BRD3(1,2) and H6-*Sm*BRD3(2).

Buffer	Reagent	Concentration
Binding buffer (pH = 7.6)	HEPES	50 mM
	NaCl	500 mM
	Imidazole	5 mM
Extraction buffer (pH = 7.6)	HEPES	50 mM
	NaCl	500 mM
	Imidazole	5 mM
	Sigma*FAST*[a]	1 Tbl/20 g pellet
Elution buffer (pH = 7.6)	HEPES	50 mM
	NaCl	500 mM
	Imidazole	500 mM
Strip buffer (pH = 7.6)	HEPES	50 mM
	NaCl	500 mM
	EDTA	100 mM
Gel filtration buffer (pH = 7.6)	HEPES	50 mM
	NaCl	500 mM

[a]SigmaFAST Protease Inhibitor Cocktail Tablet, EDTA Free (Sigma-Aldrich)

Fermentation and purification conditions for TEV protease pRK793

The plasmid for pRK793 was kindly provided by the group of Prof. Christopher Schofield, University of Oxford, as a glycerol stock of transfected *E. coli* BL21 (DE3) cells. Single colonies of transformed *E. coli* BL21(DE3) were transferred under aseptic conditions to 100 mL of 2× TY medium containing a final concentration of 50 µg/mL of kanamycin. The cultures were incubated overnight at 37 °C and 180 rpm. 10 mL of the overnight cultures were added to 1 L of 2× TY medium containing a final concentration of 50 µg/mL of Kanamycin. The cultures were incubated overnight at 37 °C and 180 rpm. When OD_{600} reached a value of 0.7, the cultures were cooled to 18 °C, and IPTG was added to the culture at a final concentration of 0.5 mM. Subsequently the cells were fermented at 18 °C, 180 rpm, overnight. Cells were harvested by centrifugation (8000 rpm, 4 °C, 10 min). The cell pellet was lysed by sonication according to our standard lab protocols and the lysate was loaded on a pre-equilibrated 5 mL Ni^{2+}-NTA column. The protein was eluted during a gradient elution from 5 mM to 500 mM of imidazole. Protein containing fractions were combined and concentrated using a Pierce™ Protein Concentrator PES (10k MWCO, 5-20 mL). 2 mL of the concentrated sample was injected in a S200 130 mL gel filtration column. Purity of the fractions was monitored using SDS-PAGE and pure fractions were combined and concentrated using a PierceTM Protein Concentrator PES (10 K MWCO, 5-20 mL) to a concentration of approximately 15 mg/mL. Glycerol was added to a final concentration of 15% prior to snap freezing the samples using liquid nitrogen.

Table S-8. Buffer type and composition for purification of TEV protease pRK793.

Buffer	Reagent	Concentration
Binding buffer (pH = 7.6)	HEPES	25 mM
	NaCl	500 mM
	Glycerol	5% (*v/v*)
	Imidazole	5 mM
	TCEP	0.5 mM
Gel filtration buffer (pH = 7.6)	HEPES	25 mM
	NaCl	500 mM
	Glycerol	5% (*v/v*)
	TCEP	0.5 mM
Elution buffer (pH = 7.6)	HEPES	25 mM
	NaCl	500 mM
	Glycerol	5% (*v/v*)
	Imidazole	500 mM
	TCEP	0.5 mM

Purification conditions for *Sm*BRD3 bromodomain for crystallisation trials

Fermentation and cell lysis was performed as described above for the other bromodomain containing proteins. Immobilised metal affinity chromatography (IMAC) was performed with a HisTrap™ (5 mL) column (GE Healthcare) charged with 4 CV of 100 mM $NiSO_4$ and washed with 10 CV of binding buffer prior to loading the cell lysate at 1 mL/min. The column was then washed with approximately 20 CV binding buffer. When all residual products of bacterial fermentation were eluted from the column by binding buffer, the protein of interest was batch-eluted with elution buffer (step gradient elution, 5 – 500 mM imidazole). Eluted protein was collected and fractionated; fractions containing the highest levels of pure protein (as determined using SDS-PAGE gels and UV traces) were concentrated using 5k spin-concentrators (GE Healthcare) in a Beckman Allegra™ 21R centrifuge to approximately 5 mL. 15 mL of TEV cleavage buffer (50 mM HEPES, 500 mM NaCl, 1 mM β-mercaptoethanol, pH 7.6) were added and the mixture was again concentrated to approximately 5 mL using 5k concentrators (GE Healthcare) in a Beckman Allegra™ 21R centrifuge. After determination of the concentration of H6-SmBRD3(2), TEV protease (pRK793) was added in a molar ratio of 2:1 (H6-SmBRD3(2)/TEV protease). TEV cleavage buffer was added to give a total volume 20 mL. The mixture was incubated overnight at 4 °C. After sufficient digestion of the substrate protein (monitored using SDS-PAGE), the mixture was concentrated to approximately 2 mL using 5k concentrators (GE Healthcare) in a Beckman Allegra™ 21R centrifuge. To separate cleaved substrate protein (*Sm*BRD3(2)) from the H6-tag that has been cleaved off and the H6-tagged TEV protease, a second immobilised metal affinity chromatography (IMAC) was performed with a HisTrap™ (5 mL) column (GE Healthcare) charged with 4 CV of 100 mM $NiSO_4$ and washed with 10 CV of binding buffer prior to loading the cell lysate at 1 mL/min. The column was then washed with approximately 20 CV binding buffer. *Sm*BRD3(2) was detected in the flow-through and H6-tagged components were subsequently eluted with elution buffer (step gradient elution, 5 – 500 mM imidazole). Eluted protein was collected and fractionated; fractions containing the highest levels of pure protein (as determined by SDS-PAGE gels and UV trace) were concentrated using 5k concentrators (GE Healthcare) in a Beckman Allegra™ 21R centrifuge to approximately 2 mL. Further purification was achieved using gel filtration chromatography. Concentrated fractions (<2 mL) were loaded on a 120/300 mL Superdex 75 size exclusion chromatography column (Amersham). Please note, for this gel filtration we used a modified gel filtration buffer with a reduced amount of NaCl (see table below). Eluted protein was collected and fractionated; fractions containing the highest levels of pure protein (as determined by SDS-PAGE gels) were concentrated to a final concentration of 12.31 mg/mL and 24.89 mg/mL, respectively.

Table S-9. Buffer type and composition for purification of *Sm*BRD3(2).

Buffer	Reagent	Concentration
Binding buffer (pH = 7.6)	HEPES	50 mM
	NaCl	500 mM
	Imidazole	5 mM
Extraction buffer (pH = 7.6)	HEPES	50 mM
	NaCl	500 mM
	Imidazole	5 mM
	Sigma*FAST*[a]	1 Tbl/20 g pellet
Elution buffer (pH = 7.6)	HEPES	50 mM
	NaCl	500 mM
	Imidazole	500 mM
Strip buffer (pH = 7.6)	HEPES	50 mM
	NaCl	500 mM
	EDTA	100 mM
Gel filtration buffer (pH = 7.6)	HEPES	50 mM
	NaCl	150 mM

[a]SigmaFAST Protease Inhibitor Cocktail Tablet, EDTA Free

Sodium dodecyl sulfate polyacrylamide gel electrophoresis (SDS-PAGE)

Samples obtained from milli-scale expression were analysed by SDS-PAGE. Gels were prepared using 70 mm × 100 mm glass plates, with 0.75 mm spacers. Tetramethylethylenediamine (TEMED) and freshly prepared ammonium persulfate were added just prior to pouring the gels. The resolving gel was cast with the addition of a separate layer of isopropyl alcohol (to ensure a level surface). Once the resolving layer had polymerised, isopropyl alcohol was removed, and the stacking gel was cast. For protein denaturation samples were prepared by mixing with sample loading buffer (4×) and incubation at 100 °C for 10 min. All gels were loaded with one of the following molecular weight ladders: blue protein standard, broad range (NEB); colour protein standard, broad range (NEB). Gels were run on a mini-PROTEAN Tetra Electrophoresis System (Bio-Rad) at a constant potential of 100 V. Following electrophoresis, gels were stained for 30 min with InstantBlue™ Coomasie® stain (Expedeon) and afterwards destained for 5 h.

Table S-10. Gel compositions (quantities for 1 gel) for SDS-PAGE.

Reagent	Resolving gel (16%)	Stacking gel (4%)
Tris-HCl (1.5 M, pH 8.8)	1.25 mL	-
Tris-HCl (0.5 M, pH 6.8)	-	0.65 mL
Milli-Q water	1.05 mL	1.45 mL
30 % (*w/v*) Acrylamide	2.65 mL	0.35 mL
SDS (10% *w/v*)	50 µL	25 µL
10% (*w/v*) APS	50 µL	25 µL
TEMED	4 µL	2.5 µL

Table S-11. Buffer composition for SDS-PAGE.

Buffer	Reagent	Amount required per litre
SDS-PAGE running buffer	Tris-HCl	30 g
	Glycine	144 g
	SDS	10 g
SDS-PAGE stain	Coomassie® brilliant blue	2.5 mL
	acetic acid	100 mL
	methanol	300 mL
SDS-PAGE destain	acetic acid	100 mL
	methanol	400 mL

Differential scanning fluorimetry assay

The differential scanning fluorimetry (DSF) or thermal stability assays were carried out following the protocol described by Niesen *et al*. and data were analysed as described previously.[8] Thermal melting experiments were carried out using the Mx3005p real-time PCR machine (Agilent) and employing a protein concentration of 5 μM for the schistosomal bromodomains [*Sm*BRD3(2), *Sm*BRD3(1,2)]. If not otherwise stated, inhibitors were adjusted to a concentration of 150 μM and the DMSO content was 0.5% (*v/v*). Buffer conditions were 50 mM HEPES buffer, pH 7.5, 500 mM NaCl and a 1:500 dilution of SYPRO® Orange (Invitrogen, CA) was used. The 96-well PCR plates were sealed and centrifuged for 2 min at 1000 rpm at 25 °C before measurement. The temperature was raised with a step of 1 °C per 30 s from 25 °C to 85 °C, and fluorescence readings were taken at each interval. Excitation and emission filters for the SYPRO® Orange dye were set to 465 and 590 nm, respectively. The observed temperature shifts, ΔT_m, for each inhibitor was recorded as the difference between the transition midpoints of sample and reference wells containing protein without inhibitor in the same plate. Reported ΔT_m values are the mean of two replicates.

Isothermal Titration Calorimetry

Isothermal titration calorimetry experiments were performed on a MicroCal PEAQ-ITC Automated or Manual (Malvern) and analysed with the MicroCal PEAQ-ITC Analysis software (Malvern 1.1.0.1262) using a single binding site model. For each experiment, the first data point was removed from the analysis. The proteins were dialysed at 4 °C overnight in a Slide-A-LyzerR MINI Dialysis Device (2000 MWCO; Thermo Scientific Life Technologies) into 50 mM HEPES, 150 mM NaCl containing 0.2% DMSO; pH 7.4. Proteins were centrifuged to remove aggregates (3 min, 3000 rpm, 25 °C). Compounds were dissolved as a DMSO stock solution and diluted to the required concentration using dialysis buffer (DMSO free) and to a final DMSO concentration of 0.2% (unless otherwise specified). The cell was stirred at 750 rpm, reference power set to 5 μcal/s and temperature held at 25 °C. After an initial delay of 60 s, 19 x 2 μL injections (first injection 0.4 μL) were performed with a spacing of 180 s. Small molecule solutions in the calorimetric cell (250 μL for manual, 400 μL for auto, 10 to 30 μM) were titrated with the protein solutions in the syringe (40 μL for manual, 120 μL for auto, 100 to 300 μM).

Determination of compound parasite permeability

Male adult schistosomes were seeded into 24-well tissue culture plates (5 worms/well), dosed with compounds (20 μM in 0.2% DMSO *v/v*) and incubated for 24 h at 37 °C in 5% CO_2. Following the incubation period, parasites were washed three times by sequentially transferring the worms to 2 mL of fresh media lacking any compound treatment. Worms were blotted dry to remove excess media between transfers. Parasites were then snap frozen and stored at −80 °C. When ready for processing, worms were removed from the −80 °C and subject to 3 rounds of repeated snap freeze:thawing. After the final thaw step a sterile 3 mm stainless steel bead was added to each tube

along with 200 µL HPLC grade MeOH and samples were homogenised with a TissueLyser (Qiagen) (3 × 3 min, 50 Hz). Finally, samples were centrifuged at 14,000 rpm for 30 min at 4 °C and the supernatant removed to a fresh tube for analysis. For the LCMS analysis of media samples, 1 mL of media was removed and lyophilised. The residue was then resuspended in 200 µL of HPLC grade MeOH and centrifuged at 14,000 rpm for 30 min at 4 °C. The supernatant was removed to a fresh tube for analysis.

Samples were analysed using a Q-Exactive Plus (Thermo Scientific) with an HESI II source coupled to a Dionex Ultimate 3000 Ultra High Performance Liquid Chromatography (UHPLC) system (Thermo Scientific). Chromatographic separation was performed on a Hypersil Gold 1.9 µm, 2.1 × 200 mm column (Thermo Scientific) using water 0.1 % formic acid (mobile phase A) and methanol 0.1% formic acid (mobile phase B) at a flow rate of 0.6 mL/min and column oven temperature at 60 °C. Each sample (5 µL) was analysed following a gradient of 0% B to 100 % B in 9 min. The flow was held at 100% B for 2.5 min and the column re-equilibrated for 1.5 min. Data were acquired in positive ESI mode. Each experiment consisted of a full scan (65-975 mass-to-charge ratio (*m/z*) at 140k resolution (AGC target 2×10^5 at 100 ms maximum injection time, IT) and MS^2 scans (*PRM multiplexed, 4* stepped, HCD normalised collision energies (nce) of 50 % and 17.5k Orbitrap mass resolution) using selected targeted mass properties for either positive or negative ionisation mode, with an AGC target of 2×10^5 and isochronous IT enabled over 13 min runtime (**Table S12**). The spray voltage was 3.5 kV for positive and 2.5 kV for negative ionisation modes. The temperatures of the ion transfer capillary and vaporiser were 350 °C and 360 °C respectively with sheath and auxiliary gas set at 30 and 15 arbitrary units, respectively. The data were acquired using Thermo Scientific Xcalibur version 4.2.47. Mass calibration was performed regularly according to the manufacturer's recommendations. Targets were quantified using quadratic models to 1) avoid pre-concentration and 2) account for low concentrated targets within a sample outside the inherent linear range. Data were extracted as absolute area of the quantified ion using Xcalibur peak integration for the target masses of the following transitions: **2.6**: Q1 452.1969 - Q3 145.05; OXFBD02 (**1.4**): Q1 296.1282 - Q3 148.08; **2.31**: Q1 480.2282 - Q3 145.05; OXFBD03 (**1.37**): Q1 338.1387 - Q3 235.10. Concentrations and peak area of compound standard solutions for a calibration curve were $\log_{10}$-tranformed and plotted as a two-point calibration curve. This was used to estimate compound concentration from samples.

Table S-12: Inclusion list for parallel reaction monitoring (PRM) on Q-Exactive Plus.

**Mass [*m/z*]**	**Formula [M]**	**Specie [*z*]**	**CS**	**Polarity**	**Start [min]**	**End [min]**	**(N)C**	**MS ID**	**Number**
452.19687	$C_{28}H_{25}N_3O_3$	+H	1	Positive	0.1	13.0	50	1	**15**
480.22817	$C_{30}H_{29}N_3O_3$	+H	1	Positive	0.1	13.0	50	2	**22**
296.12812	$C_{18}H_{17}NO_3$	+H	1	Positive	0.1	13.0	50	3	**1**
338.13868	$C_{20}H_{19}NO_4$	+H	1	Positive	0.1	13.0	50	4	**2**

CS = charge state; (N)CE = normalised collision energy

Crystallisation of *Sm*BRD3(2)

Initially, *Sm*BRD3(2) (241..368aa, 6xHis-tag cleaved) was co-crystallised with I-BET726 (**8**) by the sitting drop vapor diffusion method using high throughput (HT) crystallisation screening methods. All HT screens were performed in CrystalMation Intelli-Plate 96-3 low-profile plates (Hampton Research, HR3-119). Reservoirs and drops were dispensed using an Art Robbins Phoenix automatic liquid handler. Reservoirs contained 80 µL of sparse matrix precipitant solution and crystallisation drops (200 – 300 nL total volume) were placed in each of the three subwells; subwell 1, 200 nL protein : 100 nL well solution; subwell 2, 100 nL protein : 100 nL well solution ; subwell 3, 100 nL protein : 200 nL well solution. Plates were sealed using optically clear Xtra-Clear Advanced Polyolefin StarSeal (StarLab) seals and incubated at 19 °C. Three commercially available precipitant kits (JCSG-plus MD1-37 (Molecular Dynamics), Crystal Screen (HR2-110, 1-50, Hampton), and Crystal Screen 2 (HR2-112, 1-46, Hampton)) were screened for suitable crystallization conditions.

Crystals of the *Sm*BRD3(2):I-BET726 complex were grown by co-crystallisation in conditions containing 25% *w/v* PEG 3350, 0.1 M Bis-TRIS, pH 5.5 (sitting drop, protein to-well ratio 1:2) at a protein concentration of 12.31 mg/mL and a final I-BET726 (**8**) concentration of 2 mM added directly to the protein prior to crystallization from a 100 mM stock solution in DMSO. Crystals formed within 5 weeks (maximum crystal size 300 × 150 × 150 µm).

For *Sm*BRD3(2):**9** co-crystallisation, an optimisation screen based on the initial *Sm*BRD3(2):I-BET726 (**8**) complex crystallisation conditions were carried out using component ranges of 19% to 30% PEG 3350 and pH 5.5 to 6.9 0.1M Bis-TRIS.

The resultant crystals were cryoprotected by soaking crystals in reservoir solution diluted with 25% (*v/v*) glycerol before being flash cooled in liquid nitrogen.

Data collection and processing

Data were collected using single crystals at Diamond Light Source beamlines i24 (*Sm*BRD3(2):I-BET726 (**8**)) or i04-1 (*Sm*BRD3(2):**9**) equipped with Dectris Eiger R 4M or Pilatus 6M-F detectors, respectively. Diffraction data for *Sm*BRD3(2):I-BET726 (**8**) were processed using HKL2000[9] and data for *Sm*BRD3(2):**9** were autoprocessed by the beamlines autoprocessing pipeline using the DIALS strategy.[10] [2]Both crystals processed in Space Group $C222_1$ with similar unit cell constants (Table S13).

Structure solution and refinement

*Sm*BRD3(2) structures were solved by molecular replacement (MR) using PHASER[11] with a structure of mouse BRD4 as the search model (PDB ID 3JVL). Two molecules were identified in the asymmetric unit. Visual inspection of initial electron density maps indicated that each chain contained bound inhibitor. Inhibitor was fit to the density and iterative cycles of model fitting in COOT[12] and refinement using PHENIX[13] were carried out until the converging R_{work} and R_{free} no longer decreased. Data collection and refinement statistics for all structures can be found in Table S13.

Table S-13. Protein crystallography data collection and refinement statistics.

⌘Values in brackets indicate the outermost shell.
$R_{merge} = \sum_j \sum_h | I_{hj} - \langle I_h \rangle | / \sum_j \sum_h \langle I_h \rangle \times 100$.
$R_{work} = \sum ||Fobs| - |Fcalc|| / |Fobs| \times 100$.
R_{free}, based on 7.45% (*Sm*BRD3(2)-I-BET726 (**8**)), 4.59% (*Sm*BRD3(2)-**9**) of the total reflections.

Structure	*Sm*BRD3(2):I-BET726 (**8**)	*Sm*BRD3(2):**9**
PDB ID	7AMC	7AMH
**Data Collection**		
Radiation source	Synchrotron<br><br>Diamond Beamline I24	Synchrotron<br><br>Diamond Beamline I04-1
Detector	EIGER R 4M	Dectris Pilatus 6M-F
X-ray Wavelength (Å)	0.9786	0.9119
Resolution Range (Å)[*]	43.99-1.22 (1.25-1.22)	63.37-1.86 (1.90-1.86)[*]
Space Group	$C\ 2\ 2\ 2_1$	$C\ 2\ 2\ 2_1$
Unit Cell Dimensions<br><br>(*a* Å, *b* Å, *c* Å, α°, β°, γ°)	51.81 83.24 126.60 90.0 90.0 90.0	52.20 84.11 126.74 90.0 90.0 90.0
Total Number of Reflections Observed	1014641 (63122)[*]	276609 (10702)[*]
Number of Unique Reflections	81598 (5962)[*]	23711 (1146)[*]
Multiplicity⌘	12.4 (10.6)[*]	11.7 (9.3)[*]
Completeness (%)⌘	99.9 (99.9)[*]	99.9 (98.9)[*]
$I/\sigma(I)$	6.2 (1.1)[*]	6.8 (0.5)[*]
R_{merge} (%)	0.187 (1.950)[*]	0.227 (4.369)[*]
CC1/2⌘	0.986 (0.614)[*]	0.995 (0.470)[*]
**Refinement**		
R_{work} (%)	0.1604	0.2174
R_{free} (%)	0.1772	0.2538
RMS Deviation (Bonds/Angle)	0.009/1.032	0.012/0.889
Average *B* Factor (Å^2)	25.81	37.23
Wilson B Factor (Å^2)	15.7	28.1
Number of Water Molecules	337	210

Structures have been deposited in the RSCB as PDB ID 7AMC and 7AMH. We thank the staff at Diamond Light Source beamline i24 visit mx18069-64 and i04-1 visit mx23459-11 for providing beamtime.

NMR spectra of novel compounds

^{1}H NMR ethyl 4-{(2*S*,4*R*)-1-acetyl-4-[(1-benzothiophen-6-yl)amino]-2-methyl-1,2,3,4-tetrahydroquinolin-6-yl}benzoate (**16**)

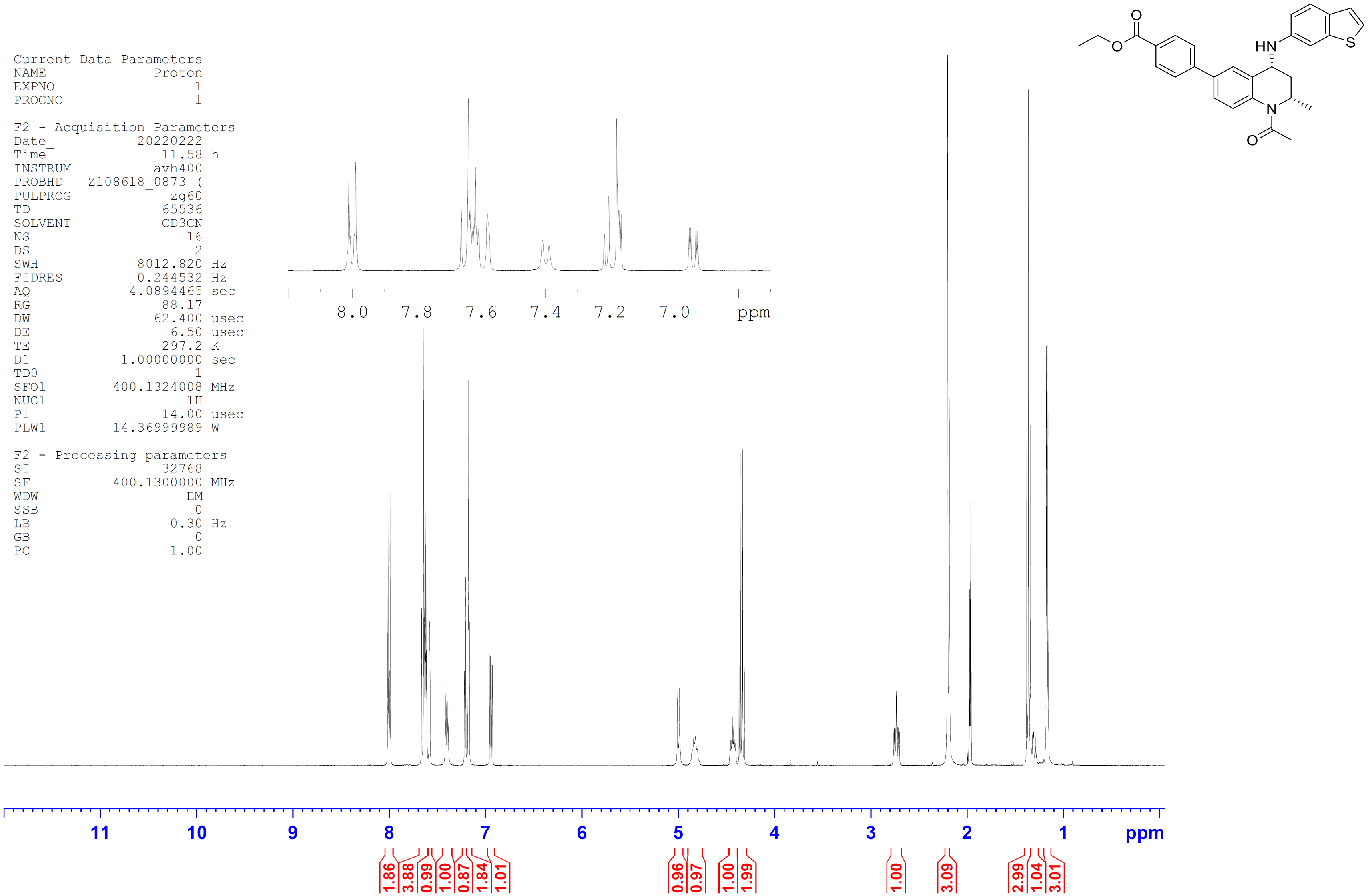

^{13}C NMR ethyl 4-{(2*S*,4*R*)-1-acetyl-4-[(1-benzothiophen-6-yl)amino]-2-methyl-1,2,3,4-tetrahydroquinolin-6-yl}benzoate (**16**)

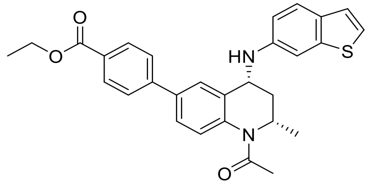

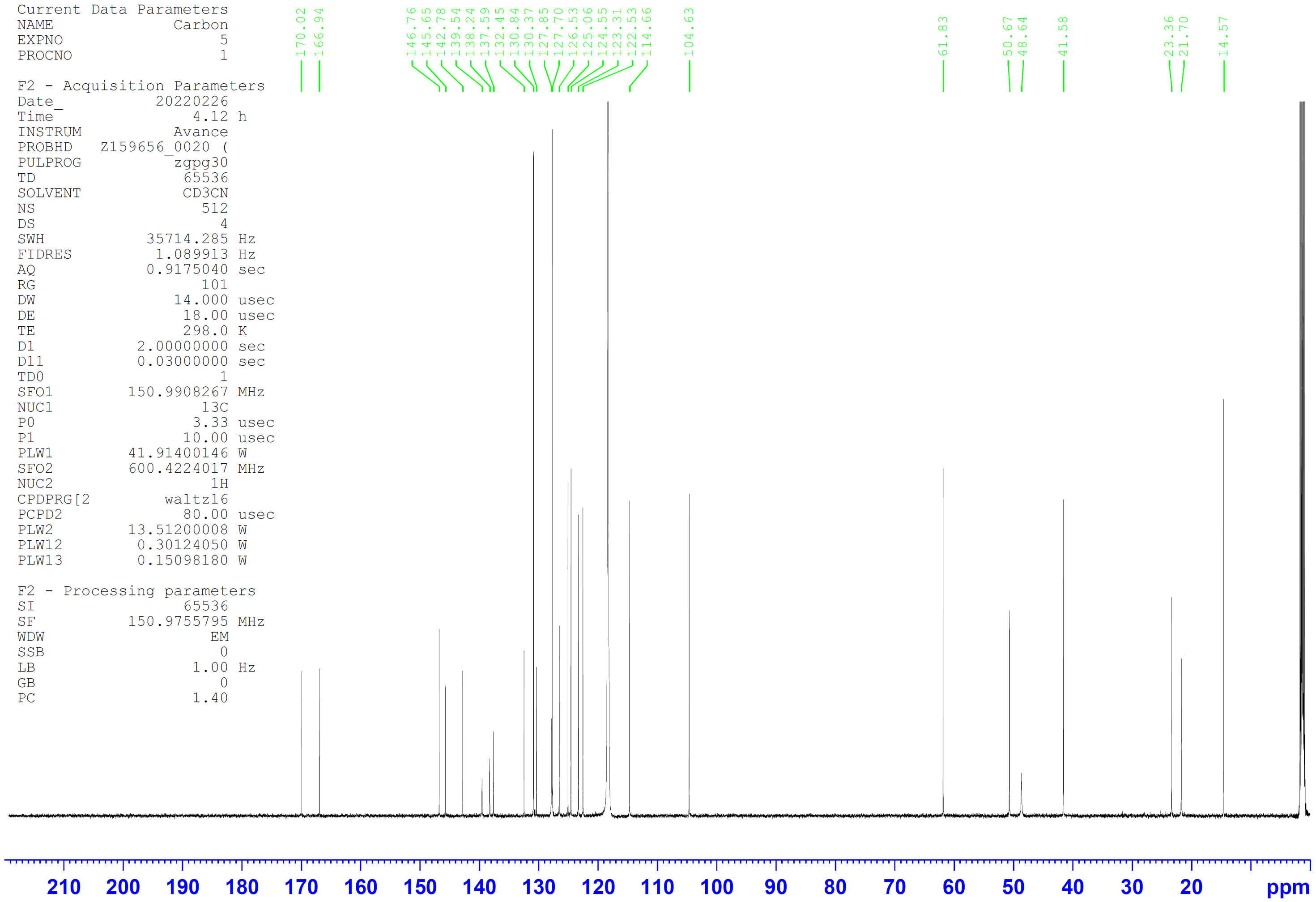

^{1}H NMR 4-{(2*S*,4*R*)-1-acetyl-4-[(1-benzothiophen-6-yl)amino]-2-methyl-1,2,3,4-tetrahydroquinolin-6-yl}benzoic acid (**9**)

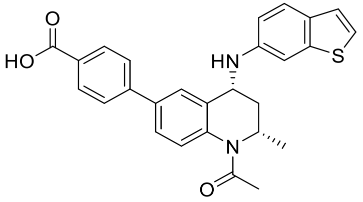


```
Current Data Parameters
NAME             Proton
EXPNO                 1
PROCNO                1

F2 - Acquisition Parameters
Date_          20220308
Time_             10.11 h
INSTRUM          avh400
PROBHD   Z108618_0873 (
PULPROG            zg60
TD                65536
SOLVENT            CD3CN
NS                   16
DS                    2
SWH            8012.820 Hz
FIDRES         0.244532 Hz
AQ            4.0894465 sec
RG                88.17
DW               62.400 usec
DE                 6.50 usec
TE                296.9 K
D1           1.00000000 sec
TD0                   1
SFO1        400.1324008 MHz
NUC1                 1H
P1                14.00 usec
PLW1        14.36999989 W

F2 - Processing parameters
SI                32768
SF          400.1300113 MHz
WDW                  EM
SSB                   0
LB                 0.30 Hz
GB                    0
PC                 1.00
```

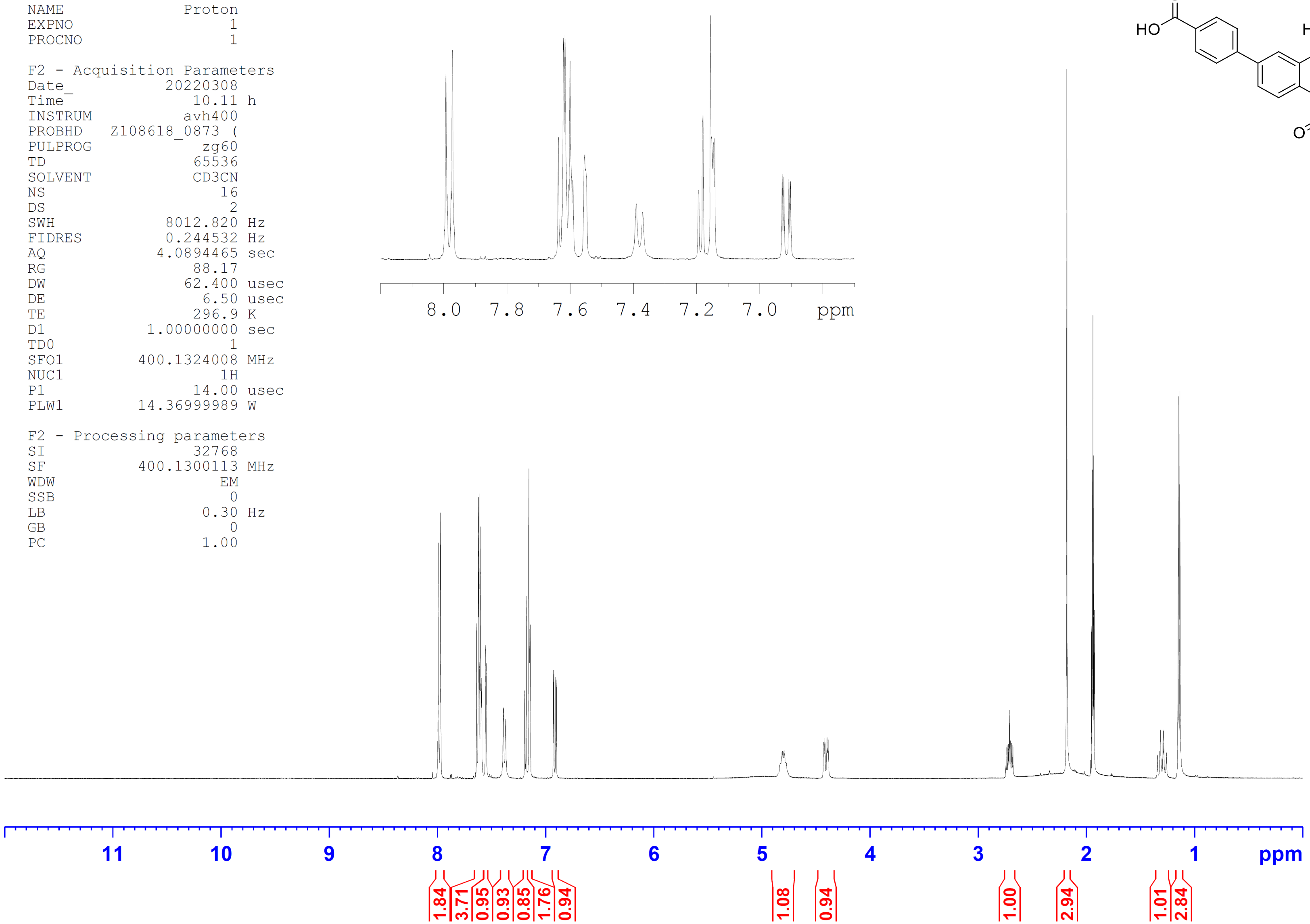

^{13}C NMR 4-{(2*S*,4*R*)-1-acetyl-4-[(1-benzothiophen-6-yl)amino]-2-methyl-1,2,3,4-tetrahydroquinolin-6-yl}benzoic acid (**9**)

```
Current Data Parameters
NAME              Carbon
EXPNO                  5
PROCNO                 1

F2 - Acquisition Parameters
Date_           20220310
Time               17.29 h
INSTRUM           Avance
PROBHD   Z159656_0020 (
PULPROG          zgpg30
TD                 65536
SOLVENT            CD3CN
NS                  1024
DS                     4
SWH            35714.285 Hz
FIDRES          1.089913 Hz
AQ             0.9175040 sec
RG                   101
DW                14.000 usec
DE                 18.00 usec
TE                 298.0 K
D1            2.00000000 sec
D11           0.03000000 sec
TD0                    1
SFO1         150.9908267 MHz
NUC1                 13C
P0                  3.33 usec
P1                 10.00 usec
PLW1         41.91400146 W
SFO2         600.4224017 MHz
NUC2                  1H
CPDPRG[2         waltz16
PCPD2              80.00 usec
PLW2         13.51200008 W
PLW12         0.30124050 W
PLW13         0.15098180 W

F2 - Processing parameters
SI                 65536
SF           150.9755777 MHz
WDW                   EM
SSB                    0
LB                  1.00 Hz
GB                     0
PC                  1.40
```

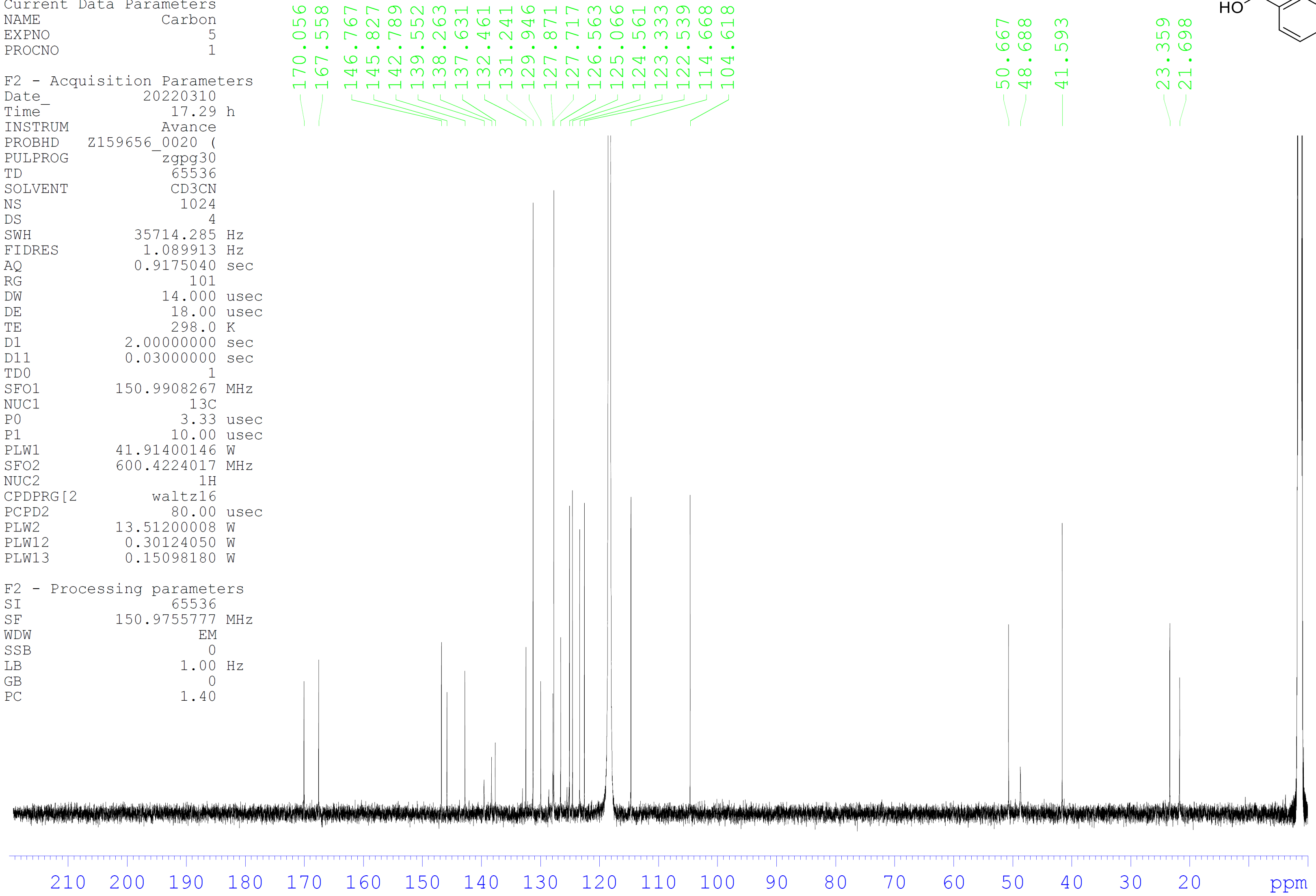

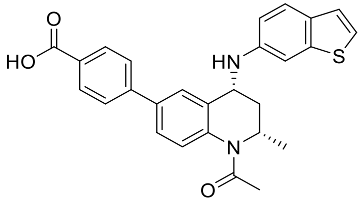

^{1}H NMR ethyl 4-{(2*R*,4*S*)-1-acetyl-4-[(1-benzothiophen-6-yl)amino]-2-methyl-1,2,3,4-tetrahydroquinolin-6-yl}benzoate (**17**)

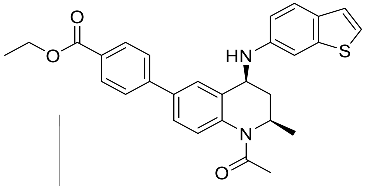

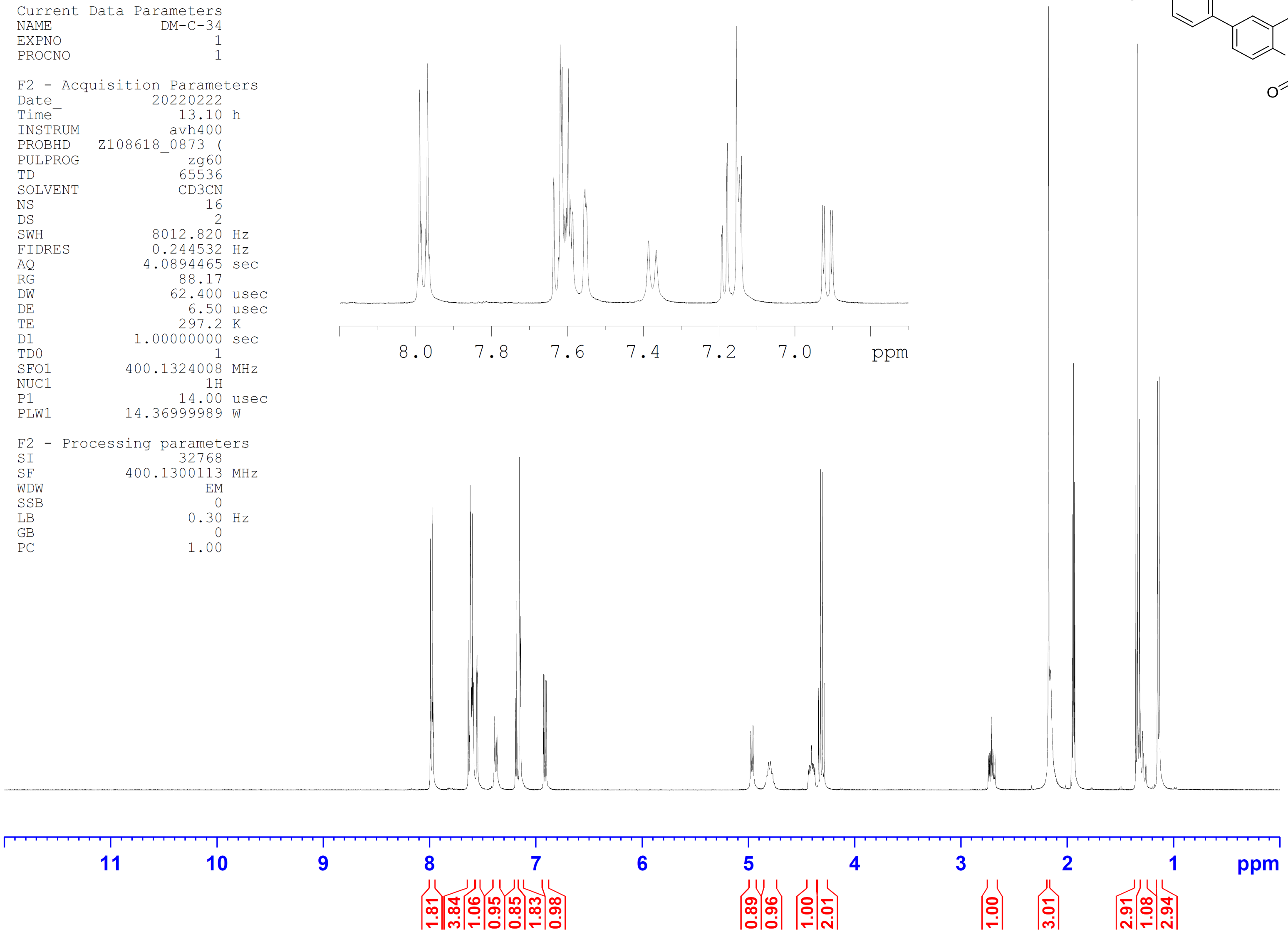

^{13}C NMR ethyl 4-{(2*R*,4*S*)-1-acetyl-4-[(1-benzothiophen-6-yl)amino]-2-methyl-1,2,3,4-tetrahydroquinolin-6-yl}benzoate (**17**)

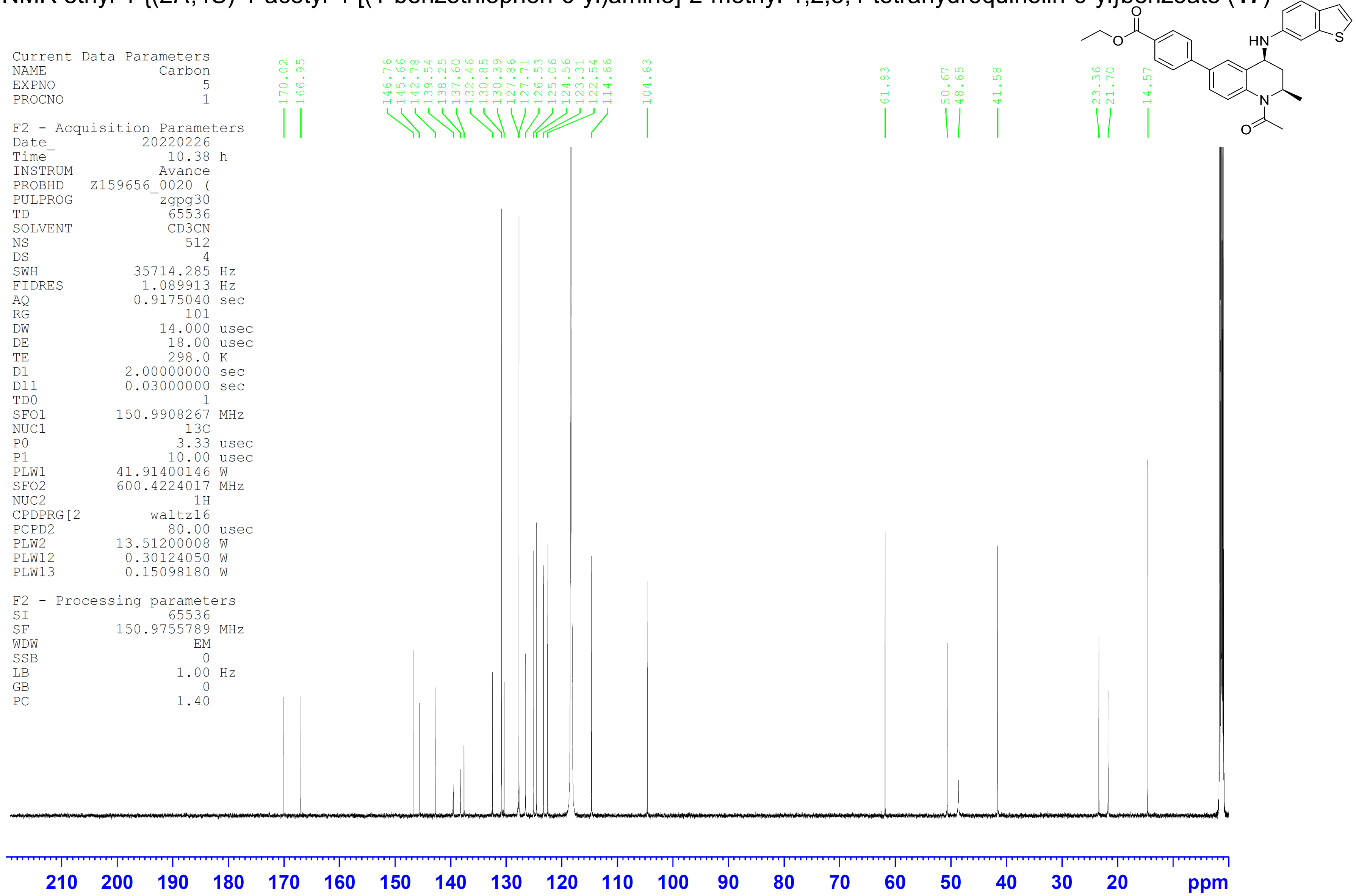

^{1}H NMR 4-{(2*R*,4*S*)-1-acetyl-4-[(1-benzothiophen-6-yl)amino]-2-methyl-1,2,3,4-tetrahydroquinolin-6-yl}benzoic acid (**10**)

```
Current Data Parameters
NAME              Proton
EXPNO                  1
PROCNO                 1

F2 - Acquisition Parameters
Date_           20220222
Time               12.07 h
INSTRUM           avh400
PROBHD   Z108618_0873 (
PULPROG             zg60
TD                 65536
SOLVENT            CD3CN
NS                    16
DS                     2
SWH             8012.820 Hz
FIDRES          0.244532 Hz
AQ             4.0894465 sec
RG                 88.17
DW                62.400 usec
DE                  6.50 usec
TE                 297.2 K
D1            1.00000000 sec
TD0                    1
SFO1         400.1324008 MHz
NUC1                  1H
P1                 14.00 usec
PLW1         14.36999989 W

F2 - Processing parameters
SI                 32768
SF           400.1300113 MHz
WDW                   EM
SSB                    0
LB                  0.30 Hz
GB                     0
PC                  1.00
```

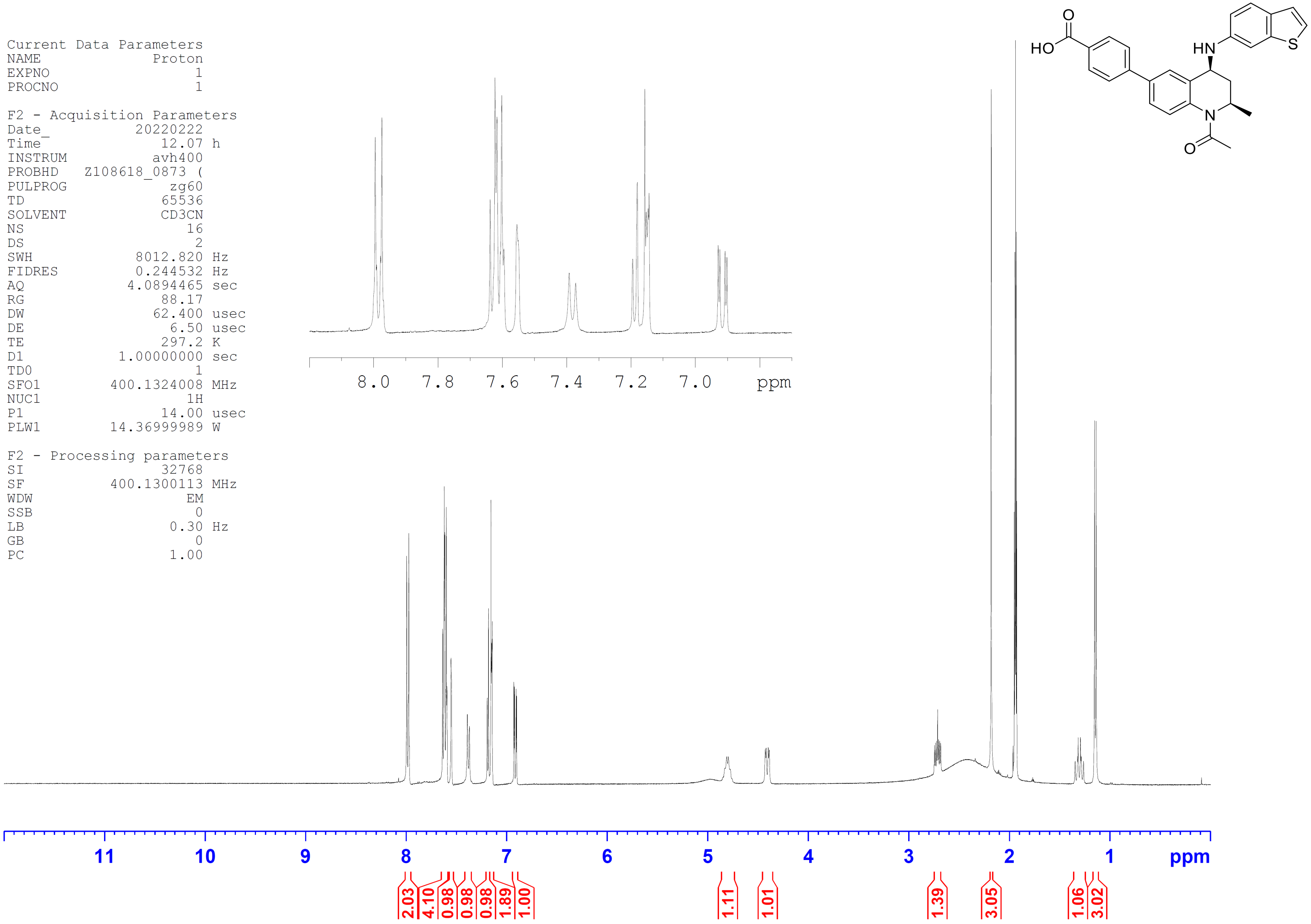

^{13}C NMR 4-{(2*R*,4*S*)-1-acetyl-4-[(1-benzothiophen-6-yl)amino]-2-methyl-1,2,3,4-tetrahydroquinolin-6-yl}benzoic acid (**10**)

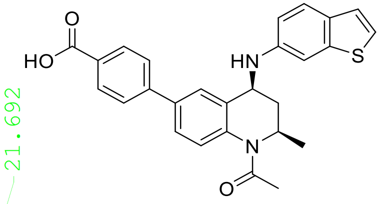

Current Data Parameters
NAME Carbon
EXPNO 5
PROCNO 1

F2 - Acquisition Parameters
Date_ 20220225
Time 8.55 h
INSTRUM Avance
PROBHD Z159656_0020 (
PULPROG zgpg30
TD 65536
SOLVENT CD3CN
NS 512
DS 4
SWH 35714.285 Hz
FIDRES 1.089913 Hz
AQ 0.9175040 sec
RG 101
DW 14.000 usec
DE 18.00 usec
TE 298.0 K
D1 2.00000000 sec
D11 0.03000000 sec
TD0 1
SFO1 150.9908267 MHz
NUC1 13C
P0 3.33 usec
P1 10.00 usec
PLW1 41.91400146 W
SFO2 600.4224017 MHz
NUC2 1H
CPDPRG[2 waltz16
PCPD2 80.00 usec
PLW2 13.51200008 W
PLW12 0.30124050 W
PLW13 0.15098180 W

F2 - Processing parameters
SI 65536
SF 150.9755785 MHz
WDW EM
SSB 0
LB 1.00 Hz
GB 0
PC 1.40

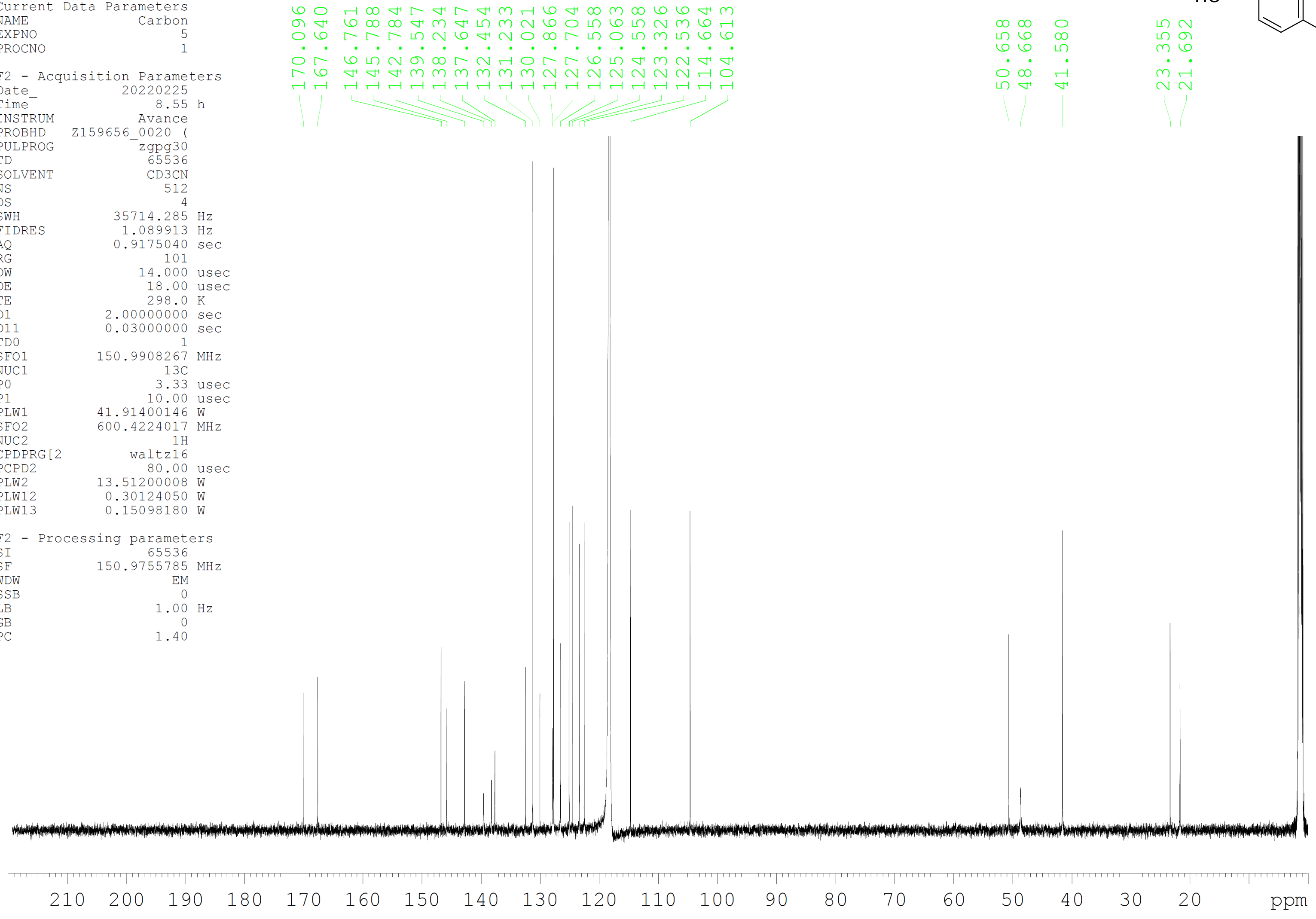

^{1}H NMR ethyl 4-{(2*S*,4*R*)-1-acetyl-4-[(1-benzofuran-6-yl)amino]-2-methyl-1,2,3,4-tetrahydroquinolin-6-yl}benzoate (**18**)

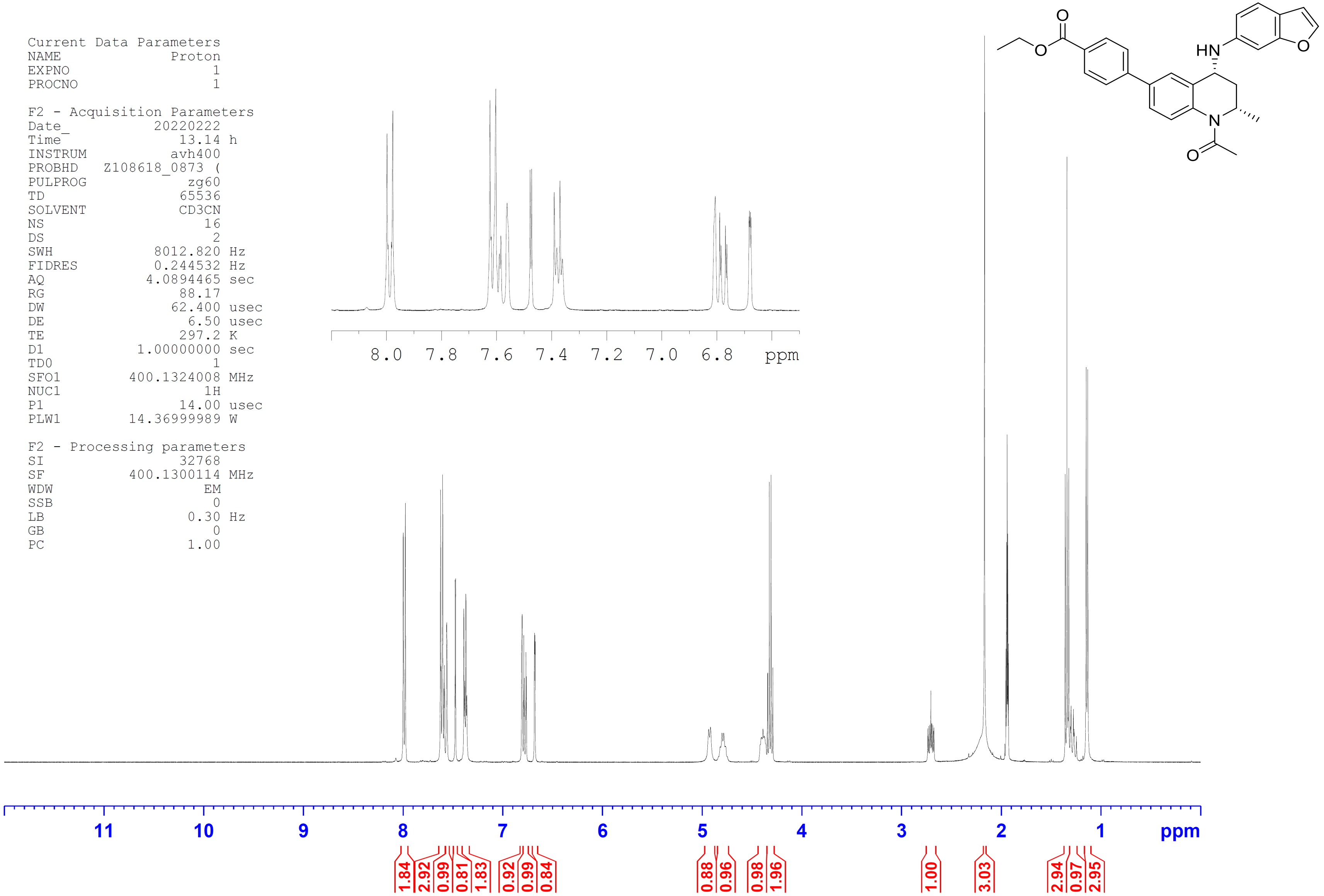

^{13}C NMR ethyl 4-{(2*S*,4*R*)-1-acetyl-4-[(1-benzofuran-6-yl)amino]-2-methyl-1,2,3,4-tetrahydroquinolin-6-yl}benzoate (**18**)

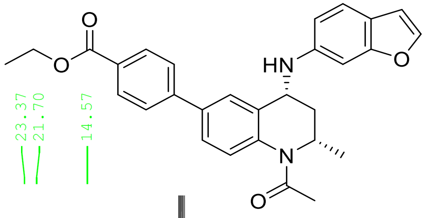


```
Current Data Parameters
NAME                Carbon
EXPNO                    5
PROCNO                   1

F2 - Acquisition Parameters
Date_             20220226
Time                  6.21 h
INSTRUM             Avance
PROBHD      Z159656_0020 (
PULPROG            zgpg30
TD                   65536
SOLVENT              CD3CN
NS                     512
DS                       4
SWH              35714.285 Hz
FIDRES            1.089913 Hz
AQ               0.9175040 sec
RG                     101
DW                  14.000 usec
DE                   18.00 usec
TE                   298.0 K
D1              2.00000000 sec
D11             0.03000000 sec
TD0                      1
SFO1           150.9908267 MHz
NUC1                   13C
P0                    3.33 usec
P1                   10.00 usec
PLW1           41.91400146 W
SFO2           600.4224017 MHz
NUC2                    1H
CPDPRG[2           waltz16
PCPD2                80.00 usec
PLW2           13.51200008 W
PLW12           0.30124050 W
PLW13           0.15098180 W

F2 - Processing parameters
SI                   65536
SF             150.9755790 MHz
WDW                     EM
SSB                      0
LB                    1.00 Hz
GB                       0
PC                    1.40
```

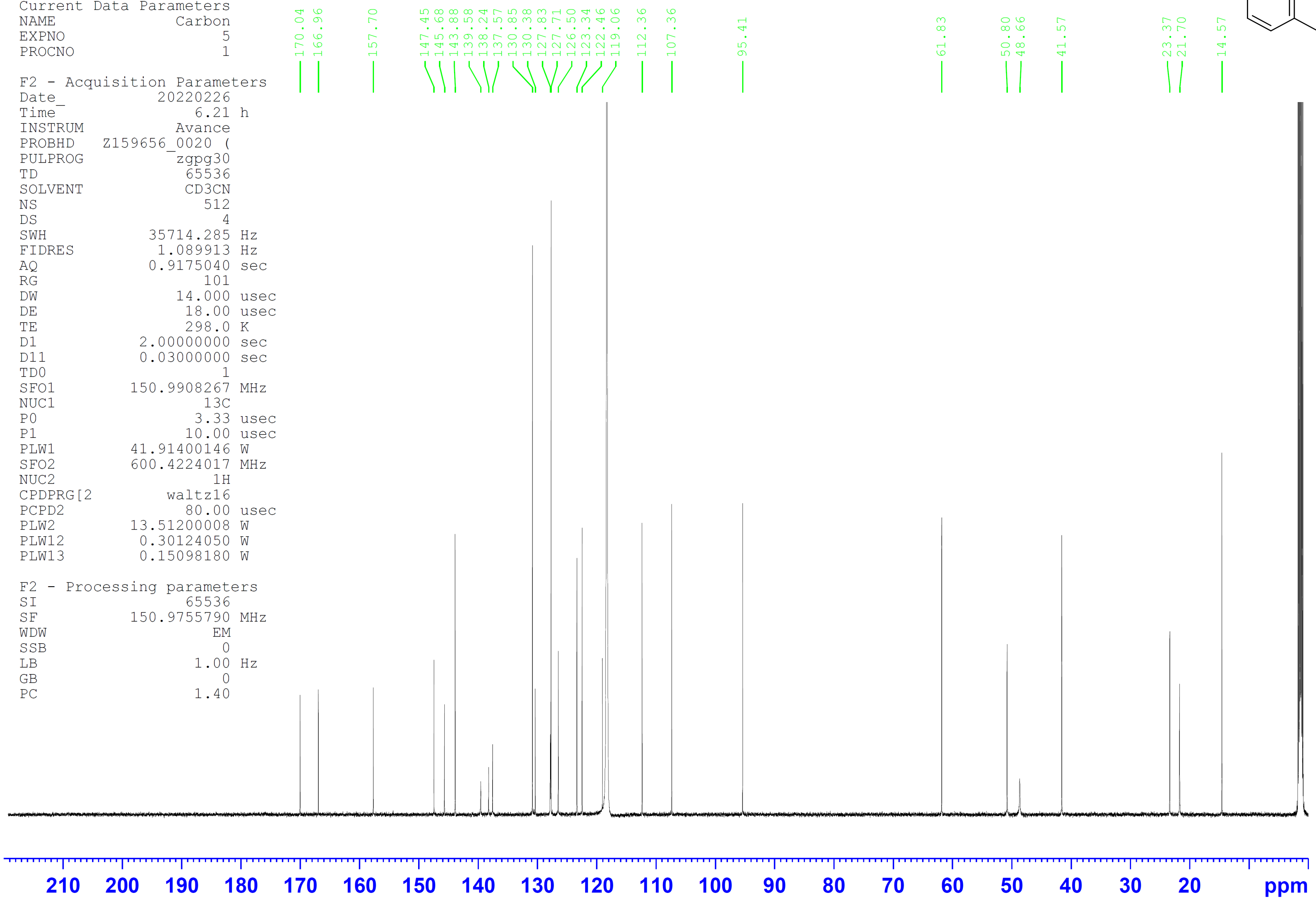

^{1}H NMR 4-((2*S*,4*R*)-1-acetyl-4-[(1-benzofuran-6-yl)amino]-2-methyl-1,2,3,4-tetrahydroquinolin-6-yl)benzoic acid (**11**)

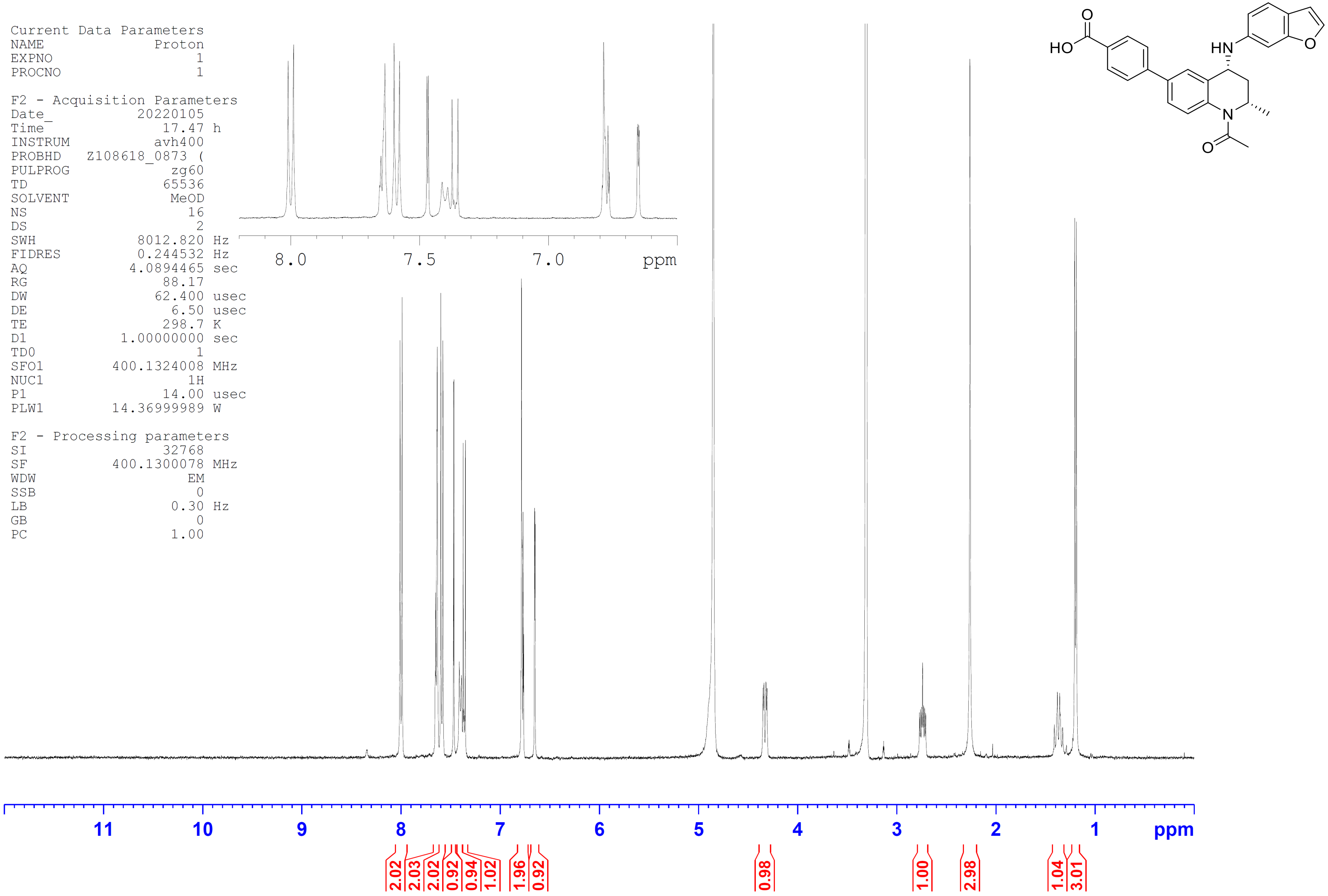

^{13}C NMR 4-((2*S*,4*R*)-1-acetyl-4-[(1-benzofuran-6-yl)amino]-2-methyl-1,2,3,4-tetrahydroquinolin-6-yl)benzoic acid (**11**)

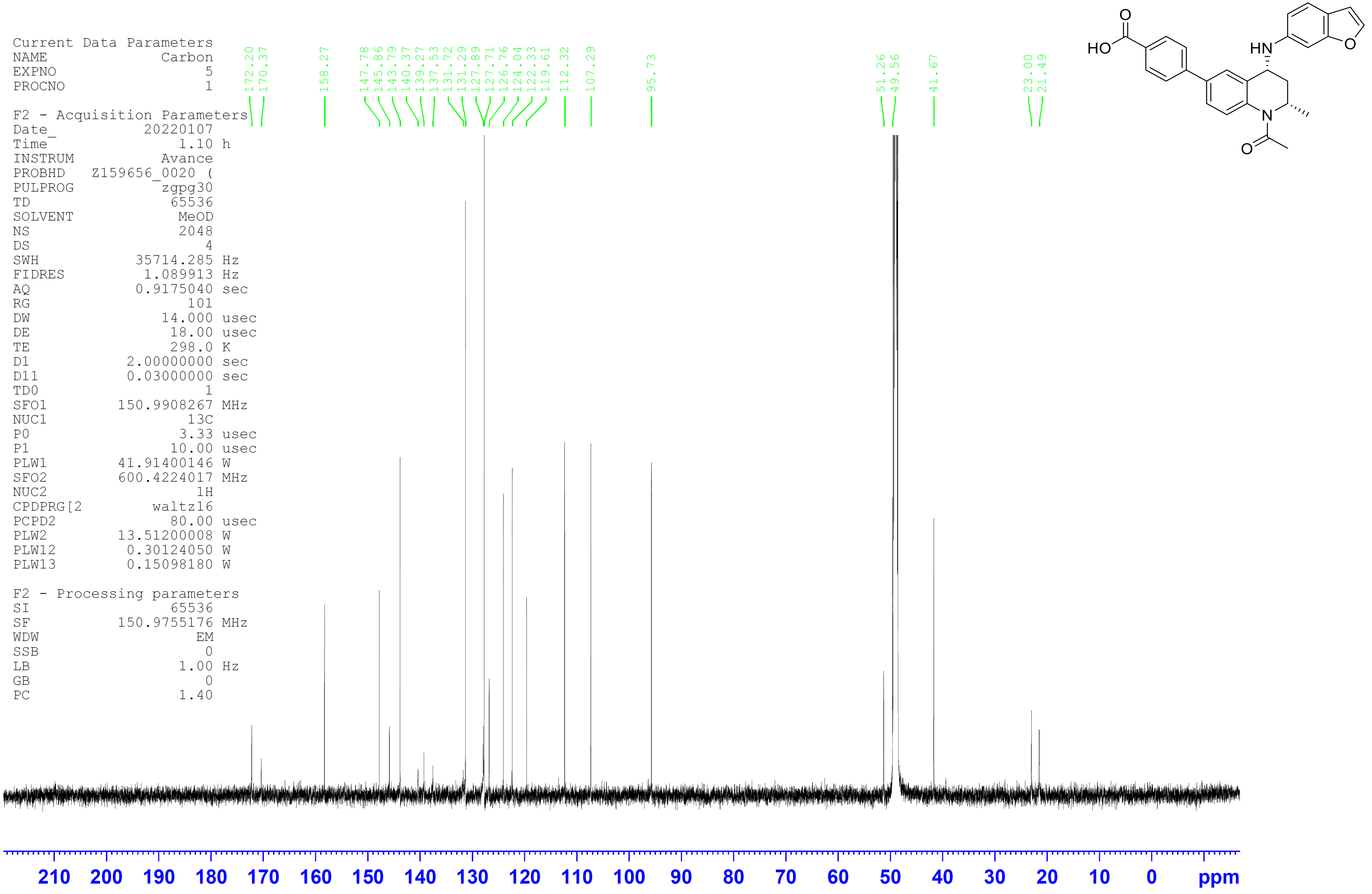

^{1}H NMR ethyl 4-{(2*S*,4*R*)-1-acetyl-4-[(1-benzothiophen-5-ylamino]-2-methyl-1,2,3,4-tetrahydroquinolin-6-yl}benzoate (**19**)

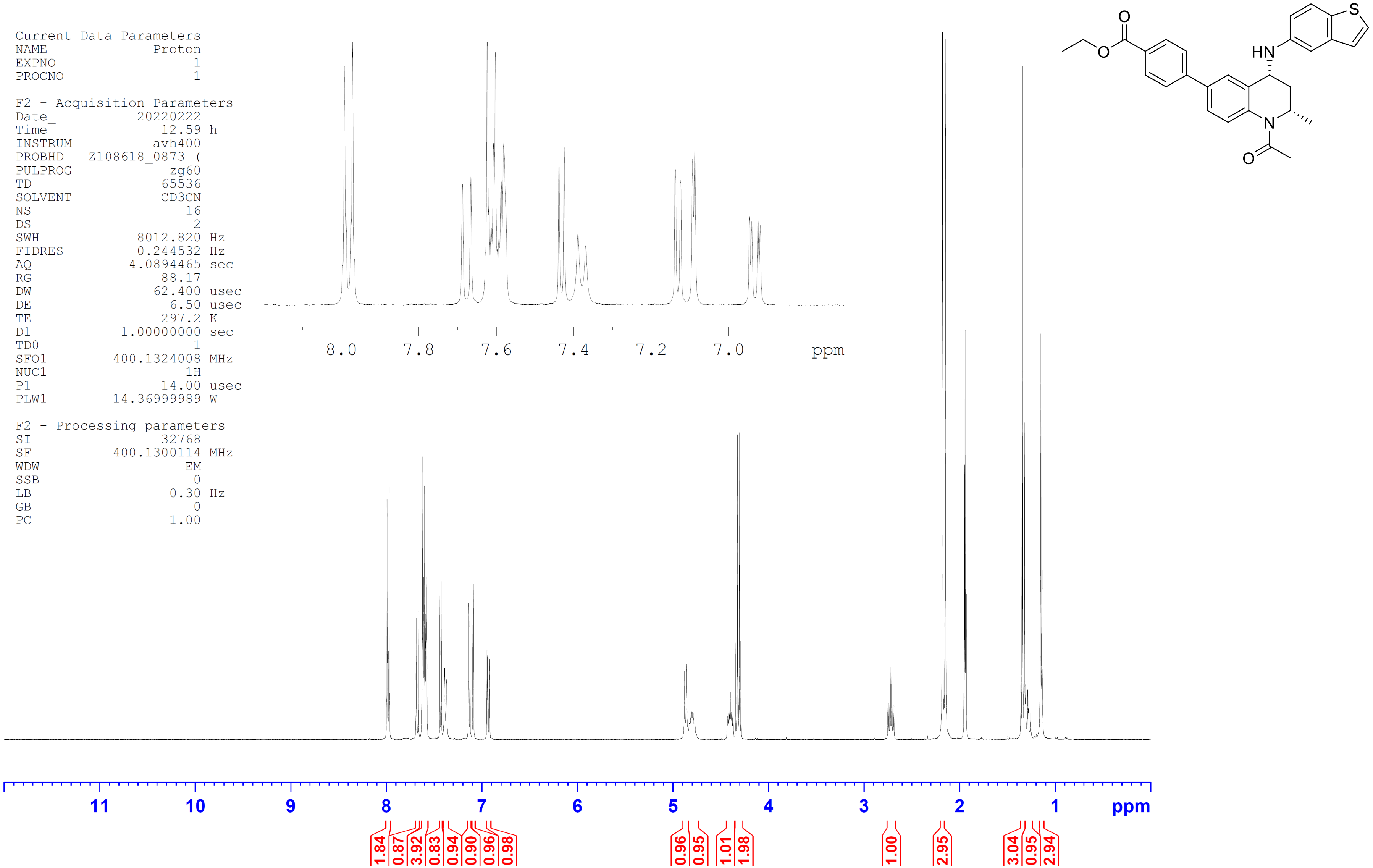

^{13}C NMR ethyl 4-{(2*S*,4*R*)-1-acetyl-4-[(1-benzothiophen-5-ylamino]-2-methyl-1,2,3,4-tetrahydroquinolin-6-yl}benzoate (**19**)

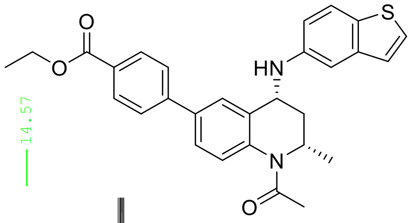


```
Current Data Parameters
NAME                Carbon
EXPNO                    5
PROCNO                   1

F2 - Acquisition Parameters
Date_             20220226
Time                8.29 h
INSTRUM             Avance
PROBHD    Z159656_0020 (
PULPROG            zgpg30
TD                  65536
SOLVENT             CD3CN
NS                    512
DS                      4
SWH             35714.285 Hz
FIDRES           1.089913 Hz
AQ              0.9175040 sec
RG                    101
DW                 14.000 usec
DE                  18.00 usec
TE                  298.0 K
D1             2.00000000 sec
D11            0.03000000 sec
TD0                     1
SFO1          150.9908267 MHz
NUC1                  13C
P0                   3.33 usec
P1                  10.00 usec
PLW1          41.91400146 W
SFO2          600.4224017 MHz
NUC2                   1H
CPDPRG[2           waltz16
PCPD2               80.00 usec
PLW2          13.51200008 W
PLW12          0.30124050 W
PLW13          0.15098180 W

F2 - Processing parameters
SI                  65536
SF            150.9755789 MHz
WDW                    EM
SSB                     0
LB                   1.00 Hz
GB                      0
PC                   1.40
```

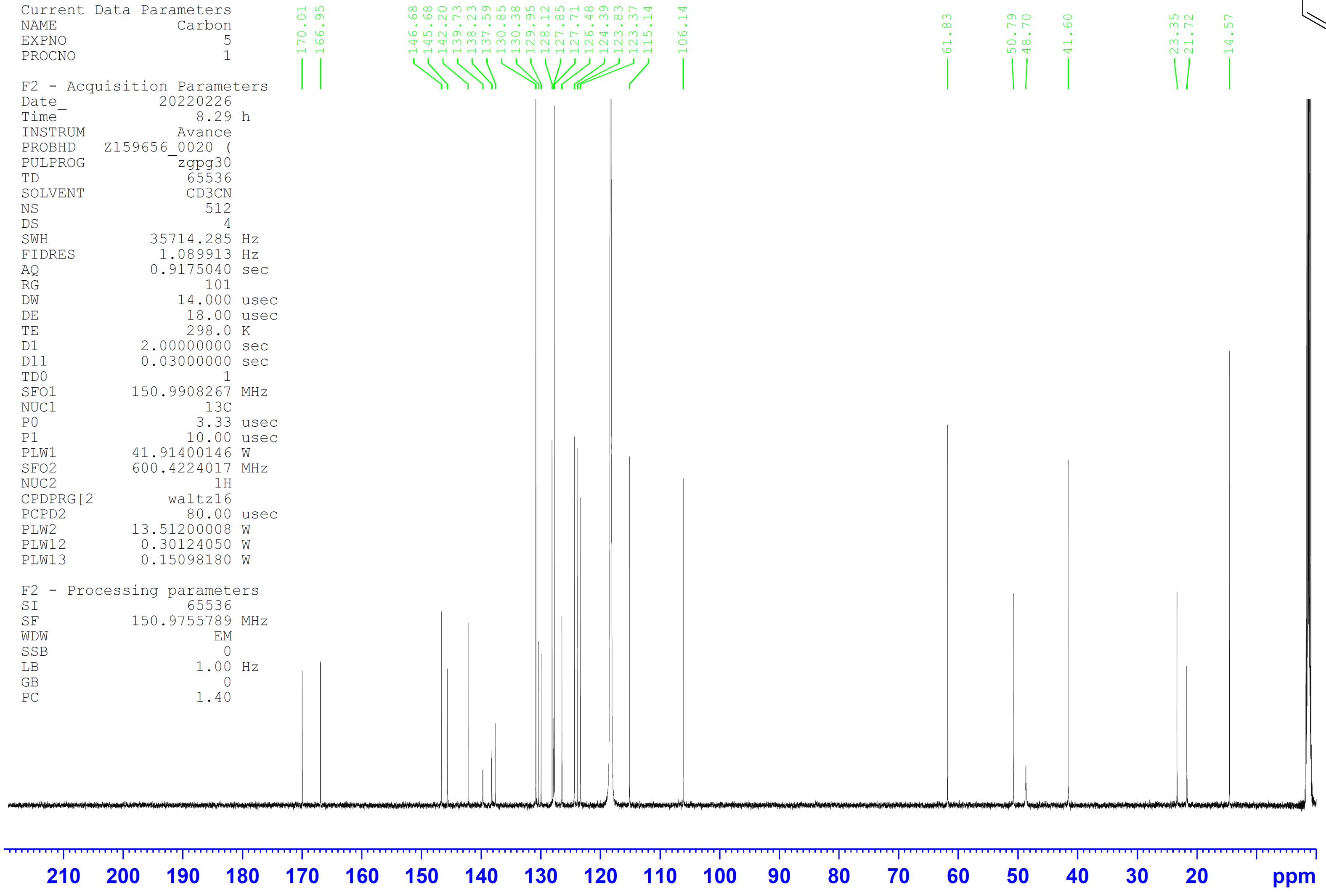

^{1}H NMR 4-((2*S*,4*R*)-1-acetyl-4-[(1-benzothiophen-5-yl)amino]-2-methyl-1,2,3,4-tetrahydroquinolin-6-yl)benzoic acid (**12**)

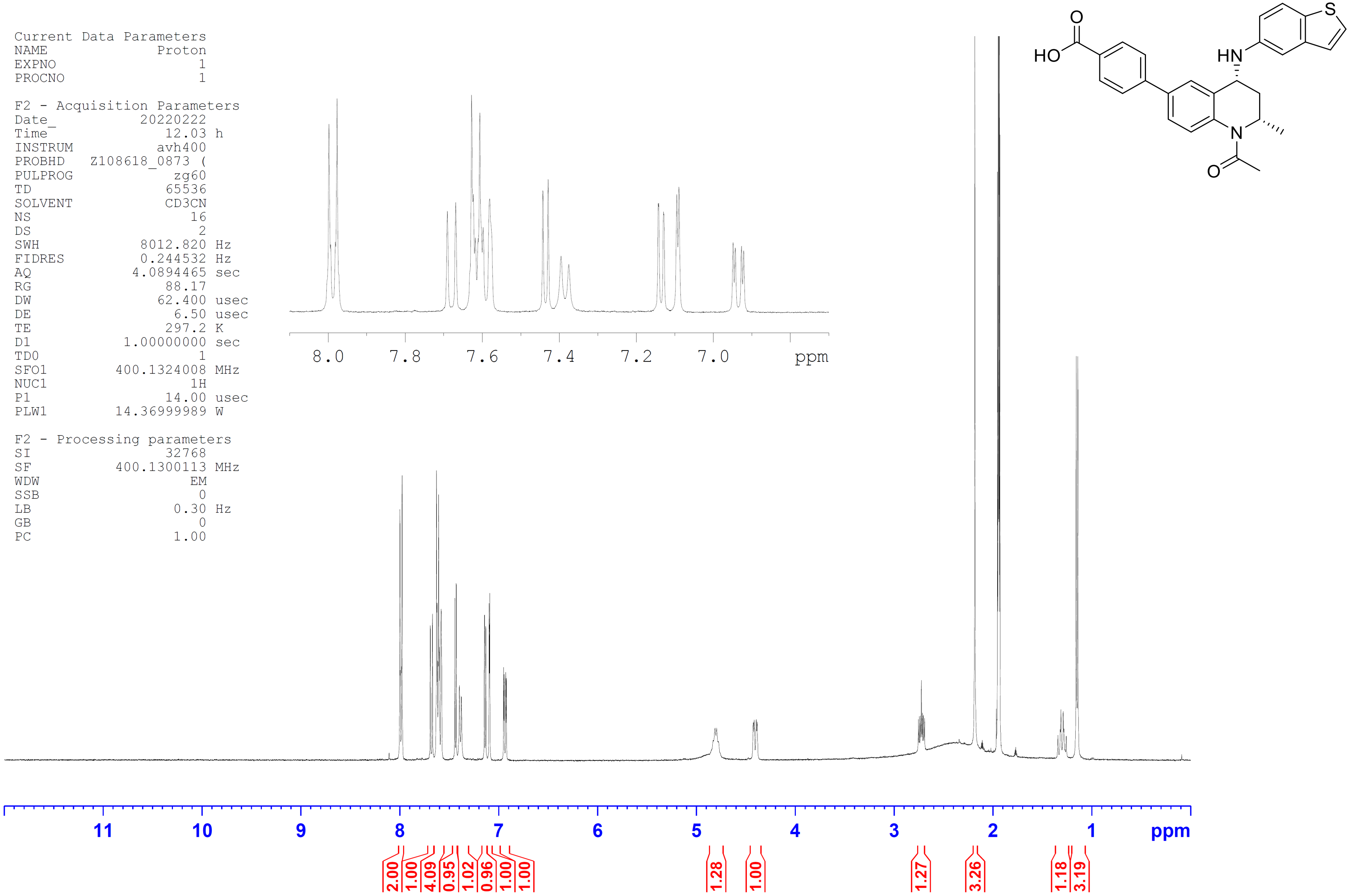

^{13}C NMR 4-((2*S*,4*R*)-1-acetyl-4-[(1-benzothiophen-5-yl)amino]-2-methyl-1,2,3,4-tetrahydroquinolin-6-yl)benzoic acid (**12**)

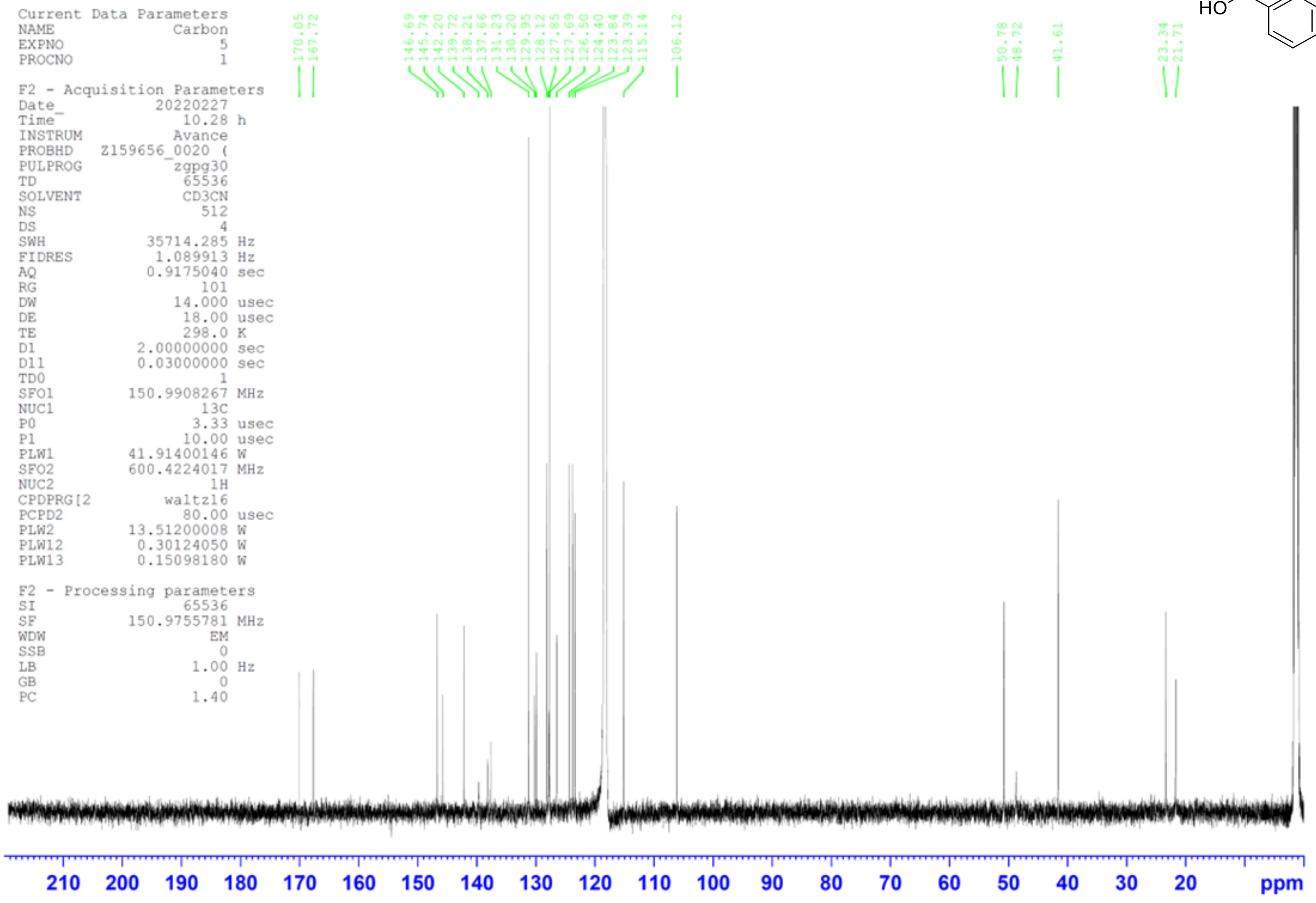

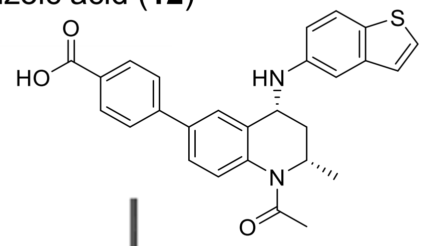

^{1}H NMR *tert*-butyl 5-{(2*S*,4*R*)-(1-acetyl-6-[4-(ethoxycarbonyl)phenyl]-2-methyl-1,2,3,4-tetrahydroquinolin-4-yl)amino}-1*H*-indole-1-carboxylate **(S9)**

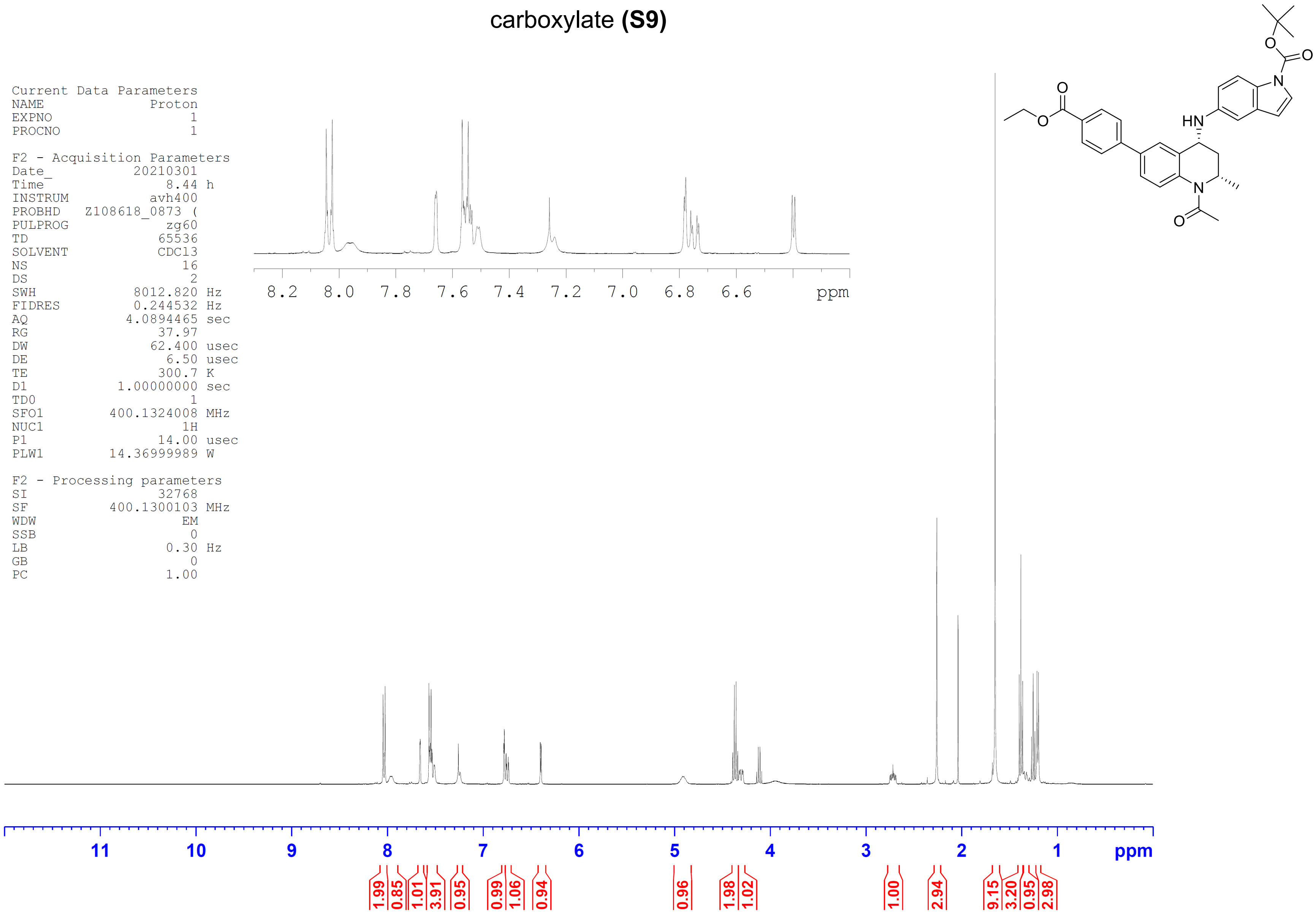

^{13}C NMR *tert*-butyl 5-{(2*S*,4*R*)-(1-acetyl-6-[4-(ethoxycarbonyl)phenyl]-2-methyl-1,2,3,4-tetrahydroquinolin-4-yl)amino}-1*H*-indole-1-carboxylate **(S9)**

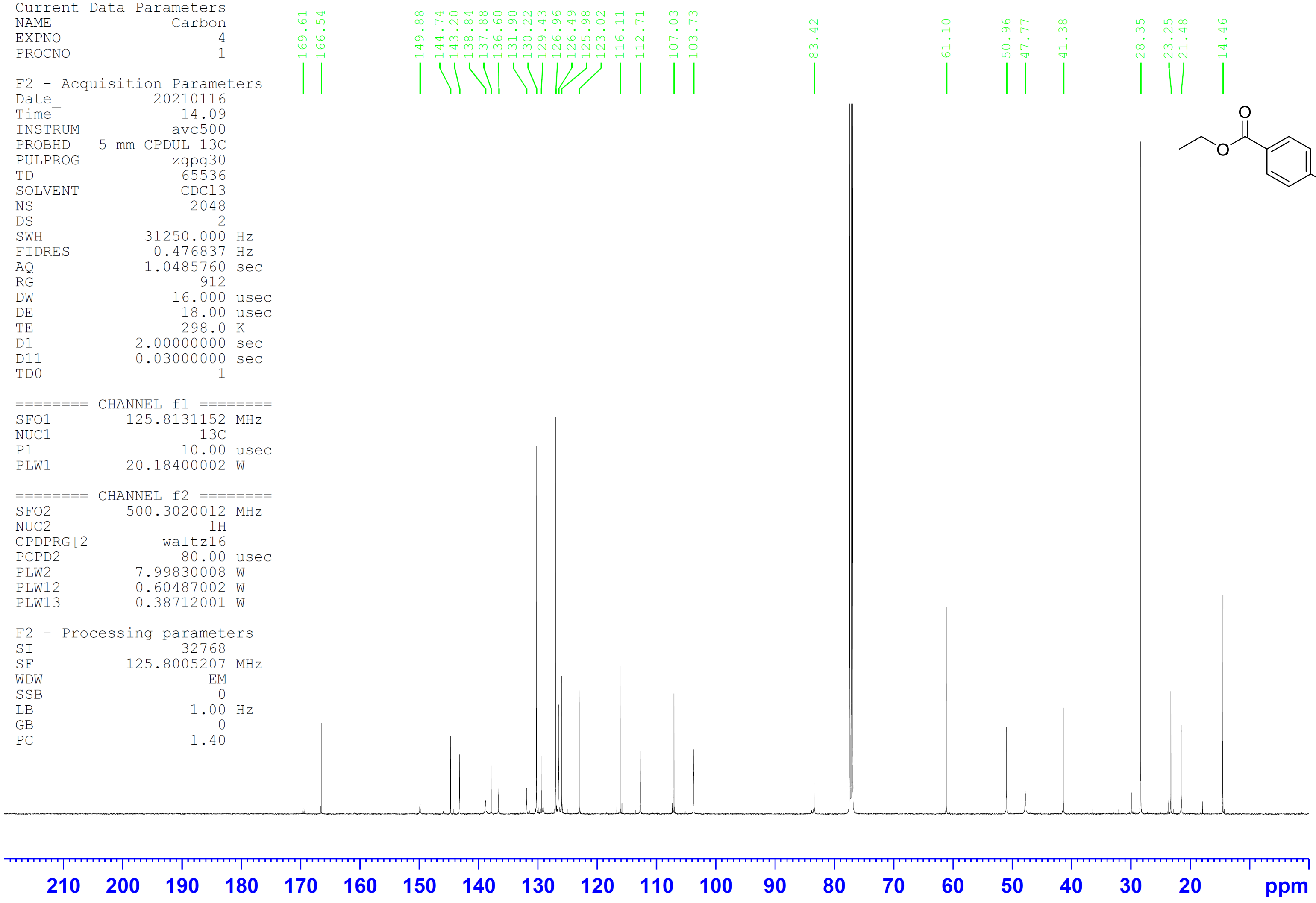

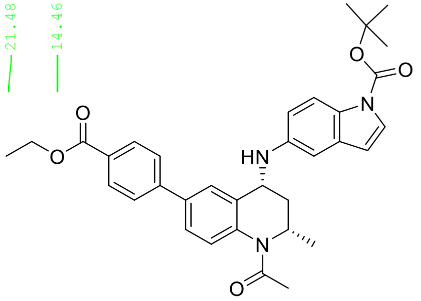

^{1}H NMR ethyl 4-{(2*S*,4*R*)-1-acetyl-4-[(1*H*-indol-5-yl)amino]-2-methyl-1,2,3,4-tetrahydroquinolin-6-yl}benzoate (**20**)

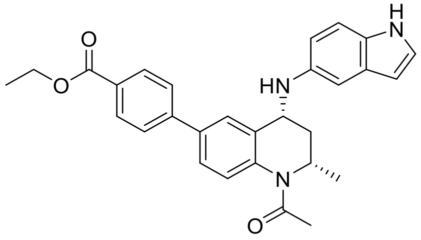


```
Current Data Parameters
NAME              Proton
EXPNO                  1
PROCNO                 1

F2 - Acquisition Parameters
Date_           20210416
Time                9.31 h
INSTRUM            av600
PROBHD   Z130037_0008 (
PULPROG             zg30
TD                 65536
SOLVENT            CD3CN
NS                    16
DS                     2
SWH            12019.230 Hz
FIDRES          0.366798 Hz
AQ             2.7262976 sec
RG                197.67
DW                41.600 usec
DE                 10.00 usec
TE                 298.0 K
D1            1.00000000 sec
TD0                    1
SFO1         600.1830009 MHz
NUC1                  1H
P0                  4.00 usec
P1                 12.00 usec
PLW1         24.00000000 W

F2 - Processing parameters
SI                 65536
SF           600.1800168 MHz
WDW                   EM
SSB                    0
LB                  0.30 Hz
GB                     0
PC                  1.00
```

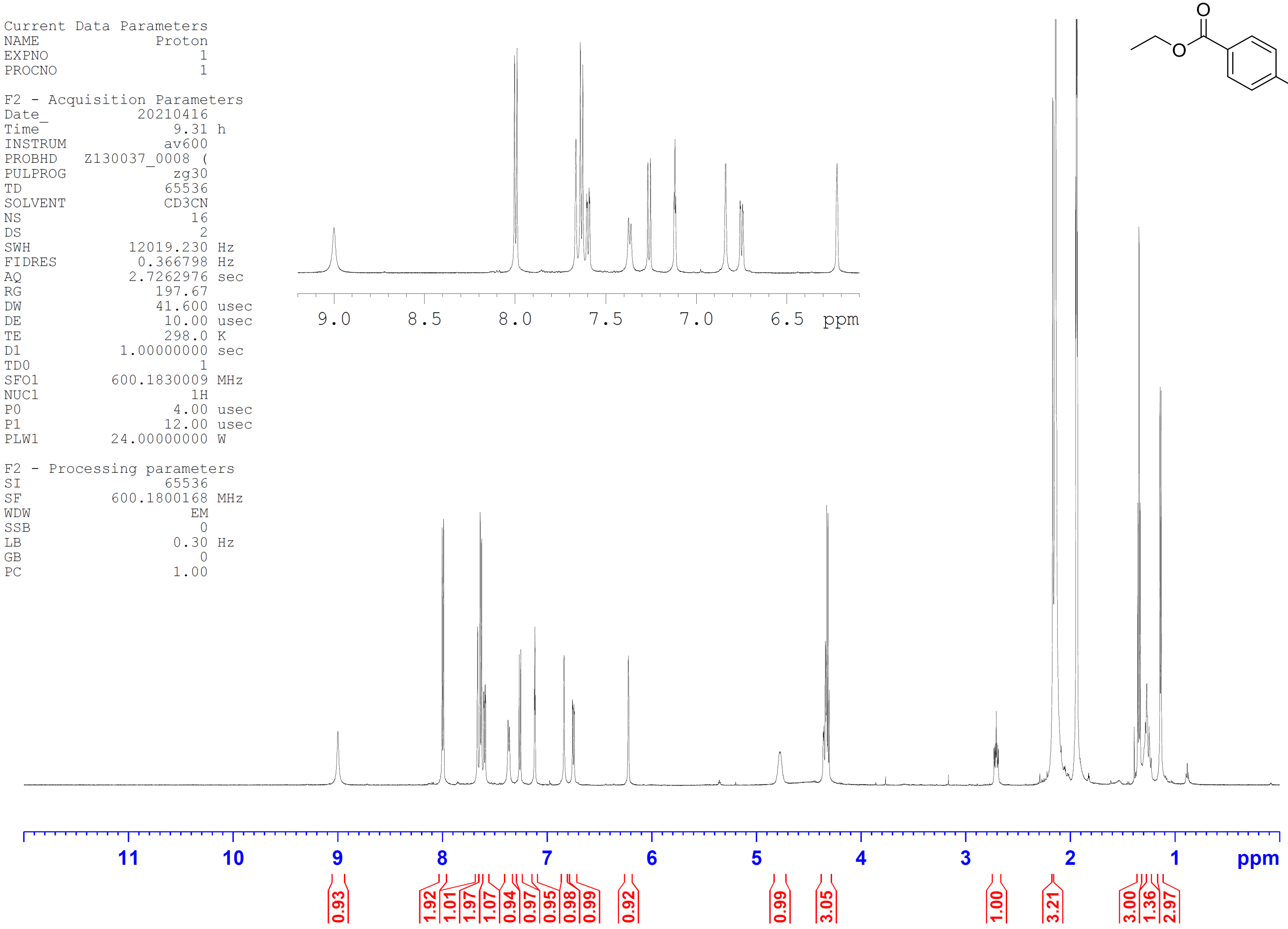

^{13}C NMR ethyl 4-{(2*S*,4*R*)-1-acetyl-4-[(1*H*-indol-5-yl)amino]-2-methyl-1,2,3,4-tetrahydroquinolin-6-yl}benzoate (**20**)

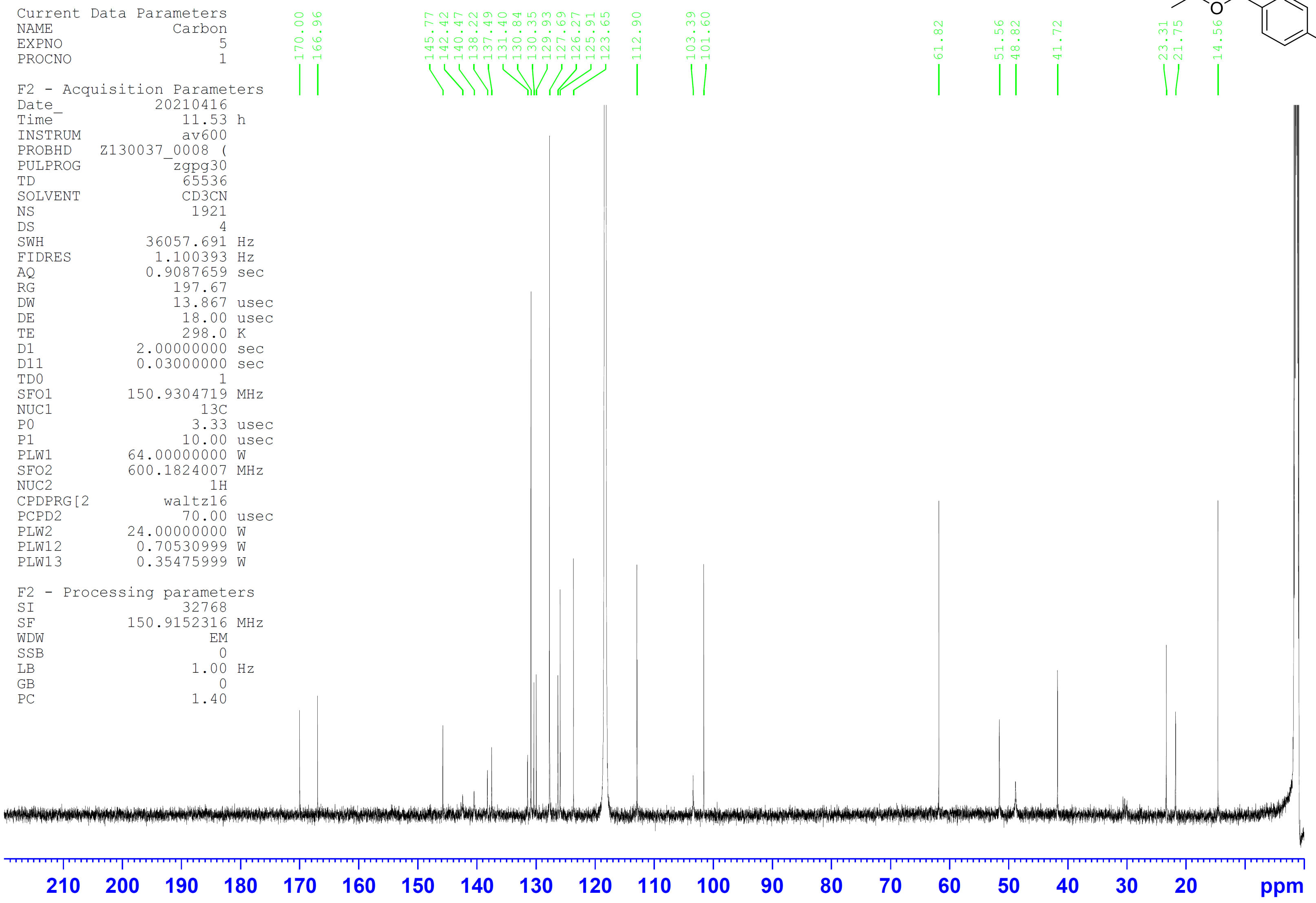

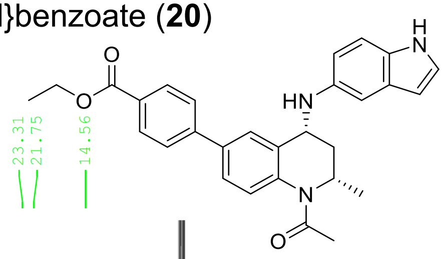

^{1}H NMR 4-{(2*S*,4*R*)-1-acetyl-4-[(1*H*-indol-5-yl)amino]-2-methyl-1,2,3,4-tetrahydroquinolin-6-yl}benzoic acid (**13**)

```
Current Data Parameters
NAME              Proton
EXPNO                  1
PROCNO                 1

F2 - Acquisition Parameters
Date_           20210802
Time               17.26 h
INSTRUM           avg400
PROBHD   Z108618_0816 (
PULPROG             zg60
TD                 65536
SOLVENT             MeOD
NS                    16
DS                     2
SWH             8012.820 Hz
FIDRES          0.244532 Hz
AQ             4.0894465 sec
RG                206.87
DW                62.400 usec
DE                  6.50 usec
TE                 299.1 K
D1           1.00000000 sec
TD0                    1
SFO1         400.2024012 MHz
NUC1                  1H
P1                 14.00 usec
PLW1         14.00000000 W

F2 - Processing parameters
SI                 32768
SF           400.2000077 MHz
WDW                   EM
SSB                    0
LB                  0.30 Hz
GB                     0
PC                  1.00
```

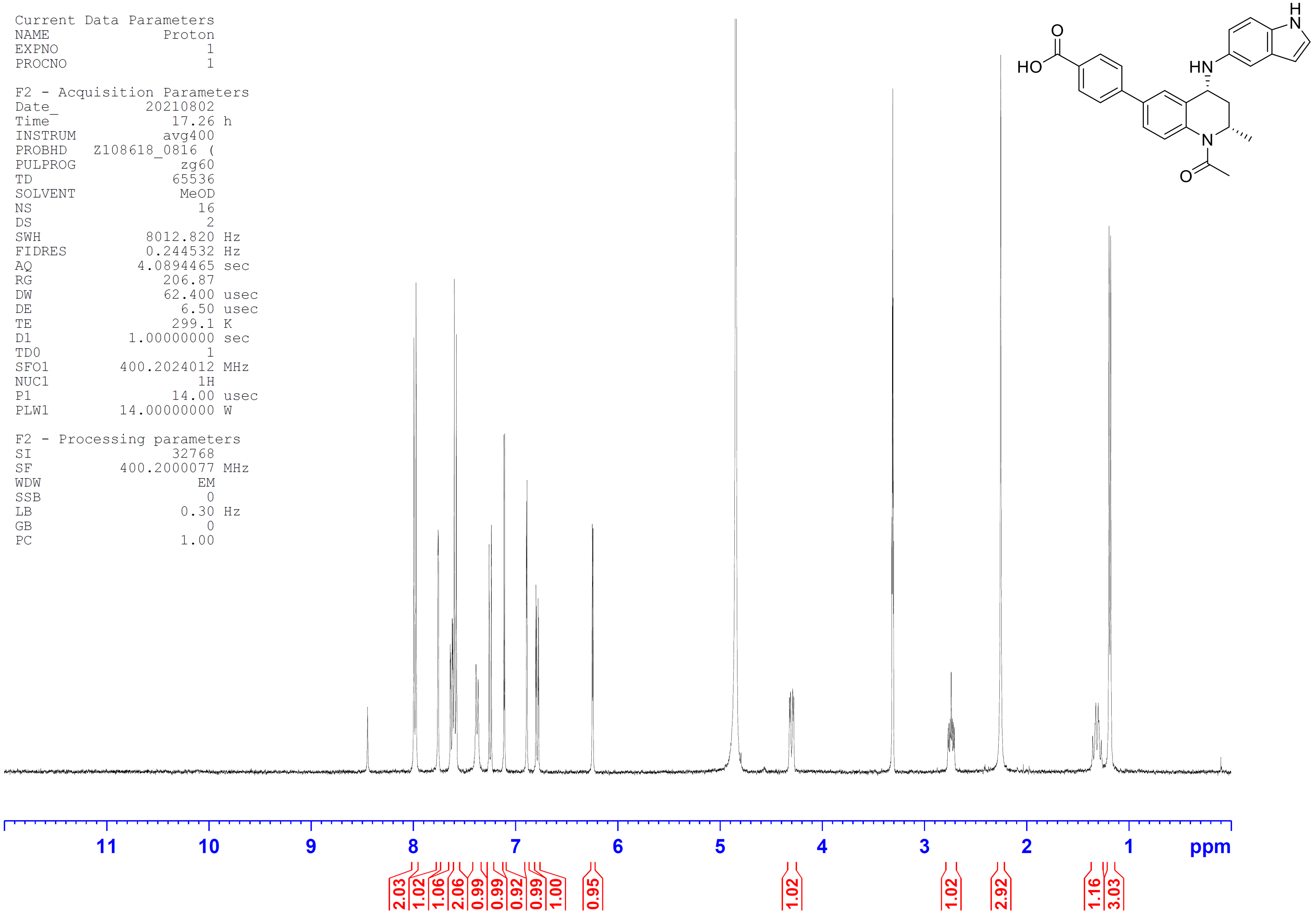

^{13}C NMR 4-{(2*S*,4*R*)-1-acetyl-4-[(1*H*-indol-5-yl)amino]-2-methyl-1,2,3,4-tetrahydroquinolin-6-yl}benzoic acid (**13**)

```
Current Data Parameters
NAME                Carbon
EXPNO                    5
PROCNO                   1

F2 - Acquisition Parameters
Date_             20210422
Time                 14.45 h
INSTRUM              av600
PROBHD   Z130037_0008 (
PULPROG            zgpg30
TD                   65536
SOLVENT               MeOD
NS                    3072
DS                       4
SWH              36057.691 Hz
FIDRES            1.100393 Hz
AQ               0.9087659 sec
RG                  197.67
DW                  13.867 usec
DE                   18.00 usec
TE                   298.0 K
D1              2.00000000 sec
D11             0.03000000 sec
TD0                      1
SFO1           150.9304719 MHz
NUC1                   13C
P0                    3.33 usec
P1                   10.00 usec
PLW1           64.00000000 W
SFO2           600.1824007 MHz
NUC2                    1H
CPDPRG[2           waltz16
PCPD2                70.00 usec
PLW2           24.00000000 W
PLW12           0.70530999 W
PLW13           0.35475999 W

F2 - Processing parameters
SI                   32768
SF             150.9151685 MHz
WDW                     EM
SSB                      0
LB                    2.00 Hz
GB                       0
PC                    1.40
```

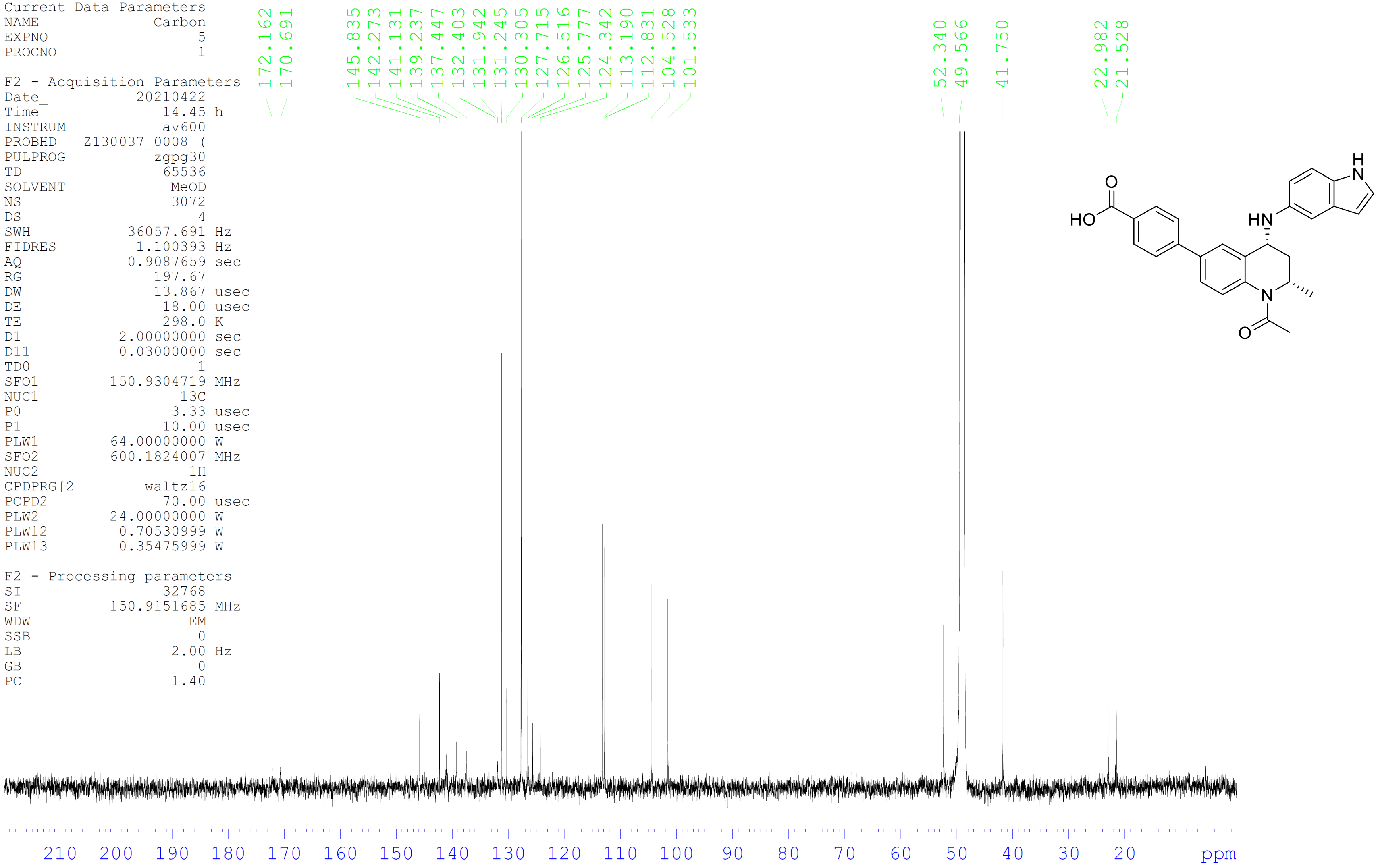

^{1}H NMR ethyl 4-{(2*S*,4*R*)1-acetyl-4-[(1,3-benzothiazol-6-yl)amino]-2-methyl-1,2,3,4-tetrahydroquinolin-6-yl}benzoate (**21**)

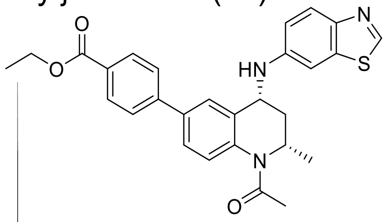


```
Current Data Parameters
NAME                Proton
EXPNO                    1
PROCNO                   1

F2 - Acquisition Parameters
Date_             20210316
Time                 23.22
INSTRUM             avc500
PROBHD    5 mm CPDUL 13C
PULPROG               zg30
TD                   65536
SOLVENT              CD3CN
NS                      16
DS                       4
SWH             10330.578 Hz
FIDRES           0.157632 Hz
AQ              3.1719425 sec
RG                    3.56
DW                  48.400 usec
DE                   10.00 usec
TE                   298.0 K
D1             1.00000000 sec
TD0                      1

======== CHANNEL f1 ========
SFO1          500.3030896 MHz
NUC1                    1H
P1                   22.00 usec
PLW1            7.99830008 W

F2 - Processing parameters
SI                   65536
SF            500.3000152 MHz
WDW                     EM
SSB                      0
LB                    0.30 Hz
GB                       0
PC                    1.00
```

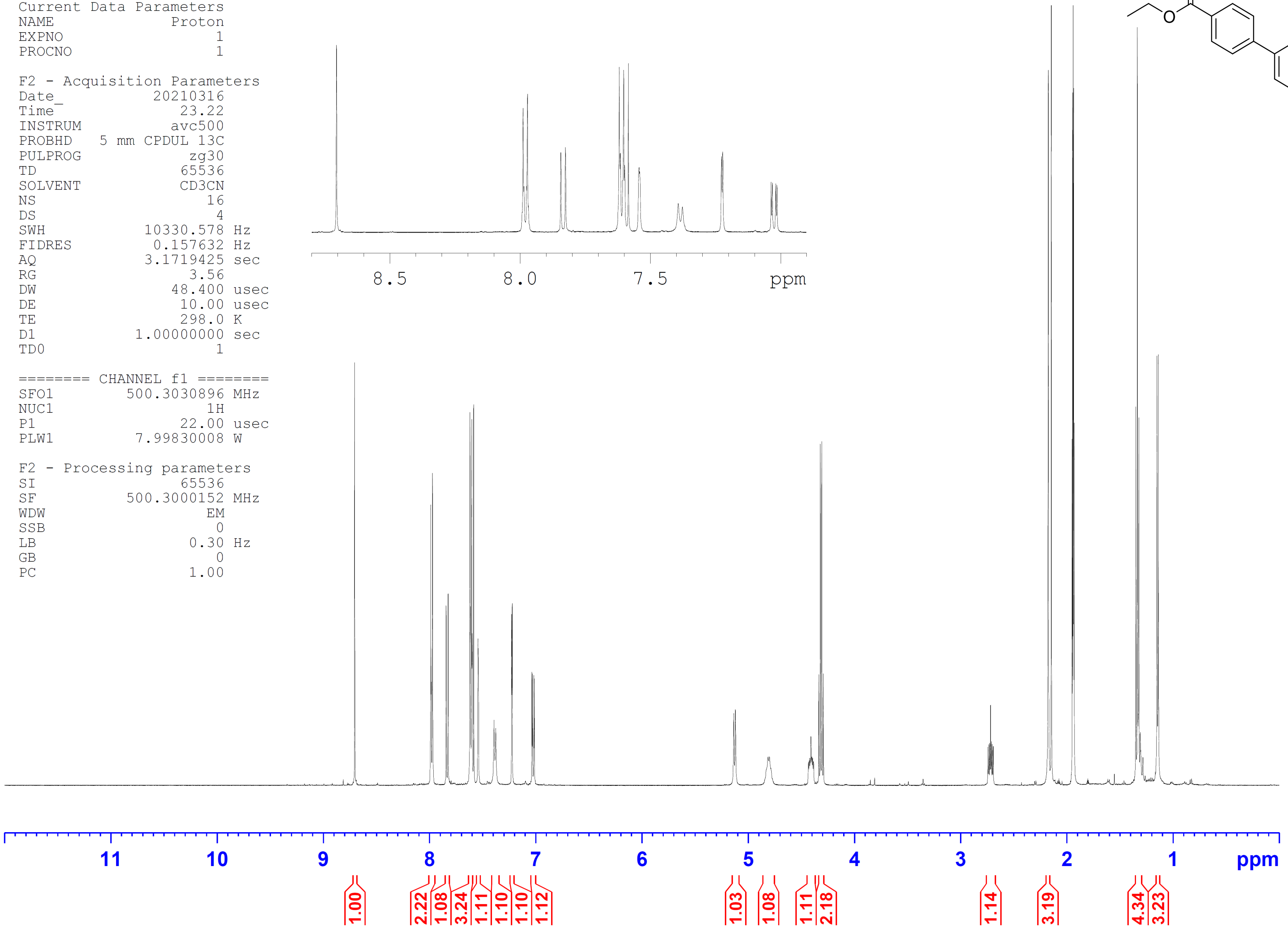

^{13}C NMR ethyl 4-{(2*S*,4*R*)1-acetyl-4-[(1,3-benzothiazol-6-yl)amino]-2-methyl-1,2,3,4-tetrahydroquinolin-6-yl}benzoate (**21**)

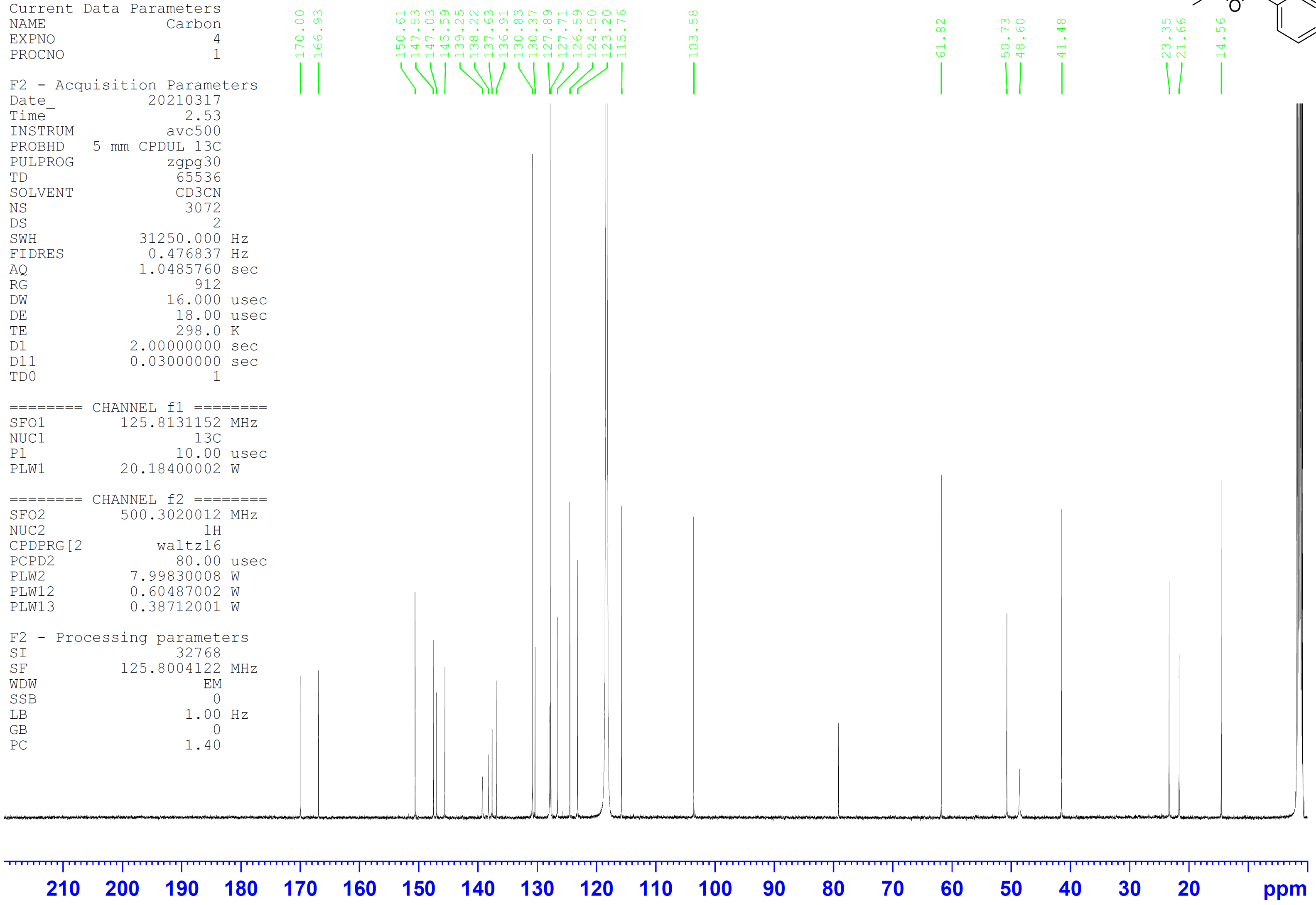

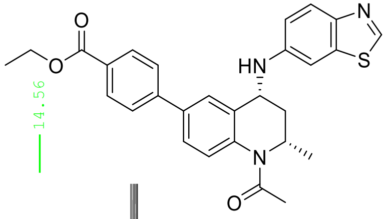

^{1}H NMR 4-{(2*S*,4*R*)1-acetyl-4-[(1,3-benzothiazol-6-yl)amino]-2-methyl-1,2,3,4-tetrahydroquinolin-6-yl}benzoic acid (**14**)

```
Current Data Parameters
NAME              Proton
EXPNO                  1
PROCNO                 1

F2 - Acquisition Parameters
Date_           20210426
Time               10.58 h
INSTRUM           avh400
PROBHD   Z108618_0873 (
PULPROG             zg60
TD                 65536
SOLVENT             MeOD
NS                    16
DS                     2
SWH             8012.820 Hz
FIDRES          0.244532 Hz
AQ             4.0894465 sec
RG                 88.17
DW                62.400 usec
DE                  6.50 usec
TE                 299.3 K
D1            1.00000000 sec
TD0                    1
SFO1         400.1324008 MHz
NUC1                  1H
P1                 14.00 usec
PLW1         14.36999989 W

F2 - Processing parameters
SI                 32768
SF           400.1300078 MHz
WDW                   EM
SSB                    0
LB                  0.30 Hz
GB                     0
PC                  1.00
```

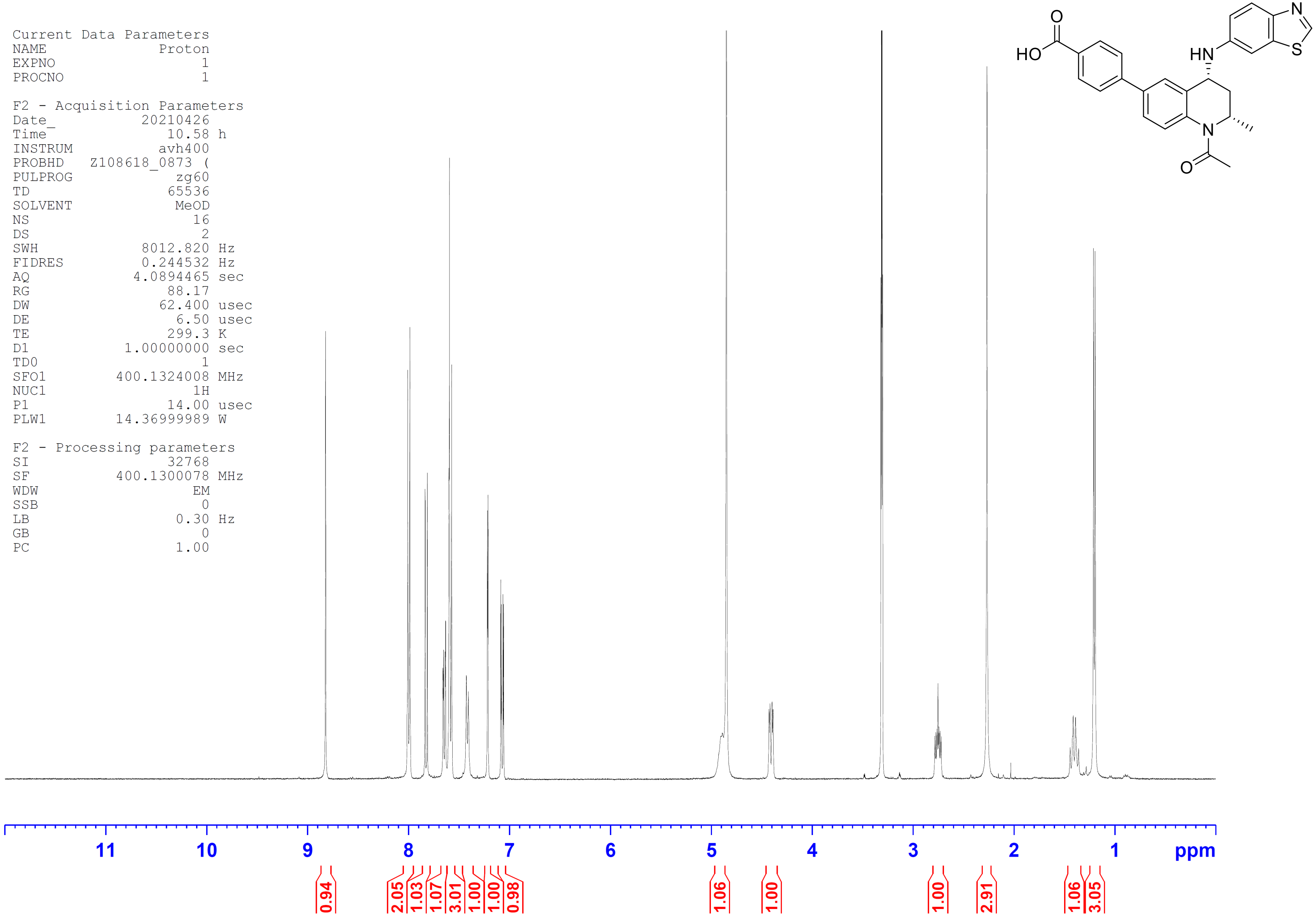

^{13}C NMR 4-{(2*S*,4*R*)1-acetyl-4-[(1,3-benzothiazol-6-yl)amino]-2-methyl-1,2,3,4-tetrahydroquinolin-6-yl}benzoic acid (**14**)

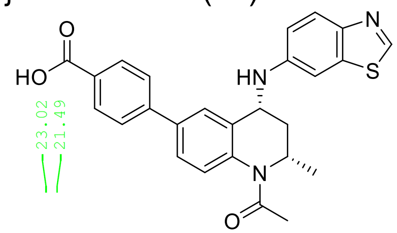


```
Current Data Parameters
NAME              Carbon
EXPNO                  5
PROCNO                 1

F2 - Acquisition Parameters
Date_           20210502
Time                1.34 h
INSTRUM           Avance
PROBHD   Z159656_0020 (
PULPROG          zgpg30
TD                 65536
SOLVENT             MeOD
NS                  1024
DS                     4
SWH            35714.285 Hz
FIDRES          1.089913 Hz
AQ             0.9175040 sec
RG                   101
DW                14.000 usec
DE                 18.00 usec
TE                 298.0 K
D1            2.00000000 sec
D11           0.03000000 sec
TD0                    1
SFO1         150.9908267 MHz
NUC1                 13C
P0                  3.33 usec
P1                 10.00 usec
PLW1         41.91400146 W
SFO2         600.4224017 MHz
NUC2                  1H
CPDPRG[2         waltz16
PCPD2              80.00 usec
PLW2         13.51200008 W
PLW12         0.30124050 W
PLW13         0.15098180 W

F2 - Processing parameters
SI                 32768
SF           150.9755171 MHz
WDW                   EM
SSB                    0
LB                  1.00 Hz
GB                     0
PC                  1.40
```

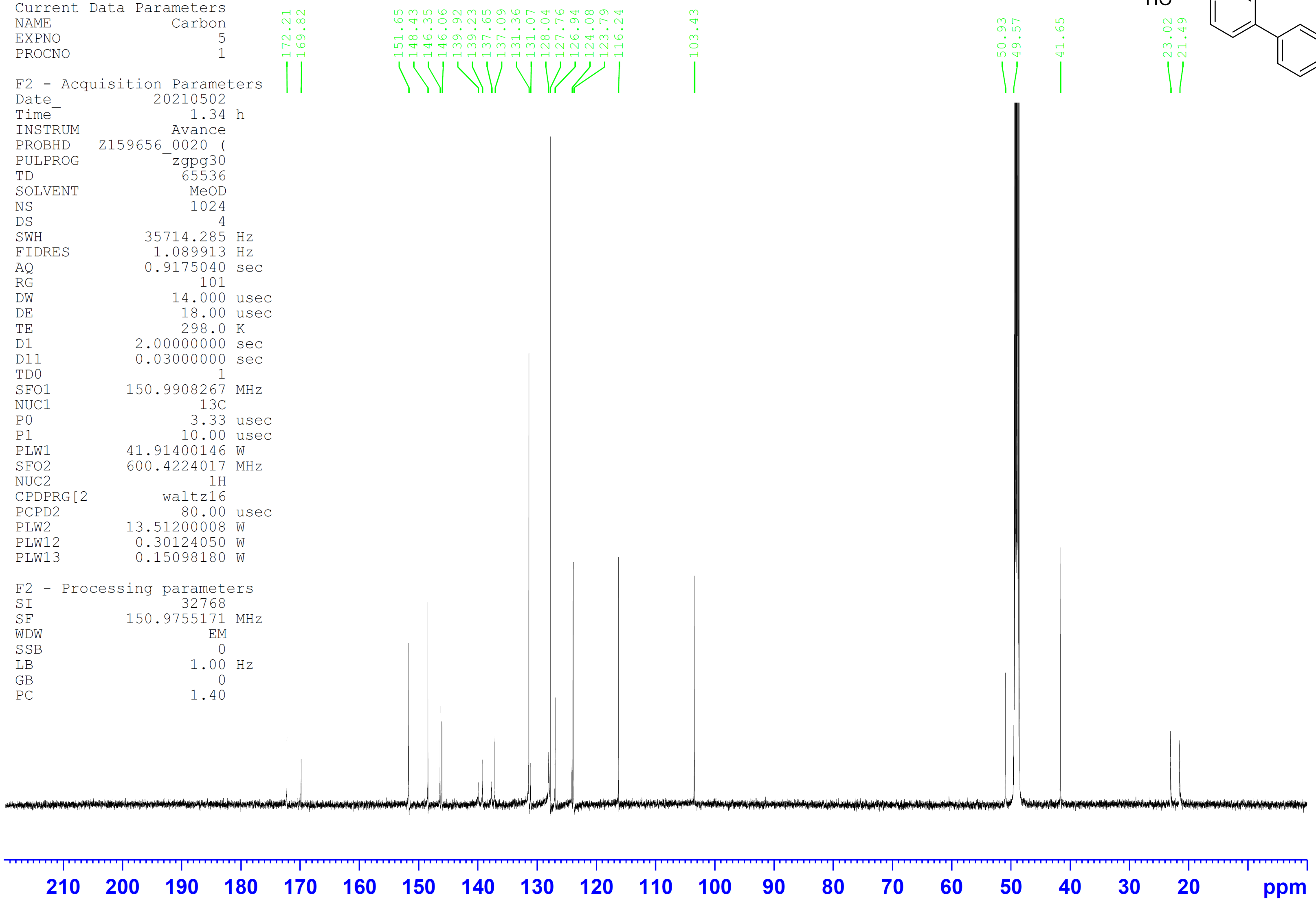

^{1}H NMR ethyl 4-{(2*S*,4*R*)-1-acetyl-2-methyl-4-[(quinolin-6-yl)amino]-1,2,3,4-tetrahydroquinolin-6-yl}benzoate (**22**)

```
Current Data Parameters
NAME                Proton
EXPNO                    1
PROCNO                   1

F2 - Acquisition Parameters
Date_             20220425
Time              10.40 h
INSTRUM             avg400
PROBHD   Z108618_0816 (
PULPROG               zg60
TD                   65536
SOLVENT              CD3CN
NS                      16
DS                       2
SWH              8012.820 Hz
FIDRES           0.244532 Hz
AQ              4.0894465 sec
RG                   91.39
DW                  62.400 usec
DE                    6.50 usec
TE                   298.0 K
D1             1.00000000 sec
TD0                      1
SFO1          400.2024012 MHz
NUC1                    1H
P1                   14.00 usec
PLW1          14.00000000 W

F2 - Processing parameters
SI                   32768
SF            400.2000113 MHz
WDW                     EM
SSB                      0
LB                    0.30 Hz
GB                       0
PC                    1.00
```

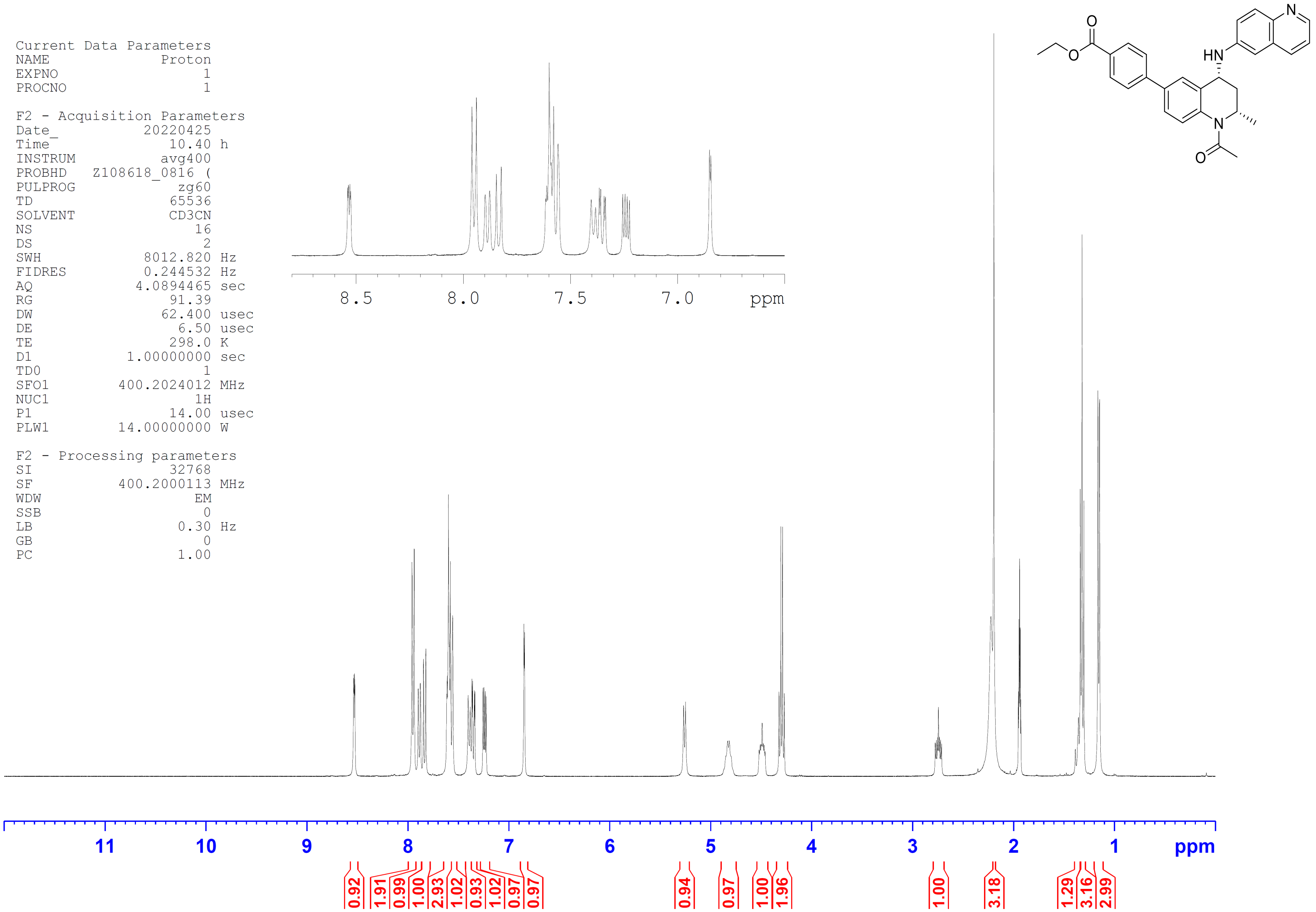

^{13}C NMR ethyl 4-{(2*S*,4*R*)-1-acetyl-2-methyl-4-[(quinolin-6-yl)amino]-1,2,3,4-tetrahydroquinolin-6-yl}benzoate (**22**)

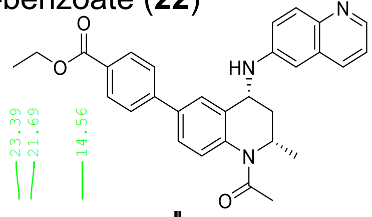

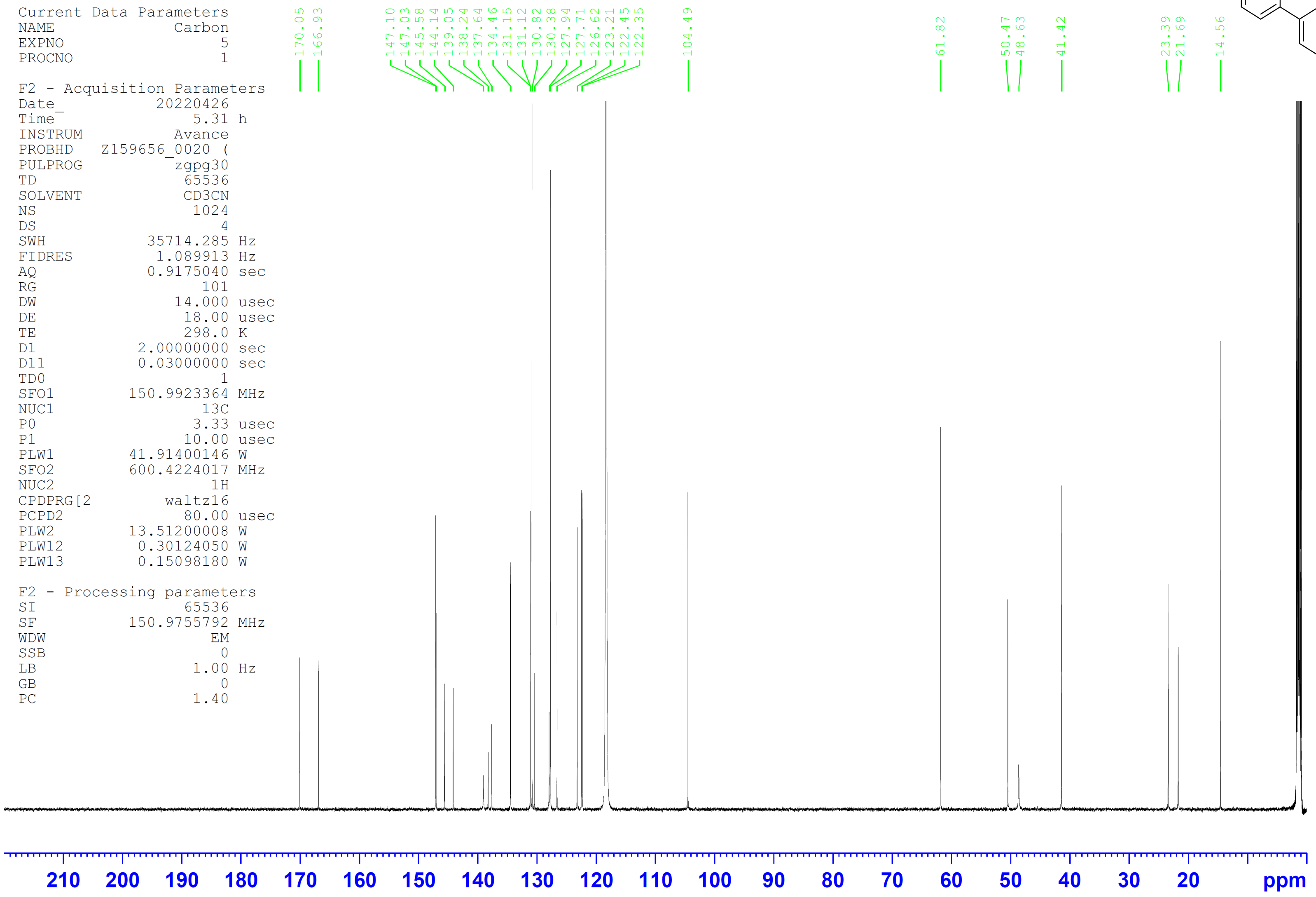

^{1}H NMR 4-{(2*S*,4*R*)-1-acetyl-2-methyl-4-[(quinolin-6-yl)amino]-1,2,3,4-tetrahydroquinolin-6-yl}benzoic acid (**15**)

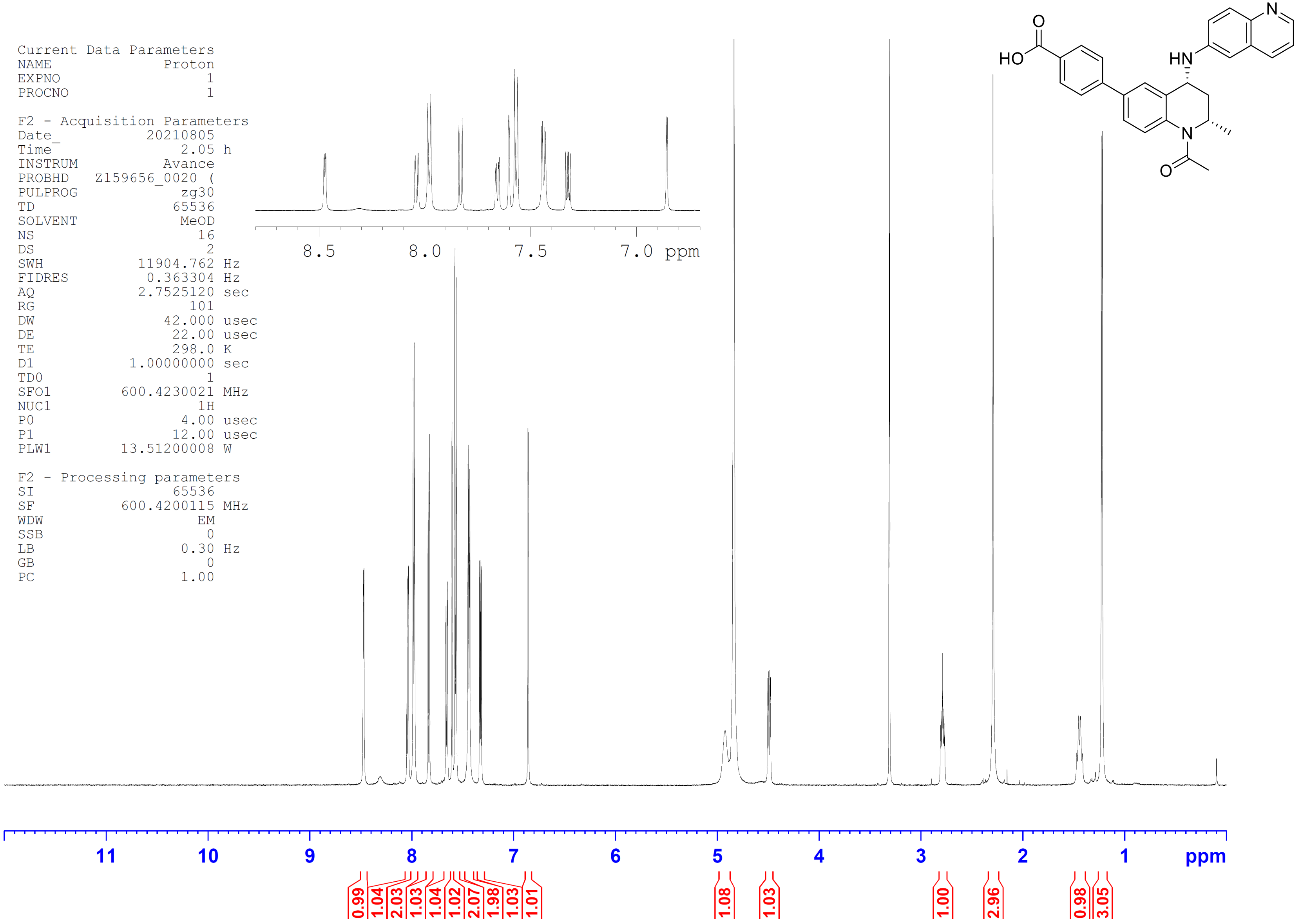

^{13}C NMR 4-{(2*S*,4*R*)-1-acetyl-2-methyl-4-[(quinolin-6-yl)amino]-1,2,3,4-tetrahydroquinolin-6-yl}benzoic acid (**15**)

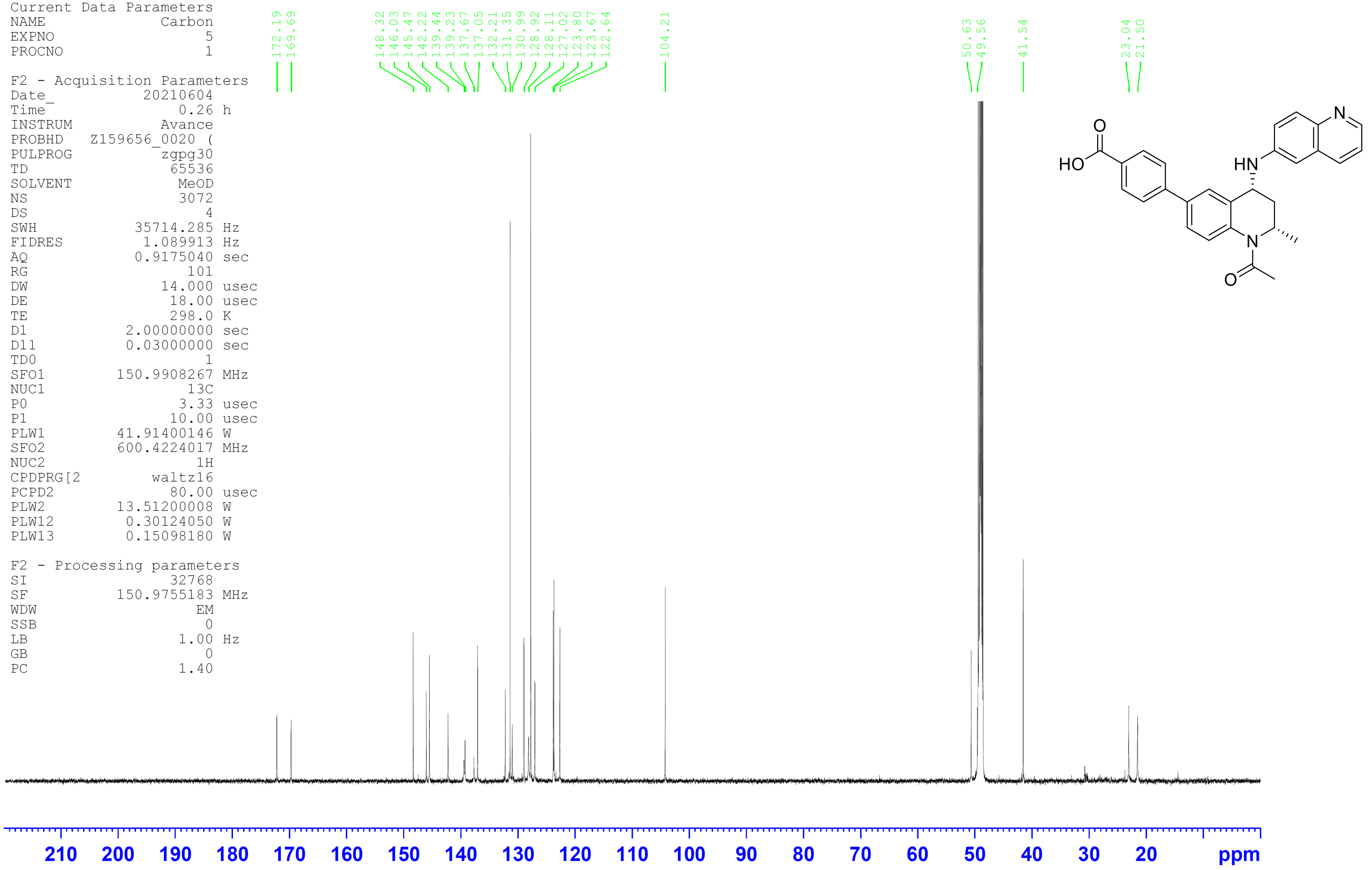

HPLC traces of biologically tested compounds

HPLC data 3-(3,5-dimethylisoxazol-4-yl)-5-(hydroxy(phenyl)methyl)phenol (**1**)

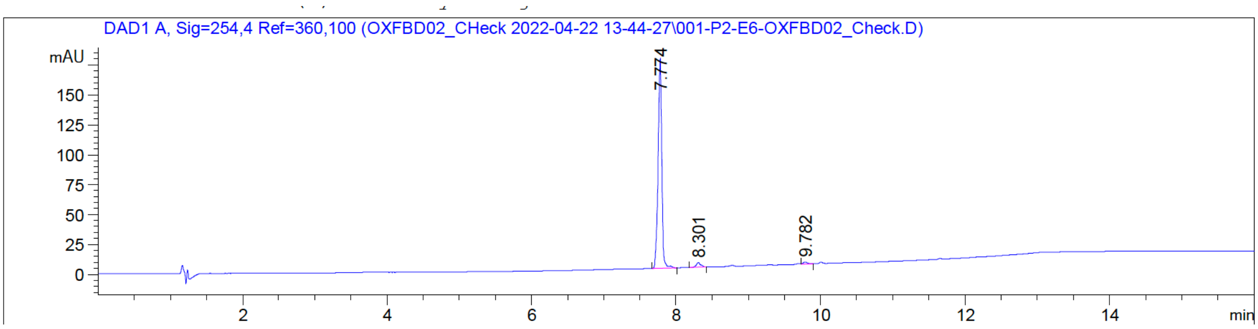

Signal 1: DAD1 A, Sig=254,4 Ref=360,100

Peak #	RetTime [min]	Type	Width [min]	Area [mAU*s]	Height [mAU]	Area %
1	7.774	BV R	0.0527	625.93250	175.22585	96.2947
2	8.301	BB	0.0620	16.89226	3.86322	2.5987
3	9.782	VB	0.0614	7.19295	1.76944	1.1066

Totals : 650.01770 180.85850

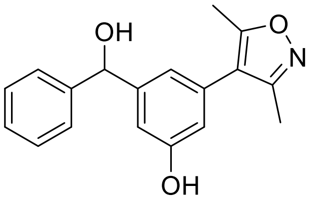

OXFBD02 (**1**)

HPLC data 3-(3,5-dimethylisoxazol-4-yl)-5-(hydroxy(phenyl)methyl)phenyl acetate (**2**)

Acquisition Method	Purity short run @254 nm
Acquisition Date/Time	8/6/2021 7:37 pm
Injection Volume	10
Sample Name	OXFBD03
Sample Description	
Batch Description	

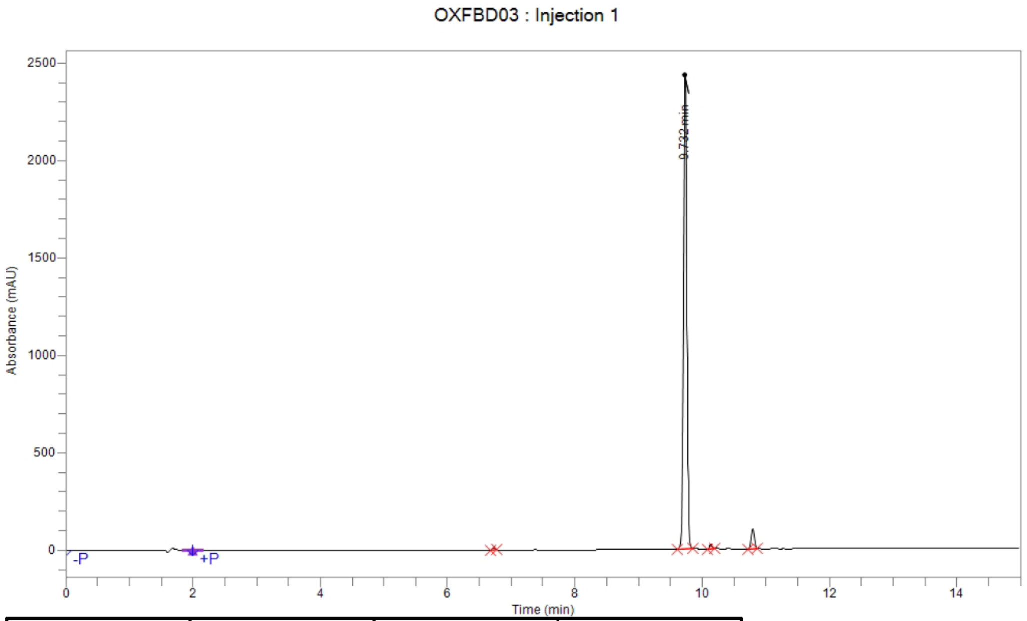

Time	Height	Area	Area %
6.721	9,489.6	27,170.4	0.28
9.732	2,437,733.8	9,113,876.6	95.41
10.139	21,669.9	65,660.8	0.69
10.795	103,878.7	345,973.9	3.62
**Total**		9,552,681.8	100.00

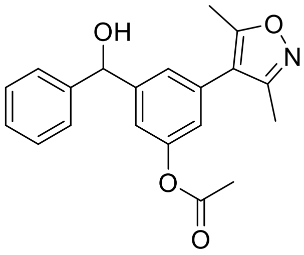

OXFBD03 (**2**)

HPLC data 3-(3,5-dimethyl-1,2-oxazol-4-yl)-5-[(4-fluorophenyl)(hydroxyl)methyl]phenol (**3**)

Acquisition Method	Purity short run @254 nm
Acquisition Date/Time	8/9/2021 5:12 pm
Injection Volume	10
Sample Name	CE_fluoro_254
Sample Description	
Batch Description	

CE_fluoro_254 : Injection 1

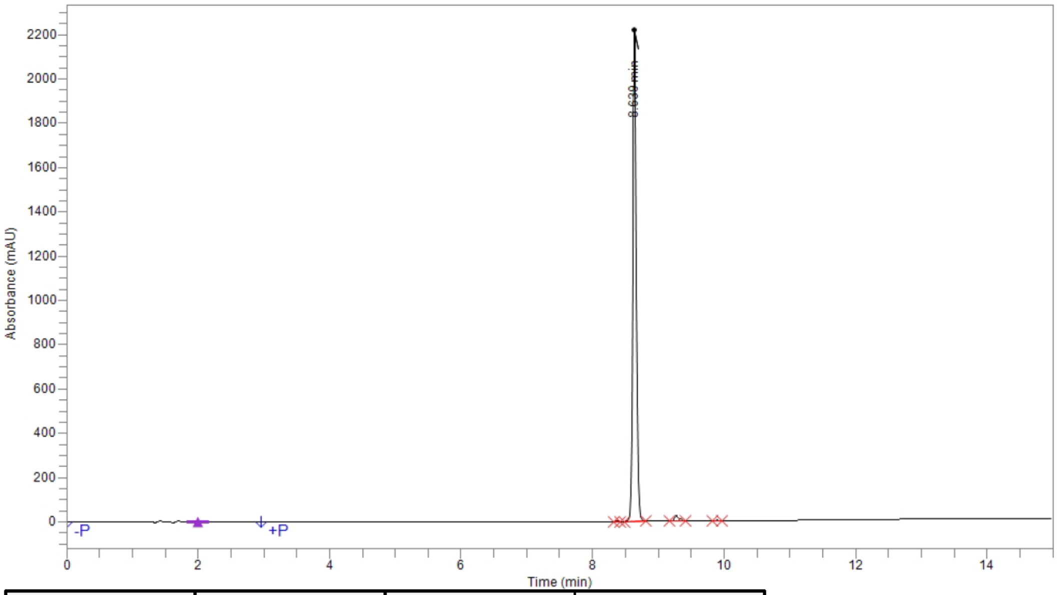

Time	Height	Area	Area %
8.374	896.2	2,655.0	0.03
8.639	2,223,007.8	8,851,577.2	98.46
9.276	24,663.8	91,640.9	1.02
9.341	10,442.9	30,140.9	0.34
9.895	3,974.7	14,103.1	0.16
**Total**		8,990,117.1	100.00

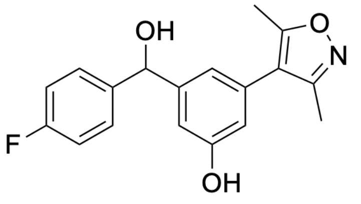

3

HPLC data 3-(3,5-dimethyl-1,2-oxazol-4-yl)-5-[hydroxy(pyridine-3-yl)methyl]phenol (**4**)

Acquisition Method	Purity short run @254 nm
Acquisition Date/Time	8/6/2021 8:12 pm
Injection Volume	10
Sample Name	DM-A-85
Sample Description	
Batch Description	

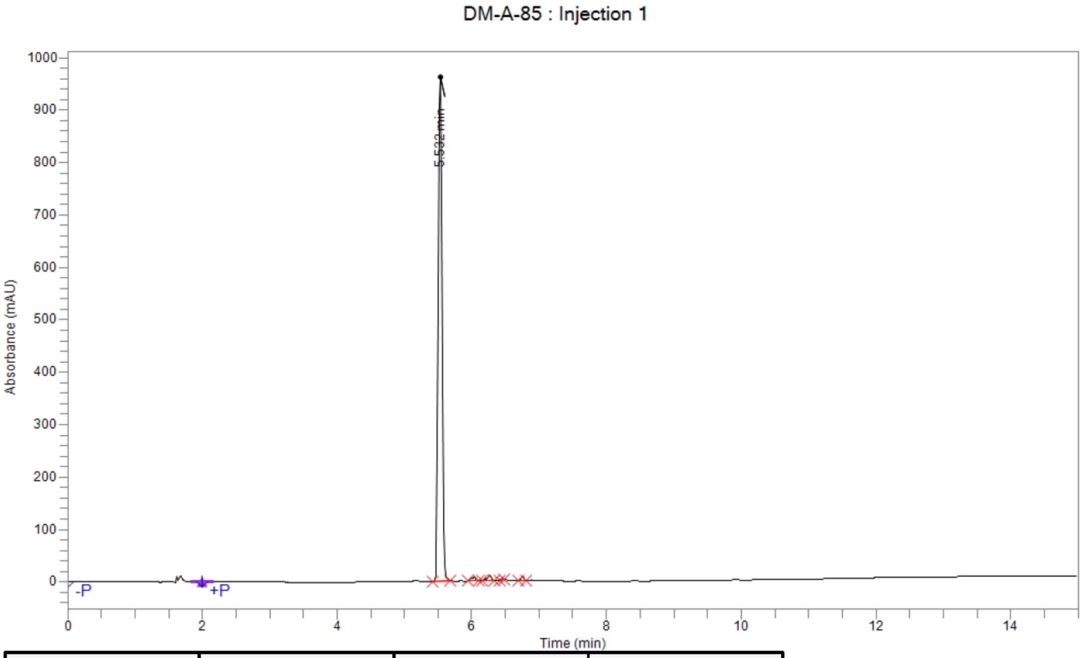

Time	Height	Area	Area %
5.532	963,220.0	3,610,657.4	97.19
6.038	7,415.8	34,196.0	0.92
6.211	4,415.7	10,973.6	0.30
6.270	11,588.8	36,623.3	0.99
6.457	1,255.1	2,546.7	0.07
6.754	7,338.2	19,876.8	0.54
**Total**		3,714,873.8	100.00

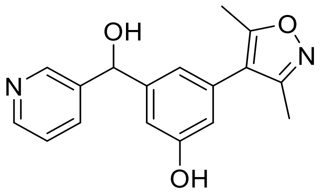

OXFBD04 (**4**)

HPLC data 4-{(2*S*,4*R*)-1-acetyl-4-[(1-benzothiophen-6-yl)amino]-2-methyl-1,2,3,4-tetrahydroquinolin-6- yl}benzoic acid (**9**)

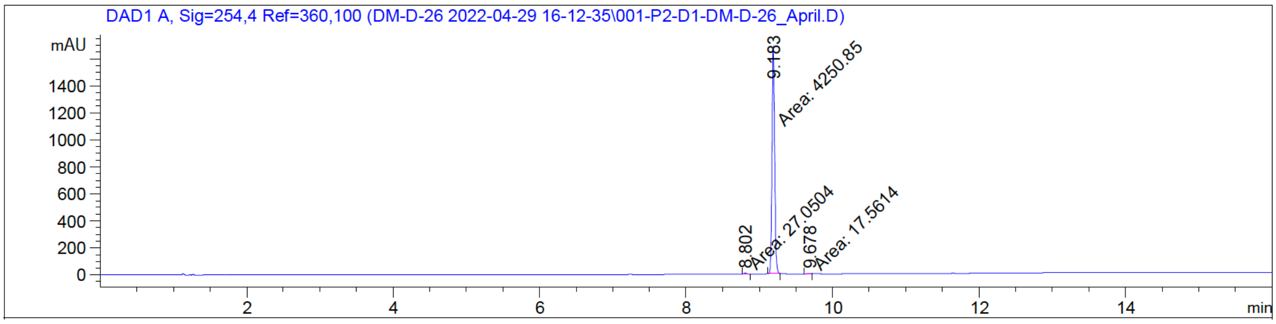

Peak #	RetTime [min]	Type	Width [min]	Area [mAU*s]	Height [mAU]	Area %
1	8.802	MM	0.0539	27.05043	8.36767	0.6297
2	9.183	MM	0.0420	4250.85156	1687.90247	98.9614
3	9.678	MM	0.0629	17.56145	4.65264	0.4088
Totals :				4295.46344	1700.92278	

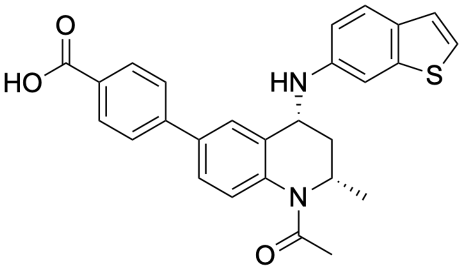

9

HPLC data 4-{(2*R*,4*S*)-1-acetyl-4-[(1-benzothiophen-6-yl)amino]-2-methyl-1,2,3,4-tetrahydroquinolin-6- yl}benzoic acid (**10**)

Acquisition Method	Purity short run @254 nm
Acquisition Date/Time	7/29/2021 1:12 pm
Injection Volume	10
Sample Name	DM-C-36_254
Sample Description	
Batch Description	

DM-C-36_254 : Injection 1

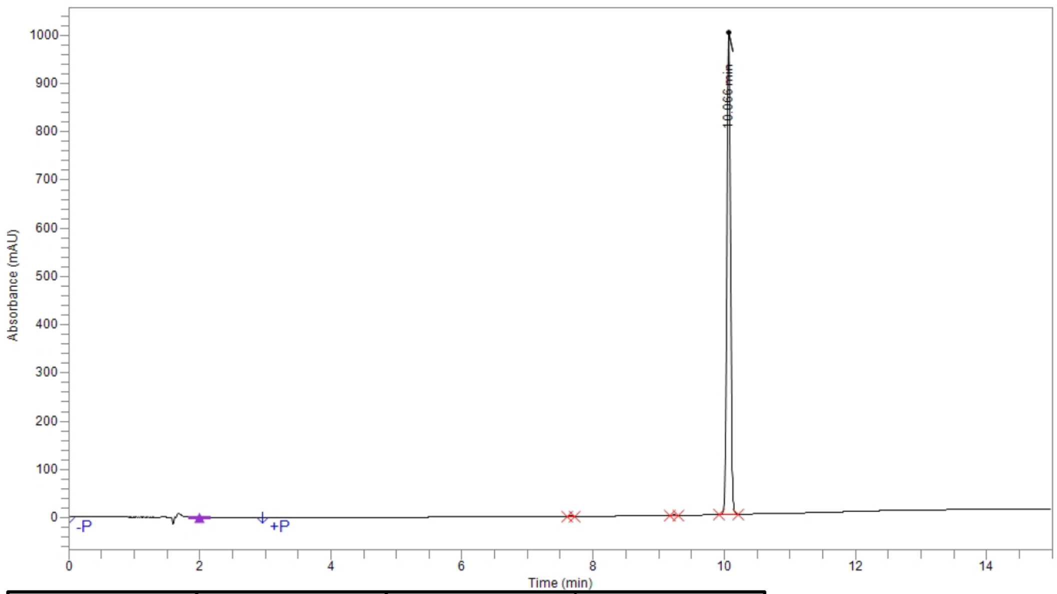

Time	Height	Area	Area %
7.660	987.6	3,444.0	0.09
9.239	1,339.7	4,672.3	0.13
10.066	1,001,222.4	3,644,262.2	99.78
**Total**		3,652,378.5	100.00

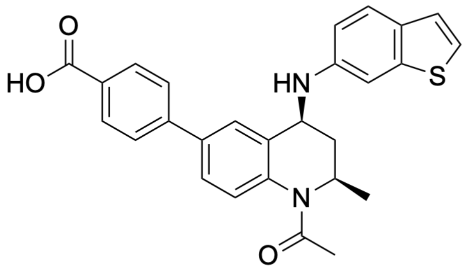

10

HPLC 4-((2*S*,4*R*)-1-acetyl-4-[(1-benzofuran-6-yl)amino]-2-methyl-1,2,3,4-tetrahydroquinolin-6-yl)benzoic acid (**11**)

Acquisition Method	Purity short run @254 nm
Acquisition Date/Time	8/2/2021 11:27 am
Injection Volume	20
Sample Name	DM-C-04_254
Sample Description	
Batch Description	

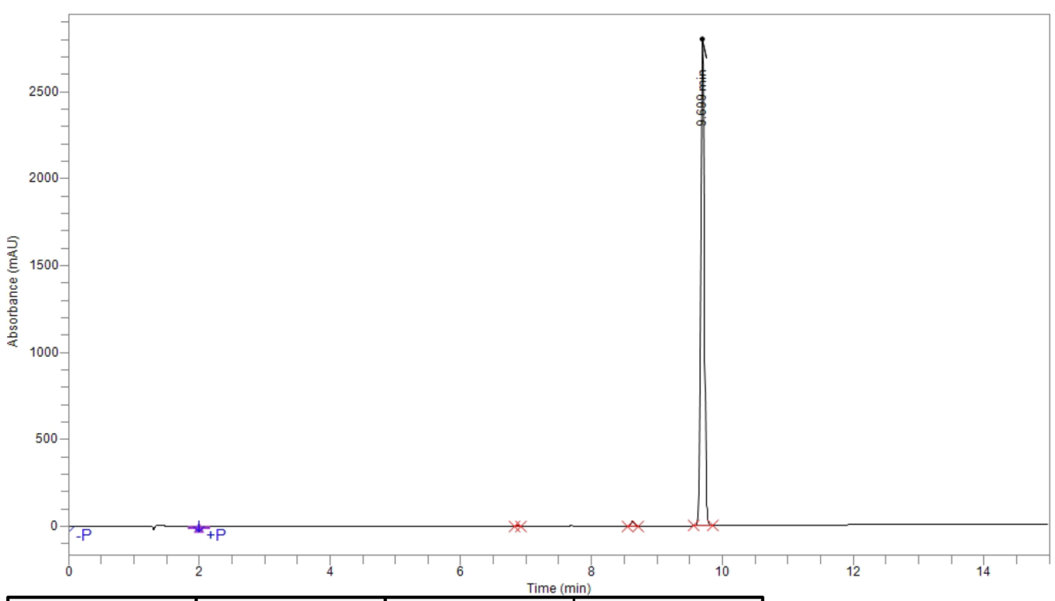

Time	Height	Area	Area %
6.873	4,367.9	12,075.5	0.12
8.629	26,999.4	106,309.6	1.02
9.699	2,805,447.7	10,312,883.1	98.87
**Total**		10,431,268.1	100.00

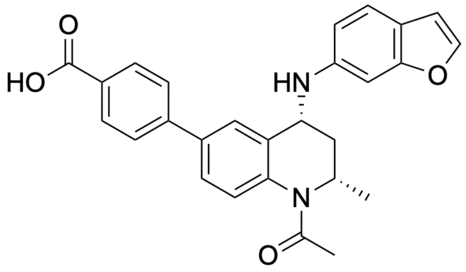

11

HPLC data 4-((2*S*,4*R*)-1-acetyl-4-[(1-benzothiophen-5-yl)amino]-2-methyl-1,2,3,4-tetrahydroquinolin-6- yl)benzoic acid (**12**)

Acquisition Method	Purity short run @254 nm
Acquisition Date/Time	7/29/2021 12:36 pm
Injection Volume	10
Sample Name	DM-C-35_254
Sample Description	
Batch Description	

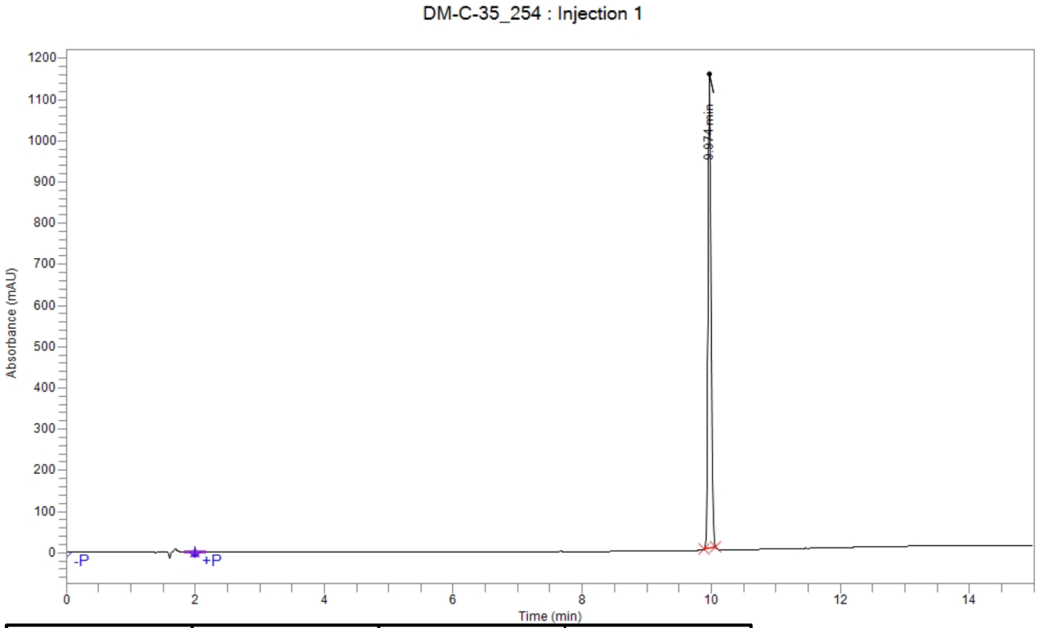

Time	Height	Area	Area %
9.974	1,150,590.9	4,025,535.8	100.00
**Total**		4,025,535.8	100.00

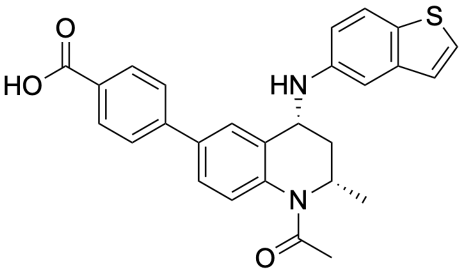

12

HPLC data 4-{(2*S*,4*R*)-1-acetyl-4-[(1*H*-indol-5-yl)amino]-2-methyl-1,2,3,4-tetrahydroquinolin-6-yl}benzoic acid (**13**)

Acquisition Method	Purity short run @254 nm
Acquisition Date/Time	4/16/2021 12:29 pm
Injection Volume	30
Sample Name	DM-C-14_RPCol_TFA_254
Sample Description	
Batch Description	

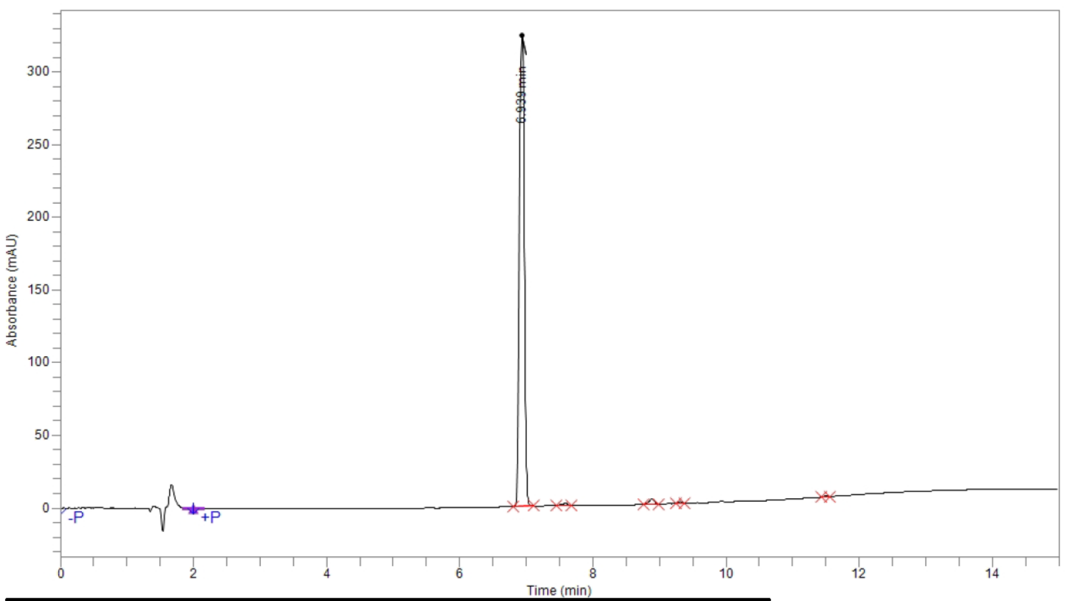

Time	Height	Area	Area %
6.939	324,479.4	1,697,874.4	97.93
7.591	1,563.8	9,865.4	0.57
8.894	3,554.7	19,742.7	1.14
9.313	1,079.0	3,916.4	0.23
11.498	623.0	2,302.2	0.13
**Total**		1,733,701.1	100.00

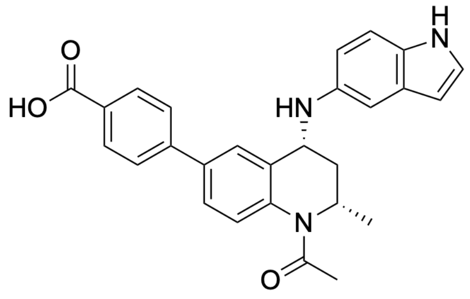

13

HPLC data 4-{(2*S*,4*R*)-1-acetyl-4-[(1,3-benzothiazol-6-yl)amino]-2-methyl-1,2,3,4-tetrahydroquinolin-6- yl}benzoic acid (**14**)

Acquisition Method	Purity short run @254 nm
Acquisition Date/Time	4/26/2021 5:06 pm
Injection Volume	10
Sample Name	DM-C-15_TFA_254
Sample Description	
Batch Description	

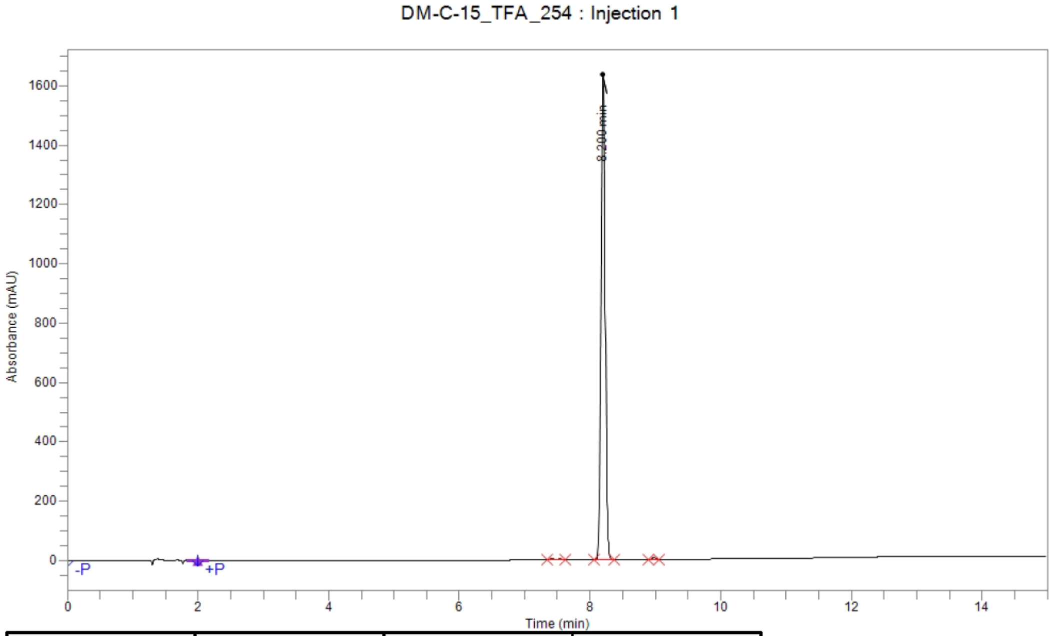

Time	Height	Area	Area %
7.417	5,023.1	20,225.8	0.28
7.544	3,939.2	15,633.4	0.22
8.200	1,637,536.5	7,065,234.4	99.14
8.975	6,745.5	25,655.1	0.36
**Total**		7,126,748.8	100.00

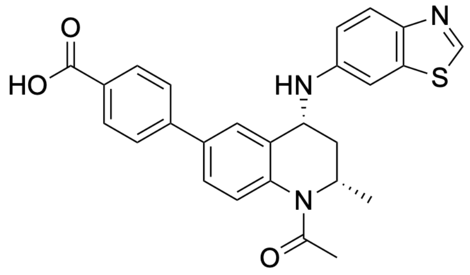

14

HPLC data 4-{(2*S*,4*R*)-1-acetyl-2-methyl-4-[(quinolin-6-yl)amino]-1,2,3,4-tetrahydroquinolin-6-yl}benzoic acid (**15**)

Acquisition Method	Purity short run @254 nm
Acquisition Date/Time	8/3/2021 11:14 am
Injection Volume	10
Sample Name	DM-C-16_254
Sample Description	
Batch Description	

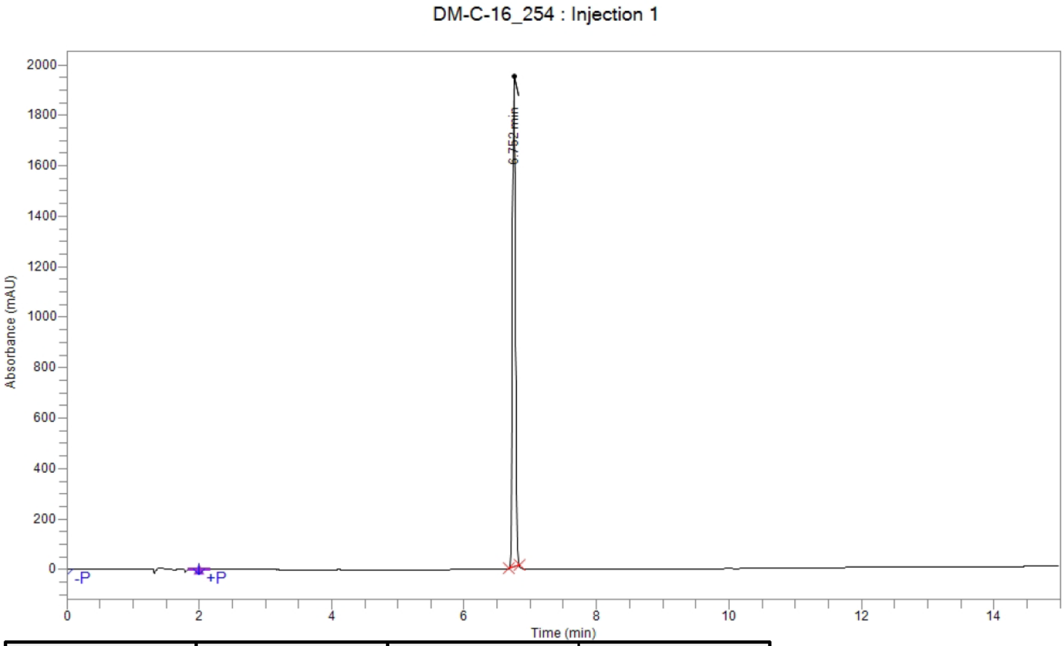

Time	Height	Area	Area %
6.752	1,950,635.1	6,319,715.2	100.00
**Total**		6,319,715.2	100.00

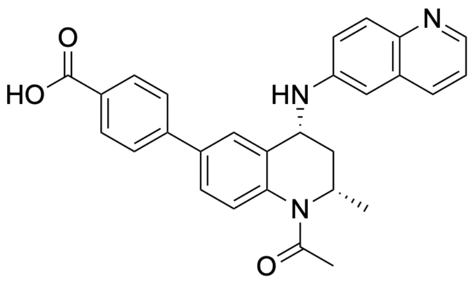

15

HPLC data ethyl ethyl 4-{(2*S*,4*R*)-1-acetyl-4-[(1-benzothiophen-6-yl)amino]-2-methyl-1,2,3,4-tetrahydroquinolin-6-yl}benzoate (**16**)

Acquisition Method	Purity short run @254 nm
Acquisition Date/Time	8/2/2021 1:19 pm
Injection Volume	10
Sample Name	DM-C-30_dil_254
Sample Description	
Batch Description	

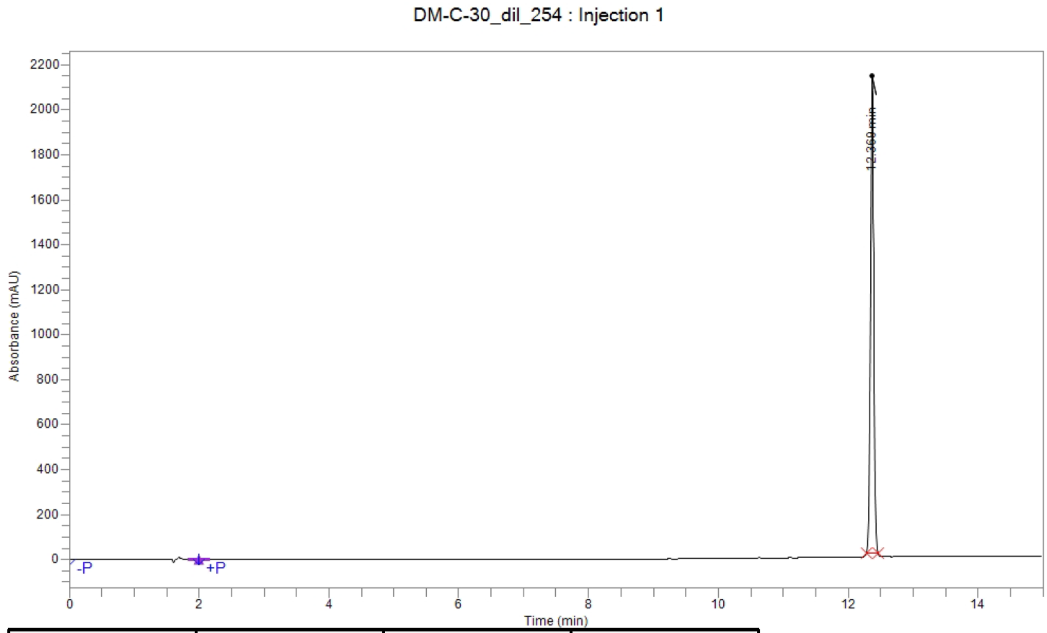

Time	Height	Area	Area %
12.369	2,124,976.7	7,960,757.9	100.00
**Total**		7,960,757.9	100.00

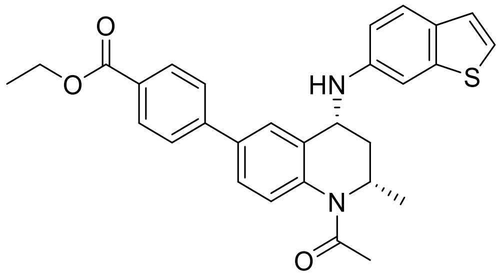

16

HPLC data ethyl 4-{(2*R*,4*S*)-1-acetyl-4-[(1-benzothiophen-6-yl)amino]-2-methyl-1,2,3,4-tetrahydroquinolin-6-yl}benzoate (**17**)

Acquisition Method	Purity short run @254 nm
Acquisition Date/Time	8/2/2021 2:15 pm
Injection Volume	10
Sample Name	DM-C-34_dil_254
Sample Description	
Batch Description	

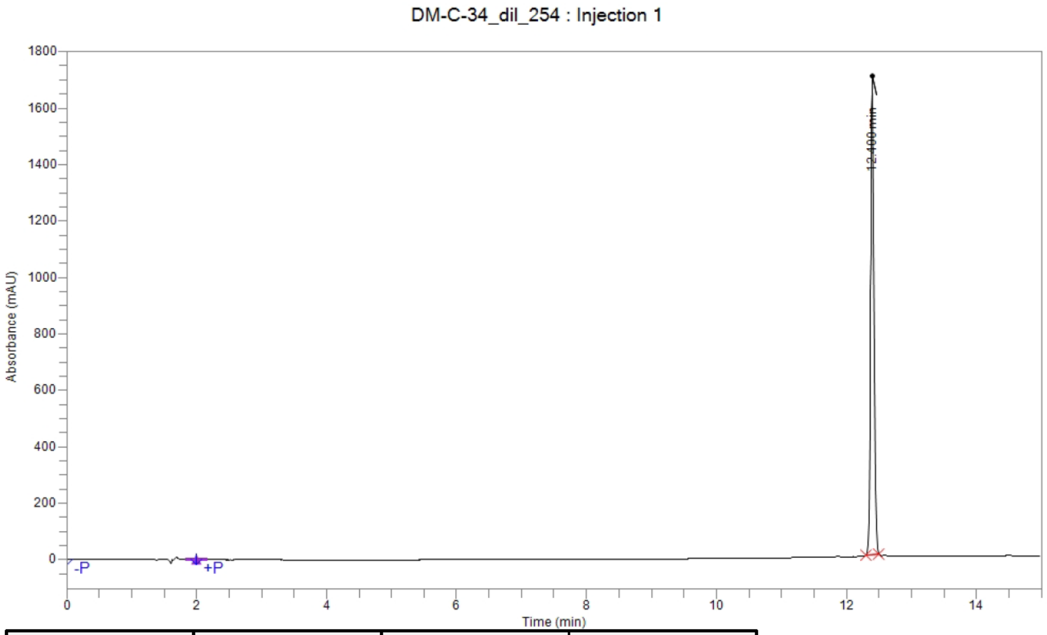

Time	Height	Area	Area %
12.400	1,697,084.8	6,441,828.6	100.00
**Total**		6,441,828.6	100.00

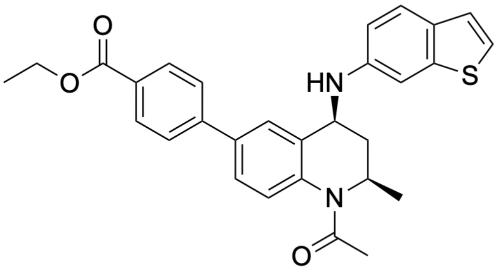

17

HPLC data ethyl 4-{(2*S*,4*R*)-1-acetyl-4-[(1-benzofuran-6-yl)amino]-2-methyl-1,2,3,4-tetrahydroquinolin-6- yl}benzoate (**18**)

Acquisition Method	Purity short run @254 nm
Acquisition Date/Time	7/30/2021 12:43 pm
Injection Volume	20
Sample Name	DM-C-38_254
Sample Description	
Batch Description	

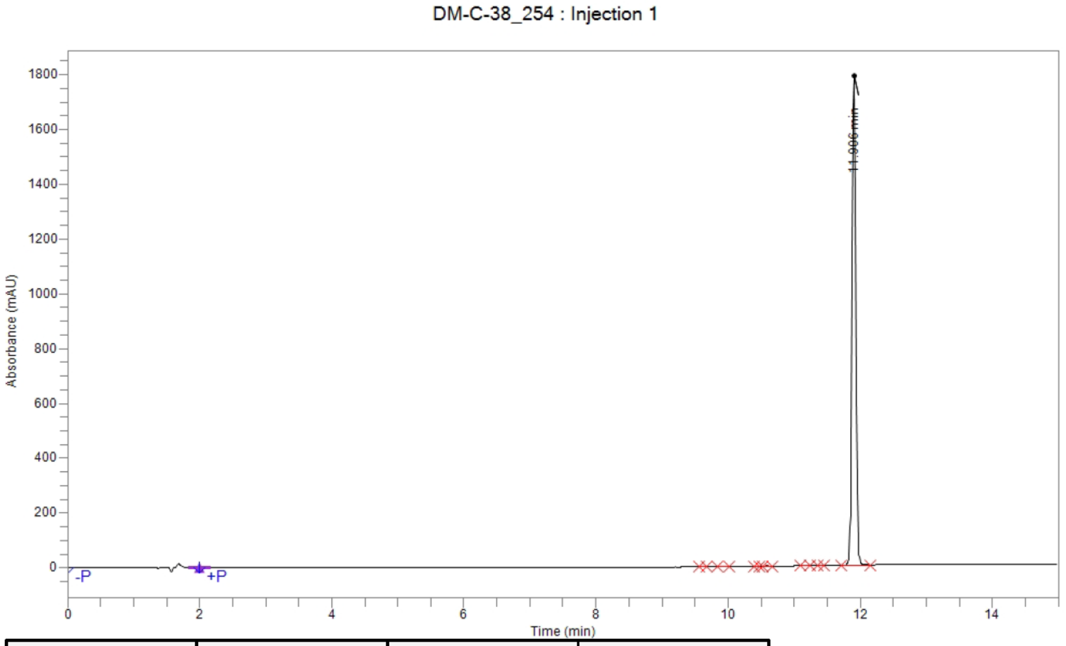

Time	Height	Area	Area %
9.625	299.2	1,395.7	0.02
9.930	1,195.7	5,165.4	0.08
10.452	130.6	477.7	0.01
10.594	1,589.5	6,002.8	0.09
11.162	1,553.5	5,675.6	0.08
11.419	463.7	1,391.3	0.02
11.906	1,789,529.3	6,775,194.1	99.70
**Total**		6,795,302.6	100.00

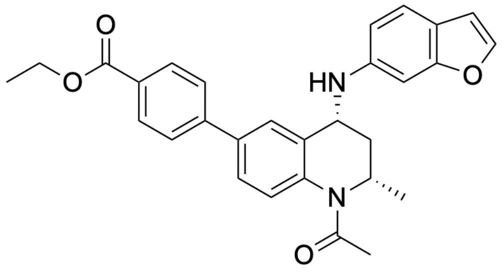

18

HPLC data ethyl 4-{(2*S*,4*R*)-1-acetyl-4-[(1-benzothiophen-5-ylamino]-2-methyl-1,2,3,4-tetrahydroquinolin-6-yl}benzoate (**19**)

Acquisition Method	Purity short run @254 nm
Acquisition Date/Time	8/2/2021 12:23 pm
Injection Volume	10
Sample Name	DM-C-29_dil_254
Sample Description	
Batch Description	

DM-C-29_dil_254 : Injection 1

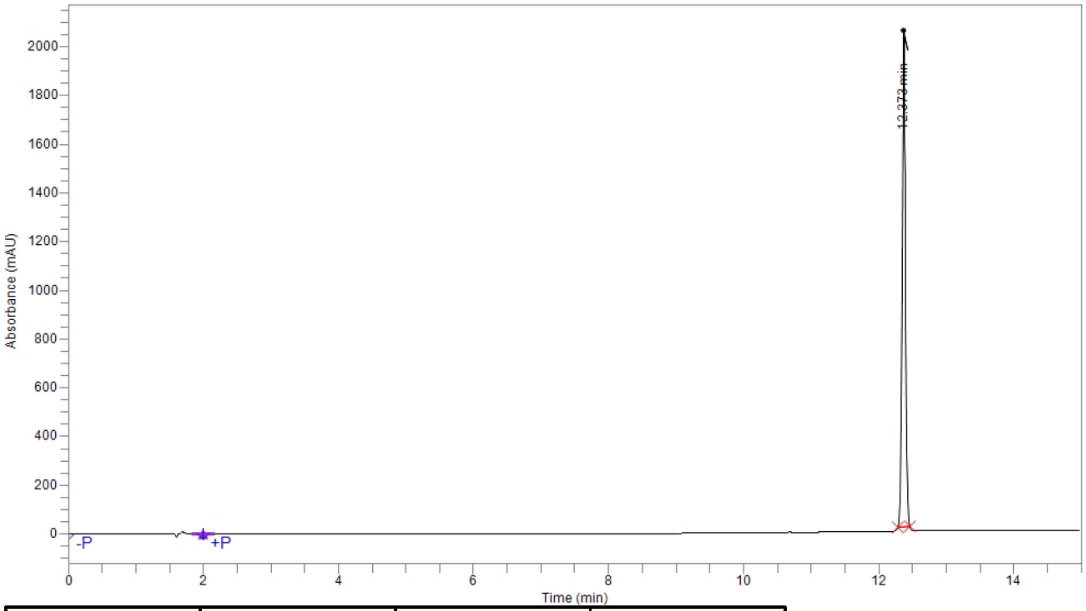

Time	Height	Area	Area %
12.373	2,041,138.1	7,599,224.0	100.00
**Total**		7,599,224.0	100.00

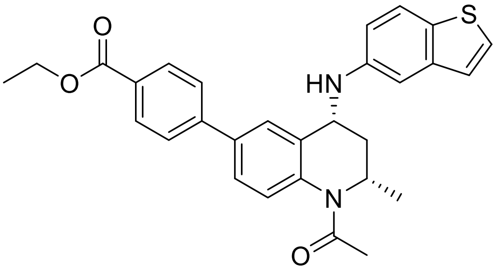

19

HPLC data ethyl 4-{(2*S*,4*R*)-1-acetyl-4-[(1*H*-indol-5-yl)amino]-2-methyl-1,2,3,4-tetrahydroquinolin-6- yl}benzoate (**20**)

Acquisition Method	Purity short run @254 nm
Acquisition Date/Time	4/2/2021 11:33 am
Injection Volume	20
Sample Name	DM-C-06_wash_254
Sample Description	
Batch Description	

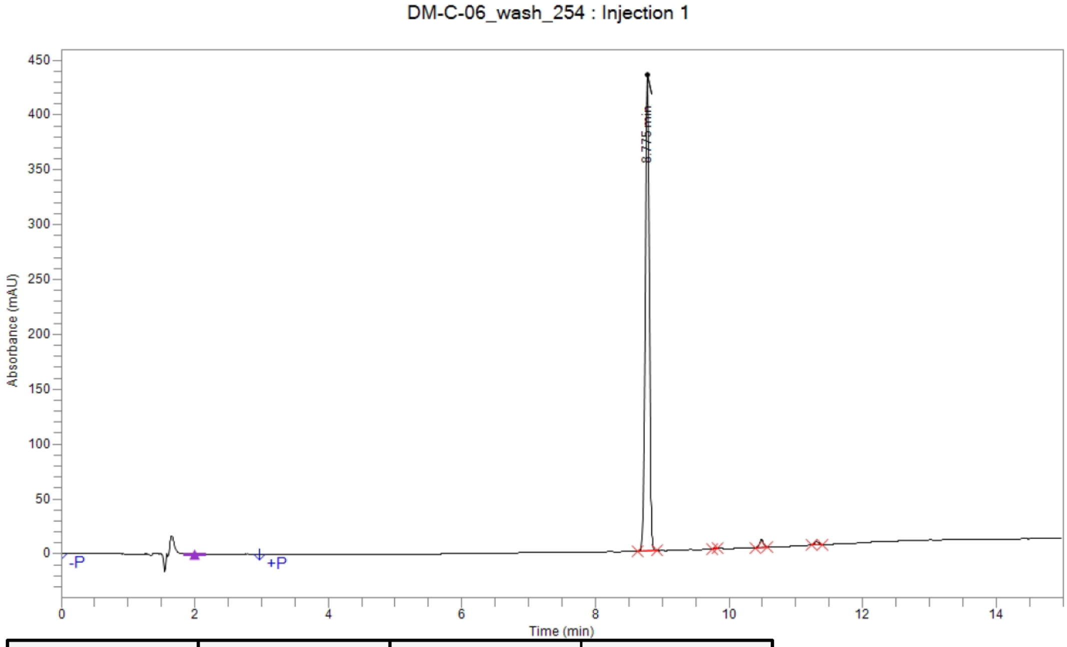

Time	Height	Area	Area %
8.775	435,039.4	1,740,246.3	97.46
9.801	467.8	1,309.8	0.07
10.487	7,430.7	31,173.1	1.75
11.313	3,123.3	12,951.6	0.73
**Total**		1,785,680.8	100.00

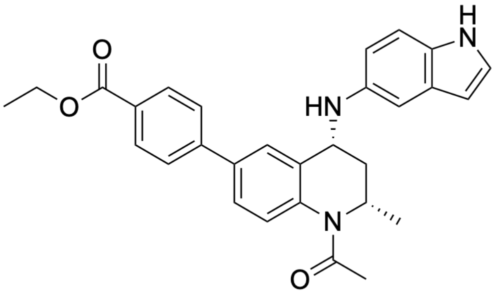

20

HPLC data ethyl 4-{(2*S*,4*R*)1-acetyl-4-[(1,3-benzothiazol-6-yl)amino]-2-methyl-1,2,3,4-tetrahydroquinolin-6-yl}benzoate (**21**)

Acquisition Method	Purity short run @254 nm
Acquisition Date/Time	3/12/2021 12:10 pm
Injection Volume	10
Sample Name	DM-B-73
Sample Description	
Batch Description	

DM-B-73 : Injection 1

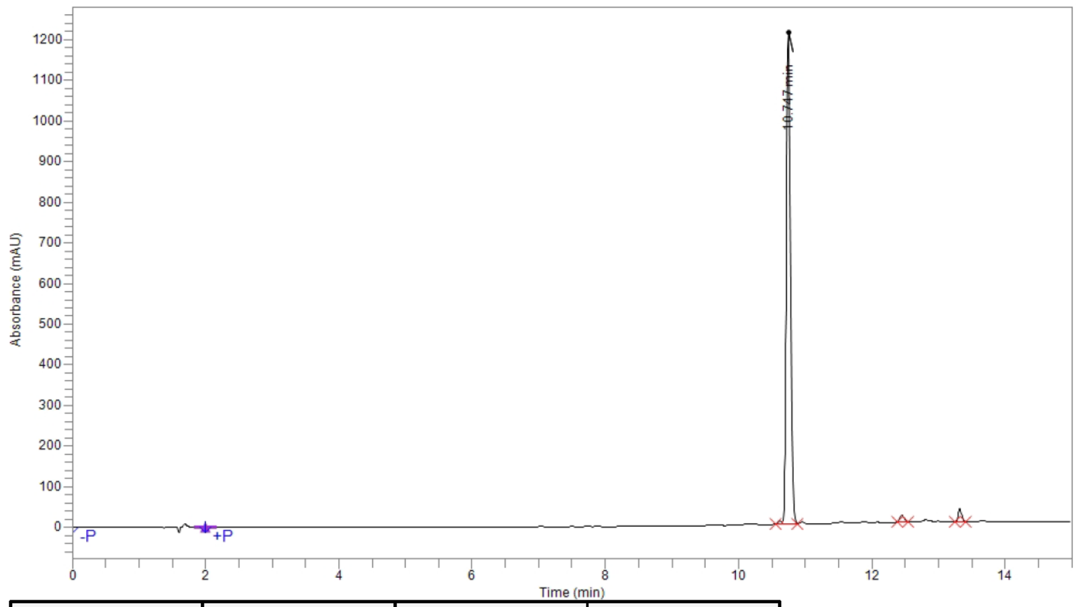

Time	Height	Area	Area %
10.622	7,317.9	23,694.3	0.44
10.747	1,211,063.6	5,157,041.3	95.98
12.454	16,594.8	74,155.0	1.38
13.323	31,272.3	117,879.9	2.19
**Total**		5,372,770.5	100.00

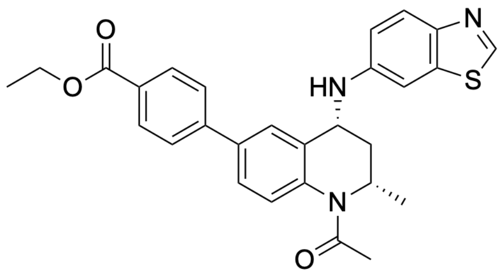

21

HPLC data ethyl 4-{(2*S*,4*R*)-1-acetyl-2-methyl-4-[(quinolin-6-yl)amino]-1,2,3,4-tetrahydroquinolin-6-yl}benzoate (**22**)

Acquisition Method	Purity short run @254 nm
Acquisition Date/Time	9/28/2020 3:19 pm
Injection Volume	10
Sample Name	DM-B-27
Sample Description	
Batch Description	

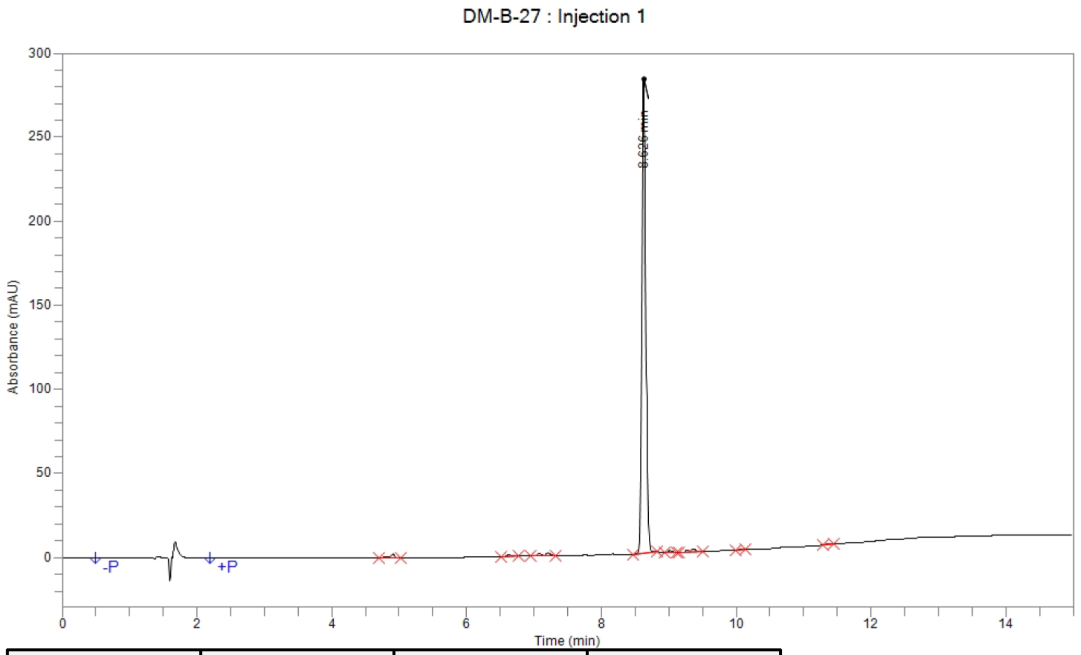

Time	Height	Area	Area %
4.908	2,391.5	13,656.7	1.08
6.632	802.8	3,777.1	0.30
7.083	1,284.6	5,017.9	0.40
7.211	1,538.7	6,397.1	0.51
8.626	282,406.3	1,208,302.0	95.79
9.017	1,152.0	5,256.9	0.42
9.273	1,535.1	6,169.8	0.49
9.366	1,956.0	8,364.1	0.66
10.063	397.5	1,722.0	0.14
11.361	601.1	2,695.2	0.21
**Total**		1,261,358.8	100.00

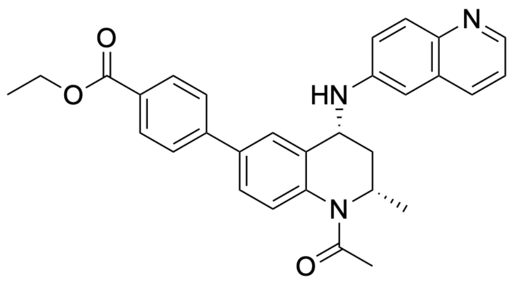

22

References

(1) Savitsky, P.; Bray, J.; Cooper, C. D. O.; Marsden, B. D.; Mahajan, P.; Burgess-Brown, N. A.; Gileadi, O. High-Throughput Production of Human Proteins for Crystallization: The SGC Experience. *J. Struct. Biol.* **2010**, *172*, 3–13. https://doi.org/10.1016/j.jsb.2010.06.008.

(2) Jumper, J.; Evans, R.; Pritzel, A.; Green, T.; Figurnov, M.; Ronneberger, O.; Tunyasuvunakool, K.; Bates, R.; Žídek, A.; Potapenko, A.; Bridgland, A.; Meyer, C.; Kohl, S. A. A.; Ballard, A. J.; Cowie, A.; Romera-Paredes, B.; Nikolov, S.; Jain, R.; Adler, J.; Back, T.; Petersen, S.; Reiman, D.; Clancy, E.; Zielinski, M.; Steinegger, M.; Pacholska, M.; Berghammer, T.; Bodenstein, S.; Silver, D.; Vinyals, O.; Senior, A. W.; Kavukcuoglu, K.; Kohli, P.; Hassabis, D. Highly Accurate Protein Structure Prediction with AlphaFold. *Nature* **2021**, *596*, 583–589. https://doi.org/10.1038/s41586-021-03819-2.

(3) Gosmini, R.; Nguyen, V.-L.; Toum, J.; Simon, C.; Brusq, J.-M. G.; Krysa, G.; Mirguet, O.; Riou-Eymard, A. M.; Boursier, E. V.; Trottet, L.; Bamborough, P.; Clark, H.; Chung, C.-W.; Cutler, L.; Demont, E. H.; Kaur, R.; Lewis, A. J.; Schilling, M. B.; Soden, P. E.; Taylor, S.; Walker, A. L.; Walker, M. D.; Prinjha, R. K.; Nicodeme, E. The Discovery of I-BET726 (GSK1324726A), a Potent Tetrahydroquinoline ApoA1 Up-Regulator and Selective BET Bromodomain Inhibitor. *J. Med. Chem.* **2014**, *57*, 8111–8131. https://doi.org/10.1021/jm5010539.

(4) Shadrick, W. R.; Slavish, P. J.; Chai, S. C.; Waddell, B.; Connelly, M.; Low, J. A.; Tallant, C.; Young, B. M.; Bharatham, N.; Knapp, S.; Boyd, V. A.; Morfouace, M.; Roussel, M. F.; Chen, T.; Lee, R. E.; Guy, R. K.; Shelat, A. A.; Potter, P. M. Exploiting a Water Network to Achieve Enthalpy-Driven, Bromodomain-Selective BET Inhibitors. *Bioorg. Med. Chem.* **2018**, *26*, 25–36. https://doi.org/10.1016/j.bmc.2017.10.042.

(5) Hewings, D. S.; Fedorov, O.; Filippakopoulos, P.; Martin, S.; Picaud, S.; Tumber, A.; Wells, C.; Olcina, M. M.; Freeman, K.; Gill, A.; Ritchie, A. J.; Sheppard, D. W.; Russell, A. J.; Hammond, E. M.; Knapp, S.; Brennan, P. E.; Conway, S. J. Optimization of 3,5-Dimethylisoxazole Derivatives as Potent Bromodomain Ligands. *J. Med. Chem.* **2013**, *56*, 3217–3227. https://doi.org/10.1021/jm301588r.

(6) Jennings, L. E.; Schiedel, M.; Hewings, D. S.; Picaud, S.; Laurin, C. M. C.; Bruno, P. A.; Bluck, J. P.; Scorah, A. R.; See, L.; Reynolds, J. K.; Moroglu, M.; Mistry, I. N.; Hicks, A.; Guzanov, P.; Clayton, J.; Evans, C. N. G.; Stazi, G.; Biggin, P. C.; Mapp, A. K.; Hammond, E. M.; Humphreys, P. G.; Filippakopoulos, P.; Conway, S. J. BET Bromodomain Ligands: Probing the WPF Shelf to Improve BRD4 Bromodomain Affinity and Metabolic Stability. *Bioorg. Med. Chem.* **2018**, *26*, 2937–2957. https://doi.org/10.1016/j.bmc.2018.05.003.

(7) Filippakopoulos, P.; Qi, J.; Picaud, S.; Shen, Y.; Smith, W. B.; Fedorov, O.; Morse, E. M.; Keates, T.; Hickman, T. T.; Felletar, I.; Philpott, M.; Munro, S.; McKeown, M. R.; Wang, Y.; Christie, A. L.; West, N.; Cameron, M. J.; Schwartz, B.; Heightman, T. D.; Thangue, N. L.; French, C. A.; Wiest, O.; Kung, A. L.; Knapp, S.; Bradner, J. E. Selective Inhibition of BET Bromodomains. *Nature* **2010**, *468*, 1067–1073. https://doi.org/10.1038/nature09504.

(8) Niesen, F. H.; Berglund, H.; Vedadi, M. The Use of Differential Scanning Fluorimetry to Detect Ligand Interactions That Promote Protein Stability. *Nat. Protoc.* **2007**, *2*, 2212–2221. https://doi.org/10.1038/nprot.2007.321.

(9) Otwinowski, Z.; Minor, W. Processing of X-Ray Diffraction Data Collected in Oscillation Mode. *Methods Enzymol.* **1997**, *276*, 307–326. https://doi.org/10.1016/s0076-6879(97)76066-x.

(10) Clabbers, M. T. B.; Gruene, T.; Parkhurst, J. M.; Abrahams, J. P.; Waterman, D. G. Electron Diffraction Data Processing with DIALS. *Acta Crystallogr. Sect. D Struct. Biology* **2018**, *74*, 506–518. https://doi.org/10.1107/s2059798318007726.

(11) McCoy, A. J.; Grosse-Kunstleve, R. W.; Adams, P. D.; Winn, M. D.; Storoni, L. C.; Read, R. J. Phaser Crystallographic Software. *J. Appl. Crystallogr.* **2007**, *40*, 658–674. https://doi.org/10.1107/s0021889807021206.

(12) Emsley, P.; Lohkamp, B.; Scott, W. G.; Cowtan, K. Features and Development of Coot. *Acta Crystallogr. Sect. D* **2010**, *66*, 486–501. https://doi.org/10.1107/s0907444910007493.

(13) Afonine, P. V.; Grosse-Kunstleve, R. W.; Echols, N.; Headd, J. J.; Moriarty, N. W.; Mustyakimov, M.; Terwilliger, T. C.; Urzhumtsev, A.; Zwart, P. H.; Adams, P. D. Towards Automated Crystallographic Structure Refinement with Phenix.Refine. *Acta Crystallogr. Sect. D* **2012**, *68*, 352–367. https://doi.org/10.1107/s0907444912001308.
